# Scientific Program 2012 ISEV meeting Wednesday 18th April

**DOI:** 10.3402/jev.v1i0.18182

**Published:** 2012-04-16

## Opening Session          10.00-11.00

**10.00-10.10 Welcome, Jan Lötvall, meeting chair and Olle Larkö, Dean Sahlgrenska Academy**

**10.10-10.20 How ISEV was established**. *Clotilde Théry and Jan Lötvall*

**10.20-10.50 Fifty years of extracellular vesicles research**. *Douglas Mulhall*

**10.50-11.00 NIH reaches out to the ISEV community**. *Elizabeth Wilder, NIH*

## Symposium Session 1          11.00-12.00

### Extracellular Vesicles as Communication Messages in biology

Production and release of exosomes and extracellular vesicles. *Willem Stoorvogel*

Exosome-based RNAi therapeutics for neurological disease, *Matthew Wood*

The importance an implications of RNA in extracellular vesicles, *Xandra Breakfield*

## Lunch and Poster Viewing          12.00-13.00

## Symposium Session 2          14.00-15.30

### 1. Production and Recovery

**The ESCRT machinery plays a critical role in the biogenesis of exosomes/microvesicles**

Stephen J. Gould

Department of Biological Chemistry, The Johns Hopkins University School of Medicine, Baltimore, Maryland, USA

Email: sgould@jhmi.edu

The ESCRT (endosomal sorting complexes required for transport) machinery is required for several cell biological processes that involve outward vesicle budding (outward = away from the cytoplasm). These include the biogenesis of multivesicular bodies (MVBs) and the budding of HIV and other retroviruses. While it was initially anticipated that the ESCRT machinery would also be essential for the formation of exosomes and microvesicles, previous studies have reported that ESCRT dysfunction does not impair the budding of exosomes/microvesicles. Here, we report that the late-acting ESCRT-associated ATPase, VPS4, interacts with a wide variety of exosome/microvesicle cargo proteins, is required for the budding of some cargoes but inhibits the budding of others. These and other data suggest a model in which VPS4/ESCRTs alters the oligomeric state of exosome/microvesicle cargo proteins at before the point of cargo selection and, thereby, modulates the targeting of proteins to secreted vesicles.

Funded by: NIH, USA

**The intracellular interactome of tetraspanin-enriched microdomains reveals their function as sorting machineries to exosomes**

María Yáñez-Mó^1^, Daniel Perez-Hernandez^2,3^, Cristina Gutiérrez-Vázquez^3^, Soraya López-Martín^1^, Angeles Ursa^4^, Inmaculada Jorge^3^, Francisco Sánchez-Madrid^3,4^ and Jesús Vázquez^2,3^

^1^Unidad de Investigación Hospital Santa Cristina, Hospital de la Princesa, Instituto de Investigación Sanitaria Princesa (IIS-IP), Madrid, Spain; ^2^Laboratory of Protein Chemistry and Proteomics, Centro de Biología Molecular “Severo Ochoa” (CSIC-UAM), Madrid, Spain; ^3^Department of Vascular Biology and Inflammation, Centro Nacional de Investigaciones Cardiovasculares, Madrid, Spain; ^4^Servicio de Inmunología, Hospital de la Princesa, Instituto de Investigación Sanitaria Princesa (IIS-IP), Madrid, Spain

Email: myanez.hlpr@salud.madrid.org

Tetraspanin-enriched microdomains (TEM) are specialized membrane platforms with key roles in cell adhesion and migration. They might also be fundamental to connect receptors with specific signaling cascades and the cytoskeleton. Using high-throughput mass-spectrometry, we have systematically characterized the intracellular ligands of TEM proteins in human lymphoblasts and their derived exosomes, revealing a clear pattern of interaction networks. Proteins interacting with TEM receptors in cytoplasmic regions presented a considerable degree of overlap, although some highly specific CD81 tetraspanin ligands, such as Rac GTPase, were detected. TEM interactome in exosomes accounted for about 45% of the protein content of the shedded microvesicles. Quantitative proteomic analyses revealed that a selective repertoire of CD81-associated molecules, including Rac, was diminished in the exosomes derived from CD81-deficient animals. Our data provide a firm evidence that insertion into TEMs is critical for the inclusion of these ligands into exosome structure.

Funded by: Grants PI080794 and PI11/01645 from the Instituto de Salud Carlos III to M.Y.-M.; SAF2011-25834 and INSINET-0159/2006 from the Comunidad de Madrid to F.S.M; grants BIO2009-07990 from the Ministerio de Educación y Ciencia, CAM BIO/0194/2006 from Comunidad de Madrid, and RECAVA RD06/0014 from the Fondo de Investigaciones Sanitarias (Ministerio de Sanidad y Consumo, Instituto Salud Carlos III) to J.V. and F.S.M.

**miR-1289 and “zipcode”-like sequence enrich mRNAs in microvesicles**

Mehmet Fatih Bolukbasi, Arda Mizrak, Gokhan Baris Ozdener, Sibylle Madlener, Thomas Ströbel, Erdogan Pekcan Erkan, Jian-Bing Fan, Xandra O. Breakefield and Okay Saydam

Molecular Neuro-Oncology Research Unit, Department of Pediatrics, Medical University of Vienna, Austria

Email: okay.saydam@meduniwien.ac.at

Despite intensive studies, the molecular mechanisms by which the genetic materials are uploaded into microvesicles are still unknown. This is the first study describing a zipcode-like 25-nucleotide sequence in the 3'UTR of mRNAs, with variants of this sequence present in many mRNAs enriched in microvesicles, as compared to their glioblastoma cells of origin. When this sequence was incorporated into the 3'UTR of a reporter message and expressed in a different cell type, it led to enrichment of the reporter mRNA in microvesicles. Critical features of this sequence are both a CUGCC core presented on a stem loop structure and a miRNA binding site, with increased levels of the corresponding miRNA in cells further increasing levels of mRNAs in microvesicles.

Funded by: NIH NCI grant CA141150 (X.O.B.), NIH NINDS grant NS037409 (X.O.B., O.S.), Forschungsgesellschaft for Brain Tumors (O.S.).

**Characterization of microvesicles released from cells constitutively and upon stimulation**

Dan Stratton^1^, Samuel Antwi-Baffour^1,2^, Gareth Williams^1^, Ryan Grant^1^, Sigrun Lange^3^ and Jameel Inal^1^

^1^Cellular and Molecular Immunology Research Centre, School of Human Sciences, Faculty of Life Sciences, London Metropolitan University, London, United Kingdom; ^2^Immunohaematology Research Centre, School of Allied Health Sciences, University of Ghana, Ghana; ^3^University College London, Institute for Women's Health, Maternal and Fetal Medicine, London,United Kingdom

Email: d.stratton@londonmet.ac.uk

Constitutively released microvesicles (cMVs) are released as a part of normal cell physiology. However, stimulated microvesicles (sMVs) are released as a result of a number of possible stress factors. We found sMVs to be released in higher numbers than cMVs, typically 10-fold higher numbers, in the same time frame, and where the stress factor was a pharmacological agent, the microvesiculation was an attempt to release this chemical stress factor. Using a mass sensing technique, the sMVs were released over a 15-minutes period after stimulation. Using sizing beads on a flow cytometer and by transmission electron microscopy, the cMVs were typically smaller (less than 300 nm in diameter) than sMVs (300–500 nm in diameter). However, cMVs were found to carry more protein. By contrast, phosphatidylserine expression was greater on the larger sMVs, which also more effectively inhibited complement-mediated lysis.

Funded by: The Cellular and Molecular Immunology Research Centre, LMU's VC scholarship to R.G.

**Myotube but not myoblast secretes exosomes**

Ronne Wee Yeh Yeo^1,2^ and Sai Kiang Lim^1^

^1^Institute of Medical Biology, A*STAR, Singapore; ^2^National University of Singapore Graduate School for the Integrative Sciences and Engineering, Singapore

Email: ronne.yeo@imb.a-star.edu.sg

Cells are known to secrete many types of membrane microparticles and the types and composition depend on the cell type, cellular microenvironment or the pathophysiological conditions of the cell. To date, several types of microparticles (e.g., microvesicles, exosomes, ectosomes and apoptotic bodies) have been identified. While the functions of these different microparticles have not been elucidated, they are known to contain both proteins and RNAs that had biological activities to either enhance cellular physiology or exacerbate pathogenesis. It was previously shown that human myotubes release membrane microparticles with an average diameter of 150 nm in response to an increase in intracellular Ca^2 +^ concentration. However, little is known about the characteristics of muscle-derived membrane microparticles (MMMs) and their biological functions. In this study, we aim to characterize MMMs using a clonal MycHSMM cell line derived by Myc immortalization of human primary skeletal muscle myoblasts. The immortalized cell line has a normal karyotype, has an accelerated rate of proliferation and retains its ability to elongate and fuse with adjacent cells to form multinucleated myotubes that twitch spontaneously in culture. These myotubes stained positively for markers of differentiated skeletal muscle such as myogenin, desmin, dystrophin and myosin heavy chain 2. In addition, we observed that mature myotubes but not myoblasts secrete MMMs. The MMMs were positive for GM1, CD9 and CD81, suggesting that they were exosomes. The flotation density, diameter and proteome are being assessed to confirm if the MMMs are exosomes. Further work to explore the biological significance of these MMMs in cell-cell signaling and skeletal muscle pathologies such as muscular dystrophy and insulin resistance are presently underway.

Funded by: A*STAR Singapore

### 2. Tissue Injury and Repair

**Procoagulant microparticles are elevated in acute traumatic injury**

N.S. Curry^1^, A. Raja^1^, K. Brohi^2^, S.J. Stanworth^1^ and P. Harrison^1,2^

^1^National Health Service Blood & Transplant/Haematology, John Radcliffe Hospital, Oxford, United Kingdom; ^2^Department of Trauma, Vascular Surgery & Critical Care, Royal London Hospital, London, United Kingdom; ^3^Oxford Haemophilia and Thrombosis Centre, Churchill Hospital, Oxford,United Kingdom

Email: paul.harrison@ndm.ox.ac.uk

*Background*: There is increasing interest in the influence of microparticles (MP) on coagulation and inflammatory responses in trauma. To characterise these procoagulant MP, blood samples were collected from 50 consecutive trauma patients within 2 hours of injury. These patients formed a subset of a larger study (Activation of Coagulation & Inflammation in Trauma-2). *Methods*: Blood was drawn into citrated 0.109 M vacutainers at hospital admission. Platelet-free plasma (PFP) was prepared within 60 minutes of collection by centrifugation at 2000g for 20 minutes and 13,000g for 2 minutes. Aliquots were frozen at −800°C. Samples were analyzed by the procoagulant phospholipid (PPL) assay (STA-Procoag-PPL, Stago Diagnostics), thrombin generation testing (TGT) (1 pM tissue factor, PRP-reagent, Stago Diagnostics) and flow cytometry (LSR II) to determine MP size, number and cellular origin using marker-specific antibodies. Twenty normal controls were analyzed for comparison. *Results*: Mean PPL results were 81.2 seconds (s) for controls and 50.8 s for patients (*p*<0.001). Mean TGT results were higher for patients than controls: ETP 1741.5 nM vs. 1008.5 nM; peak 170.3 nM vs. 60.0 nM (*p* values < 0.001). PPL results and TGT were significantly inversely correlated: PPL:ETP *r*= − 0.60 and PPL:Peak *r*= − 0.67 (*p*<0.001). Flow cytometry confirmed significantly increased numbers of CD41/annexin V and CD235a/annexin V dual positive cells (*p* values < 0.001) compared to controls. No significant difference was detected for leukocyte, monocyte or endothelial cell-derived microparticles. Admission ETP values (mean ETP = 1373.2 nM, *p*=0.007) and numbers of CD41/annexin V positive microparticles were significantly lower in trauma patients who died (median = 114.4 per µl) compared to patients who survived (median = 200.4 per µl) to day 28. Those patients who died within 12 hours had the lowest numbers of CD41/annexin V positive MP (median = 77.3 per µl) compared to those who died after 24 hours (median = 137.4 per µl). *Conclusion:* Acute trauma results in a significant increase in circulating procoagulant microparticles within 2 hours of injury. Flow cytometry confirms that these are annexin V-positive platelet and red cell microparticles. Lower numbers of procoagulant particles are found in patients who died, a result that requires further investigation.

Funded by: NHS Blood and Transplant

**Paracrine miRNA-crosstalk via exosomes between cardiac fibroblasts and cardiomyocytes**

C. Bang, S. Dangwal, A. Zeug and T. Thum

^1^Institute of Molecular and Translational Therapeutic Strategies (IMTTS), Hannover Medical School, Germany; ^2^Institute for Neurophysiology, Hannover Medical School, Germany

Email: bang.claudia@mh-hannover.de

Recent reports have shown that miRNAs are actively secreted in microvesicles/exosomes from different cell types highlighting their potential to serve as paracrine signaling molecules between cells. Whether secreted extracellular miRNAs may serve as cell-cell communicators in fibroblast-derived cardiomyocyte hypertrophy is unknown. We isolated secreted exosomes from supernatants of primary cardiac fibroblasts by ultracentrifugation. Using a miRNA-transcriptome profiling assay (388 rat miRNAs), we identified 22 detectable miRNAs in fibroblast-derived exosomes, which we termed cardiac fibroblast-secreted miRNAs (CF-sec.miR). The secretion of CF-sec.miRNAs was regulated by neutral sphingomyelinase 2 (nSMase 2), which is expressed in rat cardiac fibroblasts. Inhibition of nSMase 2 by a chemical inhibitor resulted in intracellular accumulation of CD63 + microparticles and a pronounced reduction of CF-sec.miRs. Treatment with the prohypertrophic agent angiotensin II stimulated the secretion of investigated miRNAs. Further, cardiac fibroblast-derived exosomes are taken up by cardiomyocytes, indicating a possible novel role of exosomes enriched with miRNAs to function as paracrine signaling mediators. Indeed, fibroblast-derived miR-21* is transported to cardiomyocytes and directly led to the development of cardiomyocyte hypertrophy.

**Serum microvesicle protein levels are associated with acute myocardial ischemia**

Vince C. de Hoog^1^, Leo Timmers^1,2^, J. Karlijn van Keulen^1^, Dominique P.V. de Kleijn^1,3^ and Arend Mosterd^4,5^

^1^Laboratory of Experimental Cardiology, UMC Utrecht, The Netherlands; ^2^Department of Cardiology, UMC Utrecht, The Netherlands; ^3^Interuniversity Cardiology Institute of the Netherlands; ^4^Department of Cardiology, Meander Medical Center Amersfoort, The Netherlands; ^5^Julius Center for Health Sciences and Primary Care, UMC Utrecht, The Netherlands

Email: V.C.dehoog@umcutrecht.nl

*Introduction*: Acute coronary syndrome (ACS) is a major health problem, for which early detection is crucial and adequate diagnostic biomarkers are needed. Microvesicles are small vesicles in the plasma containing significant amounts of protein and RNA that have been shown to be involved in ACS-related processes, like coagulation, tissue injury and apoptosis. Therefore, we hypothesized that serum microvesicle protein levels can be used to diagnose acute myocardial ischemia in suspected patients. *Methods*: Potential microvesicle protein biomarkers were identified with differential Q-proteomics in pooled serum samples of 30 ACS patients versus 30 sex- and age-matched controls. These biomarkers were evaluated retrospectively in a cohort of 541 ACS-suspected patients presenting to the emergence department. Microvesicles were isolated from frozen serum samples by ExoQuick isolation. Subsequently microvesicle protein levels were measured by Luminex-based multiplex panels. Protein levels were adjusted for total protein concentration and log transformed to allow for parametric analyses. Mean microvesicle protein levels were compared between ACS and non-ACS patients with Student's *t*-test and ROC-curves were calculated for each marker. Subgroup analyses were performed for patients with negative troponin levels at arrival. *Results*: Of 541 patients, 168 (31%) were diagnosed with ACS. The mean microvesicle protein concentration was significantly higher for Cystatin C (2.32±0.04 vs. 2.08±0.03, *p*<0.001) and CD169 (−2.48±0.04 vs. −2.58±0.03, *p*=0.043) and significantly lower for antithrombin 3 (AT3) (4.87±0.07 vs. 5.14±0.05, *p*=0.002) in patients with ACS compared with non-ACS. The area under the curve (AUC) of the ROC curve was 0.640 for Cystatin C (*p*<0.0001), 0.557 for CD169 (*p*=0.051) and 0.601 for AT3 (*p*<0.001). This relation was stronger in patients with negative troponin at arrival (n = 415, 16% ACS): AUC 0.659 for Cystatin C (*p*<0.001), 0.588 for CD169 (*p*=0.033) and 0.623 for AT3 (*p*=0.003). *Conclusion*: These data show for the first time that serum microvesicle protein content is associated with acute myocardial ischemia. These observations should be validated in larger prospective studies assessing the added value of microvesicle content to current biomarkers of ischemia.

Funded by: Dutch Heart Foundation, The Netherlands; University Medical Center, Utrecht, The Netherlands

**Effect of exosomes/microvesicles derived from CD133 + renal progenitors in renal repair and modulation by hypoxia**

Aldo Moggio, Veronica Dimuccio, Giovanni Camussi and Benedetta Bussolati

Department of Internal Medicine, University of Torino, Italy

Email: aldo.moggio@unito.it

Increasing evidences show that stem cells are involved in tissue regeneration through a paracrine release of factors, including exosomes/microvesicles (MVs). We recently isolated and characterized a population of CD133 + epithelial progenitors in the renal papilla, and we found that hypoxia promotes their stem/progenitor properties through a balance between Oct4A and microRNA-145 (1). In the present work, we isolated and characterized MVs released from the renal papilla CD133 + progenitor cells in normoxia and in hypoxic condition (1% O_2_), and we analyzed in vitro and in vivo their effect on renal repair. The CD133 + MV population, from both hypoxic and normoxic cells, showed a size of approximately 130 nm (peak of 127 nm), as evaluated using the NanoSight LM10 system, and was characterized by expression of exosome surface markers (CD24 and CD63) by cytofluorimetric analysis using latex beads. In vitro, only hypoxic but not normoxic CD133 + MVs stimulated the proliferation of starved epithelial tubular cells. Moreover, the RNase treatment reverted this proliferative effect. In vivo, in a murine model of acute kidney injury, injection of hypoxic but not normoxic CD133 + MVs improved renal function evaluated as serum creatinine, nitrogen urea and morphological tissue analysis. The miRNA content of the normoxic and hypoxic CD133 + MVs was analyzed by the Applied Biosystems TaqMan^®^ MicroRNA Assay, able to profile 365 mature miRNAs and confirmed by deep sequencing. The analysis showed the presence of several microRNAs involved in cell proliferation, differentiation and inhibition of fibrotic pathways. In conclusion, we show the ability of CD133 + renal papillary progenitor cells to induce repair in the injured kidney through the delivery of MVs. Moreover we show that the hypoxic microenvironment could influence the biological functions of the MVs.

Funded by: Regione Piemonte, PISTEM Project

**Reference**

1. Bussolati B, et al. Am J Physiol Renal Physiol. 2012 Jan;302(1):F116–28.

**Therapeutic efficacy of human MSC exosome is mediated by injury-calibrated enzyme activities**

Sai Kiang Lim^1,2^, Ruenn Chai Lai^1^, Fatih Arslan^3,4^, Soon Sim Tan^1^, Bin Zhang^1^, Yijun Yin^1^, Newman Siu Kwan Sze^5^, Andre Choo^6^, and Dominique P. de Kleijn^3,4^

^1^Institute of Medical Biology, A*STAR, Singapore; ^2^Department of Surgery, YLL School of Medicine, NUS, Singapore; ^3^Laboratory of Experimental Cardiology, University Medical Center Utrecht, Utrecht, The Netherlands; ^4^Interuniversity Cardiology Institute of the Netherlands, Utrecht, The Netherlands; ^5^School of Biological Sciences, Nanyang Technological University, Singapore; ^6^Bioprocessing Technology Institute, A*STAR, Singapore

Email: saikiang.lim@imb.a-star.edu.sg

Extensive clinical trials and animal models have demonstrated that the therapeutic efficacy of mesenchymal stem cells (MSCs) against a plethora of diseases is mediated by paracrine secretion. We have shown that this secretion-based efficacy against myocardial ischemial/reperfusion (MI/R) injury is mediated by exosomes with a cargo enriched in enzymes driving diverse biochemical processes. Since enzyme activities are catalytic and not stoichiometric, and thus dependent on their microenvironment, e.g., substrate concentration or pH, an enzyme-based therapeutic activity will be calibrated in a dose-independent manner by the severity of the disease-precipitating microenvironment. Furthermore, such activity is arrested when the disease or injury is resolved. Here, we show that MSC exosomes have the enzymes to compensate for biochemical processes known to be disrupted during MI/R injury and ameliorate the known consequences of such disruptions such as ATP deficiency, loss of ion homeostasis, calcium overload, ER stress, osmotic swelling, ROS production, complement activation and apoptosis. This was confirmed in a mouse model of MI/R injury, which also manifested reduced MI/R injury. This repair of complex MI/R injury at the molecular level exemplifies the nanotechnological capability of MSC exosomes and provides a mechanistic insight into the therapeutic effectiveness of MSC against diverse diseases.

Funded by: Biomedical Research Council, A*STAR, Singapore.

**Microvesicles (MVs) derived from mesenchymal stem cells for treatment of kidney injury**

S. Bruno^1^, C. Grange^1^, F. Collino1, M.C. Deregibus^1^, C. Tetta^2^ and G. Camussi^1^

^1^University of Torino, Italy; ^2^Fresenius Medical Care, Bad Homburg, Germany

Email: stefania.bruno@unito.it

Mesenchymal stem cells (MSCs) ameliorate experimental acute kidney injury (AKI) and induce functional improvement in chronic kidney disease (CKD), mainly by paracrine action, but the factors involved remain elusive. MSC-derived MVs expressed several adhesion molecules of MSCs including CD44 and CD29 that are instrumental in their internalization in renal cells. The RNA content included mRNAs and microRNA associated with the mesenchymal differentiative phenotype and with several cell functions (transcription, proliferation, immune regulation and multiorgan development). We found in vitro that MVs are able to stimulate proliferation and apoptosis resistance of tubular epithelial cells. We also purified MVs from dil-labeled MSCs to obtain fluorescent MVs to follow their in vivo biodistribution. The renoprotective effect of MVs has been compared with that of the MSCs in vivo, in different animal models of AKI induced by toxic agents (glycerol and cisplatin) or by ischemia and reperfusion (IR). MVs were found to accelerate the morphological and functional recovery of glycerol-induced AKI in SCID mice; this effect was comparable to that obtained with MSC administration. Labeled MVs accumulated within tubular cells of injured kidneys. In the lethal model of AKI induced by cisplatin, a single administration of MVs improved survival (50%) but did not prevent chronic tubular injury; multiple injections of MVs increase survival up to 80% and prevent completely chronic tubular injury. We also found that a single administration of MVs, immediately after IR, protected rats from AKI and prevented CKD. The mechanisms of protection were mainly ascribed to the stimulation of cell proliferation and the inhibition of apoptosis and, at least in part, dependent on their RNA cargo. In conclusion, MVs released from MSCs were found to exert a prosurvival effect on renal cells in vitro and in vivo, suggesting that MVs may contribute to kidney protection conferred by MSCs.

Funded by: Regione Piemonte Convercing Technologies NanoIGT Project

### 3. Production, Release and Function in Blood and Bone marrow

**Human CD34 + hematopoetic stem cells induce therapeutic angiogenesis via exosomes-mediated microRNA transfer**

Susmita Sahoo, Tina Thorne, Sol Misener, Aiko Ito, Douglas W. Losordo and Douglas E. Vaughan

Feinberg Cardiovascular Research Institute, Northwestern University, Chicago, IL, USA

Email: susmita-sahoo@northwestern.edu/susmita.sahoo@gmail.com

Locally transplanted human CD34 + hematopoietic stem cells have stimulated neovascularization in preclinical studies and have been associated with functional improvements in phases I and II clinical trials of patients with ischemic cardiovascular diseases. Our recent work (1) has demonstrated that exosomes are the primary proangiogenic component of CD34 + cell paracrine secretion and that exosomes isolated from peripheral blood-derived CD34 + cells (CD34 + Exo) can induce angiogenesis independently of the cells. Thus, we hypothesize that the beneficial angiogenesis associated with CD34 + cell therapy is primarily mediated by CD34 + Exo and that administration of CD34 + Exo alone could reproduce the therapeutic effects of CD34 + cell transplantation. CD34 + Exo-treatment induced angiogenesis and tissue repair in a mouse model of hind-limb ischemia (HLI); it significantly improved perfusion (ratio: 1.01±0.04 vs. 0.57±0.1, *p*<0.05), increased capillary density (1.8±0.3/HPF vs. 0.9±0.1/HPF, *p*<0.001) and prevented ischemic leg amputation (16% vs. 100%), as compared to exosomes from non-angiogenic, CD34 + cell-depleted-mononuclear cells (MNC Exo). Our microarray data and confirmatory tests revealed that compared to MNC Exo, CD34 + Exo are enriched in proangiogenic miRNAs and that direct transfer of miR-126 is necessary for CD34 + Exo-induced activation of endothelial cells and angiogenesis both in vitro and in vivo. Currently, we are studying the effect of inhibition of exosome secretion from CD34 + cells on miR-126 transfer and on the therapeutic benefits of the cells. These studies will positively impact cardiovascular regenerative medicine by discovering and characterizing the predominant, but as yet undefined exosomal-miRNA-induced mechanisms of CD34 + cell therapy, and by beginning to determine whether CD34 + Exo could be a useful, cell-free alternative to CD34 + cell therapy.

Funded by: National Institute of Health, USA (HL095874-01 NIH/NHLBI)

**Reference**

1. Bussolati B, et al. Am J Physiol Renal Physiol. 2012 Jan;302(1):F116–28.

**Microvesicle-mediated modulation of hematopoietic stem cell phenotype is influenced by stem cell cycle status**

L.R. Goldberg, J. Aliotta, D. Lee, N. Puente, E. Sears, M. Pereira, S. Faradyan, A. Amaral, E. Papa, M. Del Tatto, M.S. Dooner and P.J. Quesenberry

Rhode Island Hospital, Warren Alpert Medical School of Brown University, Department of Medicine, Division of Hematology and Oncology and Division of Pulmonary, Sleep and Critical Care Medicine, Providence, RI, USA

Email: lgoldberg@lifespan.org

Microvesicles have been shown to be potent modulators of hematopoietic stem cell phenotype. Studies have demonstrated that when lung-derived microvesicles (LDMV) are co-cultured with hematopoietic stem cells (HSC), HSC internalize the microvesicles and are subsequently induced to express pulmonary epithelial cell-specific mRNA and protein. Cell cycle phase has also been shown to be a critical component of marrow stem cell phenotype with dramatic and reversible alterations in gene expression, engraftment and differentiation potential linked to cell cycle transit. We hypothesized that, given the myriad changes that occur with cell cycle progression, HSC cell cycle phase itself would directly affect microvesicle-mediated communication. In the studies presented here, we first show that, contrary to current dogma holding HSCs to be predominantly quiescent, unseparated whole bone marrow (WBM) contains a population of actively cycling HSCs. Second, we show that the cell cycle status of marrow stem cells significantly influences cell susceptibility to microvesicle-mediated phenotype modulation. For cell cycle studies, WBM was either separated into cell cycle-specific fractions using Hoechst 33342/Pyronin Y or exposed to tritiated thymidine suicide for selective killing of actively cycling cells and then competitively engrafted into lethally irradiated mice. Percent donor chimerism was measured using flow cytometry to evaluate donor engraftment. These studies revealed that over 50% of the long-term engrafting potential in WBM was in S/G2/M, strongly indicating that the HSC population is actively cycling. To determine the effects of cell cycle transit on microvesicle-mediated phenotype changes, lineage-depleted, Sca-1 + cells (Lin-Sca-1 + ) were isolated from whole bone marrow and cultured with IL-3, IL-6, IL-11 and stem cell factor (cytokine-cultured cells). Cells were then subcultured with LDMV at the following timepoints corresponding to synchronized points in cell cycle: G0/G1 (time 0), late G1/early S (24 hours) and late S/early G2/M (48 hours). Phenotype changes in the target Lin-Sca-1 + cells, as measured by pulmonary epithelial cell-specific mRNA expression, were greatest when the cells were in the late G1/early S phase at the time of LDMV exposure. In contrast, if LDMV were harvested from irradiated mice, peak lung-specific mRNA expression was seen when the co-cultured Lin-Sca-1 + cells were in G0/G1. Mechanistically, this difference may be due in part to variations in adhesion proteins, including ICAM-1, TAPA-1 and CD40 ligand, whose expression levels were found to differ between LDMV from irradiated versus non-irradiated lung. Separation of Lin-Sca-1 + cells into G0/G1 and S/G2/M cell cycle- specific fractions using Hoechst 33342 in a cytokine-independent manner yielded similar results. In summary, these data indicate that hematopoietic stem cells are an actively cycling population and, as such, will be differentially susceptible to the microvesicle-mediated modulation of their phenotype depending on their cell cycle state.

Funded by: This work was supported by the following NIH grants: 5K08 HL086868-04, 8P20GM103468-04.

**Chronic lymphocytic leukemia-derived exosomes and microvesicles shuttle proteins and miRNA to influence bone marrow mesenchymal stem cells**

Etienne Moussay^1*^, Jérôme Paggetti^1*^, Wibke Jochum^1^ and Guy Berchem^1,2^

^1^Laboratory of Experimental Hemato-Oncology, Public Research Center for Health, CRP-Santé; ^2^Centre Hospitalier de Luxembourg, Luxembourg^*^Both authors contributed equally to this work

Email: etienne.moussay@crp-sante.lu/jerome.paggetti@crp-sante.lu

Exosomes and microvesicles are known to deliver signals to target cells via proteins or RNA molecules and, therefore, constitute a new component of intercellular communication. Chronic lymphocytic leukemia (CLL) B cells are supported in vivo and in vitro by bone marrow mesenchymal stem cells (MSC), which promote CLL cells survival by direct contact and secretion of soluble factors and cytokines. We hypothesize that CLL B cells stimulate MSC to release these prosurvival factors by releasing protein- and RNA-containing vesicles. We therefore isolated, purified and characterized exosomes and microvesicles derived from CLL cell lines, primary cells culture supernatants and plasma from CLL patients. Protein and microRNAs (miRNA) contents were evaluated by LC-MS and Affymetrix GeneChip miRNA 2.0 Array, respectively. Human MSC were isolated from femoral heads of patients undergoing hip surgery. By using 0.4-µm culture inserts, we observed that primary CLL B cells transfer vesicles to MSC. In addition, purified vesicles were found to transfer surface proteins, activated kinases and miRNA to MSC, which may act as potential regulators of MSC biology. Exosomes and microvesicles were also found to influence MSC cell cycle and proliferation probably through the phosphorylation of Akt kinase. In conclusion, when altered by CLL-derived exosomes and microvesicles, MSC could favor leukemia cells survival.

Funded by: Fonds National de la Recherche (FNR) Luxembourg Télévie

**Megakaryopoiesis, platelet senescence and release of platelet-derived extracellular vesicles**

Gerd Schmitz, Annika Pienimäki-Römer, Katharina Rübsaamen and Evelyn Orsó

Institut for Clinical Chemistry and Laboratory Medicine University Hospital Regensburg, Regensburg

Email: gerd.schmitz@klinik.uni-regensburg.de

Megakaryocytes release platelets into the blood by progenitor cell fragmentation. We established in vitro models for megakaryocytic differentiation of human cord blood progenitors into proplatelets, shedding of platelets and release of extracellular vesicles, and for analysis of in vitro platelet senescence in human hemapheretic platelet preparations, and analyzed platelets, platelet extracellular vesicles (PL-EVs) and surrounding autologous plasma over 5 days with transcriptomics, lipidomics, proteomics and miRNA patterning. The HDL-regulator ABCA1 is essential for proplatelet shedding and controls cargo packing of platelet granules. Treatment of megakaryocytes with HDL3/apoA-I induces proplatelet shedding and reduces PL-EVs. We purified PL-EVs by differential centrifugation and filtration into five subclasses. Stored platelets double EV-release, which is antagonized by apoA-I/HDL3 via SR-B1. PL-EVs are enriched in free cholesterol, sphingomyelin, dihydrosphingomyelin, glycerophospholipid-, plasmalogen- and lysophosphatidyl choline-lipid species. The mitochondrial marker cardiolipin is enriched in PL-EV subfraction-5, indicative for autophagic vesicles of mitochondrial origin. PL-EV subfractions are differentially enriched in nucleic acid binding proteins, caveolin-1, apolipoproteins A-I, J and E, α-synuclein, amyloid-b precursor protein (APP) and microRNA. PL-EVs have a lipid composition similar to lipid rafts and carry lipid ligands for G-protein coupled signaling, precursors for eicosanoid biosynthesis and apolipoproteins, involving PL-EVs to orchestrate cellular processes in vascular and metabolic diseases.

Funded by: Lipidomic Net

**Stromal cell regulation and drug-mediated miRNA recruitment by acute myeloid leukemia exosomes**

Noah Hornick, Jianya Huan and Peter Kurre

Pape Pediatric Research Institute, Department of Pediatrics, Oregon Health & Science University, Portland, OR, USA

Email: hornickn@ohsu.edu

Treatment success and drug resistance in acute myeloid leukemia (AML) is compromised in part by signaling between leukemic cells and the surrounding stroma. We previously reported the release of exosomes from AML cells. After entry into co-cultured stromal cells, AML exosome-containing RNA was translated and functional. Exosomes were also enriched for several prognostically relevant mRNA, including NPM-1, MMP-9, CXCR4, IGF-1R and the FLT3-ITD mutant. The latter results in constitutive kinase signaling as a source of drug resistance in AML. To demonstrate how exosomes facilitate communication between leukemic blasts and stromal cells, we used a FLT3-ITD + cell line (MOLM14). Exposure of OP9 marrow stroma to MOLM14 exosomes resulted in proliferative responses and altered protein release by OP9 cells as well as modulating migration of BaF3 cells, a validated model of FLT3 signaling. RT-PCR array analysis of RNA content revealed instances of enrichment and exclusion of transcription factor mRNA, including c-Myc, CEPB-A, GATA1, MyoD and ID-1. Global and small RNA-specific bioanalyzer profiles also revealed the relative enrichment of small RNA, containing mature as well as precursor microRNA (miRNA) in AML exosomes. A survey of 384 miRNA by array and confirmatory real-time PCR shows the enrichment of several miRNA (miR-99b, -155, -191 and let-7a) with established roles in both physiologic hematopoiesis and in AML. The exosome incorporation of miR-155 was found to be substantially altered by exposure to a FLT3-specific tyrosine kinase inhibitor. This is the first report of drug-mediated regulation of miRNA incorporation in exosomes and significantly broadens potential therapeutic drug targets. Taken together, our findings support a model whereby AML exosome traffic participates in niche signaling between leukemic blasts and stromal cells.

Funded by: National Heart, Lung, and Blood Institute (National Institutes of Health); Hope on Wheels (Hyundai Foundation)

**Microparticle-dependent procoagulant activity and thrombin generation is increased in patients with cirrhosis induced coagulopathy**

V. Jairath^1,2^, P. Harrison^3^, S.J. Stanworth^1^, J.D. Collier^2^, M.F. Murphy^1^ and E.J. Barnes^2,4^

^1^NHS Blood & Transplant, John Radcliffe Hospital, Oxford, United Kingdom; ^2^Translational Gastroenterology Unit, John Radcliffe hospital, Oxford, United Kingdom; ^3^Oxford Haemophilia and Thrombosis centre, Oxford, United Kingdom; ^4^NIHR Biomedical Research Unit, University of Oxford, United Kingdom

Email: vipul.jairath@nhsbt.nhs.uk

*Background Aims*: Recent data suggest stable cirrhotics may have a hypercoagulable phenotype. Microparticles (MPs) are submicron plasma particles formed by the exocytic budding of cell membranes and play an important role in hemostasis due to phosphatidylserine (PS) surface expression, which provides a phospholipid surface for assembly of coagulation enzymes and/or the expression of tissue factor (TF), the primary initiator of coagulation. To determine whether MPs may contribute to this hypercoagulable phenotype, we assessed microparticle-associated functional procoagulant and phenotypic characteristics in cirrhotics. *Methods*: Sevety-two consecutive cirrhotics and 30 healthy volunteers were recruited. Platelet-free plasma (PFP) was prepared by two centrifugations and MP-free plasma (MP-FP) by filtration of PFP through a 200-nm microparticle filtration unit. MP-associated procoagulant activity (PCA) was measured using the STA Procoag PPL (phospholipid) assay (Stago Diagnostics) and MP-associated thrombin generation (TG) measured using the calibrated automated thrombogram (CAT). For the CAT assay, TG was initiated by adding CaCl2 and 1 pM tissue factor, but no phospholipid (PRP reagent); therefore, TG was dependent upon phospholipid present in the sample. Flow cytometry (LSRII) was used to determine MP size, number and cellular origin using marker-specific antibodies. *Results*: PFP from cirrhotics generated significantly more thrombin than healthy volunteers reflected in the ETP (1374.3 vs. 1142.6 nM/min, *p*=0.04), the peak (101.0 vs. 66.5 nM, *p*=0.001) and a shorter time to peak (13.0 vs. 14.2 min, *p*=0.03). Similarly, MP-associated PCA was significantly increased in cirrhotics (65.9±13.2 s), compared to healthy volunteers (74.6±13.9 s, *p*=0.005). Following filtration of MPs >200 nM in size, there was a large reduction in ETP and peak in both cirrhotics and healthy volunteers, with prolongation of both the time to peak and PPL time. There was significant inverse correlation between the PPL assay and parameters of the TG test [ETP (*r*= − 0.57, *p*<0.001, peak (*r*= − 0.43, *p*<0.001]. Cirrhotic patients had high levels of annexin V-binding PS positive MPs compared to controls (1412 vs. 279 per µl, *p*<0.05). *Conclusions*: Microparticle-dependent procoagulant activity and thrombin generating capacity is increased in plasma from cirrhotics. High levels of annexin-V positive procoagulant MPs are a likely key and previously undescribed mechanism contributing to the hypercoaguable phenotype observed in cirrhotics.

Funded by: NHS Blood and Transplant

## Coffee and Poster Session 1          15.30-16.00

## Symposium Session 3          16.00-17.00

### 1. Inflammation and Infection

**Exosomes derived from HIV-1-pulsed cells induce apoptosis in Th17**

Caroline Gilbert, Sébastien Simard, Gabrielle Beaulieu-Carbonneau and Caroline Subra

Centre de Recherche du CHUQ, Faculté de Médecine, Université Laval, Québec, QC, Canada

Email: caroline.gilbert@crchul.ulaval.ca

*Background*: Mucosal CD4TL (CD4+ T lymphocytes) loss is a hallmark of human immunodeficiency virus 1 (HIV 1) infection. The recently described Th17 helper cells are the major cell type for HIV 1 replication and are rapidly depleted in the pathogenic models of the disease. These cells produce IL-17 and play a crucial role in mucosal antimicrobial immunity and tissue homeostasis. While several mechanisms promoting T cells apoptosis have been proposed, they fail to fully explain the observed T cell loss during the acute phase of the disease. Dendritic cells (DCs) are thought to play a pivotal role in the spread of viruses throughout the organism, both establishing and maintaining HIV infection. DCs capture virions and endocytose them into late endocytic compartments and subsequently deliver viral particles to target cells. Internalized viral particles are found in cellular compartments enriched with nanovesicles known as exosomes. These cellular vesicles and virions are released together by DCs. Whether or not exosomes are benign elements in HIV 1 pathogenesis is still unknown. *Methods*: By using immunocapture of exosomes and cytofluorometry, we examined the role of exosomes derived from HIV 1-pulsed DCs in the viability of several CD4TL subsets including the Th17. *Results*: DCs or CD4TL or co-cultures of both cell types exposed to HIV 1 release greater amounts of exosomes in extracellular media than control cells. Using a method that efficiently separates HIV 1 from exosomes, we showed that CD4TL incubated with exosomes purified from conditioned media of HIV 1-pulsed DCs display a proapoptotic profile. In addition, among the CD4TL subsets, Th17 are most susceptible to apoptosis induced by these non-infectious nanovesicles and in contrast to apoptosis induced by H_2_O_2_. Altogether, our results suggest that exosomes derived from HIV 1-pulsed cells can mediate apoptosis. *Conclusions*: Quite paradoxically, exosome release appears to be an important immune modulatory mechanism that may contribute to T-cell depletion observed upon HIV infection.

Funded by: An operating grant to C.G. from the Canadian Institutes of Health Research (CIHR) (MOP-90179). C.S. is the recipient of a fellowship award from the Canadian Institutes of Health Research HIV/AIDS Research Program. C.G. is the recipient of Junior 1 Scholarship Award from the Fonds de la Recherche en Santé du Québec and a New investigator Award from the CIHR.

**Antigen-specific exosomes: a new way to target delivery of selected siRNAs**

Philip W. Askenase^1^ and Krzysztof Bryniarski^2^

^1^Yale University School of Medicine, New Haven, CT, USA; ^2^Jagiellonian University School of Medicine, Kracow, Poland

We have discovered antigen-specific nanovesicles, which we judge to be exosomes that are produced by CD8+ suppressor T cells mediating immune tolerance. Antigen-specific function was demonstrated by dual reciprocal antigen-specific suppression: i.e., exosomes of two different antigen specificities were each tested for inhibition of effector cells of both specificities in recipients challenged with each specificity. Exosomes only inhibited effector cells of homologous antigen specificity. To test antigen binding, we performed exosome antigen affinity chromatography. Exosomes of a given antigen specificity bound homologous antigen-linked columns and not heterologous antigen-linked columns. This showed that a 10% exosome subpopulation that actually bound antigen had all the suppressive function. The specificity resembled that of antibodies. Indeed, flow cytometry revealed immunoglobulin kappa light chains on the surface of the exosomes. This led to experiments in JH − /− pan immunoglobulin-deficient mice that when tolerized produced non-suppressive exosomes. Importantly, incubation of these inactive exosomes with chosen antigen-specific monoclonal immunoglobulin light chains reconstituted antigen-specific suppression. Our data suggest that delivery of inhibitory miRNA that mediated the suppression, via antigen-specific targeting, determined by coating exosomes with antibody of choice, opens a new pathway for their therapeutic use. This could involve antigen-specific delivery to targeted markers on cancer cells by chosen antibodies, for delivering selected siRNA to inhibit oncogenesis.

Funded by: NIHAI-076366 and AI-07074

**Monocyte-derived microvesicles modulate proinflammatory response and TRAIL-mediated apoptosis of rheumatoid arthritis synovial fibroblasts**

M. Frank Bertoncelj, R.E. Gay, B.A. Michel, O. Distler, S. Gay and A. Jungel

Center for Experimental Rheumatology, University Hospital Zurich, Switzerland

Email: mojca.frank@usz.ch

We have recently found that monocyte-derived microvesicles (MV) significantly decrease death receptor-mediated apoptosis of rheumatoid arthritis (RA) synovial fibroblasts (SF) and may contribute to synovial hyperplasia and joint destruction in RA.

*Objective*: To assess the role of NFκB in modulating the effects of MV on inflammatory response and TNFα-related apoptosis inducing ligand (TRAIL)-mediated apoptosis of RASF. *Methods*: RASF were treated with MV from Poly(I:C)-stimulated or untreated U937 cells in the presence or absence of TRAIL and sc-514, an inhibitor of NFκB signaling. Real-time PCR and ELISA were used to determine mRNA and protein levels of IL-6 and IL-8, respectively. Apoptosis of RASF was measured by flow cytometry using annexin V/propidium iodide staining. *Results*: Poly(I:C)-induced MV, but not MV from the same number of unstimulated cells, increased the production of IL-6 and IL-8 by RASF on mRNA and protein levels in a time-dependent manner and significantly decreased TRAIL-induced apoptosis of RASF. Treatment of RASF with MV-free supernatants, obtained in the last washing of MV, had no effect on cytokine production or apoptosis of RASF. Inhibition of NFκB signaling with sc-514 significantly increased TRAIL-mediated apoptosis of RASF. Poly(I:C)-induced MV significantly decreased TRAIL-mediated apoptosis of RASF in the presence of sc-514. *Conclusion*: Monocyte-derived MV significantly increased the production of NFκB-dependent cytokines in RASF. However, their role in NFκB-dependent modulation of TRAIL-mediated apoptosis of RASF has to be further clarified.

Funded by: Articulum, IMI-BT Cure, IAR Epalinges and FP7 Masterswitch.

**Structural and genetic analysis of exosomes using cryoelectron tomography and RNA-sequencing reveals distinct features correlating with prion infectivity**

Andrew F. Hill^1^, Bradley M. Coleman^1^, Eric Hanssen^2^, Victoria A. Lawson^3^ and Shayne Bellingham^1^

^1^Department of Biochemistry and Molecular Biology, Bio21 Molecular Science and Biotechnology Institute, University of Melbourne, Parkville, Victoria, Australia; ^2^Electron Microscopy Unit, Bio21 Molecular Science and Biotechnology Institute, University of Melbourne, Parkville, Victoria, Australia; ^3^Department of Pathology, Bio21 Molecular Science and Biotechnology Institute, University of Melbourne, Parkville, Victoria, Australia

Email: a.hill@unimelb.edu.au

Exosomes are small membrane bound vesicles released from cells and found in vivo in most biological fluids. Traditionally characterized by their protein content and appearance using transmission electron microscopy, the precise structure of an exosome has yet to be firmly established, as a number of similar vesicles released by cells have similar properties. We have used cryoelectron tomography to define the molecular characteristics of the structure of highly purified populations of exosomes. This technique provides ultrastructural detail of these vesicles in their native, hydrated form and reveals that exosomes are naturally spherical in shape and have a diverse population that varies in size and internal composition such as differences in the number of membrane structures. As exosomes are known to carry protein and genetic (such as miRNA) cargo between cells, we investigated their involvement in transferring misfolded forms of proteins associated with neurodegenerative diseases. Prion diseases are a group of transmissible neurodegenerative disorders, which are associated with an abnormal isoform (PrPSc) of the host encoded cellular prion protein (PrPC). We demonstrate using in vitro bioassay and the protein misfolding cyclic amplification assay (PMCA) that exosomes contain prion infectivity and autocatalytic prion conversion activity. Furthermore, cryoelectron tomography of exosomes from prion-infected cells demonstrated a shift in the proportion of exosomes with distinct membrane structures. Analysis of the genetic component of exosomes also reveals differences in profiles of miRNA between the control and prion-infected populations. Differences in the RNA content were also identified using next-generation sequencing and qPCR analysis of exosomal RNA species. These data provide further insight into the role that the exosomal protein and genetic cargo plays on influencing the structure of the vesicles as well as highlighting the diversity of exosomes and their relationship to biological processes.

Funded by: Grants by the Australian Research Council and the National Health and Medical Research Council of Australia.

### 2. Isolation and Characterization

**Lessons of the electron microscopic analysis of microvesicle preparations produced by routinely used protocols**

Ágnes Kittel^1^, Tamás Géza Szabó^2^ and Edit I. Buzás^2^

^1^Institute of Experimental Medicine, Hungarian Academy of Sciences, Budapest, Hungary; ^2^Department of Genetics, Cell- and Immunobiology, Semmelweis University, Budapest, Hungary

Email: kittel.agnes@koki.mta.hu

Although microvesicles (MVs) and their significance in the mechanisms of both intercellular communication and in wide spectrum of biological functions have been documented, the diverse protocols applied for their isolation and detection make the comparison of the results difficult, and in some cases even impossible. Our goal was to demonstrate the effect of different isolation techniques on the composition of the resulting MV-containing pellets. Different, routinely used centrifugation forces (such as 15,000g, 20,500g, 100,000g and 200,000g) and centrifugation times (60 and 120 minutes) were compared in the case of MVs derived from the BV2 microglial cell line. The resulting pellets were analyzed by transmission electron microscopy. The different sedimentation protocols resulted in differentially damaged MV populations. The use of 200,000g during MV isolation was found to hold the risk of destroying the ultrastructure of MVs. Importantly, different parts of the same MV pellet showed highly different distribution of size, shape and electron density of MVs. Electron microscopy suggests that differential centrifugation isolation of MVs (without filtration) does not yield in homogenous populations of vesicles and draws our attention to the fact that, by selecting a given electron microscopic field of an MV pellet, we may introduce significant bias to the assessment of MV preparations. Taken together, our results suggest that there is a strong need for standardization of the electron microscopic investigation of MVs.

Funded by: OTKA K 73247, OTKA 77537

**Purification and characterization of two discrete exosome populations from the human colon cancer LIM1863 cell line**

Richard J. Simpson, Bow J. Tauro, David W. Greening, Hong Ji, Suresh Mathivanan and Rommel A. Mathias

La Trobe Institute for Molecular Science (LIMS), La Trobe University, Bundoora, Victoria, Australia

Email: richard.simpson@latrobe.edu.au

Critical to understanding the structure/function of exosomes is the need to work with homogenous moieties. Here, we describe a comparative proteomic analysis of LIM1863 cell-derived exosomes isolated by ultracentrifugation, OptiPrep™ density-based centrifugation, or EpCAM immunoaffinity capture (IAC). Label-free normalized spectral counting of GeLC-MS/MS data revealed that IAC was the superior method for enriching exosomes from cell culture medium, as assessed by enrichment of (i) typical exosome markers such as TSG101 and Alix, (ii) proteins involved with exosome biogenesis, (iii) trafficking proteins and (iv) proteins implicated in exocytic vesicle release. In another study, we compared the proteomes of A33 IAC-derived exosomes from LIM1215 and LIM1863 colon cancer cells. Interestingly, we observed two populations of exosomes in LIM1863 cells, which we were able to purify by employing a sequential IAC strategy using A33- and EpCAM mAbs. These two exosome populations were subjected to GeLC-MS/MS proteomic profiling and qualitative protein differences were determined by spectral counting. Both exosome preparations contain typical exosome marker proteins, as revealed by MS and Western blotting; they were morphologically indistinguishable by electron microscopy. Intriguingly, EpCAM-IAC exosomes were significantly enriched with apical trafficking proteins, while basolateral sorting proteins were associated with A33-IAC exosomes. These data suggest alternative exosome biogenesis and sorting pathways may occur simultaneously in LIM1863 cells. Studies to determine their biological consequences are currently underway.

Funded by: National Health & Medical Research Council of Australia (program grant #487922) and a University of Melbourne Research Scholarship (B.J.T.)

**Isolation and characterization of distinct classes of prostasomes**

Willem Stoorvogel, Marian Aalberts, Edita Sostaric, Richard Wubbolts, Marca W.M. Wauben, Esther N. Nolte-‘t Hoen and Tom A.E. Stout

Department of Biochemistry and Cell Biology, and Department of Equine Sciences, Faculty of Veterinary Medicine, Utrecht University, The Netherlands

Email: w.stoorvogel@uu.nl

During or directly after ejaculation, sperm cells are mixed with secretions from the prostate and other accessory sex glands. In addition to soluble constituents, seminal fluid from many mammalian species has been found to contain various types of extracellular vesicles, including prostasomes. Prostasomes are generated within and secreted by prostate epithelial cells in a process similar to the production of exosomes by other cell types. The proposed functions of prostasomes include prevention of immune-mediated destruction of spermatozoa within the female reproductive tract and modulation of the fertilizing capacity of sperm cells. How prostasomes could mediate such diverse functions, however, remains unclear. We identified and isolated two distinct classes of prostasomes on the basis of unique biochemical characteristics. This provided a means to study and assign specific functions to classes of prostasomes and to study their prognostic value for (sub)fertility and/or diseases such as prostate cancer.

Funded by: Utrecht University

**Preanalytical and analytical factors influencing the assessment of extracellular vesicles**

Edit I. Buzás, Bence György, Tamás G. Szabó, Krisztina Pálóczi, Ágnes Kittel, Mária Pásztói, Petra Misják, Katalin Szabó-Taylor, Balázs Szántó, Lilla Turiák, Károly Vékey, György Nagy and András Falus

Semmelweis University, Department of Genetics, Cell- and Immunobiology, Budapest, Hungary

Email: edit.buzas@gmail.com

Preanalytical variables have a major impact on the results of microvesicle assessment in biological samples. In our work, we compared different preanalytical variables (e.g., centrifugation forces and times as well as conditions of blood sample collection) in order to optimize and standardize subsequent microvesicle assessment. During differential centrifugation prior to pelleting of a given extracellular vesicle population, some of the respective vesicles may be selectively depleted. Detection of microvesicles also has numerous analytical pitfalls. We have shown that microvesicles and protein aggregates share biophysical parameters. As a consequence, flow cytometric assessment of microvesicles is confounded by protein aggregates (immune complexes, antibody self aggregates and biotin-avidin complexes). We developed a method to discriminate between microvesicles and vesicle mimicking signals. The lack of microvesicle markers and the absence of consensus protocols of microvesicle isolation substantially interfere with interpretation of research data. Based on proteomics data, we made an attempt to identify putative microvesicle markers as well as to distinguish true microvesicle-associated proteins from contaminating protein aggregates or complexes that co-sediment and co-purify with various extracellular vesicle populations. Overcoming these technical difficulties of isolation and assessment of extracellular vesicles may facilitate development of future extracellular vesicle-based clinical laboratory tests.

Funded by: OTKA K 73247, NK 84043 and Baross Gábor (REG-KM-09-1-2009-0010)

### 3. Nomenclature of Extracellular Vesicles GENERAL DISCUSSION Chairs and discussion coordinators: Graca Raposo & Stephen Gould

## Scientific Program 2012 ISEV meeting Wednesday 18th April

### Poster Session 1          17.00-19.00


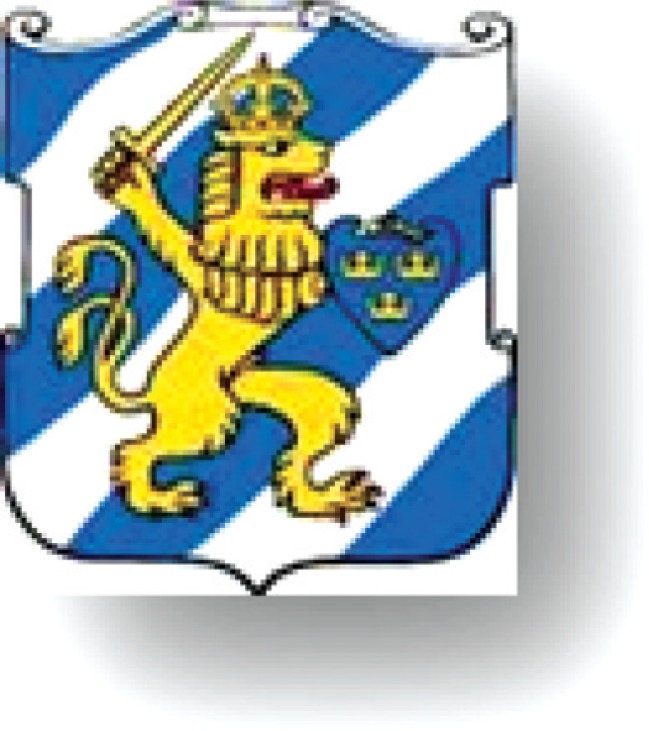
 **and Welcome Reception**

The City of Gothenburg (www.goteborg.se) is kindly inviting all ISEV delegates to the welcome reception at the congress venue. Enjoy! Interact!

### All Posters in Session 1

#### 1. Production, Secretion and function

**1. Characterization of syndecan-syntenin-1 endosomal trafficking routes**

Rania Ghossoub^1^, Frédérique Lembo^1^, Rudra Kashyap^1^ and Pascale Zimmermann^1,2^

^1^Centre de Recherche en Cancérologie de Marseille (CRCM), Aix-Marseille Univ, Inserm-U1068, Institut Paoli-Calmettes, CNRS-UMR7258, Marseille, France; ^2^Department of Human Genetics, K. U. Leuven, Leuven, Belgium

Email: rania.ghossoub@inserm.fr

Syndecans constitute a family of heparan sulfate proteoglycans and are implicated in the transport of different cargos such as growth factors, chemokines and morphogens (1). Syndecans contain a conserved cytoplasmic domain that interacts with the PDZ protein syntenin-1. Syntenin-1 supports the plasma membrane recycling (2) and the exosomal sorting of syndecans and their heparan sulfate cargo (3). Yet, how these two pathways are regulated and possibly interconnected is unknown. Addressing this question is providing some insight on the molecular mechanisms controlling cis versus trans cellular signaling.

Funded by: Fondation Santé Sport Développement Durable Institut Paoli-Calmettes Inserm-U1068

**References**

1. Lambaerts K, Wilcox-Adelman SA, Zimmermann P. The signaling mechanisms of syndecan heparan sulfate proteoglycans. Curr Opin Cell Biol. 2009;21(5):662–9.

2. Zimmermann P, Zhang Z, Degeest G, et al. Syndecan recycling [corrected] is controlled by syntenin-PIP2 interaction and Arf6. Dev Cell. 2005;9(3):377–88.

3. Baietti M-F, et al. Syndecan-syntenin-Alix regulate the biogenesis of exosomes. Nat Cell Biol. In press.

**2. Poster 2 is withdrawn**

**3. Selecting markers to analyze plasma vesicles: lack of correlation between antigens expressed on vesicles and their parent cells**

Rebecca Dragovic, Jennifer Southcombe, Dionne Tannetta, Christopher Redman and Ian Sargent

Department of Obstetrics & Gynaecology , John Radcliffe Hospital, Headington Oxford

Email: rebecca.dragovic@obs-gyn.ox.ac.uk

To identify the cellular origin of plasma microvesicles and exosomes, specific markers are required. In vitro-derived vesicles provide the ideal platform to determine whether surface antigens specific for a particular cell type are also present on vesicles derived from them. We have used flow cytometry and nanoparticle tracking analysis (NTA) in parallel to rapidly size, quantitate and phenotype in vitro-derived vesicles from platelets, red blood cells (RBCs), endothelial cells, lymphocytes, monocytes and granulocytes. Using a side-scatter threshold, we determined that our standard BD LSRII flow cytometer could analyze vesicles ≥ 290 nm but nothing smaller, whereas NTA could measure cellular vesicles down to approximately 50 nm in size. NTA of platelet, RBC and endothelial-derived vesicles revealed that their size distribution differed, ranging 50–900 nm, 50–400 nm and 50–650 nm, respectively. The modal size of vesicles from each preparation was ∼200 nm. Vesicle counts as determined by NTA vs. flow cytometry were elevated by 75-fold for platelet vesicles, 2855-fold for RBC vesicles and >10,000 fold for endothelial vesicles. Our flow cytometry data showed that: 1) not all markers are cell-specific; e.g., the typically used platelet marker CD61 was not only expressed on platelets (99%) but also on endothelial cells (99%), monocytes (>90%) and a subset of lymphocytes (∼40%) and granulocytes (∼15%); (2) antigens expressed on the parent cell are not necessarily expressed on the vesicles derived from them; e.g., 99% of endothelial cells express CD144 and CD146 whereas only ∼20% and < 5% of endothelial-derived vesicles express these antigens, respectively. Similarly, CD45 expression on monocytes (>99%), lymphocytes (>99%) and granulocytes (100%) was reduced to ∼15%, ∼20% and < 5%, respectively, on the vesicles derived from them; (3) antigen expression was dependent on the stimulation method used to produce the vesicles; e.g., CD3 expression on lymphocyte vesicles derived after stimulation with staurosporine (∼50%) was significantly different to those stimulated with PMA + ionomycin (∼10%). These results provide evidence that vesicles do not necessarily have the same antigenic repertoire as their parent cells and bring into question the use of several standard cellular markers for quantifying plasma vesicles.

Funded by: Wellcome Trust Programme Grant and by the Oxford Partnership Comprehensive Biomedical Research Centre with funding from the Department of Health's NIHR Biomedical Research Centres funding scheme.

**4. Toward tailored exosomes: the exosomal tetraspanin web contributes to target cell selection**

Shijing Yue, Sanyukta Rana, Daniela Stadel and Margot Zöller

Department of Tumor Cell Biology, University Hospital of Surgery, Heidelberg, Germany

Email: shijingyue@gmail.com

Exosomes are discussed as potent therapeutics as they efficiently transfer proteins, mRNA and miRNA in selective targets. However, therapeutic exosome application requires knowledge on target structures to avoid undue delivery. Our previous work suggesting exosomal tetraspanin-integrin complexes to be involved in target cell binding, we aimed to control this hypothesis and to define target cell ligands. Exosomes are rich in tetraspanins that associate besides other molecules with integrins. Co-immunoprecipitation of exosome lysates from rat tumor lines that differ not only with respect to Tspan8 and beta4 revealed promiscuity of tetraspanin-integrin associations but also few preferential interactions like that of Tspan8 with alpha4 and beta4 integrin chains. These minor differences in exosomal tetraspanin-complexes strongly influence target cell selection in vitro and in vivo, efficient exosome-uptake being seen in hematopoietic cells and solid organs. Exosomes expressing the Tspan8-alpha4 complex are most readily taken up by endothelial and pancreas cells, CD54 serving as a major ligand. Selectivity of uptake was confirmed with exosomes from an alpha4 cDNA transfected Tspan8+ lymph node stroma line. Distinct from exosomes from the parental line, the latter preferentially targeted endothelial cells and in vivo the pancreas. Notably, too, pulldown experiments provided strong evidence that exosome-uptake occurs in internalization prone membrane domains. This is the first report on the exosomal tetraspanin web contributing to target cell selection such that predictions can be made on potential targets, which will facilitate tailoring exosomes for drug delivery.

Funded by: German Research Foundation

**5. The Pak kinases, Paxillin and lipid rafts are required to upload microvesicles with activated ADAM17/10 proteases**

Jung-Hyun Lee, Florian S. Dreyer, Tanja Bräu and Andreas S. Baur

Department of Dermatology, University Hospital of Erlangen, Germany

Email: Jung-Hyun.Lee@uk-erlangen.de

Recent studies suggest that membrane vesicles play a pivotal role in several physiological and pathological processes, most notably in tumorigenesis. Tumor-derived microvesicles (MV) are enriched in a selective repertoire of proteins and nucleic acids from parental cells believed to play a role in cancer development and modulation of the microenvironment. ADAM-metalloproteinases, particularly ADAM17 and 10, are repeatedly found in microvesicles derived from tumor cells; however, neither their function nor mechanism of uploading from parental cells is understood. Here, we show that the Pak kinases (Pak1 and 2) as well as Paxillin are instrumental in shuttling ADAM17 and 10 into microvesicles. PAK2, which is known to promotes malignant tumor progression, increased ADAM17/10-Paxillin association and secretion through microvesicles by site-specific phosphorylation. Conversely, Pak1 blocked both functions again through a specific phosphorylation step. Mechanistically, Pak2-mediated phosphorylation of Paxillin was required for the transfer of activated ADAMs into lipid rafts and correlated with their appearance in microvesicles. Our results illustrate how cargo is uploaded into MV and imply that lipid rafts are a crucial sorting platform.

Funded by: Deutsche Forschungsgemeinschaft (DFG)

**6. Evi/Wls-mediated release of active Wnt proteins**

Julia Christina Gross, Varun Chaudary and Michael Boutros

German Cancer Research Center (DKFZ), Division Signaling and Functional Genomics, Heidelberg, Germany

Email: julia.gross@dkfz-heidelberg.de

During development, cells need to communicate to transform an undifferentiated sheet of cells into a functional, heterogeneous type of tissue. This is achieved by spatially restricted secretion of morphogens, such as proteins of the Wnt family. These secreted glycoproteins are required for a variety of developmental processes, which are highly conserved from fly to human. How hydrophobic Wnt proteins spread in the extracellular space has been a long-standing question. Different hypotheses have been proposed to explain how Wnt could travel in the extracellular space, such as coating of lipoprotein particles and/or as part of secreted vesicles. We have previously identified the transmembrane protein Eveness interrupted (Evi/Wls) as the cargo receptor of Wnt proteins. Evi shuttles Wnt proteins from the Golgi to the plasma membrane and is then recycled by the retromer complex. Newer data suggest that Evi/Wnt complexes are not just dissociating at the plasma membrane and that Evi, as a multipass membrane protein can transfer from one cell to another. Our current research focuses on understanding the trafficking of Evi/Wnts complexes into endosomal compartments leading to the secretion of Wnts on extracellular vesicles. To identify additional factors required for this process different screening approaches were used, i.e., focused sets of RNAi libraries against membrane proteins, secreted proteins and proteins involved in exocytosis/endocytosis were selected for testing in human cell culture and in the model organism *Drosophila melanogaster*. Here, we present our data on the novel functional role of Evi in Wnt secretion and our RNAi screening approaches along with candidates that are under investigation.

Funded by: DFG

**7. Human adipose tissue-derived mesenchymal stem cells secrete neprilysin-bound exosome-like vesicles**

Takeshi Katsuda^1,2^, Reiko Tsuchiya^3^, Nobuyoshi Kosaka^1^, Yusuke Yoshioka^1,4^, Kentaro Takagaki^3^, Katsuyuki Oki^3^, Fumitaka Takeshita^1^, Yasuyuki Sakai^2^ and Takahiro Ochiya^1^

^1^Division of Molecular and Cellular Medicine, National Cancer Center Research Institute, Tsukiji, Chuo-ku, Tokyo, Japan; ^2^Institute of Industrial Science (IIS), The University of Tokyo, Komaba, Meguro-ku, Tokyo, Japan; ^3^Research and Development Department, SEEMS, Inc., Aomi, Koto-ku, Tokyo, Japan; ^4^Center for Advanced Biomedical Sciences, Wakamatsu, Shinjuku, Tokyo, Japan

Email: katsuda@ncc.go.jp

One of the neuropathological hallmarks of Alzheimer's disease (AD) is accumulation of amyloid β-peptide (Aβ) in the brain because of an imbalance between Aβ production and clearance. Neprilysin (NEP), a rate-limiting Aβ-degrading enzyme in the brain, is thus regarded as a potential therapeutic target for AD. NEP has also been known as a cell surface antigen of mesenchymal stem cells (MSCs). Here, we report human adipose tissue-derived MSCs (ADSCs) secreted 150–200-nm sized exosome-like vesicles carrying NEP protein. Enzyme activity assay using fluorogenic substrate Mca-RPPGFSAFK(Dnp) and a selective NEP inhibitor thiorphan revealed that these vesicles had NEP-specific enzyme activity. NEP-specific activity levels of 1 mg-protein of ADSC-derived exosome-like vesicles was equivalent to that of ∼0.3 ng recombinant human NEP. Remarkably, we also found that ADSCs expressed NEP at a clearly higher level than bone marrow-derived MSCs, indicating that ADSCs are the reliable source of NEP-bound exosome-like vesicles. Given the recent reports that microvesicles potentially cross the blood-brain barrier, the present results suggest a new therapeutic direction for a delivery strategy of NEP using microvesicles.

Funded by: A grant-in-aid for the Third-Term Comprehensive 10-Year Strategy for Cancer Control of Japan, a grant-in-aid for Scientific Research on Priority Areas Cancer from the Japanese Ministry of Education, Culture, Sports, Science and Technology, a grant-in aid for cancer research promotion from National Cancer Center of Japan, grant-in-aid for Japan Society for the Promotion of Science (JSPS) Fellows and the Program for Promotion of Fundamental Studies in Health Sciences of the National Institute of Biomedical Innovation (NiBio) of Japan

**8. Hypoxia increases lysyl oxidase like 2 abundance on endothelial cell-derived extracellular vesicles**

O.G. De Jong^1^, Y. Chen^2^, M. Gucek^2^, M.C. Verhaar^1^ and B.W.M. van Balkom^1^

^1^Nephrology and Hypertension, UMC Utrecht, Utrecht, The Netherlands; ^2^NHLBI, NIH, Bethesda, MD, USA

Email: o.g.dejong@umcutrecht.nl

Endothelial cells (EC) produce extracellular vesicles (EVs), which induce effects in their target cells. We hypothesized that hypoxic conditions influence the constitution of EC-derived EVs. We performed a quantitative proteomics study on EVs derived from EC cultured under normoxia or hypoxia (2% O2). We identified 1385 proteins in EC-derived EVs, of which 5 were up- and 6 were downregulated when ECs were cultured in hypoxic conditions. One of the upregulated proteins, lysyl oxidase like 2 (LOXL2), was of specific interest given its role in extracellular matrix (ECM) remodeling and its association with the formation of tumor metastases. By immunoblotting, we confirmed a 2-fold increase of LOXL2 in EVs grown in 2% O_2_. Sucrose density gradient analysis showed that LOXL2 expression correlated with the density of Flotillin-1, indicating that LOXL2 was present in EVs and not in precipitated protein aggregates. Using electron microscopy, a biotinylation assay, and a proteinase K protection assay, we showed that LOXL2 is present on the surface of EVs, placing it in the same compartment as the ECM, indicating a potential role for EV-associated LOXL2 in ECM remodeling. Altogether, our data suggest that LOXL2 may be delivered through EC-derived EVs for focal ECM remodeling. Given its association with breast tumor metastasis, this process may be implicated in influencing the microenvironment of certain tumors.

Funded by: Netherlands Institute of Regenerative Medicine

**9. Interaction of erythrocyte microvesicles with activated endothelium**

Raymond M. Schiffelers^1^, Grietje Andringa^1^, Richard van Wijk^1^, Jerney Francois^1^, Nikoleta Sachini^1^, Pieter Vader^1^, Marcel H.A.M. Fens^2^ and Wouter W. van Solinge^3^

^1^University Medical Center Utrecht, Utrecht, The Netherlands; ^2^Children's Hospital Oakland Research Institute, San Francisco, CA, USA; ^3^University Medical Center Utrecht, Utrecht, The Netherlands

Email: r.schiffelers@umcutrecht.nl

Erythrocytes constantly shed microvesicles. Shedding of these vesicles ensures longevity of the red blood cells in the circulation as they clear the cell surface of exposed phosphatidylserine. Phosphatidylserine is one of the molecules mediating erythrocyte clearance. Microvesicle shedding can be further stimulated by a variety of triggers: addition of ionophore, oxidative stress, fever (42°C), and pharmaceuticals. All triggers result in the formation of phosphatidylserine-positive microvesicles of around ∼0.2 µm that contain hemoglobin. These vesicles can be taken up by endothelial cells. Uptake is increased when the vesicles are preincubated with MFG-E8/lactadherin, a protein that bridges phosphatidylserine and alpha v-integrins. In response to vesicle internalization, endothelial cells alter expression of a variety of genes, including genes involved in iron processing and recycling. We hypothesize that the increased production of erythrocyte microvesicles under specific conditions can contribute to disease by changing endothelial cell behavior.

Funded by: ERC Starting Grant 260727

**10. 3D in vitro model of angiogenesis as a platform for analysis of endothelial cell-derived microvesicles**

Benjamin D. Zeitlin^1^ and Jacques E. Nor^2^

^1^Pacific Dugoni School of Dentistry, San Francisco, CA, USA; ^2^School of Dentistry, University of Michigan, Ann Arbor, MI, USA

Email: bzeitlin@pacific.edu

The aim of this study was to determine the utility of an in vitro 3D model of endothelial cell (EC) angiogenic sprouting for the microscopic study of EC microvesicle (MV) release and cellular interaction. Few protocols allow simple observation of cell interaction with MVs in vitro. For those that do, the focus is primarily on cellular response to MVs from other cell types. ECs are well known to release both MVs and exosomes when activated. Also, non–EC-derived extracellular vesicles may stimulate angiogenesis but less is known of the action of EC-derived MVs on other ECs. Herein, ECs were encapsulated in solid droplets of a peptide matrix. ECs formed networks in the droplets prior to embedding in collagen. Proangiogenic mediators induced planar cell invasion and growth of capillary-like sprouts. MV release and cellular interaction were observed microscopically using either differential interference contrast (DIC), Nikon advanced modulation contrast (NAMC), normal or confocal fluorescence. The EC release and uptake of MVs, estimated to be ∼1 µm in diameter, were readily observable by eye using the 3D planar model with either DIC or NAMC. Actin probes clearly defined vesicles by confocal fluorescence, yet more variable results were obtained by fluorescent microscopy with probes to EC markers. This planar 3D in vitro model of angiogenesis is a useful tool for investigating the interaction of ECs with MVs around 1 µm in diameter. It also allows for fluorescent labeling for specific markers of both cells and MVs of ∼1 µm or potentially smaller.

Funded by: Pacific Dugoni School of Dentistry Research Enhancement Award Grant (BDZ) Grant P50-CA97248 (University of Michigan Head & Neck SPORE) from the NIH/NCI and grants R01-DE14601, R01-DE15948, R01-DE16586, R21-DE19279 and R01DE021139 from the NIH/NIDCR (JEN).

**11. Screening a library of shRNA to ESCRT components to analyze their effect on exosome secretion**

Graça Raposo^1,2^, Marina Colombo^1–3^ and Clotilde Théry^1,3^

^1^Institut Curie; ^2^CNRS UMR144; ^3^INSERM U932, Paris, France

Email: graca.raposo@curie.fr

Exosomes correspond to the small intraluminal vesicles (ILVs) of endosomal compartments called multivesicular bodies (MVBs), which are formed by inward budding of the endosomal limiting membrane. The molecular mechanisms involved in the formation of ILVs are still poorly understood in eukaryotic cells: several mechanisms have been described, dependent or not on the ESCRT machinery, a family of four protein complexes that have been involved in cargo sorting and MVB biogenesis. The aim of this study is to assess the involvement of the different ESCRT proteins in exosome biogenesis in HeLa-CIITA cells. A medium-throughput screening was performed using a FACS-based assay, which allows relative quantification of CD63- and MHC class II-bearing exosomes in the supernatant of cells treated with small-interfering RNA. shRNA to seven of these proteins were shown to affect exosome secretion: whereas knock-down of HRS, STAM and TSG101 induced a decrease, knock-down of ALIX, CHMP4C, VTA1 and VPS4B caused an increase in the amount of secreted exosomes as compared to the control. Protein quantification and Western blotting performed on exosomes purified by ultracentrifugation revealed that silencing of ALIX induced an increase in the MHC class II content in the exosomes, rather than of their total amount. Immunoelectron microscopy analysis indicated that vesicles released by ALIX KD cells were larger in size and showed increased labeling for MHC class II as compared to the control and that MVBs from ALIX KD cells accumulate MHC II molecules. These observations reveal ALIX as a candidate involved in MHC II sorting and exosome biogenesis, which could be of potential use as a means to increase immunogenicity of vesicles secreted by antigen-presenting cells.

Funded by: Institut national du cancer, Agence nationale de la recherche, Institut Curie, Institut national de la santé et de la recherche médicale, Centre national de la recherche scientifique, France.

#### 2. Isolation and Characterization

**12. A novel method for trapping serum microvesicles**

Chihchen Chen and Nithin Devarajulu Palavalli

National Tsing Hua University, Institute of NanoEngineering and MicroSystems Hsinchu, Taiwan

Email: chihchen@mx.nthu.edu.tw

Cellular stimulation, oncogenic transformation and death of cells cause the release of microvesicles into their surrounding environment like body fluids and blood. The microvesicles, carrying nucleic acids, signaling molecules, proteins and proteases, act as mediators for intercellular communications due to their ability to navigate and to transport molecules containing bioactive information to recipient cells. Cancer cells tend to shed higher quantities of microvesicles compared with healthy cells. Hence, isolated microvesicles could be used as biomarker tools for clinical applications. We propose a new method for the enhanced sorting of selective microvesicles from serum samples. With the help of nanostructures, which corresponds to the size of microvesicles, high purification yields can be produced along with high quality extracted nucleic acids to be designated as biomarkers for cancer detection and diagnosis.

Funded by: NSC 99-2320-B-007 -005 – MY2, Taiwan National Science Council

**13. The direct and real time visualization, sizing, phenotyping and counting of exosomes: principles and methodology of nanoparticle tracking analysis (NTA)**

Bob Carr, Andrew Malloy, Agnieszka Siupa, Claire Hannell, Patrick Hole, Matthew Wright and Jeremy Warren

NanoSight, Ltd, Amesbury, Wiltshire, United Kingdom

Email: bob.carr@nanosight.com

Research into, and exploitation of, extracellular exosomes and microvesicles is currently hampered by a lack of easy and rapid methods for their detection, characterization and enumeration. Existing techniques such as EM, AFM, flow cytometry or DLS each have their advantages but cannot individually fulfill all the requirements necessary. The new laser-based, multiparameter technique of NTA allows users to directly visualize, on an individual basis, microvesicles and exosomes as small as 30 nm. It is possible to rapidly and simultaneously determine their size and high-resolution particle size distribution by analysis of their Brownian motion and to count the number of particles of any given size in a sample through analysis of a video of the laser-illuminated sample in real-time. Fluorescently labeled exosomes and microvesicles can be separately tracked in complex backgrounds allowing them to be phenotyped. Furthermore, the zeta-potential of each particle seen can be simultaneously determined. Following isolation by conventional methods, sample preparation is minimal requiring simple dilution. The principles and methodology of NTA will be described and examples of its application in the study of exosomes outlined.

Funded by: NanoSight, Ltd.

**14. Nanopore-based detection: improved multiparameter measurement of extracellular vesicles**

Clement Roux

IZON, Christchurch, New Zealand

Email: clement.roux@izon.com

Nanopore-based detection utilizing size tunable nanopores has been developed for accurate multiparameter measurement of a wide range of naturally occurring and synthetic micro- and nanosized vesicle types. The technique measures the resistive pulses that occur when particles pass through a size-tunable nanopore via electric field and/or with pressure, transiently disrupting current flow through the pore. Interrogation of these signals allows rapid and accurate determination of the concentration (particles/ml), size distribution, aggregation levels and relative surface charge distribution of particles under physiologically relevant conditions. Nanopore-based detection provides increased sensitivity compared to current methods for vesicle analysis such as flow cytometry, which are limited in their ability to accurately detect submicron-sized particles. The ability to individually analyze each particle also addresses the shortcomings of ensemble systems such as dynamic light-scattering and of static systems using electron microscopy. The approach has been applied for accurate characterization of a wide variety of vesicle types including drug delivery particles (liposomes) and biologically occurring vesicles, such as exosomes and microparticles.

Funded by: MacDiarmid Institute for Advanced Materials and Nanotechnology and Izon Science

**15. Improvement of microparticle detection using a new high-sensitivity routine flow cytometer: exploring the hidden part of the iceberg**

L. Statello, J. Wahlgren, M. Purrello and H. Valadi

^1^Department of Gian Filippo Ingrassia, Unità di BioMedicina Molecolare Genomica e dei Sistemi Complessi, Genetica, Biologia Computazionale, University of Catania, Italy; ^2^Department of Rheumatology and Inflammation Research, University of Gothenburg, Gothenburg, Sweden

Email: romaric.lacroix@univmed.fr

Cellular microparticles (MP) are promising biomarkers in many pathological situations. Although widely used for their measurement, flow cytometry (FCM) has raised controversies since the lowest MP size falls below the detection limit of “standard” flow cytometry (sd-FCM). Following recent technological improvements leading to higher sensitivity FCM (hs-FCM), our objectives were (1) to evaluate the potential of hs-FCM for extended MP detection, (2) to set up a standardized protocol for MP enumeration and (3) to compare MP counts obtained with both sensitivity levels. Compared to sd-FCM, hs-FCM displayed improved forward scatter (FS) resolution and lower background noise allowing to discriminate previously undetectable small MP in plasma samples. Using fluorescent beads with appropriate sizes (0.1/0.3/0.5/0.9 µm) and relative amounts, a new 5 colors standardized hs-FCM MP protocol was setup and provided reproducible MP counts. Applied to coronary patient samples, it resulted into 8- to 20-fold increases in MP counts as compared to sd-FCM. Interestingly, the ratio between small and large MP varied not only according to clinical status but also depending on MP subset, suggesting access to new biological information. Recent improvements in FCM provide access to previously undetectable MP and represent a new opportunity to enhance their impact as biomarkers in clinical practice.

Funded by: Aix-Marseille University

**16. Lectins as tools for exosome characterization**

Maja M. Kosanovic and Miroslava M. Jankovic

University of Belgrade, Institute for the Application of Nuclear Energy, INEP, Serbia

Email: maja@inep.co.rs

Characterization of exosomes, aimed at revealing their function and biomarker potential, is still ongoing. Most studies deal with protein and RNA aspects of these membrane vesicles. Since exosomes have a specific glycoprotein composition, analysis of this should be relevant for their characterization. However, there have been few such studies so far. The aim of this work was to create assays for characterization of exosomes, exploiting lectins as carbohydrate binding proteins. Normal urinary exosomes were isolated using differential centrifugation and ultrafiltration. Interaction with plant lectins was examined using scanning electron microscopy and a solid phase test with immobilized exosomes. Simple and reliable assays for exosome analysis were established and validated experimentally. The results obtained, in both experimental systems, showed that lectins bound to exosomes specifically and such interactions were dependent on the specificity of the lectin used. Since changes in glycosylation often occur in human diseases, the lectin binding pattern of normal urine exosomes can be employed as a reference, directing further work to comparative analysis of exosomes in different pathological conditions.

Funded by: Ministry of Education and Science of Republic of Serbia, grant 173010

**17. Preliminary studies: preparation and handling of plasma samples for MP activity assays**

Ditte Sørensen, Shona Pedersen and Søren Risom Kristensen

Department of Clinical Biochemical Medicine, Aalborg Sygehus Syd, Forskningens Hus, Søndre Skovvej, Aalborg

Email: dsoer07@student.aau.dk

Studies are on going to identify microparticle (MP) populations and develop methods to quantify these, as MP can serve as diagnostic and prognostic biomarkers of disease. However, the potential for MP as biomarkers has been limited by inadequate validation and standardization of sample preparation and handling, reagents and assays. The commercial Zymuphen Microparticle Activity assay and the Zymuphen MP-TF assay claim to quantify phosphatidylserine positive MP and tissue factor bearing MP, respectively, present in plasma. In order to move toward standardized handling of the assays, the centrifugation settings recommended by the manufacturer was compared to the one most prominent in the literature and the one used at our department, in order to see if the one from the literature could be preferred. Plasma from healthy men and women were studied. All three centrifugation settings gave platelet free plasma; however, the amount of phosphatidylserine positive microparticles registered by the assay differed within samples from the same person, suggesting that there might be some platelet residues left due to centrifugation. Additionally to centrifugation, the difference between analyzing fresh and frozen plasma, the difference between using manual and automated wash during assays conduction and the difference between plasma from healthy persons and patients will be investigated.

Funded by: Department of Clinical Biochemical Medicine, Aalborg Sygehus Syd

**18. Isolation of microvesicles and exosomes by filtration and estimation of normal reference range in blood plasma**

Ryan Grant^1^, Ephraim Ansa-Addoa^1^, Dan Strattona^1^, Samuel Antwi-Baffour^1,2^, Samireh Jorfi^1^, Sharad Kholia^1^, Lizelle Krige^3^, Sigrun Lange^4^ and Jameel Inal^1^

^1^Cellular and Molecular Immunology Research Centre, Faculty of Life Sciences, London Metropolitan University, London, United Kingdom; ^2^Hammersmith Medicines Research, London, United Kingdom; ^3^Immunohaematology Research Centre, School of Allied Health Sciences, University of Ghana, Accra, Ghana; ^4^University College London Institute for Women's Health, Maternal and Fetal Medicine, United Kingdom

Email: ryg0007@londonmet.ac.uk

Current protocols for the isolation of microvesicles (MVs) and exosomes, which in the main focus on differential centrifugation, vary considerably. In an attempt to set a new standard, we describe a filtration protocol for isolating phosphatidylserine-positive MVs (larger than 200 nm in diameter) and exosomes. The key preparative step to successfully isolate both MVs and exosomes to a high degree of purity was a gentle sonication to break up exosome clumps. Filtration through a 200 nm pore size Millipore filter allowed for collection of exosomes in the filtrate. The larger MVs could then be recovered from the filter. Annexin V-PE MVs were sized and quantified using Polysciences Polybead Microspheres (200 nm) and BDTrucount tubes, respectively, on a FACS CaliburTM flow cytometer. The normal reference range from normal human donors was found to be 0.51–2.82×10^5^ MVs/ml. Freeze/thawing of samples had little effect on MV counts and with age MV levels seemed only marginally reduced. Fasting status also affected MV levels, appearing up to 3-fold higher in fasting individuals. Smokers had reduced MV counts and nicotine reduced MV release from THP-1 cells.

Funded by: Cellular and Molecular Immunology Research Centre/Faculty of Life Sciences and Hammersmith Medicines Research. S.K. received a VC scholarship (London metropolitan University).

**19. The pellet and the pellet cap of unfiltered extracellular vesicles have different characteristics and RNA content**

Cecilia Lässer, Margareta Sjöstrand and Jan Lötvall

Krefting Research Centre, Department of Internal Medicine, University of Gothenburg, Gothenburg, Sweden

Email: cecilia.lasser@gu.se

*Background*: A variety of vesicles can be released by cells into its surrounding environment. To obtain an exosome fraction with little contamination, an appropriate isolation method is crucial. At the moment many different isolation protocols are used and favored by different researchers. We have previously suggested that a 200-nm filtration before the final 120,000g ultracentrifugation is a valuable method of eliminating larger particles. The aim of this study was evaluate the impact of the filtration step on the RNA profile of the isolated exosome fraction. *Method*: Exosomes were analyzed from the human mast cell line, HMC-1. Cell, debris and larger vesicles were eliminated by a 300g and a 16,500g centrifugation. At this point, half of the supernatant were filtered through a 200-nm filter, while the rest of the supernatant were just transferred to new tubes. The exosomes were pelleted by a 120,000g ultracentrifugation. The RNA was isolated with the miRCURY™ RNA Isolation Kit from Exiqon and analyzed with a Bioanalyzer. *Results*: For the samples isolated without the filtration step, we could separate two fractions of the ultracentrifuged pellet, one that was firmly attached to the tube wall, and a second pellet being gelatinous and non-fixed (cap). The gelatinous pellet cap could easily be separated from the firmly fixed pellet using a pipette. The gelatinous pellet cap was never seen in the sample where the nanofiltration was used. When the RNA profile was analyzed, it was shown that the pellet from the samples that have been filtrated contained no or little rRNA. In the non-filtered samples, RNA analysis showed that the pellet firmly attached to the tube wall showed small amounts of rRNA and the gelatinous extra pellet cap the RNA profile showed large amounts of rRNA. *Conclusion*: The method used for isolating exosomes affects the RNA profile of the exosome fraction. This needs to be further investigated to find the most suitable method for isolating exosomes, as well to be able to compare results between different studies.

Funded by: The Swedish Research Council, the Swedish Heart and Lung Foundation, the Swedish Asthma- and Allergy Foundation and VBG GROUP'S Herman Krefting Foundation for Allergy and Asthma Research

**20. Impact of preanalytical parameters on the measurement of circulating microparticles: toward standardization of protocol**

Romaric Lacroix, Coralie Judicone, Philippe Poncelet, Stephane Robert, Laurent Arnaud, José Sampol and Françoise Dignat-George

UMR-S 608 INSERM-Aix-Marseille Université, UFR de Pharmacie, Marseille, France; Laboratoire d'hématologie, CHU Conception, AP-HM, Marseille, France; Biocytex, 140, Chemin de l'Armée d'Afrique, Marseille, France

Email: romaric.lacroix@univmed.fr

Microparticles (MP) are small vesicles of 0.1–1 µm, released in response to activation or apoptosis. Over the past decade, they received an increasing interest both as biomarkers and biovectors in coagulation, inflammation and cancer. Clinical studies were conducted to assess their contribution to the identification of patients at cardiovascular risk. However, among the limitation of such studies, preanalytical steps remains an important source of variability and artifacts in MP analysis. Because data from the literature are insufficient to establish recommendations, our objective was to assess the impact of various preanalytical parameters on MP measurement. These parameters included the type of collection tube, phlebotomy conditions, transportation practices, centrifugation steps and freezing. MP were assessed by three methods: flow cytometry using a standardized approach, a thrombin generation test (Calibrated Automated Thrombogram^®^) and procoagulant phospholipid-dependent clotting time assay (STA^®^-Procoag-PPL). Main results show that the three major preanalytical parameters, which impact on MP-related data are the delay before the first centrifugation, agitation of the tubes during transportation and the centrifugation protocol. Based on both this work and literature data, we proposed a new protocol that is being challenged in a multicenter study including 15 laboratories. Standardizing the preanalytics of MP analysis is a mandatory step for the relevant evaluation of this potential biomarker in clinical studies.

Funded by: Aix-Marseille University

**21. A crowdsourcing approach to find consensus on a reasonable methodology to isolate exosomes**

Jan Lötvall, Maria Eldh, Cecilia Lässer and Clotilde Thery; for 263 members of the for the ISEV Community

Krefting Research Centre, University of Gothenburg, Sweden; Institut Curie, INSERM U932, Immunité et cancer, Paris, France

Email: jan.lotvall@gu.se

There are diverse opinions on how exosomes ideally shall be isolated and/or enriched. We reached out to a wide group of experts in the area, in an attempt to achieve “reasonable consensus” on how exosomes can be isolated, with the assumption that the collective experts will reach a reasonable consensus. Such collective intelligence is used in many areas and is defined as a shared intelligence that emerges from the collaboration of a large group of individuals with specific competence or experience. Crowdsourcing is a version of collective intelligence that includes the assumption of awards for some of the participants, and this award can be pecuniary or figurative (or both). A questionnaire with a total of 48 questions, focusing on the appropriateness of different steps to isolate exosomes, was developed and circulated by email to 583 individuals who either attended or were invited to the International Workshop of Exosomes in Paris in January 2011 or published in the exosomes field over the last 3 years prior to May 20, 2011 (PubMed: search term “exosomes”). The questionnaire was circulated between the May 26 and June 26, 2011, and 263 responses were received of which 227 finished the survey. A majority of respondents were from Europe 69%, followed by North America 21%; 80% or respondents felt that exosomes “have to” come from the endosomal pathway, and 66% voted that they cannot be shedded from the plasma membrane. A small majority suggested that exosomes must be < 100 nm in diameter, and scoring suggested that it is important to distinguish exosomes from other extracellular vesicles primarily not only for identification/characterization but also for studies of biological effect, biomarker discovery and/or therapy/vaccine/gene therapy. No consensus could be reached about the appropriateness of diluting the source of exosomes prior to isolation or whether to use filter techniques or size exclusion chromatography for the isolation of exosomes. Very strong consensus could, however, be reached on the appropriateness of different centrifugation steps for the isolation of exosomes. A small majority suggested that using sucrose gradient was appropriate, but some arguments were brought forward that it may not be necessary and that this approach may lead to lower yield. Sucrose cushion was mentioned as an alternative approach. Only 30% of respondent use sucrose-based methodology as a standard approach to isolate exosomes. A small majority thought that immunoaffinity could be an appropriate methodlogy to isolate exosomes, but ExoQuick was not felt to be appropriate by a majority of respondents. If exosomes were harvested from cell cultures needing FCS, a majority felt that the used FCS should be ultracentrifuged for at least 6 hours or longer to avoid contamination of FCS exosomes. Overall, reasonable consensus could be reached regarding the utilization of different methods for exosomes isolation, with some disagreement on the need of using sucrose gradient and/or filtration steps.

Funded by: VBG Groups Centre For Asthma- and Allergy Research, The Swedish Research Council.

**22. Isolation of tumor-associated exosomes from clinical samples using the ultrafiltration method**

Wei-Lun Huang, Chien-Chung Lin and Wu-Chou Su

Department of Internal Medicine, National Cheng Kung University College of Medicine and Hospital, Tainan, Taiwan

Email: r4215652@gmail.com

Exosomes are nanometer-sized vesicles released in body fluids to mediate cell communication. Exosomes have been isolated by different methods based on the physical, chemical and biological properties. The ultrafiltration (UF) method can potentially separate exosomes rapidly based on the characteristics of the physical size. The procedure can be carried out by regular centrifuges instead of ultracentrifuges. The UF method has been shown to be reliable for isolating exosomes from urine samples, but it has not been tested for its application in tumor samples. In the current study, we try to show that the UF method is ideal for isolating tumor-associated exosomes from clinical specimens. First, we tested the UF method to isolate exosomes from conditioned media of the lung cancer cell line-AS2 and compared it with the ultracentrifugation method. The two methods showed similar results. TEM revealed that the exosomes were closed vesicles of nanometer size. Exosome markers including Alix, CD63 and Tsg101 were detected in the isolated particles. Nanosight system revealed that the size distribution of the main population of particles were from 30 to 150 nm, fitting well to the definition of exosome. Similar results were showed in other lung cancer cell lines as well as cancer cells and immune cells derived from clinical malignant pleura effusion (MPE) samples. Finally, we isolated exosomes from clinical MPE and serum samples of lung cancer patients. These results demonstrated that the UF method is ideal for isolating tumor-associated exosomes from clinical samples.

Funded by: Comprehensive Cancer Center in Southern Taiwan, DOH101-TD-C-111-003

**23. Exosome immunocapturing assay (EXOTEST™) enables accurate quantitative analysis of unfractioned plasma samples**

T. Oja, A.L. Kubo, K. Talpsepp, N. Zarovni and A. Chiesi

HansaBioMed OU, Tallinn, Estonia

Email: antonio.chiesi@hansabiomed.eu

Use of exosomes and exosome displayed molecules as diagnostic sensors for assessment of functional state of parent cell in basic and clinical research is currently hampered by the lack of reliable, affordable and optimized tools for quantitative analysis of exosomes in complex biological samples (e.g., plasma or urine). Here, we report the use of an ELISA-based method that permits isolation and concentration of exosomes of specific origin prior to determining their molecular profile (ExoTESTTM). This assay enables direct capture and quantification of exosomes from human plasma samples that were previously precleared by low speed centrifugation or microfiltration, without prior chemical precipitation or ultracentrifugation. Specific capture and analysis of overall exosomes, or exosome subpopulations of interest, is ensured by selected combinations of capture and detection antibodies against persistently expressed exosomal markers (i.e., Rabs or tetraspanins) or against tissue-specific markers (i.e., tumor markers). The reliability and sensitivity of such analysis of unfractioned samples was assessed by parallel analysis of exosomes purified by differential ultracentrifugation (gold standard). As a synergistic and complementary approach to sample fractionation and concentration, we included as an upstream step, the incubation of a sample with antibodies-conjugated beads that were subsequently analyzed on ELISA plates. This immune-capturing approach provides double-targeted selective enrichment of desired exosome population and increases overall sensitivity and specificity of the assay. Such assays are appealing cost-effective and friendly to use solutions for routine applications in research and clinical laboratories, avoiding the use of capital equipment and extensive sample handling.

Funded by: EAS EU29269

**24. The effect of red blood cells on microparticle transport to glass surfaces under flow**

Y.-H. Lee^1^, A. Fogelson^2^ and V. Turitto^1^

^1^Illinois Institute of Technology, Chicago, IL, USA; ^2^University of Utah, Salt Lake City, UT, USA

Email: leeyinghui0217@gmail.com

Elevated monocyte-derived MPs have been found to correlate with thrombotic complications. During thrombus propagation, these MPs may be delivered to the vascular injury site and influence the process of thrombosis. MPs are submicron membrane vesicles that may have increased transport and/or binding capabilities compared to platelets or other cell types due to their decreased size and their higher Brownian diffusion. Little is known of the mechanisms by which MPs are transported to and impart their biological activity on surfaces, especially in blood. We had previously observed that monocyte-derived MP adhesion to glass surfaces was strongly shear-dependent. In addition to flow (shear) rate, red blood cells (RBCs) may enhance MP adhesion due to the complex movement of RBCs under flow in a manner similar to enhanced platelet transport. The extent to which MP adhesion can be enhanced by RBCs similar to platelets was investigated using a parallel flow chamber. Fluorescently labeled MP suspensions containing 0%, 15% and 40% RBCs were perfused over a glass surface at 400 s^−1^ and images were taken every 10 s up to 5 minutes. The initial studies show no significant difference in MP adhesion for the three RBC concentrations studied. To clarify possible binding interactions between MPs and RBCs, the number of particles in supernatant of the 40% MP-RBC mixture, and the 0% MP solution were compared after centrifugation at 3000g for 10 minutes using nanoparticle tracking analysis. We found increased particle concentration in the supernatant from 40% RBCs, presumably produced from RBCs during the experimental processing; the extent to which MPs and particles from RBCs influence the adhesion of MPs needs to be determined. Future experiments will delineate the importance of RBC on the MP transport mechanism and the extent to which activation of blood coagulation is induced by MP adhesion on the surface.

Funded by: National Institutes of Health (NIH R01GM090203) and the Pritzker Institute of Biomedical Science and Engineering at IIT

**25. The influence of low intensity resistance exercise on circulating endothelial microparticles (EMPs)**

Mark Ross, Antony Wekesa, John Phelan and Michael Harrison

Biomedical Research Cluster, Department of Chemical and Life Sciences, Department of Health, Sport and Exercise Sciences, Waterford Institute of Technology, Waterford, Ireland

Email: jpphelan@wit.ie

*Introduction*: The endothelium plays an important role in the maintenance of vascular and cellular homeostasis. Endothelial microparticles (EMPs) are “blebs” of endothelial cell membranes, which “bud” from endothelial cells due to a variety of stimuli, e.g., cell activation, apoptosis, vascular injury etc. These microparticles in turn act as biovectors as they express cell surface signaling markers, which can induce various cellular responses in adjacent cells. Thus, these EMPs can act as sensitive biomarkers of endothelial status. *Hypothesis*: We propose a high volume low intensity exercise exerts an effect on circulating EMPs. *Method*: Thirteen healthy resistance trained young men undertook an acute bout of high volume low intensity resistance-exercise. Peripheral bloods were taken at preexercise and at and 10 minutes, 2 hours and 24 hours postexercise. EMPs reflecting endothelial cell activation (CD62E + EMP) and apoptosis (CD144 + EMP) were measured using flow cytometry. *Results:* CD62E + EMP levels but not CD144 + EMP levels increased postexercise with the former significantly higher at 24 hours postexercise. These changes were accompanied by increases in the serum markers; VEGF and G-CSF. Other MP subsets were not changed. *Conclusion*: Our data suggest that resistance training can increase the expression of the activation marker CD62E+ on endothelial cells leading to increased endothelial cell stress as a result of resistance exercise for adaption purposes. Mechanistically, the observed increase in the activation marker CD62E + could facilitate the binding of endothelial progenitor cells to the endothelium, thus promoting localized cellular regeneration.

Funded by: Strand III funding

**26. SMR-derived peptide disrupts HIV-1 Nef's interaction with mortalin and blocks virus and Nef exosome release**

Vincent C. Bond, Martin N. Shelton, Ming-Bo Huang, Syed A. Ali and Michael D. Powell

Department of Microbiology, Biochemistry, Immunology, Morehouse School of Medicine, Atlanta, GA, USA

Email: vbond@msm.edu

Nef is secreted from infected cells in exosomes and is found in abundance in the sera of HIV-infected individuals. Secreted exosomal Nef (exNef) induces apoptosis in uninfected CD4 T cells and may be a key component of HIV pathogenesis. The exosomal pathway has been implicated in HIV-1 virus release, suggesting a possible link between these two processes. We have previously described a Nef motif, the secretion modification region (SMR; amino acids 66–70), which is required for exNef secretion. We hypothesized that the Nef SMR binds a cellular protein involved in protein trafficking and that inhibition of this interaction would abrogate exNef secretion. By using tandem mass spectrometry and coimmunoprecipitation with a novel SMR-based peptide (SMRwt) that blocks exNef secretion and HIV-1 virus release, we identified mortalin as an SMR-specific cellular protein. A second set of coimmunoprecipitation experiments with full-length Nef confirmed that mortalin interacts with Nef via the SMR motif and that this interaction is disrupted by the SMRwt peptide. Overexpression and microRNA knockdown of mortalin revealed a positive correlation between exNef secretion levels and mortalin protein expression. Using antibody inhibition, we demonstrated that the Nef/mortalin interaction is necessary for exNef secretion. This work constitutes a significant step in understanding the mechanisms underlying exNef secretion, identifies a novel host-pathogen interaction and introduces an HIV-derived peptide with antiviral properties.

Funded by: NIH/NIAID/NRSA grant F31 AI091484, NIH/NIGMS/MBRS grant R25 GM058268, NIH/NIGMS/MBRS grant S06 GM08248, NIH/NCRR/RCMI grant G12-RR03034, Georgia Research Alliance funding grant GRA.VAC08.W and Emory CFAR grant P30 A1050409. This investigation was conducted in a facility constructed with support from Research Facilities Improvement Grant #C06 RR18386 from NIH/NCRR.

**27. Interaction between exosomes and surfaces of materials**

Kiyotaka Shiba and Kanako Suga

Division of Protein Engineering, Cancer Institute, Japanese Foundation for Cancer Research, Tokyo, Japan

Email: kshiba@jfcr.or.jp

Understanding of the interaction between exosomes and surfaces of various materials is important for the development of diagnosis and characterizing devices of exosome. In this poster, we will present our initial characterizations of interaction between the isolated exosomes (from cell culture) and solid materials (including SiO_2_ and Al_2_O_3_) by using QCM-D technology. We will also present characterizations of EpCAM molecules on exosomes.

Funded by: Japan Science and Technology Agency

**28. Plasmatic microvesicles: their size, morphology, phenotype revealed by cryoelectron microscopy and specific gold labeling**

Alain R. Brisson, Sisareuth Tan, Nicolas Arraud and Céline Gounou

Molecular Imaging & NanoBioTechnology, UMR-CBMN University Bordeaux-1, Pessac, France

Email: a.brisson@iecb.u-bordeaux.fr

Our aim is to provide a comprehensive structural description of circulating microvesicles (MVs) found in various body fluids in physiological and pathological situations. In this initial study, we focused on MVs from blood plasma and activated platelets, with the objective of characterizing the morphology and size distribution of the whole MV population as well as the subpopulation of MVs that expose the procoagulant lipid phosphatidylserine (PS). We used cryoelectron microscopy and PS-specific markers made of annexin-A5 covalently coupled to gold nanoparticles. Cryo-EM has the unique advantage to reveal the genuine structure of MVs in pure plasma, in the absence of any treatment, like fixation, staining or drying. Plasmatic MVs could be classified into three major morphological families, namely isolated MVs, MVs made of clusters of vesicles and aggregate-like particles. Platelet-derived MVs range in size from 50 nm to 3 µm. Their size histogram was determined, showing that 75% of MVs are smaller than 500 nm. The subpopulation of PS-exposing MVs constitutes 70% of the total MV population. About 50% of MVs smaller than 400 nm are not labeled by annexin-A5. This study provides novel structural information on plasmatic MVs and opens avenues for characterizing MVs in various physiopathological situations. These results and their perspectives will be discussed in the context of previous studies.

Funded by: ANR

**29. Impact of sampling and isolation procedures on concentration of microvesicles in blood isolates**

E. Ogorevc^1^, R. Štukelj^2^, A. Zavec-Bedina^3^, V. Šuštar4, U. Škufca^2^, A. Mrvar-Brečko^5^ and V. Kralj-Iglič^2^

^1^Laboratory of Biophysics, Faculty of Electrical Engineering, University of Ljubljana, Slovenia; ^2^Group for Biomedical Research, Faculty of Health Sciences, University of Ljubljana, Slovenia; ^3^Laboratory of Biosynthesis and Biotransformation, National Institute of Chemistry, Ljubljana Slovenia; ^4^Laboratory of Clinical Biophysics, Faculty of Medicine, University of Ljubljana, Slovenia; ^5^Department of Anesthesiology and Intensive Care, University Medical Centre Ljubljana, Slovenia

Email: eva_ogorevc@yahoo.com

Analysis of the number and composition of microvesicles (MVs) is a proposed potential method for diagnosis and treatment of diseases where invasive diagnostic procedures are now necessary. However, a clinically relevant method for isolation of MVs from blood has not yet been agreed upon as the mechanisms of vesiculation and the processes that occur during the isolation procedure are poorly understood. The aim of our study was to reveal the morphology, identity and origin of particles in isolated material and to evaluate the impact of several external parameters during sampling and isolation (temperature, sample volume and the properties of the needle) on concentration of MVs in blood isolates. Blood was taken from volunteers and MVs were isolated from fresh samples by repeated centrifugation and washing. To identify isolated material, isolates were visualized by scanning electron microscopy, analyzed by flow cytometry and calculated theoretically. To study the effect of the blood sampling, different needles varying in thickness and length, and different sample volumes were used at different temperatures (4°C, 20°C and 37°C). Numerous microparticles heterogeneous in size and shape as well as residual blood cells were found in blood isolates. Comparison of MV shapes with theoretical shapes obtained by minimization of the membrane-free energy showed good agreement. The concentration and size of MVs were sensitive to temperature during isolation process. We demonstrated that the concentration of MVs was higher while the MVs were on average smaller at lower temperatures. Concentration of MVs in isolates increased with the volume of sampled blood (effect depending on temperature) and was proportional to the work of shear force at the inner wall of the needle The corresponding correlations were statistically significant. Sensitivity of the concentration and size of MVs to the external parameters such as temperature, sample volume and the type of the needle during isolation process indicates that a large pool of MVs is created after blood sampling. The contents of isolates reflect the properties of the blood cells and their interaction with the surroundings (rather than showing only “circulating” MVs).

Funded by: Slovenian Research Agency

#### 3. Function in Blood and Bone marrow

**30. Characterization of platelet-derived microvesicles**

Maria Aatonen, Mikaela Grönholm and Pia Siljander

Division of Biochemistry and Biotechnology, Department of Biosciences, University of Helsinki, Finland

Email: maria.aatonen@helsinki.fi

Upon activation, platelets release microvesicles (MVs) from both plasma membrane (PMPs) and multivesicular bodies (exosomes). We compared the size distribution, concentration and molecular properties of differently in vitro generated MVs from human platelets. Platelets were isolated with a modified gradient procedure to improve sample purity and activated by various physiological stimuli in comparison to Ca^2 +^-ionophore (A23187). PMV populations were isolated by prevalent differential centrifugation methods. Distribution of PMV sizes was analyzed by nanoparticle tracking analysis (NTA) and electron microscopy (EM). Size distributions by NTA and EM correlated well showing that over 90% of PMVs were < 500 nm and over 70% were < 250 nm irrespective of the agonist. Very few PMVs were detected in the range of 0.5–1 mm, and data from dynamic light scattering (DLS) suggest a large shift of the activated platelets into this area. These findings show that the majority of PMVs are much smaller than previously defined by flow cytometry. Molecular comparison of differentially generated MVs is currently being done by mass spectroscopy and functional studies by PMV-leukocyte interaction studies. Preliminary data suggest qualitative agonist-dependent differences in the PMV-specific cargo, which respectively influence their function. Novel detection methods and a broader understanding of MV physiology are changing our understanding of MP/exosome sizes and properties.

Funded by: The Magnus Ehrnrooth Foundation, The Oskar Öflund Foundation, The Paavo Nurmi Foundation, The Otto Malm Foundation and The Medicinal Foundation Liv och Hälsa.

**31. High-density lipoproteins (HDL) and the plasticizer DINCH suppress formation of platelet-derived extracellular vesicles**

Annika Pienimäki-Römer, Astrid Fischer, Maria Tafelmeier and Evelyn Orsó

Gerd Schmitz Institute for Clinical Chemistry and Transfusion Medicine, University of Regensburg, Germany

Email: annika.pienimaeki-roemer@klinik.uni-regensburg.de

Human platelets are anucleated cells with a short lifespan of 8–10 days in the circulation. Approximately 70–90% of extracellular vesicles in circulating blood are derived from activated or senescent platelets, developing a platelet storage lesion (PSL). Excessive formation and release of platelet-derived EVs (PL-EVs) is critical for vascular repair and a major problem in stored platelet apheresis products for transfusion. We introduced a novel quadruple miniplatelet bag system with low shear-stress (PVC-DINCH foil) to improve the current in vitro platelet storage procedures. In comparison with other bag materials, platelets stored in PVC-DINCH bag show impaired PSL and reduced formation of PL-EVs. Beyond its role in reverse cholesterol transport and cell membrane homeostasis, HDL exert protective effects toward antioxidant, anti-inflammatory and antithrombotic effects, beneficial for megakaryopoiesis, platelet function and platelet senescence. In vitro, HDL3/apo A-I induce extensive proplatelet podia formation and ABCA1-dependent platelet shedding from megakaryocytes. In stored platelets, HDL3 improve platelet membrane microviscosity and antagonize platelet activation, PL-EV release, and modulate senescence-dependent changes in platelet lipidomics, expression of surface markers and micro RNAs (miRNA). Taken together, the low shear-stress PVC-DINCH bag combined with HDL3 treatment significantly suppress release of PL-EVs, correlating negatively with the degree of HDL-particle oxidation, and improve platelet viability.

Funded by: Lipidomic Net

**32. Microparticle-associated tissue factor activity measured with the Zymuphen MP-TF kit and the calibrated automated thrombogram (CAT) assay**

M. Hellum, R. Øvstebø, A.M.S. Trøseid, J.P. Berg, P. Brandtzaeg and C.E. Henriksson

Blood Cell Research Unit, Section for Research, Department of Medical Biochemistry, Oslo University Hospital, Ullevål, Norway

Email: m.s.hellum@medisin.uio.no

*Background*: Microparticles (MPs) are small (<1 µm) shedded membrane vesicles that carry markers from their cell of origin. Monocyte-derived microparticles in the circulation may express tissue factor (TF), the main physiological initiator of blood coagulation. There is an increasing clinical interest for measuring (MP)-associated TF activity owing to its possible role as a prothrombotic biomarker in diseases such as atherosclerosis, diabetes and meningococcal sepsis. However, the methods used to detect MP-associated TF activity are to various extents hampered by lack of standardization and limited published documentation. *Aim*: The aim of this study was to evaluate the performance of two commercially available assays: the recently launched Zymuphen MP-TF kit and the calibrated automated thrombogram (CAT) assay—for measurements of MP-associated TF activity. *Methods*: *Neisseria meningitidis* (Nm), the causative bacteria of meningococcal sepsis with coagulopathy in vivo, induces TF on monocytes and monocyte-derived MPs. We incubated citrated whole blood from healthy volunteers with increasing numbers of heat-inactivated Nm (1×10^2^, 1×10^4^, 1×10^6^ and 1×10^8^ Nm/ml) or vehicle (TBS). After 4 hours (37°C, 15 rpm), plasma was prepared (1500g, 15 minutes + 13,000g, 2 minutes, both at room temperature), and TF activity measured in the CAT assay and the Zymuphen MP-TF kit. *Results*: Nm dose-dependently induced TF-dependent procoagulant activity in the CAT assay; incubating whole blood with 1×10^2^–1×10^8^ bacteria/ml shortened the lagtimes from 20 to 5 minutes. With the Zymuphen TF-MP kit, only the highest Nm-dose (1×10^8^ bacteria/ml) resulted in TF activity above the lowest standard. *Conclusions*: Using plasma obtained from whole blood incubated with Nm, MP-associated TF activity was reflected in a more sensitive manner in the CAT assay than with the Zymuphen MP-TF kit.

Funded by: Oslo University Hospital and University of Oslo

**33. Disseminated intravascular coagulation in critically ill patients: in vitro functional analysis of related changes in microparticle-induced clot formation**

M. van Schilfgaarde^1^, S.V. Oussoren^1^, P.J. Molenaar^1^, R. Nieuwland^2^, A. Sturk, A. Leyte^1^ and J.P.J. Wester^3^

^1^Departments of Haematology and Clinical Chemistry; ^2^Clinical Chemistry ^3^Intensive Care Medicine, Onze Lieve Vrouwe Gasthuis, Academic Medical Centre, Amsterdam, The Netherlands

Email: m.vanschilfgaarde@olvg.nl

*Introduction:* Disseminated intravascular coagulation (DIC) often occurs in critically ill patients with sepsis. Coagulation triggers cells to become activated or to undergo apoptosis, resulting in the release of microparticles. Microparticles are vesicles from cell membranes exposing phosphatidylserine, which facilitates thrombin generation and clot formation. To obtain insight in the role of microparticles in the hemostatic balance in DIC due to sepsis, numbers and origin of microparticles, microparticle-mediated thrombin generation and fibrin generation were investigated. *Methods*: Blood was collected at admission of patients to the intensive care unit and at day 7, when DIC had subsided. Control blood was collected from matched patients at day 1 in the ICU. Each group contained 10 patients. *Results*: DIC patients had less microparticles compared to control patients. At the second blood withdrawal, DIC patients still had low numbers of microparticles compared to controls. Microparticles in DIC patients were of different cellular origin and more often expressed markers related to cellular response to infection than those in control patients. DIC patients showed a significantly less thrombin generation in plasma (*p*<0.01), but thrombin generation per microparticle was increased compared to control patients. Microparticle-dependent fibrin generation results revealed an important role of tissue factor-mediated coagulation in DIC patients but not in control patients. *Conclusions*: DIC patients have lower numbers of circulating microparticles, altered subsets of microparticles, and these microparticles trigger tissue factor-dependent coagulation. In this extreme form of systemic activation of coagulation, procoagulant microparticles not only may be involved in massive coagulation activation leading to depletion of clotting factors but may also be formed and possess more procoagulant capacity secondary to such depletion.

**34. Spectroscopic analysis of microvesicles in clotted and non-clotted blood and urine in healthy, diabetic hypertensive and prostate cancer patients**

Wilfred G. Chen

Urology Research Unit, US Chemicals, Carlton Centre, San Fernando, Trinidad and Tobago

Email: wilfredgchen@hotmail.com

DNA has been regarded as the essential genetic element in every cell. This is based on several studies, the key being that of:

- Avery who in 1944 demonstrated Pneumococcus transformation by DNA
- Crick and Watson who in 1953 proposed the double helix structure of DNA
- Nirenberg, Khoranna and others who in 1961–1966 elucidated the genetic code. Since each cell has its full instructional complement of DNA, there is no need for intercellular exchange of genetic material. DNA was regarded as stable and invariable. This was and is a central biological dogma. Contrary to this dogma, the author Chen proposed in 1968 the Hypothesis of Genetic Exchange, which postulated that “there exists a system of exchange of intercellular and intertissue factors including genetic material (DNA/RNA) between cells and tissues. This is important for the homeostatic control of growth and differentiation. As a corollary to this hypothesis, any disturbance of the exchange system or the messages themselves results in abnormal growth and cancer.” An updated version of this hypothesis has been recently published 2011. The purpose of this study is
- To detect the presence of RNA, DNA, proteins, lipids and glucose in microvesicles, making them possible intercellular carriers of genetic information, in the blood and urine of healthy, hypertensive diabetic and prostate cancer patients, using spectroscopy.
- To determine the effect of EDTA on microvesicles in the urine and clotted blood in these patients. Spectroscopy is an accurate method to determine the structure and biochemical composition of macromolecules both within cells and outside. It involves direct analysis of the specimen, in the standard state, without the disturbances of ultracentrifugation, anticoagulants, presence of liproprotein particles and small platelets. Results revealed the presence of microvesicles with a phospholipid outer membrane enclosing RNA molecules > 250 nucleotides, microRNA 18–28 nucleotides long, DNA molecules > 270 nucleotides, microDNA 22–26 nucleotides long, proteins, lipids and glucose molecules in both clotted and non-clotted blood and urine in healthy, hypertensive diabetic and prostate cancer patients. EDTA reduced the microvesicular abundance in both clotted blood and urine of all patients. EDTA formed complexes with heavy metal and proteins in solution.

*Conclusion*: These results confirmed the presence of RNA, microRNA, DNA, microDNA, lipids, glucose and proteins in microvesicles in blood and urine previously reported by this laboratory. EDTA commonly used in blood collection can affect microvesicles. It is perhaps the first time microDNA is being detected in microvesicles in blood and urine. Its role needs clarification in further studies. It may be concluded that microvesicles contain a repertoire of factors including genetic material making them possible mediators in intercellular communication, supporting Chen's (1968) original Hypothesis of Genetic Exchange.

**35. Persistence of microvesicle-induced gene expression changes in murine marrow cells using an in vitro and in vivo model**

M. Pereira^1^, J.M. Aliotta^1,2^, A. Amaral^1^, M.S. Dooner^1^, K. Brilliant^1^, A. Sorokina^1^, D.C. Hixson^1^ and P.J. Quesenberry^1^

^1^Divisions of Hematology/Oncology; ^2^Pulmonary, Sleep, Critical Care Medicine, Department of Medicine, Rhode Island Hospital, Warren Alpert Medical School of Brown University, Providence, RI, USA

Email: mpereira7@lifespan.org

*Background*: Microvesicles are subcellular entities that are derived from a variety of cells and contain DNA, RNA and protein. We have previously shown that microvesicles isolated from murine lung tissue (lung-derived microvesicles, LDMV) enter murine whole bone marrow cells (WBM) in culture and WBM that has consumed LDMV produce lung-specific mRNA and protein up to 3 weeks later. We wished to determine the persistence of these phenotypic changes in a long-term in vitro culture and an in vivo transplant model. *Methods/Results*: In vivo study: cell-free conditioned media (CM) was made using murine lungs. WBM cells were then co-cultured with either LDMV, minced lung fragments (separated from WBM by a 0.4-micron, cell-impermeable membrane) or neither. One week later, cultured WBM was transplanted into cohorts of lethally irradiated C57BL/6 mice. Recipient mice were sacrificed 6 weeks posttransplant and WBM isolated from each recipient was lysed and examined for the presence of lung-specific genes, including surfactants A, B, C and D (Sp-A, B, C, D), aquaporin-5 (Aq-5) and Clara cell-specific protein (CCSP), via real-time PCR. Mice transplanted with WBM exposed to LDMV express pulmonary epithelial cell genes in the cells of their bone marrow, liver, spleen and thymus 6 weeks after transplantation. In vitro study: CM was made in same fashion as with the in vivo study. One week later, cells were washed and placed into a secondary culture, without lung. WBM was harvested at 2-week intervals and RNA and protein was extracted for real-time PCR and Western blot analysis. WBM maintains expression of pulmonary epithelial cell genes and proteins in vitro up to 12 weeks after exposure to soluble factors released by lung cells. A hybrid rat/mouse co-culture system was used to determine whether mRNA species seen were the product of simple transfer or newly made transcripts. WBM from either rat or mouse is co-cultured with either rat lung, mouse lung, mouse liver or rat liver (or neither, control) opposite a cell-impermeable membrane. After 1 week, inserts are removed and cells are placed into a secondary culture without lung or liver. Samples are collected at the time of replating and every 2 weeks thereafter for RNA isolation and real-time PCR analysis using species-specific primers (Sp-B, Sp-C, albumin and ABC11b). The surfactant specific to the lung donor species is present only at the earliest time point and disappears over time, suggesting that these mRNA species are transferred from the donor lung to the recipient bone marrow cells and are transient. The surfactant specific to the bone marrow species increases expression with time, suggesting that these mRNA species are made de novo by the recipient bone marrow cell. *Conclusion*: These findings suggest that LDMV-induced phenotypic alteration of marrow cells is not a transient phenomenon and that a lung cell-derived transcriptional agent is transferred to marrow cells in culture and responsible for these stable phenotypic changes.

Funded by: 8P20GM103468-04 and 5K08HL086868-04

**36. Role of microvesicles in the adipocyte conversion of osteoblasts inside bone marrow**

Perrine J. Martin^1,2^, Aline Clabaut^1,2^, Olfa Ghali^1,2^, Odile Broux^1,2^ and Pierre Hardouin^1,2^

^1^Univ Lille Nord de France, Lille, France; ^2^ULCO, PMOI, EA4490, Boulogne/Mer, France

Email: Perrine.Martin@univ-littoral.fr

*Background*: Osteoporosis is suspected to be a consequence of significant increase of adipocyte number inside bone marrow, correlated with bone loss. Arguments show that a dialogue between adipocytes and osteoblasts is one of the ways occurring in the competition between mesenchymal stem cells’ (MSC) differentiation routes, supporting adipocyte differentiation at the expense of osteoblast differentiation. It is indeed now well-known that medullary adipocytes secrete adipokines that are mainly regulators of adipocyte, osteoblast and hematopoietic cells development. In addition, we have demonstrated, thanks to a coculture model, that in the presence of MSC-derived adipocytes, MSC-derived osteoblasts express less osteogenic markers but exhibit expression of typical adipogenic genes. Nevertheless, the mechanisms underlying this cellular conversion of osteoblasts toward an adipocyte-like phenotype are not clarified. It has already been shown that adipocytes and MSC can release microvesicles (MV), but this way has not been explored in bone domain yet. Here, we postulate that medullary adipocytes are able to produce MV responsible for the transfer of RNA to osteoblasts, contributing to the phenotype conversion. *Methods*: High amount of MSC were differentiated in adipocytes. After 48 hours in serum-free medium, medium was collected to isolate MV. Adipocyte-specific gene expression (PPARγ, adiponectin, etc.) was measured by real-time PCR on RNA isolated from MV, adipocytes and osteoblasts grown in medium containing adipocyte MV. *Results*: Here, we show that MSC-derived adipocytes are able to release MV containing adipocyte-specific RNA. RNA expression level in osteoblasts cultured in medium containing adipocyte MV is indeed directly proportional to the amount of RNA inside MV. As a result, MVs seem to be one of the way by which adipocytes can influence osteoblast phenotype. They could be a new intercellular communication mode identified inside bone marrow and open new leads in understanding of osteoporosis physiopathology and discovery of new therapeutic ways.

Funded by: Université du Littoral Côte d'Opale

**37. Characterization of osteoblast-secreted vesicles: a new mode of intercellular communication**

J. Morhayim, R. Burggraaf, J.A. Demmers, M. Buitenhuis, M. van Driel, J. van de Peppel and J. van Leeuwen

Department of Internal Medicine; Department of Pathology; Proteomics Center, Department of Hematology, Erasmus MC, Rotterdam, The Netherlands

Email: j.morhayim@erasmusmc.nl

Osteoblasts are in close contact with neighboring cells in their bone marrow microenvironment, and proper communication systems are crucial for the regulation of healthy bone turnover as well as pathogenesis. Recently, a novel mode of communication via extracellular vesicles has attracted much attention in the scientific community. During bone formation, osteoblasts secrete matrix vesicles (MVs) involved in the mineralization of the extracellular matrix; however, information about other secreted vesicles is still lacking. In this study, we focus on the characterization of human osteoblast-secreted vesicles and investigate their potential as mediators of communication with bone marrow cells and other cells in the vicinity, e.g., metastatic cancer cells. We used a human preosteoblast-based in vitro bone formation model (svHFOs) to isolate vesicles by ultracentrifugation at various timepoints during osteoblast differentiation. We characterized the vesicles by transmission electron microscopy (TEM) and mass spectrometry-based proteomics and studied their interaction with other cells by fluorescent labeling and FACS analysis. Our TEM images showed structural heterogeneity among osteoblast-secreted vesicles, and proteomics analyses showed that the vesicles contained proteins not primarily linked to mineralization, suggesting the presence of vesicles other than MVs. Confocal analyses of fluorescently labeled vesicles showed that osteoblast-secreted vesicles were taken up by HEK 293 cells, and most interestingly by prostate cancer cells and hematopoietic stem cells, in a dose-dependent manner, proposing a novel mechanism of intercellular communication.

Funded by: Erasmus stem cell institute for regenerative medicine

#### 4. Inflammation and Infection

**38. Reticulocyte-derived exosomes from malaria infections are involved in antigen presentation and modulation of immune responses**

Hernando A. del Portillo^1,2^, Ernesto Nakayasu^3^, Mireia Ferrer^1^, Igor C. Almeida^3^ and Lorena Martin-Jaular^1^

^1^Barcelona Centre for International Health Research, Barcelona, Spain; ^2^Institució Catalana de Recerca i Estudis Avançats (ICREA), Barcelona, Spain; ^3^The Border Biomedical Research Center, University of Texas at El Paso, El Paso, TX, USA

Email: hernandoa.delportillo@cresib.cat

Reticulocyte-derived exosomes have a unique role in the removal of proteins during the maturation of reticulocytes to erythrocytes. Noticeably, there are malaria parasites with a unique tropism for reticulocytes, including *Plasmodium vivax*, the most widely distributed human malaria parasite. Here, we describe the isolation and characterization of exosomes from peripheral blood of human and mice infected with reticulocyte-prone malaria parasites. Importantly, proteomic analysis revealed the presence of parasite proteins in these vesicles. Moreover, immunization of mice with purified exosomes from peripheral blood elicited IgG antibodies capable of recognizing malaria-infected red blood cells. Furthermore, lethal challenge of immunized mice caused attenuation in the course of parasitemia and increased survival time. These results were obtained also when exosomes were isolated from in vitro cultures of infected reticulocytes, demonstrating that reticulocyte-derived exosomes are involved in antigen presentation and immune modulation in reticulocyte-prone malaria infections. Moreover, inclusion of CpG ODN 1826 in exosome immunizations elicited IgG2a and IgG2b antibodies and promoted survival and subsequent sterile protection of 83% of the animals lethally challenged. Present experiments are being conducted to determine the proteomics composition of reticulocyte-derived exosomes from human patients and to compare the adjuvanticity of flagellin vs. CpG. To our knowledge, this is the first report of immune responses elicited by exosomes derived from reticulocytes, opening new avenues for the exploration of these non-inflammatory nanovesicles in modulation of immune responses.

Funded by: Ministry of Science and Innovation, Spain; EviMalaR and Fundació Cellex

**39. Microvesicles of Dictyostelium discoideum as a model of eukaryotic extracellular vesicles**

Irène Tatischeff^1^, Sergei Kruglik^1^, Eric Larquet^2^, Monique Cheron^1^ and François Treussart^3^

^1^AnBioPhi, UPMC, Paris, France; ^2^LEBS, UPR 3082 CNRS, Gif-sur-Yvette, France; ^3^LPQM, CNRS UMR 8537, ENS Cachan, Paris, France

Email: irene.tatischeff@upmc.fr

We found that the secretion of extracellular vesicles is a constitutive process of *Dictyostelium discoideum* cells, occurring both during cell growth and early development. Moreover, this process was found to be associated to a detoxification mechanism of different structurally-unrelated drugs. The secreted vesicles, acting as “Trojan horses,” are capable of both transporting the drug and transferring it into human cells. This is why *Dictyostelium* microvesicles have been suggested as a new biological drug delivery tool (1). Constitutive *Dictyostelium* microvesicles secreted during starvation-induced development do also play a role in intercellular communication, by inhibiting normal cell aggregation and inducing an apoptotic process. Considering that *Dictyostelium* cells are much easier to manipulate than human cells, we suggest that they can be used to unravel the biological functions of extracellular vesicles. However, a better knowledge of these vesicles is an important prerequisite. Therefore, we studied *Dictyostelium* extracellular vesicles by cryoelectron microscopy, near-infrared Raman and spectrofluorimetry. We also report tentative endogeneous cell labeling of *Dictyostelium* extracellular vesicles by using fluorescent nanoparticles or a lipid-specific dye.

**Reference**

1. Tatischeff et al. Patent European priority No. 03 291 752 07/15/2003 (DRITT-UPMC) European Patent (Danemark, Deutschland, France, Great Britain, Italy, Netherland and Spain), US Patent and Pending Canadian Patent.

**40. Interaction of Trypanosoma cruzi infective forms and monocytes produces microvesicles that increase parasite infection**

Poliana Deolindo and Marcel I. Ramirez

Molecular Biology of parasites and vectors, Laboratory Instituto Oswaldo Cruz Fiocruz Manguinhos, Brasil

Email: p_deolindo@yahoo.com.br

*Trypanosoma cruzi*, the causative agent of Chagas disease, has a complex life cycle and presents several mechanisms to subvert vertebrate host recognition and clearance. We have seen that, when in contact with monocytes, metacyclic trypomastigotes (infective form from the insect vector) and cell derived trypomastigotes (infective form in vertebrate host) induce higher plasma membrane vesicles (PMVs) formation than insect-derived epimastigotes (non-infective form in insect gut). Proteomic analysis of purified PMVs has shown that both the membrane of the parasite and the monocyte are involved in the formation of these structures. A higher percentage of exposed phosphatidylserine (59%) is observed in PMVs produced during monocytes and cell-derived trypomastigote interaction. Further analysis using NBD-PE fluorescent lipids showed that PMVs from monocytes and all stages of parasite can fuse, resulting in a vesicle with membranes from both cells. However, these PMVs have different role in parasite infectivity. In vitro transwell assays showed that PMVs produced exclusively during the interaction of the infective forms and monocyte in the upper chamber increased significantly (∼90%) the infectivity of parasites into Vero cells in the lower chamber. The same was not observed with non-infective parasite stage. Taken together, these findings present a novel mechanism for *T. cruzi* infection based on the PMV formation as a result of the interaction between infective parasite and monocyte. We are interested in dissecting the PMVs pathway in infection as well as understanding the signals in the communication between parasite and host cell.

Funded by: Fiocruz, CNPq, CAPES

**41. Microvesicles secreted by Trypanosoma cruzi are linked to small RNA machinery and function as a system of intercellular communication and life cycle regulation**

M.R. Garcia Silva^1^, J. Sanguinetti^1^, F. Cabrera-Cabrera^1^, R. Neves^2^, T. Souto-Padron^2^, W. De Souza^2^, A. Parodi^3^, C. Rovira^4^, C. Robello^1^ and A. Cayota^1,5^

^1^Functional Genomics, Institut Pasteur de Montevideo, Montevideo, Uruguay; ^2^Laboratory de Biologia Celular e Ultra-estrutura Instituto de Microbiologia Prof. Paulo de Góes Universidade Federal do Rio de Janeiro, Brazil; ^3^Genetics, Faculty of Sciences, Universidad de la Republica, Montevideo, Uruguay; ^4^Oncology, Lund University, Lund, Sweden; ^5^Medicine, Faculty of Medicine, Montevideo, Montevideo, Uruguay

Email: rgarcia@pasteur.edu.uy

The key components of the sRNA machinery, mainly the Argonaute proteins bound with different miRNAs, have been found by some authors associated with the formation of multivesicular bodies related with the endosomal trafficking. Moreover, these studies revealed that the sRNAs transferred between cells are functional and are able to induce target inhibition of protein expression in the acceptor cells. These findings exposed a novel mechanism of targeted intercellular gene expression regulation between cells. This could have relevance in unicellular parasites of human health care as *Trypanosoma cruzi* (causative agent of Chagas's disease) because the molecules involved in the communication process between parasites are still poorly known. It has been taken for granted that communication between parasites to parasites and to its host cells exists. However, it has not been shown at cellular level the presence of the components responsible for these phenomena. What it is extensively demonstrated is that the success of the infectious process is based on molecular “crosstalk” with the host's immune cells in trypanosomes. Today, in the era of microvesicles, they are the cell factor candidates that could be responsible for these phenomena but that have still not been well studied. It is not known the content that these microvesicles secreted by *T. cruzi* shed to the medium or to their host environment. Therefore, the main purpose of this study was to know the proteome and small transcriptome of the secretome contained into microvesicles in this parasite. Results from high throughput sequencing of the sRNAs population contained into these microvesicles secreted by *T. cruzi* (data not published) exposed a large quantity of sRNAs compared with the intracellular sRNAs reported recently by our group. In this previous work, we surprisingly described that, among these sRNAs highlight the presence of 25% of tRNA-derived sRNAs (mini-tRNAs), these tRNA halves were recruited to particular cytoplasmic granules in *T. cruzi* epimastigotes into no previously reported organelles (1). Into the microvesicles secreted by epimastigotes of *T. cruzi*, these mini-tRNAs make up 56% of the sRNAs between 25 and 35 bp. These mini-tRNAs have been found in all the organisms where they were searched and seem to be a new group of sRNAs. With respect to other components related with the sRNA machinery, in *T. cruzi*, our group have recently demonstrated the presence of a cytoplasmic Argonaute protein named TcPIWI-tryp that could represent a distinctive subfamily for trypanosomatids and which functional role is still unknown (Garcia Silva, Tosar et al.). This TcPIWI-tryp was also found in the content of the microvesicles released to the medium. Proteome analysis of the microvesicles content revealed a great number of proteins with RNA or DNA binding domains, some proteins related to metabolic pathways and others derived from membrane composition. By the above, at this time, we aimed to reveal some functional role for these microvesicles. We demonstrated the exchange of microvesicles between parasites and its host cells. We reported here that mini-tRNAs contained into microvesicles released to the extracellular environment are internalized not only by neighboring parasites but also by mammalian host cells. Furthermore, the incubation of parasites with these microvesicles evidenced that the content of these secreted microvesicles could induce the transformation to metacyclic forms of the parasite. Interestingly, although *T. cruzi*-derived microvesicles were not fused with the non-susceptible K562 cells, the experimental fusion by electroporation rendered these cells susceptible to *T. cruzi* infection. Thus, microvesicles were not only associated with life cycle transition of epimastigotes toward the infective trypomastigote forms but also associated to infection susceptibility of resistant mammalian cells. However, it is still unknown the processes behind the phenomena related with the formation and function of these microvesicles that could be so significant for the parasite biology. This study brings out several unexpected features of the possible mechanisms of communication between parasites and to its host cells and opens novel perspectives concerning the strategies for exchange of genetic and protein elements contained into microvesicles in *T. cruzi*. In addition, concordant lines of evidence support the original hypothesis of the involvement of microvesicles in the survival strategy allowing trypanosomatids to exchange proteins at least between parasites and/or to manipulate the host immune system. We focus on shedding light on the secretion mechanisms involved in the production of the *T. cruzi* microvesicles content. Understanding the complexity of the *T. cruzi* secretome is a key component to elucidating the mechanisms used by these parasites for some kind of intercellular communication for example during its life cycle. A comprehensive understanding of the secretome of these parasites could provide novel targets for rational drug design or a source of antigens for vaccine development.

Funded by: ANII

**References**

1. Garcia-Silva MR, Frugier M, Tosar JP, et al. A population of tRNA-derived small RNAs is actively produced in *Trypanosoma cruzi* and recruited to specific cytoplasmic granules. Mol Biochem Parasitol. 2010;171(2):64–73.

2. Garcia Silva MR, Tosar JP, Frugier M, et al. Cloning, characterization and subcellular localization of a *Trypanosoma cruzi* argonaute protein defining a new subfamily distinctive of trypanosomatids. Gene 2010;466(1–2):26–35.

**42. The intracellular parasite, Trypanosoma cruzi, utilizes microvesicle release to invade host cells**

Jameel M. Inal^1^, Ephraim A. Ansa-Addo^1^, Igor Cestari^2,3^, Paras Pathak^1,4^, Maria V. McCrossan^5^, Sigrun Lange^6^ and Marcel I. Ramirez^2^

^1^Cellular and Molecular Immunology Research Centre, School of Human Sciences, Faculty of Life Sciences, London Metropolitan University, London, United Kingdom; ^2^Instituto Oswaldo Cruz - Fiocruz, Laboratório de Biologia Molecular de Parasitas e Vetores, Rio de Janeiro, Brazil; ^3^Seattle Biomedical Research Institute, Seattle, WA, USA; ^4^Medical Research Council Harwell, Harwell Science and Innovation Campus, Oxfordshire, United Kingdom; ^5^London School of Hygiene and Tropical Medicine, London, United Kingdom; ^6^University College London, Perinatal Brain Repair Group, London,United Kingdom

Email: j.inal@londonmet.ac.uk

Host microvesicles (MVs) help pathogens, such as the intracellular parasite, *Trypanosoma cruzi*, to evade complement attack. We have now found that the infectious metacyclic trypomastigote forms, by interacting with host integrins, lipid rafts and stretch activated channels, stimulate a calcium-mediated depolymerization of the actin cytoskeleton and MV release. The release of MVs in turn stimulates a lysosomal repair mechanism in the host cell, to plug the breach in the plasma membrane. By using specific inhibitors of lysosomal exocytosis and both pharmacological and siRNA-mediated inhibition of microvesicle release, we describe a novel entry mechanism by which the parasite opportunistically takes advantage of a host membrane repair mechanism, to execute entry before membrane integrity is fully restored.

Funded by: The Royal Society (London) to JI Brazilian Ministry of Education to IC

**43. Toxoplasma gondii: qualified to secrete exosomes?**

Marbel Torres, Céline Ducournau and Isabelle Dimier-Poisson

UMR 1282 ISP Université-INRA Immunologie Parasitaire et Vaccinologie, Biothérapies Anti-Infectieuses, Université François Rabelais, UFR de Pharmacie, Tours, France

Email: dimier@univ-tours.fr

*Toxoplasma gondii* is a protozoan parasite responsible for toxoplasmosis, a worldwide disease that leads to encephalitis in immune-compromised individuals and to congenital toxoplasmosis of infected fetus. In order to design effective vaccine strategy a more comprehensive understanding of the biology of *Toxoplasma*-host interactions is crucial. *T. gondii*, as eukaryotic cell, contains an endomembrane network including an endoplasmic reticulum and a single Golgi stack. A Rab5 protein-like, localized to tubovesicular structures adjacent to but distinct from the Golgi, is involved in the parasite cholesterol pathway. *T. gondii* apicoplast expresses Rab7 late endosomal marker. Moreover, *Toxoplasma rhoptries*, secretory organelles essential for cell invasion, present certain features of lysosomal secretory pathway. These data suggest that, among all these parasite secretory pathways, one could be dedicated to exosomal secretion. Using TEM, we observed *Toxoplasma* secretory organelles and detected MVB-like structure containing 65-nm vesicles confirming this hypothesis. We purified by sucrose cushion vesicles from peritoneal fluid of *Toxoplasma*-infected mice, which displayed biochemical and morphological characteristics of exosomes. Immunostaining with colloidal gold indicated the presence of *Toxoplasma*-specific proteins and proteomic analysis revealed the presence of exosomal markers (Tsp7, Tsp18, CD82, Rab5 and Rab11) and specific *Toxoplasma* proteins (SAG, MIC, GRA, GPI, ubiquitin and cyclophilin). These results suggest a novel exosome-based pathway as a mechanism of protein secretion used by *T. gondii*.

Funded by: University of Tours and INRA

**44. Exosomes in the interaction host-parasitic trematodes**

Antonio Marcilla^1^, María Trelis^1^, Alba Cortés^1^, Javier Sotillo^1^, M. Luz Valero^2^, Manuel M. Sánchez del Pino^3^, Carla Muñoz-Antoli^1^, J. Guillermo Esteban^2^, Rafael Toledo^2^ and Dolores Bernal^3^

^1^Área de Parasitología, Departamento de Biología Celular y Parasitología, Universitat de València, Burjassot, Valencia, Spain; ^2^Servicio de Proteómica, Centro de Investigación “Príncipe Felipe,” Valencia, Spain; ^3^Departamento de Bioquímica y Biología Molecular, Universitat de València, Burjassot, Valencia, Spain

Email: antonio.marcilla@uv.es

The study of host-parasite interactions has increased considerably in the last decades, with many studies focusing on the identification of parasite molecules [i.e., surface or excretory/secretory proteins (ESP)] as potential targets for new specific treatments and/or diagnostic tools. In parallel, in the last few years, there have been significant advances in the field of exosome research. These vesicles carry several atypical secreted proteins in different organisms, including parasitic protozoa. Here, we present experimental evidence for the existence of exosomes in parasitic helminthes, specifically the trematodes *Echinostoma caproni* and *Fasciola hepatica*. These microvesicles are actively released by the parasites and penetrate into the host cells. Trematode exosomes contain most of the proteins previously identified as components of ESP, as confirmed by proteomic and electron microscopy studies. In addition to parasitic proteins, we also identify host proteins in these structures. The existence of exosomes explains the secretion of atypical proteins along with host proteins in trematodes, and the demonstration of their uptake by host cells suggests an important role for these structures in host-parasite communication, as described for other infectious agents.

Funded by: FIS09/02355 and SAF2010-16236 from Ministerio de Ciencia e Innovación and FEDER; PROMETEO/2009/081 from Conselleria d'Educació, Generalitat Valenciana (Valencia, Spain), and UV-AE-10- 23739 from the Universitat de València (Valencia, Spain).

**46. Characterization of the exosome-associated prion protein in hamster plasma**

F. Properzi^1^, M.A. Logozzi^2^, H. Abdel-Haq^1^, I. Cristofaro^1^, T. Azzarito^2^, F. Cardone^1^, C. Federici^2^, L. Lugini^2^, S. Fais^2^; M. Pocchiari^1^

^1^Department of Cell Biology and Neuroscience and ^2^Department of Therapeutic Research and Medicines Evaluation, Istituto Superiore della Sanità (National Institute of Health), Rome, Italy

Email: francesca.properzi@iss.it

In recent years, a number of publications have shown that both the cellular and misfolded prion proteins (PrP) are secreted in association with exosomes in vitro. These exosome preparations are in some cases infectious and capable of propagating prions by inducing de novo formation of scrapie in healthy cells and animals. Despite these findings suggest that exosomes could be involved in prion propagation and partially confirm the “Troyan exosome hypothesis” of Gould et al. (1), there is still no evidence that disease-associated PrP is carried by microvesicles in vivo. To date, in vivo studies have only confirmed the presence of cellular PrP (PrPC) on exosomes of ovine cerebrospinal fluid. The identification of PrP in main body fluid systems remains elusive and controversial. Here, we show that PrPC is unequivocally associated with hamster blood exosomes in vivo. Western blot analysis of purified plasma exosome preparation revealed the presence of aggregates of high molecular weight. They were detected on plasma exosomes but not in vesicles derived from other sources such as cell-conditioned medium. Interestingly, the use of stringent conditions progressively disaggregates large molecular weight bands while increases the intensity of monomeric PrP bands. Immunoprecipitation showed that these aggregates include exosomal markers. These results suggest that PrP in plasma is associated with exosomes, but other proteins could mask PrP, possibly also explaining why the detection of prions in body fluids is elusive.

Funded by: Istituto Superiore della Sanità, Rome, Italy

**Reference**

1. Gould SJ, Booth AM, Hildreth JE. Sci USA. 2003 Sep 16;100(19)

**47. Small RNA sequencing of neuronal exosomes identifies a distinct miRNA signature associated with prion disease**

Shayne A. Bellingham^1–3^, Bradley M. Coleman^1,2^, Belinda B. Guo^1,2^, Robyn A. Sharples^1,2^ and Andrew F. Hill^1–3^

^1^Department of Biochemistry and Molecular Biology, The University of Melbourne, Melbourne, Victoria, Australia; ^2^Bio21 Molecular Science and Biotechnology Institute, The University of Melbourne, Melbourne, Victoria, Australia; ^3^Mental Health Research Institute, Melbourne Brain Centre, The University of Melbourne, Melbourne, Victoria, Australia

Email: shayneb@unimelb.edu.au

Exosomes are small membranous vesicles, 50–130 nm, which have been shown to contain mRNA and microRNA (miRNA), termed exosomal RNA. Significantly, miRNA profiles of circulating exosomes isolated from peripheral blood, serum and saliva have been generated and suggest they have diagnostic potential for human disease. Prion diseases are a family of rare progressive neurodegenerative disorders that affect both humans and animals. These diseases are associated with the conversion of the host-encoded cellular prion protein (PrPC) into an abnormal pathogenic isoform (PrPSc) by a template-directed mechanism. Previously, we have shown that both PrPC and PrPSc are released in association with exosomes from neuronal cells and can transmit infection in vitro and in vivo. Here, utilizing our prion disease cell models, we performed the first small RNA deep sequencing study of neuronal exosomes and demonstrate that exosomes contain a diverse range of RNA species including retroviral RNA repeat regions, mRNA fragments, tRNA fragments, non-coding RNA, small nuclear RNA, small nucleolar RNA, small cytoplasmic RNA, silencing RNA as well as known and novel candidate miRNA. Significantly, we show that exosomes released by prion-infected neuronal cells have increased miR-128a, miR-21, miR-222 and miR-29b levels with decreased miR-146a levels compared to non-infected exosomes. Overall, these results demonstrate that circulating exosomes released during prion infection have a distinct miRNA signature that can be utilized for diagnosis and understanding mechanisms in prion disease.

Funded by: National Health Medical Research Council of Australia (Early Career Fellowship to S.A.B., Dora Lush Biomedical Postgraduate Scholarship to B.M.C.); Australian Research Council Future Fellowship to A.F.H; and The University of Melbourne Early Career Grant Scheme to S.A.B.

## Scientific Program 2012 ISEV meeting Thursday 19th April

### Symposium Session 4          8.30-10.00

#### 1. Characterization of Extracellular Vesicles by High Resolution Microscopy and Flowcytometry

**Advances in nanoscale superresolution microscopy – relevance for extracellular vesicles research**.

Hjalmar Brismar

(no abstract)

**The atomic force microscopy a suitable technique to characterize microvesicles in the nanometer scale**

Julie Hardij, Francesca Cecchet, François Mullier, Christian Chatelain, Bernard Chatelain and Jean-Michel Dogné

Namur Research Institute for Life Sciences (NARILIS), Department of Pharmacy, University of Namur, FUNDP; Laboratory of Lasers and Spectroscopies (LLS) Research Centre in Physics of Matter and Radiation, FUNDP; Namur Research Institute for Life Sciences (NARILIS), Hematology Laboratory-NTHC, CHU Mont-Godinne, UCL Mont-Godinne

Email: julie.hardij@fundp.ac.be

*Introduction:* Patients suffering from cardiovascular disease or cancers present a high risk of developing thrombotic events. An observation associated with the high incidence of thrombosis is an elevated number of circulating microparticles. Microparticles are small vesicles shed from potentially all cell types and may be involved in the prothrombotic risk due to their expression of procoagulant proteins such as the tissue factor (TF). Currently, no technique allows determining the size and expression analysis of these MVs in the nanometer scale. Atomic force microscopy (AFM) was first reported in 2010 to characterize plasma MVs. This technique provides topographic information of a sample coated on a functionalized substrate. Three major observations are brought with AFM: a number of particles, an estimation of their volume and a selection of them regarding to an antigenic expression. The aim of this study is to validate the use of AFM for the specific characterization of TFMVs. Breast cancer cells MDA-MB231 known to produce TF-MVs will be used as model in our study. *Material and methods:* The measurements were performed using AFM nanoscope III (Veeco) in air-tapping mode. (1) Micas sheets substrate was activated with ethanolamine and glutaraldehyde. Substrate was washed and incubated with 25 µl of 10 µg/µl antibody (anti CD142 = TF or anti CD41 = negative control) for 30 minutes. Twenty-five microliters of the microvesicle suspension was added for 1 hour. Substrate is softly washed again and blow-dried with nitrogen gas for the observation. (2) Twelve million of MDA-MB231 breast cancer cells were suspended in 1 mL PBS and placed at 37°C for 45 minutes. Cells were eliminated with a 5-minute centrifugation at 200g. PBS vehicle was also tested as negative controls. *Results:* From AFM images, the number of CD142 antibodies on the surface was estimated to approximately 2200/µm^2^, which is 100-fold larger than obtained by Yuana et al. (1). The molecular diameter was about 20±4 nm, this being the limiting size below which MVs cannot be distinguished from CD142 antibodies. The mean size of MVs released by MDA-MB231 cells, as estimated from 1200 MVs, was 38±9 nm [range: >20 to 60 nm]. The TF-MVs concentration produced by 12×106 MDA-MB231/ml was between 170×10^9^ and 170×10^10^ MVs/ml. The vehicle showed no coated particles on 10×10 µm^2^ images. Cancer cells-derived MVs did not adsorb on CD41 antibodies attesting the specificity of the coating. *Conclusion and prospects:* AFM is suitable to analyze a microvesicle size distribution based on a specific TF expression. These measures were achieved in air condition and should also be tested in a liquid background in order to get conditions closer to native ones. Plasma samples TF-MVs from well-defined cancer patients could be tested and compared to healthy plasma donors, to identify patients with high levels of TF-MVs. These observations should be compared with procoagulant activity measured by thrombin generation assay, to identify patients at high risk of thrombosis.

Funded by: FRIA research

**Reference**

1. Yuana Y, et al. J Thromb Haemost. 2010 Feb;8(2):315–23.

**Label-free identification of extracellular vesicles by Raman microspectroscopy**

E. van der Pol, C.M. Hau, C. Otto, A.T.M. Lenferink, A. Sturk, T.G. van Leeuwen, and R. Nieuwland

Laboratory Experimental Clinical Chemistry; Biomedical engineering and Physics, Academic Medical Center, Amsterdam, The Netherlands; Medical Cell BioPhysics, University of Twente, Enschede, The Netherlands

Email: e.vanderpol@amc.uva.nl

*Background*: The cellular origin of extracellular vesicles is usually established by fluorescent antibody labeling, which is laborious, is expensive and involves practical problems. Using an ultramodern Raman microspectroscopy setup, we have identified extracellular vesicles without fluorescent antibody labeling. *Methods*: Platelet and erythrocyte vesicles were isolated from blood bank concentrates. In addition, tumor-derived vesicles were isolated from BXPC3 and SIHA cell lines. Vesicles were isolated using differential centrifugation and analyzed by flow cytometry, transmission electron microscopy, Raman microspectroscopy and resistive pulse sensing. For Raman microspectroscopy, a 100-mW krypton ion laser operating at a wavelength of 647 nm was focused to a probe volume of 0.3 µm^3^. The Stokes shift from light scattered by vesicles was measured using a spectrograph dispersing in the range 646–849 nm. Resistive Pulse Sensing is a novel method capable of measuring the diameter, concentration and surface charge of single vesicles in suspension using a nanopore. *Results*: The Raman spectrum of vesicles with a different cellular origin is assessed. Platelet vesicles have a Raman spectrum containing spectral transitions characteristic of phospholipids. However, the Raman spectrum of, for instance, erythrocyte vesicles is entirely different. *Conclusions*: For the first time, vesicles are identified without fluorescent antibody labeling.

Funded by: Academic Medical Center

**High resolution flow cytometry-based multiparameter analysis of nanosized cell-derived vesicles**

Marca H.M. Wauben, Els J. van der Vlist, Willem Stoorvogel, Ger J.A. Arkesteijn and Esther N.M. Nolte-‘t Hoen

Department of Biochemistry & Cell Biology and Department of Immunology, Faculty of Veterinary Medicine, Utrecht University, Utrecht, The Netherlands

Email: m.h.m.wauben@uu.nl

The total population of extracellular vesicles in body fluids or produced during in vitro cell culture is heterogeneous and the majority of these vesicles is in the nanosized range (<300nm). To yield information about the (sub)cellular origin, molecular composition and potential function of these vesicles, highly sensitive high-throughput multiparameter techniques are needed to analyze vesicles at the single particle level. Recently, we developed a high resolution fluorescence-based flow cytometric method enabling integrated analysis of multiple parameters such as light scattering, quantity, buoyant density and surface markers (based on antibody binding) of individual nanosized vesicles and particles. We found that biological vesicles, such as DC-derived exosomes and 100 nm virions, induced forward light scattering (FSC) largely comparable to polystyrene beads with the same size, while artificial membrane vesicles with a low relative refractive index, such as empty liposomes, were much less comparable to beads. Thus, this method can be used for relative sizing of cell-derived vesicles. By combining quantitative analysis, FSC signals and specific protein staining, we were able to perform subset analysis of extracellular vesicles, which opens novel possibilities for basic research, biomarker profiling and quality control of cell-derived vesicles used as therapeutic agents.

Funded by: Utrecht University

**Characterization of extracellular vesicles by multiparameter nanoparticle tracking analysis**

C. Gardiner, R.A. Dragovic, D.S. Tannetta, P. Harrison, C.W.G. Redman and I.L. Sargent

Nuffield Department of Obstetrics and Gynaecology, University of Oxford, United Kingdom

Email: chris.gardiner@obs-gyn.ox.ac.uk

The study of extracellular vesicles (EV) has been hampered, to date, by their small size as they are unresolvable by light microscopy and most are below the limit of resolution of conventional flow cytometry. We have applied nanoparticle tracking analysis (NTA) to overcome many of the limitations of existing techniques for vesicle analysis. Sizing, verified by NIST standards, is accurate across the size range of EVs (50—1000 nm). Through the use of low refractive index reference silica microspheres, it is possible to achieve more accurate estimates of vesicle concentration. Our data show that many exosomes are larger than previously reported with modal sizes ranging from 80 to 150 nm depending on the parent cell type. Previously sizing was performed by electron microscopy, which requires dehydration of the sample resulting in shrinkage. NTA requires no sample preparation and agrees with size data obtained by cryo-EM. Analysis of blood plasma has revealed that there are many times more EVs in blood than previously reported with typical values of 1–10×10^10^/mL. NTA immunophenotyping may be performed using quantum dot labeled antibodies or antibodies labeled with conventional fluorophores under constant flow to reduce photobleaching. While specific labeling of very small vesicles may be problematic due to low antigen numbers, limited availability of suitable avid antibodies with sufficiently bright fluorophores and difficulty in resolving them from unbound label, all vesicles can be labeled using membrane specific dyes (e.g., Cell Mask Orange). These can be used to distinguish EV from lipoprotein vesicles in plasma. We have demonstrated nucleic acids in vesicles released from several tumour cell lines, which were not seen in the majority of those isolated from the blood or urine of normal healthy donors. The recent addition of zeta potential measurement to NTA has allowed us to study the surface charge of EV from a variety of cell types and biological fluids. All EVs measured to date have had zeta potentials of −20 to −80 mV but there are cell-specific differences. We conclude that multiparametric NTA is a powerful tool for the study of EVs.

Funded by: Wellcome Trust Technology Development Grant (ref GR087730), Wellcome Trust Programme Grant (ref GR079862MA) and by the Oxford Partnership Comprehensive Biomedical Research Centre with funding from the Department of Health's NIHR Biomedical Research Centres funding scheme

#### 2. Diagnostic and Treatment at Malignancy

**Exosomes from neuroblastoma-differentiated cells enclose an integrated and transmissible acetylcholine signaling**

I. Parolini^1^, F. Manfredi^1^, C. Coscia^1^, S. Camerini^2^, C. Zanetti^1^, S. Cardarelli^3^, G. Poiana^3^ and M. Sargiacomo^1^

^1^Department of Hematology Oncology and Molecular Medicine, Istituto Superiore di Sanità, Rome, Italy; ^2^Department of Cell Biology and Neuroscience, Istituto Superiore di Sanità, Rome, Italy; ^3^Department of Biology and Biotechnology, “Charles Darwin” Sapienza University, Rome, Italy

Email: isabella.parolini@iss.it

In the last few years, it has been reported that both tumoral and normal nervous cells release exosomes in extracellular medium, whose functions are still poorly understood. We studied a possible role for exosomes in neuronal differentiation using N18TG2 parental neuroblastoma cells and a clone of N18TG2 transfected with cDNA encoding choline acetyltransferase (2/4 ChAT). This transfection activates acetylcholine synthesis and stimulates progression of cells in their differentiation program. Acetylcholinesterase (AChE), a protein related to the progression of neuronal development, has been previously indicated as an exosomal marker, but AChE-containing exosomes have not been studied in neuronal differentiation. Exosomes isolated from N18TG2 parental and 2/4 ChAT-transfected cells were tested for the presence of AChE and acetylcholine. We found both molecules enriched in 2/4 cells-derived exosomes. N18TG2 cells treated with 2/4 derived exosomes showed an increased proliferation, adhesion and neurite outgrowth. Proteomic analysis by mass spectrometry performed on exosomes from 2/4 ChAT-transfected and parental cells indicated a different protein profile of the two populations. In particular, a selective expression of proteins involved in neuronal development, like collapsin response mediator protein 1 (CRMP), was found in exosomes from ChAT-trasfected cells. In conclusion, exosomes from differentiated ChAT-transfected cells contain signals that may drive neuronal developmental progression.

Funded by: Italian Ministry of Health

**Exosomal miRNA signature as potential biomarker for the early detection of non–small-cell lung cancer**

D. Andrējeva, E. Zandberga, A. Ābols, U. Kopeika, G. Purkalne, P. Zajakins and A. Linē

Latvian Biomedical Research and Study Centre, Riga, Latvia; University of Latvia, Riga, Latvia; Pauls Stradins Clinical University Hospital, Riga, Latvia

Email: Diana.Andrejeva@lbmc.lv

Non–small-cell lung cancer (NSCLC) is a rapidly growing cancer for which diagnosis is usually delayed, and therefore it is the main cause for cancer-related death in worldwide. MiRNA expression profile is altered in many cancers, and there is evidence that, in cancer, overexpressed miRNAs might be used as serological biomarkers for the early detection of cancer. However, our previous study of miRNA profiles in thyroid tumour tissues and sera suggested that the miRNA profile in tissues does not match to that in the serum. In the present study, we used a new approach based on the profiling of exosomal miRNA derived from the culture medium of primary lung cancer cells and PBMC. The comparison between the lung cancer cell and the PBMC exosomal miRNA profile revealed 16 differentially expressed (log2 > 1–2-fold change) miRNAs, 10 of them were upregulated (miR-1246, miR-1290) and 6 miRNAs where downregulated (miR-382, miR-1197) in lung cancer exosomes. In literature is shown that miR-1246 might be responsible for apoptosis induction in response to DNA damage and that this miRNA is released by malignant mammary epithelial cells, but not non-malignant. These data suggest that miR-1246 and others could be used as lung cancer serological biomarkers, but testing with qRT-PCR in larger patients’ cohorts is needed.

Funded by: European Social Fund (ESF); Latvian Council of Sciences collaborative project (No. 10.0010.03); European Regional Development Fund (ERDF).

**RNA profile of exosomes from the isolated hepatic perfusion of uveal melanoma patients with liver metastases**

Cecilia Lässer, Maria Eldh, Serena O'Neil, Per Lindnér, Margareta Sjöstrand, Roger Olofsson, Joar Svanvik and Jan Lötvall

Krefting Research Centre, Department of Internal Medicine, University of Gothenburg, Gothenburg, Sweden; Transplant Centre, Sahlgrenska University Hospital, Gothenburg, Sweden

Email: cecilia.lasser@gu.se

*Background:* Uveal melanoma is a tumor arising from the melanocytes of the eye, which primarily metastasise to the liver. Patients with liver metastases from malignant melanoma have a poor 1-year survival rate, and systemic chemotherapy has only shown limited effects. Regional isolated perfusions have, therefore, been used to distribute a local high cytostatic drug concentration and have been shown to lead to tumor remission in more than 50% of the patients. The aim of this study was to determine the presence of exosomes, and their RNA content, from isolated hepatic perfusion from uveal melanoma patients with liver metastases. *Method/Results:* Exosomes from isolated hepatic perfusion were detected and analyzed for commonly used exosomal markers with electron microscopy (CD63) and flow cytometry (CD9, CD63 and CD81). Exosomal RNA isolated from three patients was detected using a Bioanalyzer. Furthermore, RNA from one patient was analysed with real-time PCR and shown to contain let-7b and miR-199a, miRNAs previously demonstrated to be upregulated in uveal melanoma. The RNA was further analyszd by using a RT2 miRNA PCR Array, and the 11 most prominent miRNA from the array were analyzed with Ingenuity Pathway Analysis. *Conclusion:* The pathway analysis showed that the miRNA from the liver perfusate exosomes regulates proteins associated with melanoma. This is an important finding, as it has been shown that tumor exosomes can prepare lymph nodes for metastasis. Furthermore, early diagnosis is important in the battle against cancer and there is a need for biomarkers. These miRNA will therefore be analyzed for their presence in plasma exosomes in these patients.

Funded by: The Swedish Research Council, the Swedish Heart and Lung Foundation, the Swedish Asthma- and Allergy Foundation, the Swedish Cancer Society and VBG GROUP'S Herman Krefting Foundation for Allergy and Asthma Research

**Exosomes from mutant KRAS cells transfer KRAS and transform wild-type KRAS recipient cells**

Jeffrey L. Franklin, Michelle Demory Beckler, James N. Higginbotham and Robert J. Coffey

Vanderbilt University Medical Center, Nashville, TN, USA

Email: jeff.franklin@vanderbilt.edu

Secreted vesicles in general have been shown to influence the cellular environment and, in the case of tumor cells, can transmit signals that promote tumorigenesis. We have determined that oncogenic mutations affect the composition and function of tumor-derived exosomes, influencing the level of signaling molecules present, like the EGF receptor ligand amphiregulin that provides a uniquely potent invasive signal (1). In order to understand how tumor cell exosomes affect their environment and how oncogenic mutations alter the output of tumor-derived exosomes, we have performed LC-MS-MS proteomic analysis of exosomes from colon cancer cells DLD-1 (with one mutant and one wild-type KRAS allele) and isogenically matched DKO-1 (with only a mutant KRAS allele) or DKS-8 cells (with only a wild-type KRAS allele). DKO-1 cells are transformed, growing in soft agar and in nude mouse xenografts, while DKS-8 cells do not. We have found that cells with mutant KRAS produce exosomes containing tumor-promoting proteins including mutant KRAS itself. Precise quantification by multiple reaction monitoring (MRM) has shown that mutant KRAS itself can be transferred to cells with only wild-type KRAS and purified DKO-1 cell-derived exosomes added to DKs-8 cells enhances their growth in 3D culture. We are now determining how these KRAS-containing exosomes function in in vivo tumor models, and we are characterizing patient serum exosomes using fluorescence-activated vesicle sorting (FAVS), a method that we developed to address whether their contents can be used to predict colon cancer progression.

Funded by: NCI CA46413, GI Special Program of Research Excellence P50 95103 and NCI 1R25CA92043 to MDB.Vanderbilt Ingram Cancer Center (P30 CA68485) and the Vanderbilt Digestive Disease Research Center (DK058404)

**Reference**

1. Higginbotham JN, et al. Curr Biol. 2011 May 10;21(9):779–86.

**HIV and cancer cells induce TNFalpha secretion by TACE and ADAM10-uploaded microvesicles**

Andreas Baur, Jung-Hyun Lee, Florian Dreyer, Sebastian Wittki, Tanja Bräu and Jochen Dindorf

Department of Dermatology, University Hospital of Erlangen, Germany

Email: andreas.baur@uk-erlangen.de

Recently, we and others have reported that HIV causes a massive secretion of microvesicles in tumor cell lines and in primary PBMC in vivo. While these vesicles contained high amounts of the viral Nef protein, their relevance for the viral life cycle was not evident. Likewise, tumor cells secrete large amounts of vesicles for reasons that are not clear. Here, we demonstrate that both cancer and HIV-infected cells shuttle activated TACE and ADAM10 into microvesicles in order to induce TNFa secretion by an autocrine and paracrine mechanism. We assume that ADAM-loaded microvesicles are induced by both diseases because (1) they have access to perinuclear compartments that are particularly rich in cytokine precursors and (2) they reach compartments in bystander cells. In both cell types, the generation of these vesicles is mediated by an integrin-dependent signaling pathway in which the polycomb protein Eed plays a pivotal role. Importantly, these in vitro characterized vesicles were also found in plasma of melanoma and HIV-infected patients. Our study implies that HIV and cancer cells exploit an integrin-activation pathway to generate ADAM-loaded vesicles for better proliferation/growth conditions in their microenvironment.

Funded by: The German Science Foundation (DFG): SFB 643 (K.K., J.D.), BA961/4-1 (J-H.L.), BA961/3-1 (S.W.) and GRK1071 (T.B.). F.D. is supported by the Hans Seidel Stiftung.

**Protein typing of circulating microvesicles allows real-time monitoring of glioblastoma therapy**

Huilin Shao^1,2^, Jaehoon Chung^1^, Leonora Balaj^3^, Alain Charest^4^, Darell D. Bigner^5^, Bob S. Carter^6^, Fred H. Hochberg^7^, Xandra O. Breakefield^3,8^, Ralph Weissleder^1,7,9^ and Hakho Lee^1^

^1^Center for Systems Biology, Massachusetts General Hospital, Boston, MA, USA; ^2^Harvard Biophysics Program, Harvard Medical School, Boston, MA, USA; ^3^Neuroscience Center, Department of Neurology, Massachusetts General Hospital, Charlestown Navy Yard, Boston, MA, USA; ^4^Molecular Oncology Research Institute, Tufts University School of Medicine, Boston, MA, USA; ^5^Brain Tumor Center, Department of Pathology, Duke University Medical Center, Durham, NC, USA; ^6^Division of Neurological Surgery, UCSD School of Medicine, San Diego, CA, USA; ^7^Massachusetts General Hospital Cancer Center, Boston, MA, USA; ^8^Program in Neuroscience, Harvard Medical School, Boston MA, USA; ^9^Department of Systems Biology, Harvard Medical School, Boston, MA, USA

Email: hshao@fas.harvard.edu

Glioblastomas shed large quantities of small, membrane-bound microvesicles (MVs) into the circulation. While these hold promise as potential biomarkers of therapeutic response, there remain hurdles to their identification and quantitation. Here, we describe a highly sensitive and rapid analytical technique for profiling circulating MVs directly from blood samples of glioblastoma patients. MVs, introduced onto a dedicated microfluidic chip, are labeled with target-specific magnetic nanoparticles and detected by a miniaturized nuclear magnetic resonance system. Compared with current standard assays (e.g., Western blotting, ELISA and flow cytometry), this integrated system has a much higher detection sensitivity and can differentiate glioblastoma multiforme (GBM) MVs from non-tumor host cell-derived MVs. The system further showed that circulating GBM MVs could serve as a surrogate for primary tumor by reflecting its molecular signature and a predictor of treatment-induced changes. We expect that this converging nanotechnology platform would have a wide range of applications, providing both an earlier indicator of drug efficacy and a potential molecular stratifier for human clinical trials.

Funded by: U54CA151884, R01EB010011, R01EB004626, P01CA069246, P50CA86355, U01CA141556, U24CA092782 and R21CA14122

#### 3. Proteomics and Biomarkers

**The future of advanced proteomics—implications for low-abundance proteins**.

Christian Scharf

Bremen, Germany

(no abstract)

**Multi-omics biomarker identification and characterization of human cell-derived extracellular vesicles**

Evelyn Orsó^1^, Markus Peer^1^, Stefan Wallner^1^, Annika Pienimaeki-Roemer^1^, Oliver Kenyon^2^, Alexander Sigruener^1^ and Gerd Schmitz^1^

^1^Institute for Laboratory Medicine and Transfusion Medicine, University Hospital Regensburg, Regensburg, Germany; ^2^Apogee Flow Systems Ltd., Hemel Hempstead, Hertfordshire, United Kingdom

Email: evelyn.orso@klinik.uni-regensburg.de

The booming extracellular vesicle (EV) research requires improved enabling technologies and appropriate biomarkers for EV-analysis. Three diverse human cell-derived EVs have been characterized in our group by multiomics analysis: (i) platelet-derived EVs (PL-EVs) and their five subsets, prepared by density gradient centrifugation, upon in vitro platelet senescence, (ii) EVs of circulating neutrophils, monocytes and in vitro monocyte-derived macrophages exposed to prototypic atherogenic lipoprotein loading, (iii) EVs of large vs. small subcutaneous and visceral adipocytes, isolated by nanomagnetic beads. The EVs were analyzed by different novel technologies, including resistive pulse sensing (IZON), nanoparticle tracking analysis (NanoSight), field-flow fractionation (Postnova Analytics) and advanced high sensitivity flow cytometry (Apogee). Biomarker search of isolated EVs was carried out by multiparameter flow cytometry, mass spectrometry-based lipidomics and proteomics as well as microarrays for micro-RNA (miRNA) expression. The lipid biomarker composition of these EVs resembles that of raft-microdomains, as they are enriched in free cholesterol, ceramide, phosphatidylserine and lysophosphatidic acid lipid species. They also enrich glycosyl-phosphatidylinositol-anchored molecules and diverse miRNAs.

Funded by: “The European Lipidomics Initiative; shaping the life sciences,” a specific support action subsidized by the EC 2006-2007 (proposal number 013032), the 7th framework program of the EU-funded “LipidomicNet” (Lipid droplets as dynamic organelles of fat deposition and release: translational research toward human disease; proposal number 202272) and SysMBio/BMBF (Systembiologie der metabolischen Phänotypen, TP 5: Lipidomics), proposal number 0315494C

**The proteome of nasal exosomes**

S.E. O'Neil, C. Lässer and J. Lötvall

Krefting Research Centre, Department of Internal Medicine, University of Gothenburg, Sweden

Email: serena.oneil@lungall.gu.se

Nasal lavage fluid (NLF) is a non-invasive sample commonly used for assessing mediators related to nasal diseases. We have recently established the presence of exosomes in the NLF (1). The aim of this study was to drill deep into the proteome of nasal exosomes using exclusion lists, which may provide clues as to the cellular origin and role of these exosomes in the nose. Exosomes were isolated from two pools of NLF, consisting of two healthy individuals each. The exosomal proteins were extracted and divided into 8 chromatographic fractions before being run on an Orbitrap Velos. Three consecutive exclusion lists were used to drill deeper into the proteome. Mass spectrometry analysis of the exosomal protein identified 382 proteins in pool A (87 µg) and 450 proteins in pool B (67 µg) in the first run (identified at FDR 1%, 1 peptide); 270 proteins were common to both pools (including ezrin and CD9), while 112 proteins were unique to pool A and 181 proteins were unique to pool B. Compared to the human exosomal proteins in Exocarta, 260 proteins were common, while 100 proteins have not been previously reported. Ingenuity pathways analysis revealed that the proteins were associated with several biological functions and pathways, including cell movement, immune cell trafficking and the clathrin-mediated endocytosis signalling pathway. This study has analyzed the baseline proteome of nasal exosomes under healthy conditions. Associated with normal host defense activities, this proteome may be altered under the influence of nasal diseases.

Funded by: Lars Hiertas Minne, Swedish Research Council

**Reference**

1. Lässer C, et al. Am J Rhinol Allergy. 2011 Mar-Apr;25(2):89–93.

**Characteristic and proteomic analysis of exosomes derived from ovarian cancer cell line**

Bing Liang^1^, Peng Peng^1^, Xialu Li^2^ and Shen Keng^1^

^1^Department of Obstetrics and Gynecology, Peking Union Medical College Hospital, Peking Union Medical College, Chinese Academy of Medical; ^2^National Institute of Life Science, Beijing, China

Email: liangbing0516@sina.com.cn/pengp1999@yahoo.com.cn

Ovarian cancer is the most lethal tumor among all frequent gynecologic malignancies, because of the most patients presenting with advanced disease at diagnosis. Exosomes are important intercellular communication vehicles, released by various cell types. Although recent studies have indicated that tumor-derived exosomes play important roles in tumor growth and invasion, the mechanisms of secretion and the biological roles of ovarian cancer-derived exosomes are not well understood. Here, we presented first the protein profile of highly purified exosomes derived from two ovarian cancer cell lines. Exosomes were prepared by classic method, including several steps of centrifugation and purified by sucrose/D2O cushion ultracentrifugation. Exosomal marker proteins were detected by Western blotting. The morphology of exosomes was analyzed by cryoelectron microscopy and Image J software. The density of exosomes was determined by sucrose density gradient centrifugation. The proteome of the highly purified exosomes was profiled by 1D SDS-PAGE electrophoresis and LC-MS/MS analysis. The MS results were also confirmed by Western blotting. We found that exosomal marker proteins were all detected in the exosomes derived from all the six ovarian cancer cell lines. Exosomes were found to be round and 30–200 nm in diameter. The range of density was between 1.10 and 1.19 g/ml. Total 2143 proteins were identified from OVCAR-3 cell-derived and IGR-OV1 cell-derived exosomes. Among them, 1017 proteins were identified in both two exosomes, including 318 proteins unreported as yet. We also found that 62 proteins associated with tumorigenesis and 18 proteins associated with angiogenesis. Taken together these results, we conclude that exosomes are generally released by ovarian cancer cells and provide a potential source of serum-based protein biomarkers. These data will be helpful to search for early diagnostic biomarkers of ovarian cancer, elucidate the roles of exosome in tumorigenesis and develop new therapeutic targets.

Funded by: Youth Fund of National Natural Science Fundation (Program grant: 81101976)

**Oncogenic Ras-induced epithelial-mesenchymal transition in MDCK cells alters proteome profiles of secreted exosomes**

Bow Tauro, Rommel Mathias, David Greening, Viet Phuong Anh Le, Hong Ji, Suresh Mathivanan, Hong Jian Zhu and Richard J. Simpson

La Trobe Institute for Molecular Science (LIMS), La Trobe University, Bundoora, Victoria, Australia

Email: b.tauro@latrobe.edu.au

Epithelial-mesenchymal transition (EMT), a process thought to underline cancer metastasis, involves polarized epithelial cells losing their basic morphological properties and gaining mesenchymal cell characteristics such as increased cell migration and invasion. We have previously shown that the extracellular microenvironment (secretome) is an important modulator of EMT in Ras-induced MDCK cells (21D1 cells). We hypothesize that exosomes are important regulators of EMT. In this study, exosome density (typically 1.08–1.22 g/mL) was exploited for isolating exosomes from MDCK and 21D1 cells using OptiPrep-based density gradient ultracentrifugation. Differential proteome profiling was determined by GeLC-MS/MS and peptide spectral counting. Of the total 457 proteins unambiguously identified (at least 2 tryptic peptides), 325 were common to both MDCK and 21D1 exosomes. Of these, 23 proteins were associated with exosome formation, intracellular membrane trafficking and exosome release, and proteins that may facilitate exosome uptake. Significant differences were observed between the exosome proteomes during EMT with 57 and 75 proteins unique to MDCK and 21D1 cells, respectively. Differentially expressed proteins were predominantly involved in extracellular matrix remodeling, cell-matrix interactions, transcript splicing and protein translation. We speculate that dysregulation of these exosomal proteins during EMT may confer functional significance in a recipient cell.

Funded by: National Health & Medical Research Council of Australia (program grant #487922) and a University of Melbourne Research Scholarship (B.J.T)

### Coffee and Poster Session 2          10.00-12.00

### Lunch and Poster Viewing          12.00-13.00

### Symposium Session 5          13.00-15.00

#### 1. Extracellular Vesicle RNA Content and Delivery

**Identification and characterization of exosome-encapsulated small RNA populations by deep sequencing: the foundation for a novel exosome-based cancer diagnostic platform**

Danijela Koppers-Lalic^1^, Michael Hackenberg^2^, Monique van Eijndhoven^1^, Daoud Sie^1^,Bauke Ijlstra^1^, Jaap Middeldorp^1^ and Michiel Pegtel^1^

^1^Department of Pathology, VU University Medical Center, The Netherlands; ^2^Computational Genomics and Bioinformatics Group, University of Granada, Spain

Email: d.koppers@vumc.nl

Cancer diagnosis through accessible and reliable molecular biomarkers is paramount for early detection of disease. The presence of stable, non-coding small RNA in tumor-derived exosomes circulating in biofluids opens unique possibilities for using this complex tumor-derived material as accessible biomarkers. Our aim is to combine experimental and computational analysis in constructing tumor-specific microRNA profiles in tumor tissues and exosomes from patient sera. To obtain comprehensive “inside-out” visibility of complete cancer microRNA signatures, we have initiated and recently completed massively parallel, high-throughput (deep) sequencing technique of cellular and exosomal small RNAome. The human oncogenic Epstein-Barr virus (EBV)-infected cells contain specific EBV-miRNA signatures and secrete large amounts of exosomes, thus representing a suitable model for our studies. Cell-secreted exosomes were isolated from different EBV-/+ lymphoma cell-lines by collecting growth-media and differential ultracentrifugation. Cellular and exosomal small RNAs were processed for deep-sequencing, and generated libraries were sequenced using the Illumina/Solexa platform. The first results indicate successful generation of exosomal small libraries with over > 5 million reads per sample. We identified 338 known miRNAs in lymphoma cells and 335 known miRNAs identified in lymphoma exosomes. Although there was considerable overlap for highly abundant microRNAs, some microRNAs are confined within the cells, whereas others appear preferentially sorted into the endosomal-exosomal pathway. The rest of analyzed reads encompasses sequences matching rRNAs, tRNAs, piRNAs, genomic repeats, EBV-related miRNAs and many (∼500) unknown novel miRNAs. Using the latest deep-sequencing techniques, we confirmed that miRNAs are an abundant class of RNA molecules in EBV-cancer exosomes. Importantly, the most prevalent cellular miRNAs are also abundant in exosomes, providing the additional support to employ exosomal small-RNAs as circulating biomarkers.

Funded by: AICR, The Association for International Cancer Research, UK; NWO, The Netherlands Organisation for Scientific Research

**Deep sequencing of RNA in cell-derived vesicles reveals the selective incorporation of specific sets of small non-coding RNAs with potential regulatory function**

Esther N.M. Nolte-‘t Hoen^1^, Henk P.J. Buermans^2^, Maaike Waasdorp^1^, Willem Stoorvogel^1^, Peter A.C. 't Hoen^2^ and Marca H.M. Wauben^1^

^1^Department of Biochemistry & Cell Biology, Faculty of Veterinary Medicine, Utrecht University, Utrecht, The Netherlands; ^2^Center for Human and Clinical Genetics, Leiden University Medical Center, Leiden, The Netherlands

Email: e.n.m.nolte@uu.nl

Shuttling of RNA into vesicles allows cells to disseminate genetically encoded messages, which may modify the function of target cells. Although the presence of microRNA (miRNA) in these vesicles has been frequently reported, the full range of small RNA biotypes present in these vesicles has not been analyzed in depth. We applied deep sequencing for an unbiased screen of small (<70 nt) RNAs in vesicles released during interaction of dendritic cells and T cells. This key event in the initiation of immune responses induces increased release of nanosized vesicles. We found that miRNAs formed only a minority of vesicle-enclosed small RNA species. In contrast, several other classes of small non-coding RNAs with regulatory capacity were highly abundant in cell-derived vesicles. Remarkably, most of these RNA biotypes were polymerase III-generated transcripts. The most abundant RNAs included RNA repeat sequences and fragments of known non-coding transcripts that could regulate gene expression similar to miRNAs. In addition, the cell-derived vesicles were highly enriched in a set of non-coding structural RNA species that may facilitate the incorporation of RNA subsets into vesicles or their functional transfer to target cells. Conclusively, we found several leads for unraveling how cells can modify other cells via nan-sized vesicles containing selectively incorporated RNA species.

Funded by: Netherlands Genomics Initiative (NGI) Horizon Breakthrough Project grant (No. 93519013)

**Exosomes as gene delivery vehicles—limits of size and chemistry with electroporation**

Yiqi Seow, Yan Teng Yew, Wei Mei Guo and Sydney Brenner

Molecular Engieering Laboratory, Science and Engineering Institutes, Singapore

Email: seowy@scei.a-star.edu.sg

Exosomes has the potential to be used as effective gene delivery vehicles in vivo, and to that end, we have previously shown that exosomes can be loaded with siRNA and targeted after intravenous delivery in a murine model. Electroporation of exosomes enables the encapsulation of siRNA while maintaining structural integrity of the exosomes as measured by NTA. However, the ability of exosomes to encapsulate other genetic cargoes is not clear. Here, we will present data demonstrating the efficiency of electroporation for encapsulation of genetic material including ssDNA, dsDNA, plasmid and RNA of different sizes. This knowledge will help establish the limits of exosome-mediated gene delivery.

Funded by: Agency for Science, Technology and Research, Singapore

**Delivery of miRNA to the EGFR-expressed tumor by systemic injection of targeted exosomes**

Shin-Ichiro Ohno^1^, Masakatsu Takanashi^1^, Katsuko Sudo^2^,Takayuki Mizutani^1^, Takahiro Ochiya^3^ and Masahiko Kuroda^1^

^1^Department of Molecular Pathology, Tokyo Medical University, Shinjyuku-ku, Tokyo, Japan; ^2^Animal Research Center, Tokyo Medical University, Shinjyuku-ku, Tokyo, Japan; ^3^Division of Molecular and Cellular Medicine, National Cancer Center Research Institute, Tsukiji, Chuou-ku, Tokyo, Japan

Email: kuroda@tokyo-med.ac.jp

Despite the therapeutic potential of nucleic acid drugs, their clinical application is limited in part by the lack of appropriate delivery systems. Exosomes or microvesicles are small endosomally derived vesicles that are secreted by a variety of cell types and tissues. Here, we show that exosomes can be used to deliver microRNA efficiently to epidermal growth factor receptor (EGFR)-expressing breast cancer cells. Targeting was achieved by engineering the donor cells to express the transmembrane domain of platelet-derived growth factor receptor fused to the GE11 peptide. Intravenously injected exosomes delivered let-7a to xenografted EGFR-expressing breast cancer tissue in Rag2-/- mice. Our results suggest that using exosomes to target EGFR-expressing cells may allow delivery of nucleic acid drugs to cancerous tissues.

Funded by: The ‘‘Private University Strategic Research–Based Support Project: Epigenetics Research Project Aimed at a General Cancer Cure Using Epigenetic Targets’’ from the MEXT (Ministry of Education, Culture, Sports, Science and Technology (Japan) and in part by a Grant-in-Aid for scientific research on Priority Areas (B) and (C) from the MEXT (Japan) and the Tokyo Medical University Cancer Research Foundation (Japan).

**Micromanaging of microenvironmental endothelial cells by breast cancer cells via exosomal microRNAs**

Nobuyoshi Kosaka, Yusuke Yoshioka, Keitaro Hagiwara, Fumitaka Takeshita and Takahiro Ochiya

Division of Molecular and Cellular Medicine, National Cancer Center Research Institute, Tokyo, Japan

Email: nkosaka@ncc.go.jp

Our recent study demonstrated that neutral sphingomylinase 2 (nSMase2) regulates exosome-mediated microRNAs (miRNAs) secretion and that secreted miRNAs showed cell-to-cell transfer. Here, we present that secretory miRNAs are vital for cancer cell metastasis via controlling tumor angiogenesis. The nSMase2-knockdown breast cancer cells, in which the amount of exosomes and secretory miRNAs were downregulated, did not exhibit any differences compared with control cells in vitro proliferation, invasion and migration assay. Although growth of tumor was not different as well between these cell lines in vivo; however, surprisingly, the metastatic ability to the lungs in the xenograft model was remarkably inhibited in this cell line, suggesting that exosome from cancer cells might influence the status of their microenviromental cells. To investigate the microenvironment in the primary tumor site, we performed an immunohistological analysis against CD31 and found that number of blood vessels in the inoculated nSMase2 knockdown cells was less than that of control cells. Interestingly, some sets of angiogenic miRNAs were found in exosomes isolated from metastatic breast cancer cell line, indicating that exosome secretion containing angiogenic miRNAs can contribute to the formation of tumor vessel network, thereby promoting the metastasis.

Funded by: A Grant-in-Aid for the Third-Term Comprehensive 10-Year Strategy for Cancer Control, a Grant-in-Aid for Scientific Research on Priority Areas Cancer from the Ministry of Education, Culture, Sports, Science and Technology, and the Program for Promotion of Fundamental Studies in Health Sciences of the National Institute of Biomedical Innovation (NiBio), and the Japan Society for the Promotion of Science (JSPS) through the “Funding Program for World-Leading Innovative R&D on Science and Technology (FIRST Program)” initiated by the Council for Science and Technology Policy (CSTP).

**BEAMing qRT-PCR analysis of IDH1 mutant in tumor microvesicles**

Leonora Balaj, Walter Chen, Linda M Liau, Horacio Soto, Mathew Garret, Lin Dan Zhu, Sarada Sivaraman, Eric T. Wong, Bob Carter, Fred Hochberg, Xandra O. Breakefield, Johan Skog

Breakefield Laboratory, Departments of Neurology and Radiology, Massachusetts General Hospital, and Neuroscience Program, Harvard Medical School, Boston, MA, USA

Email: Balaj.Leonora@mgh.harvard.edu

Biofluid-based diagnostic tools allow for the molecular characterization of tumors in a minimally invasive fashion. One major obstacle facing all such techniques is assay sensitivity, since the vast majority of the interrogated genetic material is usually not only from the tumor itself but also from normal cells. We were interested in characterizing the genetic composition of microvesicles secreted by gliomas into cerebrospinal fluid (CSF). Because these microvesicles carry the mRNA of the parent cell, we hypothesized that they could be used to accurately assess mutational profiles of tumors in a minimally invasive manner. We thus developed a tumor diagnostics technology that incorporates analysis of microvesicles from CSF with BEAMing (Beads, Emulsions, Amplification, Magnetics) PCR into a single platform. BEAMing PCR is a highly sensitive form of digital PCR that allows both detection of extremely rare sequence variants using FACS analysis and quantification of the percentage of mutant copies in a starting population of mRNA. To determine the efficacy of this technology, we assayed CSF microvesicles from patients with glioma tumors, which had been typed for a dominant mutation (G395A) in the isocitrate dehydrogenase (IDH1) gene. IDH1 is a [NADP + ]-dependent cytosolic enzyme that is mutated in 80% of grades II–III gliomas and secondary glioblastomas. Initial results indicate that we can detect mutant RNA in CSF microvesicles with high specificity and sensitivity. We are currently expanding our study to include more samples and to expand this analysis to serum samples. This is the first time a point mutation has been detected in CSF samples from glioma patients. This finding expands the diagnostic potential of circulating microvesicles and demonstrates the great promise for this technology in next-generation companion diagnostics and mutational profiling of individual tumors.

Funded by: NIH/NCI grants CA069246 (F.H., X.O.B., B.C.), CA141226 (X.O.B.), CA156009 (X.O.B.) and CA141150 (X.O.B.); Brain Tumor Funders’ Collaborative (B.C., X.O.B.); American Brain Tumor Association (ABTA; J.S.); Harvard Catalyst (B.C.); Hyugens Scholarship NL (L.B.)

**MicroRNA profiling of prostate cancer cell-derived oncosomes identifies a signature of invasion and metastasis**

Matteo Morello^1,2^, Andrew C. Dudley^3^, Peter Schow^4^, Rosalyn M. Adam^2^, Samantha Morley^2^, David Mulholland^5^, Luigi Insabato^6^, Marsha A. Moses^3^, Francesca Demichelis^7,8^, Michael P. Lisanti^9^, Hong Wu^10^, Michael Klagsbrun^3^, Neil A. Bhowmick^11^, Mark A. Rubin^7^, Crislyn D'Souza-Schorey^12^, Michael R. Freeman^1,2,13^ and Dolores Di Vizio^1,2^

^1^Departments of Surgery and Biomedical Sciences, Samuel Oschin Comprehensive Cancer Institute, Cedars-Sinai Medical Center, Los Angeles, CA, USA; ^2^Departments of Surgery and Biological Chemistry and Molecular Pharmacology and ^3^Department of Surgery and Vascular Biology Program, Children's Hospital Boston and Harvard Medical School, Boston, MA, USA; ^4^Dana Farber Cancer Institute Flow Cytometry Core Facility, Boston, MA, USA; ^5^Department of Molecular and Medical Pharmacology, Institute for Molecular Medicine, David Geffen School of Medicine, University of California at Los Angeles, CA, USA; ^6^Department of Biomorphological and Functional Science, University Federico II, Naples, Italy; ^7^Department of Pathology and Laboratory Medicine, and ^8^Institute for Computational Biomedicine, Weill Cornell Medical College, New York, NY, USA; ^9^Department of Medical Oncology, Kimmel Cancer Center, Thomas Jefferson University, Philadelphia, PA, USA; ^10^Department of Molecular and Medical Pharmacology, Institute for Molecular Medicine, David Geffen School of Medicine, University of California at Los Angeles, CA, USA; ^11^Uro-Oncology Research Program, Samuel Oschin Comprehensive Cancer Institute, Cedars-Sinai Medical Center, Los Angeles, CA, USA; ^12^Department of Biological Sciences, University of Notre Dame, Notre Dame, IN, USA; ^13^The Urological Diseases Research Center, Children's Hospital Boston, Boston, MA, USA

Email: dolores.divizio@cshs.org

We recently demonstrated that amoeboid prostate cancer (PCa) cells shed bioactive membrane particles (oncosomes), including novel particles up to 10 *µ*m in diameter. Oncosomes can transfer oncogenic signals horizontally within the tumor microenvironment, stimulating migration of tumor and endothelial cells. They contain proteases, Caveolin-1, the GTPase ARF6 and other proteins that we have used as markers to identify them in the circulation of mice with PCa and in murine and human tissues with metastatic disease. Oncosomes also contain miRNAs. Aberrant expression of miRNAs has been linked with pathogenesis of prostate cancer. In order to identify oncosome-specific miRNAs relevant to metastasis, we performed a GeneChip^®^ miRNA 2.0 Array screen of oncosomes shed by tumorigenic RWPE-2 PCa cells. Non-tumorigenic RWPE-1 cells were also analyzed. The miRNA profile of the oncosomes was compared to the miRNA profile of the cells of origin. Interestingly, while the miRNA profile of RWPE-2 almost completely matched the profile of RWPE-1 cells, the profiles differed significantly between oncosomes isolated from the two cell lines. In particular, RWPE-2-derived oncosomes contained miR-1228*, miR-150*, miR-373*, miR-135a*, miR-34b and miR-491-3p, which have been implicated in cancer cell proliferation and migration. In particular, the miRNAs miR-135a* and miR-373* target and inhibit the focal adhesion kinase (FAK), which plays a role in promoting a mesenchymal to amoeboid transition. Moreover, the miR-135a* and miR-373* have also been demonstrated to stimulate cell invasion and are upregulated in metastatic prostate and breast cancer. Target prediction analysis identified members of pathways involved in metabolic deregulation and in oncogenic intercellular signaling and cell-cell interaction specifically in the oncosomes purified from the tumorigenic cell line. Collectively, our results indicate that tumor cells can use oncosome formation to actively export prooncogenic miRNAs and that oncosomes therefore serve as a potential source of biomarkers of tumor progression.

Funded by: NCI K99CA131472 (to D.D.V.), NIH R01CA143777 and R01DK57691 (to M.R.F.), and PDA 09A107 from the American Institute for Cancer Research (S.M.)

**Secreted exosomes from cultured bladder cells are enriched for distinct miRNAs detected in circulation of metastatic bladder cancer patients**

Marie Stampe Ostenfeld^1^, Dennis Jeppesen^1^, Jens Preben Morth^2^, Huy Bui Khanh^3^, Dan Theodorescu^4^, Michael Borre^5^, Lars Dyrskjøt^1^ and Torben Ørntoft^1^

^1^Department of Molecular Medicine, Aarhus University Hospital, Skejby, Aarhus N, Denmark; ^2^Centre for Molecular Medicine Norway (NCMM), University of Oslo, Oslo, Norway; ^3^EMBL Heidelberg, Heidelberg, Germany; ^4^Department of Urology, Aarhus University Hospital, Skejby, Denmark; ^5^University of Colorado Cancer Center, Aurora, CO, USA

Email: marie.stampe@ki.au.dk

Exosomes are small nanovesicles (30–100 nm) secreted from the cell surface of most cell types. The vesicles are secreted at increased levels by cancer cells and can be detected in body fluids of cancer patients. Exosomes function in paracrine delivery of proteins, RNA and DNA into recipient cells. By this, exosomes are speculated to be involved, e.g., in premetastatic niche formation at distant sites. Here, we present a characterization of exosome vesicles isolated from bladder cancer cells as examined by electron microscopy, dynamic light scattering and Western blotting of common exosome protein markers. The expression of 671 unique human miRNAs by qPCR was analyzed in both exosomes and donor cells from: (1) cultivated normal urothelial cells (NHU), (2) T24 bladder carcinoma cell line (non-metastatic) and its metastatic isogenic derivates FL3 and SLT4 and (3) UMUC3 bladder carcinoma cell line (moderate metastatic) and its highly metastatic isogenic derivative LUL2. Several miRNAs were detected in the vesicles yet absent in their parental donor cell indicating a possible preferential vesicular packaging. miRNAs miR92a, -106a, -20a, -21, -16, and let-7a/b/d/e were detected among the top100 ranked miRNAs in all vesicles. Among the top 30 enriched exosomal miRNAs, miR-223 and-383 were detected in all cancer vesicles and miR-921 in the highly metastatic vesicles (FL3, SLT4 and LUL2). The release of miRNAs (miR-21, -16 and let7b) from FL3 cells into the culture media was time dependent and a fraction of these miRNAs were stable to RNaseOne degradation indicating possible protection inside the vesicles. Examination of miRNAs in human plasma samples also suggested resistance toward endogenous RNAse degradation as well as toward repetitive freeze/thawing. The profiling of miRNA expression by Affymetrix microarray analysis of crude exosome-containing fractions of human plasma samples (10 healthy controls and 10 cases of metastatic bladder cancer) showed significant upregulation in cancer plasma of, e.g., miR-92a, -106a, -23a, -20a and -21, some of which were the same as the miRNAs highly expressed in the tumor vesicles. The significant increase was confirmed by q-RT-PCR for miR-92a and -23a and validated on an independent set of plasma samples (8 healthy controls and 8 metastatic cancer cases). Our results suggest that specific miRNAs are encapsulated into exosomes in bladder cancer and that these may be detected in circulation in advanced stages of bladder cancer.

Funded by: Danish Cancer Society, Toyota Foundation, Novo Nordisk Foundation

#### 2. Nanotechnology meets Biology—the Next Leap in Exosomes and Microvesicles Biology?

**Nanotechnology meets biology**

Fredrik Höök

Chalmers, Gothenburg, Sweden

(no abstract)

**Microfluidic crossflow filtration system to isolate exosomes from blood**

Ryan Davies, Junho Kim, Yong Song Gho and Jaesung Park

Department of Mechanical Engineering, POSTECH, Korea

Email: rtd123@postech.ac.kr

Various mammalian cells including tumor cells secrete exosomes. Tumor-derived exosomes may be potential targets for blood-based cancer diagnosis. However, studies performed on bloodborne exosomes to date have been limited by lack of effective, standardized purification strategies. Using in situ prepared nanoporous membranes, we are developing a strategy employing a microfluidic crossflow filtration system to isolate exosomes from blood samples. This method can be applied to purify nanosized particles from blood allowing isolation of intact exosomes, avoiding the need for laborious and potentially damaging centrifugation steps or overly specific antibody-based affinity purification. Continuous flow mode also allows high throughput sample processing. Porous polymer membranes were integrated into PMMA microfluidic chips by benchtop UV photopolymerization through a mask, allowing positioning of membrane elements while preserving simplicity of device preparation. Pore size could be manipulated by changing the ratio of porogenic solvent to prepolymer solution and was tuned to a size proper for extraction of exosomes. From the whole blood of melanoma-grown mice, we isolated exosomes and performed RT-PCR to verify their contents of mRNA. Melan A mRNA present in melanoma-derived exosomes were found in filtered samples, while the complexity of raw blood prevented markers from being detected in original samples. This filtration system can be incorporated into other microdevices enabling integrated sample preparation for the downstream analysis of blood-based exosomes, realizing the direct handling of an original clinical sample without pretreatment.

Funded by: National Research Foundation of Korea (NRF) grant funded by the Korea government (MEST) (2011-0030075, 2011-028845 and 2010-004003)

**Selective isolation of tumor microvesicles from serum using microfluidic capture**

K.E. van der Vos, N. Atai, F.P. Floyd, Jr., S. Sivaraman, J. Skog, M. Toner,X.O. Breakefield and S.L. Stott

Departments of Neurology and Radiology, Massachusetts General Hospital, and Neuroscience Program, Harvard Medical School, Boston, MA, USA; Center for Engineering in Medicine, Massachusetts General Hospital, Harvard Medical School, Boston, MA, USA; Exosome Diagnostics, Inc., New York, NY, USA

Email: vandervos.kristan@mgh.harvard.edu

Cancer cells release microvesicles (MVs) containing genetic information from the tumor into the bloodstream that can serve as a diagnostic marker for disease status. However, human serum also contains a variety of MVs released from normal cells that may hamper mutational analysis of tumor-derived MVs. To increase our sensitivity of detection of oncogenic mutations and reduce background noise, we used antibody-mediated capture in microfluidic chambers to selectively isolate tumor MVs from serum. Efficient MV capture was validated using defined amounts of tumor MVs from a mouse glioblastoma cell line expressing human EGFRvIII spiked into healthy human serum. Samples were passed through microfluidic chambers coated with an EGFR antibody, using a herring-bone pattern to maximize contact of MVs with the chamber surface. Captured MVs were lysed inside the chip and RNA was isolated and compared with input and output RNA. RT-PCR analysis of tumor-specific EGFRvIII RNA showed that tumor MVs were markedly enriched in the chamber. This enrichment was illustrated visually by labeling tumor MVs with PKH67 and fluorescent imaging of the devices. In conclusion, tumor-derived MVs in serum can be selectively captured in microfluidic chambers, providing a source of information about the genetic status of tumors.

Funded by: NIH (NCICA069246) (to X.O.B) and a Quantum Grant from the National Institute for Biomedical Imaging and Bioengineering (to M.T.). K.vdV. is supported by the Dutch Scientific Organisation (NWO-Rubicon) and S.L.S. is supported through an American Cancer Society New England Division Research Grant.

**Microparticle measurements by nanoparticle tracking analysis and dynamic light scattering**

S. Nejlund, M. Mørk, S. Pedersen and S.R. Kristensen

Department of Clinical Biochemistry, Aalborg Hospital, Aarhus University Hospital, Aalborg, Denmark

Email: snejlu06@student.aau.dk

*Background*: Microparticles (MP) produced by cellular activation or apoptosis exert a potential role as biomarkers. New technological possibilities to detect MP have emerged but have not been evaluated clinically. The aim of this study was to assess and compare two novel devices recently developed for the characterization of MP, namely nanoparticle tracking analysis (NTA) (NanoSight^®^) and dynamic light scattering (DLS) (ThromboLUX^®^), i.e., to evaluate preanalytical procedures and accuracy and imprecision of the methods. *Methods*: Polystyrene beads were used to determine the accuracy of the size measurements. The effect of various preanalytical variables including storage, freezing and centrifugation on plasma obtained from healthy and diseased persons besides imprecision of measurements were evaluated. *Results:* Both methods were accurate and reproducible for particle sizes ranging from 50 nm to 350 nm. Preliminary data showed no substantial differences between two double-centrifugation procedures, storage at −80°C up to 1 month did not influence the results, and a substantial higher concentration of MP in patient samples compared to the healthy samples was found. CV (between days) was in the range of 7–15% for size determinations and 20–30% for concentrations in plasma. *Conclusion*: Both NTA and DLS can be used for MP detection displaying rather reproducible measurements, although with some imprecision. ThromboLux^®^ is very user-friendly whereas NanoSight^®^ requires some experience but gives more detailed results.

**Characterization of human cell-derived exosomes by nanoparticle tracking analysis**

Anna-Kristin Ludwig, Viktoriya Sokolova, Thorsten R. Doeppner, Stefan Durst, Sandra Hornung, Johannes Ruesing, Matthias Epple, Peter A. Horn and Bernd Giebel

Institute for Transfusion Medicine, University Hospital Essen, Essen, Germany; Giebel Institute for Inorganic Chemistry, University Duisburg–Essen, Essen, Germany; Department of Neurology, University Hospital Essen, Essen, Germany

Email: anna-kristin.ludwig@uk-essen.de

Exosomes derived from otherwise non-manipulated or genetically manipulated, CD63-eGFP expressing human embryonic kidney cells (HEK 293T) were characterized by scanning electron microscopy (SEM) and nanoparticle tracking analysis (NTA). Exosomes from both cell types revealed a diameter of around 110 nm. The stability of purified exosomes was examined following storage at −20°C, 4°C and 37°C for different time periods. The size of the exosomes progressively decreased over time at 4°C and 37°C, possibly indicating a dissociation of aggregated vesicles or a continuous degradation. Multiple cycles of freezing (−20°C) and thawing did not affect the exosome size. In addition, exosomes released by CD63-eGFP expressing HEK 293T cells showed a green fluorescence signal. Upon supplementation of culture media with eGFP-labeled exosomes, mesenchymal stromal cells showed an incorporation of these exosomes. Notably, mesenchymal stromal cells incorporated freshly prepared exosomes as well as exosomes that have been frozen and thawed for up to 3 times. These results suggest that exosomes can be stored at −20°C without changing their structural properties.

Funded by: IFORES

**Visualizing and tracking microvesicles with membrane-targeting fluorescent proteins**

Charles Lai

Departments of Neurology and Radiology, Massachusetts General Hospital, MA, USA; Neuroscience Program, Harvard Medical School, Charlestown, MA, USA

Email: lai.charles@mgh.harvard.edu

Due to their nanoscale size (<1µm in diameter), MVs are currently visualized by electron and conventional microscopy techniques using MV-specific antibodies, direct fluorescent chemical labeling of MVs and in MV-donor cells, as well as exogenous expression of “vesicle-specific” proteins fused with fluorophores. Here, we describe a method to visualize MVs by using membrane-targeting fluorescent proteins. By fusing N-terminus palmitoylation signal with either enhanced green fluorescent protein (palm-GFP) or tdTomato (palm-tdTomato) and introducing them into MV-producing cells via lentiviral infection, MVs isolated from the transduced cells by ultracentrifugation were readily visible under epifluorescence and confocal microscopy using corresponding excitation wavelength for each fluorophore. To verify this finding, isolated MVs were further processed by sucrose gradient centrifugation, and both palm-GFP and palm-tdTomato was confirmed to be specifically incorporated in MVs by Western blot analysis of appropriate fractions. Using this paradigm, we also successfully tracked MV release from donor cell and their subsequent uptake by recipient cells, as well as exchange of MVs between two separate cell populations by live-cell confocal microscopy. Efforts are currently underway to analyze the interactive dynamics of MV exchange between two cell populations. This is the first report of a general MV labeling strategy that encompasses the entire MV populations and allows immediate detection and tracking of MVs without further sample manipulation. Since MV populations are generally divided by their sizes, different subtypes can be differentiated by size exclusion, either by filtering steps during MV isolation or microscopic image analysis.

Funded by: National Cancer Institute (NCI) Canadian Institute of Health Research (CIHR)

**Determination of exosome concentration using surface-based sensing**

Deborah Rupert, Marta Bally, Cecilia Lässer, Maria Eldh, Jan Lötvall and Fredrik Höök

Biological Physics, Chalmers University of Technology, Göteborg, Sweden; Respiratory Medicine and Allergology, Göteborg University, Göteborg, Sweden

Email: rupert@chalmers.se

Despite the increased evidence of the importance of exosomes in a broad range of physiological processes and pathological conditions, accurate methods capable of quantifying their concentration in biological fluids remain lacking. We present a generic approach for exosome quantification. As a proof of principle, the concentration of CD63-positive exosomes released from human mast cells has been analyzed. Our platform is based on determining the rate of binding and the number of individual exosomes captured on a surface via exosome-specific antibodies. To suppress non-specific binding, the surface is passivated with a polyethylene-glycol coating and further functionalized with anti-CD63 antibodies. For read-out, we evaluate three surface-sensitive methods: quartz crystal microbalance with dissipation (QCM-D), surface plasmon resonance (SPR) and total internal reflection fluorescence (TIRF). The first two methods are label free and monitor the adsorbed mass versus time upon binding of the object of interest onto the sensor surface. TIRF microscopy is used for direct observation of the binding of fluorescently labeled exosomes. In the latter case, high fluorescent signals were obtained using a novel staining method based on self-insertion of fluorescently labeled oligonucleotides into the lipid membrane, allowing each individual exosome to be detected and counted. Each technique is discussed in view of their advantages and disadvantages and further compared to a commercial solution-based quantification method (Nanoparticles Tracking Analysis, Nanosight).

Funded by: VINNOVA: Innovation for future health

#### 3. Tissue Function and Remodeling in Relation to Extracellular Vesicles

**Production of extracellular vesicles by cancer cells harboring oncogenic epidermal growth factor receptor (EGFR): impact of the microenvironment and epithelial-to-mesenchymal transition**

J. Rak, D. Garnier, C. Milsom, V. Bentley, T.-H. Lee, L. Montermini, K. Al-Nedawi and B. Meehan

McGill University, Montreal Children's Hospital, Montreal, Canada

Email: janusz.rak@mcgill.ca

Cancer cells release increased amounts of extracellular vesicles (EVs) in a manner that is influenced by oncogenic pathways. Epidermal growth factor receptor (EGFR) in its upregulated or mutant form promotes formation of EVs and becomes incorporated into their cargo. This process leads to formation of vesicles containing an active oncogene (oncosomes), which may impact the phenotypes of cells they interact with and trigger features of malignancy in them (e.g., angiogenic phenotype). However, EVs may also contain opposing influences, such as tumour suppressor proteins, including PTEN, and their emission and biological activity may be modulated by non-genetic events. In the present study, we examine vesiculation during the processes of epithelial-to-mesenchymal transition (EMT), hypoxia and under influence of targeted anticancer agents. Thus, mesenchymal phenotype can be induced in cancer cells by stimulation with growth factors and inhibitors of E-cadherin. Under such conditions A431 cells change their EV output, both quantitatively and qualitatively, and in terms of both EV size and cargo. This is revealed by the application of nanoparticle tracking technology (NTA), testing for EGFR, tissue factor (TF) and an unbiased profiling of the cellular proteome. The EV profiles are also affected by hypoxic conditions, and the content of EGFR and PTEN modulate these responses in human glioblastoma cells. Finally, exposure to agents targeting EGFR in vitro and in vivo alters the profiles of cancer cell-derived EVs. Thus, EVs emanating from cancer cells and stroma may serve as a source of biological information as to the genetic status, molecular subtype, functional state, microenvironment and therapeutic stress, affecting these cellular populations and relevant for the biology of the disease.

Funded by: Canadian Institutes of Health Research

**Angiogenic activity of tumor-derived extracellular vesicles**

Yong Song Gho, Yae Jin Yoon, Dong-Sic Choi, E.J. Choi, J.H. Kim, Y.K. Kim and K.P. Kim

Division of Molecular and Life Sciences, POSTECH, Pohang, Republic of Korea

Email: ysgho@postech.ac.kr

The secretion of extracellular vesicles (EVs), otherwise known as exosomes and microvesicles, is a universal cellular process occurring from simple organisms to complex multicellular organisms, including humans. Throughout evolution, both prokaryotic and eukaryotic cells have adapted to manipulate EVs. Actively growing tumor cells shed EVs into extracellular milieu, and the rate of shedding increases in malignant tumors. Although recent progress in this area has revealed that tumor-derived EVs play multiple roles in tumor growth and metastasis via immune escape, tumor invasion and angiogenesis, the detailed mechanism on how EVs elicits endothelial cell activation has not been studied extensively. Here, we investigated in detail the mechanism governing the angiogenic activities of tumor-derived EVs by interrogating proteomic and genomic data using systems approaches. Colorectal cancer cell-derived EVs harbored several proteins and mRNAs that might function in tumor progression via the induction of neovascularization. By global transcriptomic analysis of human endothelial cells treated with colorectal cancer cell-derived EVs, furthermore, we identified several candidate pathways involved in tumor EV-mediated endothelial cell activation. Our study suggests that EVs of cancer cells can be involved in tumor growth and metastasis by facilitating angiogenesis-related processes. This information will help elucidate the pathophysiological functions of tumor-derived EVs and aid in the development of cancer diagnostics.

Funded by: Mid-career Researcher Program of NRF grant funded by the Korea government MEST (No. 20110016474)

**Cardiomyocyte progenitor cell and mesenchymal stem cell derived exosomes stimulate angiogenesis in vivo and in vitro**

Krijn R. Vrijsen, Steven A.J. Chamuleau, C.H.G. Metz, J.C. Deddens, Pieter A.F.M. Doevendans and Joost P.G. Sluijter

University Medical Center Utrecht, Utrecht, The Netherlands

Email: k.vrijsen@umcutrecht.nl

Cardiac cell transplantation therapy is suggested to be a novel therapeutic approach for chronic patients with impaired heart function. Although promising, cell injections lead mainly to increased capillary density, while only a small percentage of transplanted cells successfully engraft the myocardium. These phenomena could be explained by paracrine factors, including exosomes, secreted by transplanted (stem) cells, thereby stimulating endogenous repair. Here, we focused on the stimulating effect of human cardiac-derived cardiomyocyte progenitor cell (CMPC) and bone marrow-derived mesenchymal stem cell (MSC) exosomes on endothelial cells in vitro and on the angiogenic capacity in an in vivo matrigel plug assay. In addition, we studied the role of extracellular matrix metalloproteinase inducer (EMMPRIN) on exosomes in vitro. CMPCs and MSCs exosomes, isolated from the conditioned medium by centrifugation at 100,000g or separated on a sucrose gradient, are visualized via electron microscopy and Western blot analysis for exosomal marker flotillin-1. Both types of exosomes are approximately 100 nm in size and have a density between 1.10 and 1.12 g·ml^−1^. In vitro, both types of exosomes stimulate angiogenesis of different types of endothelial cells. In a scratch wound assay, spheroids assay and in a matrigel assay, exosomes enhance the migration, tubular formation and organization of endothelial cells, respectively. When treated with an EMMPRIN antibody, the stimulating effect of exosomes on endothelial cells was reduced. In vivo MSC and CMPC exosomes lead to a higher influx of cells (total cell number) into the matrigel plug implanted in C57Bl6 mice (*n =*4–5). There was no significant difference in the percentage of PECAM and SMA+ cells into the plug, although a trend revealing that CMPC exosomes lead to more SMA+ cells could be observed. CMPC and MSCs release exosomes, which can enhance angiogenesis in vitro and in vivo. This action could be a novel mechanism in which transplanted cells communicate with their surroundings and explain partly the paracrine effects observed upon cardiac cell transplantation.

Funded by: This research forms part of the Project P1.04 SMARTCARE of the research program of the BioMedical Materials institute, co-funded by the Dutch Ministry of Economic Affairs, Agriculture and Innovation. The financial contribution of the Nederlandse Hartstichting is gratefully acknowledged.

**Exosomal-TGFbeta drives enhanced angiogenesis through recruitment of tumor stroma**

Jason Webber, Lisa Spary, Malcolm Mason, Zsuzsanna Tabi and Aled Clayton

Department of Pharmacology, Oncology & Radiology, School of Medicine, Cardiff University, Velindre Cancer Centre, Whitchurch, Cardiff, United Kingdom

Email: webberjp@cf.ac.uk

The stroma surrounding carcinomas are often associated with the presence of altered stromal cells, which resemble myofibroblasts. Myofibroblasts drive remodeling of extracellular matrix and produce assorted growth factors, which promote tumour growth, vascularization and metastasis. We have studied the role of cancer exosomes in stromal cell activation in prostate cancer, demonstrating exosomes can trigger normal-prostatic stromal cells to acquire a phenotype and function that mimics that of disease-derived stroma. This exosome effect is TGFbeta1 dependent, but the phenomenon cannot be reproduced using soluble TGFbeta1. Targeted knockdown of Rab27a in Du145 cells reduced exosome but not soluble TGFbeta1 secretion. This led to abrogated stromal-differentiation and a subsequent negative impact on stromal cell proangiogenic influence. These data suggest a role for exosomal-TGFbeta and not soluble TGFbeta as the physiological modulator of the tumour stromal environment, driving multiple changes supporting disease progression.

Funded by: Cancer Research Wales; Prostate Action

**Vascular smooth muscle cell-derived exosomes isolated from human blood are enriched with fetuin-A and may be involved in the mechanisms of vascular calcification**

Sundeep S. Kalra, Alexander Kapustin and Catherine Shanahan

King's College, London, United Kingdom

Email: sundeep.kalra@nhs.net

*Background & Aims*: In vitro, vascular smooth muscle cell (VSMC)-derived exosomes (EX) usually contain the calcification inhibitor fetuin-A. However, we have recently shown in vitro that, in the absence of fetuin-A, VSMCs secrete EXs, which can trigger nucleation of calcium phosphate and thereby mediate vascular calcification (VC). In vivo, lower levels of circulating fetuin-A are associated with VC. We hypothesize that, in vivo, VSMC EXs are released into the blood and their fetuin-A content may reflect calcification stress. *Methods & Results*: EXs isolated from human whole blood by differential centrifugation were enriched with the exosomal marker CD63 and contained the VSMC-specific marker α-smooth muscle actin, as detected by Western blot. VSMC EXs from both patients with quantified VC and healthy controls contained fetuin-A; however, we observed lower levels of fetuin-A in subjects with VC. In a cohort (*n*=16) of patients with quantified VC, we investigated the relationship between exosomal fetuin-A and quantified VC (computed tomography-derived calcium score). Linear regression analysis showed negative correlation between the exosomal fetuin-A:calcium ratio and calcium score (*r* ^2^= − 0.73; *p*=0.05). *Conclusions*: VSMC-derived EXs are present in the human circulation and contain the VC inhibitor fetuin-A. A negative correlation exists between VSMC exosomal fetuin-A and clinical calcification, confirming previous in vitro observations, that fetuin-A enriched VSMC EXs may inhibit VC.

Funded by: King's College Hospital Research and Development Grant

**Calcifying matrix vesicles released from macrophages**

S.E.P. New^1^, J.F. Marchini^1^, M. Aikawa^1^, C.M. Shanahan^2^, K. Croce^1^ andE. Aikawa^1^

^1^Center for Interdisciplinary Cardiovascular Sciences, Brigham and Women's Hospital, Harvard Medical School, Boston, MA, USA; ^2^King's College London, London, United Kingdom

Email: snew@partners.org

Chronic renal disease (CRD) accelerates formation of macrophage-rich atherosclerotic plaques and calcification. However, the mechanism behind CRD-acerbated calcification remains obscure. Here, we hypothesized that macrophages release microcalcification-generating matrix vesicles (MV), promoting atherosclerotic calcification under CRD conditions. Macrophage number and calcified area were increased in apoE − /− mice with CRD vs. apoE − /− mice (*p*<0.001 and *p*<0.01, respectively; *n*=24). Near-infrared fluorescence microscopy, using hydroxyapatite-binding imaging agent, detected microcalcifications, containing vesicular structures. S100A9, a newly suggested promoter of inflammation and calcification, was identified to be increased in calcifying plaques and co-localized with both macrophages and microcalcifications. ApoE − /−S100A9 − /− mice contained fewer lesions (*p*<0.02) and less macrophages (*p*<0.003) compared with the apoE − /− cohort (*n*=9 per group). Electron microscopy identified the presence of calcifying MV in plaques of apoE − /− mice with CRD. Elevated calcium and phosphate levels, comparable to those observed in CRD patients, increased the calcific potential of macrophage-derived MV (RAW 264.7) (*p*<0.01; *n*=6). These conditions did not induce apoptosis, and thus this effect is not attributed to apoptotic bodies. The addition of S100A9 further enhanced the calcific potential. The expression of S100A9 and annexin V is enriched in calcifying MV, suggesting a role for them in this mechanism. This demonstrates a direct role of macrophage-derived MV in inflammation-driven calcification associated with CRD.

Funded by: Kowa Company, Ltd.

**The circulating calcification inhibitor fetuin-A is recycled via the exosomal pathway in vascular smooth muscle cells**

Alexander Kapustin^1^, Ignat Drozdov^1^, Daniel Soong^1^, Daniel Alvarez-Hernandez^1^, Pilar Sanchis^1^, Xiaoke Yin^1^, Jeremy Skepper^2^, Manuel Mayr^1^ and Catherine M. Shanahan^1^

^1^King's College London British Heart Foundation Centre of Excellence, Cardiovascular Division, The James Black Centre, London, United Kingdom; ^2^Multi-Imaging Centre, Department of Anatomy, Downing Street, Cambridge, United Kingdom

Email: Alexander.kapustin@kcl.ac.uk

Vascular calcification contributes to the high cardiovascular mortality in patients with renal failure and diabetes. We have shown previously that vascular calcification is initiated by the release of matrix vesicles (MV) enriched with the calcification inhibitor fetuin-A from vascular smooth muscle cells (VSMCs) yet little is known about MV biogenesis. We shown that fetuin-A is internalized by VSMC and delivered to the early endosomes and then transported to the late endosomal/lysosomal compartment where it either degraded or recycled in MVs. We found that MVs originate from multivesicular bodies and exhibited properties of exosomes. An inhibitor of neutral sphingomyelinphosphodiesterase 3 (SMPD3) and the exosomal production pathway, GW4869, blocked MV release and induced fetuin-A accumulation within VSMCs. Moreover, treatment of VSMCs in calcifying conditions induces SMPD3 expression and enhances exosome secretion in vitro and in vivo. Proteomic analysis of VSMC-derived exosomes revealed unique functional proteins involved in extracellular nucleotide catabolism. Furthermore, an AMP degrading enzyme, ecto-5’-nucleotidase (CD73), was enriched in VSMCs exosomes, and inhibition of CD73 activity efficiently blocked VSMCs calcification. In conclusion, VSMCs-derived exosomes are implicated in pathological calcification by transporting the calcification inhibitor fetuin-A and regulating extracellular adenine nucleotide metabolism.

Funded by: British Heart Foundation

**Tumor-derived exosomes and the matrix**

Sanyukta Rana, Mei Wu and Margot Zöller

Department of Tumor Cell Biology, University Hospital of Surgery, Heidelberg, Germany

Email: sanyukta.rana@gmail.de

Exosomes are important intercellular communicators, which does not only account for cells but also for the extracellular matrix (ECM), which is an important constituent of non-transformed tissues as well as tumors. We here asked whether exosomes derived from a metastatic rat tumor affect the organization of the extracellular matrix and whether this has consequences on tumor cell motility. Tumor-derived exosomes bind to individual components of the extracellular matrix, the preferential partner depending on the exosomes’ adhesion molecule profile such that high CD44 expression is accompanied by HA binding and high alpha6beta4 expression by laminin332 binding. Exosome binding to the ECM proceeds naturally during exosome delivery as well as upon contact of exosomes with distinct organ matrices. Importantly, exosomes, which are rich in proteases, modulate the extracellular matrix as demonstrated for collagen IV, laminin332 and hyaluronic acid (HA) degradation. By matrix degradation, exosomes also contribute to the delivery of matrix-incorporated chemokines and growth factors, such that tumor cells and stroma cells display increased motility in an exosome-modulated matrix. Taken together, the modulation of the extracellular matrix by exosomes may well be an important factor not only in premetastatic niche preparation including the recruitment of hematopoietic cells but also in physiological and pathological angiogenesis, leukocyte migration and, last not least, embryology.

Funded by: Deutsche Krebshilfe

### Coffee and Poster Session 2          15.00-15.30

### Symposium Session 6          15.30-17.00

#### 1. The Neural Network and the role of Extracellular Vesicle Transfer

**New aspects on the integrative capabilities of neural networks: the microvesicle-mediated transfer of GPCRs in cell cultures**

K. Fuxe^1^, M. Guescini^2^, G. Leo^3^, C. Carone^4^, S. Genedani^3^, M. Mantuano^2^, V. Stocchi^2^, D. Guidolin ^5^, R. De Caro R.^5^ and L.F. Agnati^4^

^1^Department of Neuroscience, Karolinska Institutet, Sweden; ^2^Department of Biomolecular Sciences, University of Urbino, Carlo Bo, Italy; ^3^Department of Biomedical Sciences, University of Modena and Reggio Emilia, Italy; ^4^IRCCS San Camillo, Venice, Italy; ^5^Department of Human Anatomy and Physiology, University of Padova, Italy

Email: kjell.fuxe@ki.se

The proposal on the existence of two main modes of intercellular communication in the central nervous system (CNS) was introduced in 1986 and called wiring transmission (WT) and volume transmission (VT). The major criterion for this classification was the different characteristics of the communication channels with physical boundaries well delimited in the case of WT (axons and their synapses; gap junctions) but not in the case of VT (the extracellular fluid-filled tortuous channels of the extracellular space and the cerebrospinal fluid-filled ventricular space and subarachnoidal space). Recently, the Roamer type of VT (a subclass of VT) has been introduced based on the release by source cells of microvesicles (MV) containing proteins, mtDNA and RNA. Such safe vesicular carriers flow in the extracellular fluid along energy gradients from the source to target cells. Western blot, TEM and gene expression analyses demonstrate that MVs can transport GPCRs, suggesting the MV involvement in cell-to-cell communication. In order to further demonstrate that GPCRs can be exchanged among cells by means of MVs, we created two populations of cells, the first transfected with D2R-CFP and the second with A2AR-YFP. These two types of cells were co-cultured, and acceptor photobleaching FRET analysis demonstrated cells positive to both D2R-CFP and A2AR-YFP. Furthermore, recipient cells preincubated for 24 h with A2AR positive MVs were treated with the adenosine A2A receptor agonist CGS-21680. The significant increase in cAMP accumulation clearly demonstrated that A2ARs were functionally competent in target cells. These findings demonstrate that A2A receptors capable of recognizing and decoding extracellular signals can be safely transferred via MVs from source to target cells. The possibility of pharmacological interventions on composition and/or release of microvesicles (i.e., the Roamer type of VT) should be considered as new therapeutic approaches capable of modulating the decoding capabilities of neurons and/or glial cells.

Funded by: IRCCS Hospital Lido VE; Regione Marche; M.M. Wallenberg Foundation, Karolinska Institutet Forskningsstiftelser 2010

**The role of neurotransmitter-triggered exosome transfer in reciprocal neuron-glia communication**

Eva-Maria Krämer-Albers, Carsten Frühbeis, Dominik Fröhlich and Wen Ping Kuo

Department of Molecular Cell Biology, Johannes Gutenberg University Mainz, Mainz, Germany

Email: alberse@uni-mainz.de

Myelination in the CNS is performed by oligodendrocytes (OL) and requires intimate communication between OL and neurons over lifetime. Independent of myelination, OL maintain axonal integrity by an undefined pathway of glial support. We studied the role of OL exosomes in neural cell communication and their potential to support neurons. Our findings demonstrate that neurons control OL exosome release by activity-dependent secretion of the neurotransmitter glutamate and stimulation of OL NMDA and AMPA receptors. Furthermore, neurons internalize the released exosomes by endocytosis. Exosome-mediated transfer of cre-recombinase resulted in target gene recombination in recipient neurons in culture and after injection of isolated exosomes into the brain. Thus, exosomal cargo can be functionally retrieved by the recipient neurons. Our results further indicate that OL exosomes are capable to protect neurons from starvation and metabolic/oxidative stress. Taken together, oligodendroglial exosomes participate in bidirectional neuron-glia communication and mediate the transfer of bioactive molecules from glia to neurons. We suggest that the signal-mediated exosome transfer from glia to neurons is implicated in neuroprotection and glial maintenance of axonal integrity.

Funded by: European Leukodystrophy Association, Studienstiftung Rheinland Pfalz, Focus Programme Translational Neuroscience JOGU Mainz

**Exosome-mediated transfer of luminal and cytosolic amyloids**

Lawrence Rajendran, Georgia Minakaki, Nino Jejelava, Garima Thakur and Jitin Bali

Systems & Cell Biology of Neurodegeneration, Division of Psychiatry Research, University of Zurich, Switzerland

Neurodegenerative diseases including Alzheimer's disease (AD), Parkinson's disease and prion diseases, are characterized by protein aggregation and deposition in specific brain regions. While the exact pathological significance of these aggregates remains to be conclusively resolved, the biology behind their formation is crucial to the understanding of the disease. Recent findings, on the release and spread of several amyloid proteins, both luminal and cytosolic, suggest a model where these proteins can be released from affected cells in the form of amyloid seeds and then reenter other cells and aid in the spread of the disease. How are these aggregates released from the cells? Once released, how do they form plaques and propagate in the aqueous extracellular space to gain access to their host counterparts? How are cytosolic proteins and amyloids released from cells? We propose that exosomes, endocytically derived nanovesicles, are a major way to shuttle cytosolic proteins and amyloids out of the cell. Release of amyloid β (Aβ) peptides on exosomes aid in the plaque formation. We provide evidence that amyloids involved in neurodegeneration such as Aβ are released via exosomes and that exosome-associated amyloids act as seeds for plaque formation.

**Neural stem cells sort protein and RNA cargoes for export with exosomes in response to inflammation**

Chiara Cossetti^1^, Tim R. Mercer^2^, Emanuele Alpi^3^, Tommaso Leonardi^1^, Nunzio Iraci^1^, Clara Alfaro-Cervello^1^, Marcel E. Dinger^2^, Sabine Dietmann^4^, Joanna Crawford^2^, Carla Caddeo^5^, Jose-Manuel Garcia-Verdugo^6^, Angela Bachi^3^, John S. Mattick^2^ and Stefano Pluchino^1^

^1^Cambridge Centre for Brain Repair, Department of Clinical Neurosciences, University of Cambridge, Cambridge, United Kingdom; ^2^Institute for Molecular Bioscience, University of Queensland, St Kucia, Queensland, Australia; ^3^Biomolecular Mass Spectrometry Unit, Division of Genetics and Cell Biology, San Raffaele Scientific Institute, Milano, Italy; ^4^Wellcome Trust Centre for Stem Cell Research and Department of Biochemistry, University of Cambridge, Cambridge, United Kingdom; ^5^Department of Pharmaco-Chemical Technology, University of Cagliari, Cagliari, Italy; ^6^Departamento de Neurobiologa Comparada, Instituto Cavanilles, Universidad de Valencia, Valencia, Spain

Email: spp24@cam.ac.uk

Neural stem/precursor cell (NPC) transplantation protects the central nervous system from inflammatory damage. This is in part due not only to cell-to-cell communication that occurs via diffusible factors but also to mechanisms involving the spread of information via extracellular membrane structures. Here, we show that NPCs secrete membrane vesicles primarily comprising exosomes and observe novel cytokine-regulated pathways that sort proteins and RNAs into these vesicles. Deep RNA sequencing reveals 35 exosomal microRNAs (miRs) and consistent depletion of miR* sequences within exosomes. All but six exosomal miRs undergo repression in response to inflammatory cytokines. We finally observe regulation of the cell cycle along with highly specific induction of Stat1 signaling response in recipient cells exposed to vesicles emitted under proinflammatory cytokines. Our study sheds new light on the mechanisms of transcellular information exchange and demonstrates that cytokine-regulated exosome signaling is an important pathway by which NPCs may mediate their protective capacities.

Funded by: This work has received support from the National Multiple Sclerosis Society (NMSS, partial grants RG-4001-A1 to S.P.), the Italian Multiple Sclerosis Association (AISM, grant 2010/R/31 to S.P. and 2010/R/31/B to C.C.), the Italian Ministry of Health (GR08-7 to S.P.), Wings for Life (grant XBAG/163 to S.P.), Banca Agricola Popolare di Ragusa (BAPR, unrestricted grant to S.P.), the European Research Council (ERC) under the ERC-2010-StG Grant agreement No. 260511-SEM_SEM; the Australian Research Council/University of Queensland co-sponsored Federation Fellowship (grant FF0561986 to J.S.M.), the Australian National Health and Medical Research Council Australia Fellowship (grant 631668 to J.S.M.) and the Career Development Award (CDA631542 to M.E.D.). C.C. has received a fellowship (SFRH/BD/15899/2005) from the Fundaao para a Cincia e a Tecnologia (FCT) and is now the recipient of the FISM fellowship 2010/R/31/B. T.R.M. is supported by the Human Frontiers Science Program, and M.E.D. is supported by Queensland Government Department of Employment, Economic Development and Innovation Smart Futures Fellowship.

**Transfer of genetic information between the hematopoietic system and the brain without cell fusion**

Stefan Momma, Kirsten Oesterwind, Sascha Keller, Maria Dams, Jadranka Macas, Britta Landfried, Karl H. Plate, Candan Depboylu, Günter Höglinger and Peter Altevogt

Institute of Neurology (Edinger Institute), Frankfurt University Medical School Frankfurt, Frankfurt, Germany; Tumor Immunology Program, German Cancer Research Center (DKFZ), Heidelberg, Germany; Department of Neurology, Philipps University, Marburg, Germany; German Center for Neurodegenerative Diseases e.V. (DZNE), Technical University Munich (TUM), Department for Translational Neurodegeneration, Munich, Germany

Email: stefan.momma@kgu.der

The contribution of hematopoietic cells to non-hematopoietic tissues that could be observed after bone marrow transplantation from genetically marked mice into lethally irradiated hosts is a phenomenon that has been discussed controversially in the recent past. Initially seen as evidence for an unexpected differentiation potential of hematopoietic stem cells, it was eventually demonstrated that the experimentally observed plasticity was largely attributable to cell fusion, rather than transdifferentiation. To what extent these cell fusion events occur under physiologically relevant conditions remains unclear. We now show that expressing Cre recombinase specifically in the hematopoietic compartment causes the induction of reporter gene expression in non-hematopoietic tissues without any evidence of cell fusion. In the brain, peripheral inflammation leads to a major increase in the number of recombined Purkinje neurons. We provide evidence that reporter expression is caused by intercellular transfer of Cre recombinase messenger RNA. Furthermore, biochemical analysis shows that Cre RNA is contained in secreted membrane vesicles, specifically in exosomes. Thus, while cell fusion occurs under specific experimental conditions, we suggest that lateral transfer of ribonucleotides by exosomes is the more physiological relevant mechanism. Importantly, these observations reveal the existence of previously unrecognized mechanisms to communicate RNA-based signals between the immune system and various organs, including the brain.

Funded by: Edinger Foundation, Deutsche Forschungsgemeinschaft (DFG)

#### 2. Biorepository, Proteome and Genome Databases for Extracellular Vesicles

**EVpedia: an integrated and comprehensive proteome and genome database for systemic analyses of extracellular vesicles**

Yong Song Gho^1^, Dae-Kyum Kim^1^, Byeongsoo Kang^2^, Yae-Jin Yoon^2^ and Daehee Hwang^2^

^1^Department of Life Science and ^2^School of Interdisciplinary Bioscience and Bioengineering, Pohang University of Science and Technology, Pohang, Republic of Korea

The secretion of extracellular vesicles is a universal cellular process occurring from simple organisms (archaea or Gram-negative and Gram-positive bacteria) to complex multicellular organisms, suggesting that this extracellular vesicle-mediated communication is an evolutionarily conserved. These extracellular vesicles are spherical bilayered proteolipids with an average diameter of 40–250 nm and enriched with various bioactive materials including proteins, lipids and genetic materials. Although proteomic analyses have allowed several thousands of vesicular proteins to be cataloged, there has been lack of resources providing vesicular proteomes from diverse types of cells and analytical tools for the comparative analyses. Here, we present EVpedia: an integrated and comprehensive proteome database of extracellular vesicles derived from archaea bacteria, and eukarya including human. EVpedia provides an array of tools, such as search and browse of vesicular proteins, comparison of vesicular datasets by ortholog identification, Gene Ontology enrichment analyses and network analyses of vesicular proteins. EVpedia further provides the databases of vesicular mRNAs, miRNAs and lipids. Thus, this free web-based EVpedia (http://evpedia.info) might serve as a useful community resource to trigger systemic and comprehensive studies of extracellular vesicles and to unveil fundamental roles of these complex extracellular organelles.

**Vesicelpedia: a compendium of nucleic acids, proteins and lipids identified in extracellular vesicles**

Richard J. Simpson and Suresh Mathivanan

Department of Biochemistry, La Trobe Institute for Molecular Science, La Trobe University, Melbourne, Victoria, Australia

Extracellular vesicles (EVs) are a class of membrane-bound organelles secreted by various cell types. EVs include (i) exosomes: 40–100 nm diameter membraneous vesicles of endocytic origin, (ii) ectosomes (also referred to as shedding microvesicles, SMVs): large membranous vesicles (50–1000 nm diameter) that are shed directly from the plasma membrane (PM) and (iii) apoptotic blebs (50–5000 nm diameter): released by dying cells. Over the last 5 years, the field of EVs has witnessed tremendous growth (more than 3500 research articles published) mainly due to their purported role in intercellular signaling and possible source of disease biomarkers. Vesiclepedia (http://www.microvesicles.org) is a manually curated database of nucleic acids, proteins and lipids identified in EVs. The database catalogs information from both published and unpublished EV studies. The mode of EV purification and characterization, the biophysical and molecular properties are listed in the database, aiding biomedical scientists in assessing the quality of the preparation and the corresponding data obtained. Currently, Vesiclepedia contains information on 35, 264 protein entries, 18,718 mRNA entries and 1772 miRNA entries that were obtained from 311 EV studies.

**A bioinformatic system for globally collaborative extacellular vesicle science**

William E. Butler, Bob Carter, Sarada Sivaraman, Lin Dan Zhu and Fred Hochberg

Massachusetts General Hospital, Boston, MA, USA; Lifeformatica, Boston, MA, USA; University San Diego Medical School, San Diego, CA, USA

Email: wbutler@partners.org

The Richard Floor Biorepository supports collaborative studies of microvesicular RNA and proteins found in human fluid and tissue specimens. We created an informatic system that offers responsibility for the entire scientific specimen lifecycle: demographic and clinical patient data; molecular classification; specimen management; and queries of analytic data. This informatic system currently tracks 4442 specimens drawn from 605 subjects.

The system provides bar code tracking of specimen aliquots, of aliquot fractionation and of the distribution of aliquots to test specific hypotheses including shipment tracking. Subject anonymization is protected by the use of synthetic identifiers and by data partitioning. There is geotracing of aliquot location from macro- to microlevels. At the macrolevel, there is display of aliquot containers on earth maps. At the microlevel, the system tracks aliquot locations within geometric models of storage containers.

We propose to work with ISEV to make this informatic system available for international correlative collaborations. The informatic system is designed to grow and adapt to evolving definitions and technologies within microvesicle science. This system's graphical user interface (GUI) and data storage and retrieval methods are data and metadata driven to accommodate to these scientific changes. It leverages where appropriate external validated ontologies including Foundational Model of Anatomy, HUGO, NCI Metathesaurus and SNOMED. The program employs software and hardware architectures known to scale globally, draws on open source technologies and is readily accessible via secure web. It is bundled in a Google Apps Domain to support collaboration via email, document sharing, calendars, task management and password and security management. This informatics system would support global collaborative ISEV efforts to provide correlation between clinical human diseases and exosome biology.

Funded by: The Floor Biorepostiory

**A collaborative biorepository supports exosomal studies of biofluids from patients with glioblastoma multilforme**

Fred H. Hochberg^1^, William Butler^1^, Will Curry^1^, Sarada Sivaraman^1^, Leonora Balaj^1^, Ryan Kim^2^, Lin Dan Zhu^1^, Johan Skog^1^, Linda M. Liau^3^, Eric T. Wong^4^, Steven Kalkanis^5^, Keith Flaherty^1^, John H. Sampson^6^ and Bob Carter^2^

^1^Neuroscience Center, Departments of Neurology, Neurosurgery and Radiology, Massachusetts General Hospital and Harvard Medical School, Boston, MA, USA; ^2^Division of Neurological Surgery, UCSD School of Medicine, San Diego, CA, USA; ^3^Department of Neurosurgery, UCLA, Los angeles, CA, USA; ^4^Beth Israel Medical Center, New York, NY, USA; ^5^Henry Ford Hospital, Detroit, MI, USA; ^6^Duke University Medical Center, Durham, NC, USA

Email: fhochberg@partners.org

The efforts for creating a biorepository for exosome-based clinical studies started at MGH back in 2008 and have now been expanded to include several institutions in the United States. Our focus is biomarker development, and we currently collect serum, plasma, urine as well as CSF samples from patients with GBMs, melanomas, meningiomas and schwannomas, and we are preparing to expand this collection to a broader range of cancers such as lung, pancreas, breast, colon, etc. Samples obtained both just prior to initial surgical removal of tumor, and at sequential follow-up visits thereafter, are filtered, aliquoted and stored at −80°C. We use a bar-code system to accurately input each sample into the system and for downstream tracking. The system allows one to monitor the time of accession, track residual sample volume, sample storage location and sample distribution. Studies supported by this biorepository include EGFR and EGFRvIII detection in exosomal RNA (exoRNA) from serum, BEAMing PCR for CSF-exosome-derived mutant IDH1, examinations of de novo vs. secondary GBM, detection of novel oncogenes using microarray profiles, BRAF V600E in serum-derived exoRNA, as well as the exoRNA contribution of circulating lymphocytes and protein detection using a novel NMR technology. The BIOREPOSITORY synergizes 7 (MGH, UCSD, Henry Ford, Amsterdam, Duke, UCLA and Beth Israel) collaborating institutions, by providing anonymized clinical, radiographic and molecular pathologic data while fulfilling IRB and ethical requirements. Multitiered access to the database is provided within firewalls and with error safeguards. Sample annotation is linked to hypotheses to be tested and supports clinical correlation of exoRNA findings. Clinical information includes over 40 fields, e.g., diffusion character of MRI lesions or EGFR tissue amplification that might correlate with EGFRvlll expression in sera. We provide this collaboration as a model for ISEV and the MCV community. To date, nearly 4500 samples have been obtained from over 600 glioma and metastatic cancer patients in addition to controls. Samples are available, upon petition, for ancillary studies performed by collaborators. We will present the Biorepository structure, sample distribution and SOPs, which will serve as a model for similar collaborations by exoRNA scientists.

Funded by: Brain Tumor Funders' Collaborative

#### 3. Role of Extracellular Vesicles in Pregnancy

**Do exosomes contribute to successful establishment of pregnancy?**

York Hunt Ng, Harmeet Singh, Cassandra Hincks and Lois A. Salamonsen

Prince Henry's Institute of Medical Research, Monash Medical Centre, Clayton, Victoria, Australia

Infertility is increasing worldwide with 1%of births in developed countries now achieved by assisted reproductive technologies. Embryo implantation into the womb is a critical step in establishment of pregnancy and requires close synchrony of development between embryo and the lining of the womb (endometrium). This is properly prepared (receptive) only briefly in each menstrual cycle. Inability of the endometrium to produce appropriate signals is a major cause for female infertility and IVF failure. Only some of the molecular events contributing to the implantation microenvironment are known. We hypothesize that exosomes released from receptive endometrium into the intrauterine environment contribute to implantation success: disturbance of this release or alterations in exosome contents may result in infertility. Our aim is to determine whether exosomes are released into the uterine cavity by receptive endometrium, their regulation and their function in fertile women. We have localized exosomal markers (CD9, CD63 and CD81) in human endometrial epithelium/cells using immunohistochemistry/immunofluorescence and shown the presence of these markers also in crude exosomal fractions from endometrial epithelial cell culture medium using fluorescence activated cell sorting (FACS). Studies to determine the regulation and function of exosomes in endometrial receptivity are still in progress. We believe this study will provide targets for diagnosis and treatment of women with infertility problems as well as increasing our understanding of the mechanisms underlying this critical process.

**Placental exosome-mediated apoptosis in human pregnancy**

Eva Delin^1^, Ann-Christin Stenqvist^1^, Olga Nagaeva^1^, Magnus Strand^2^, Lennart Kjellgren^2^, Vladimir Baranov^1^ and Lucia Mincheva-Nilsson^1^

^1^Departments of Clinical Microbiology/Clinical Immunology; ^2^Obstetrics and Gynecology, Umeå University, Umeå, Sweden

Email: Eva.Dehlin@climi.umu.se

The mammalian fetus is a challenge to the immune system and its survival is associated with the placenta and its role as an immunomodulatory organ. Several mechanisms for immune privilege of the semiallogeneic fetus have been described. To elucidate the role of apoptosis in fetal tolerance, we investigated the expression of three major proapoptotic molecules, Fas ligand (FasL), TNF-related apoptosis-inducing ligand (TRAIL) and programmed death ligand 1 (PD-L1), in human placenta. We found that all three molecules are constitutively transcribed and expressed by the syncytiotrophoblast. Exosomes, isolated from placental explant cultures expressed FasL, TRAIL and PD-L1 on their surface. Interestingly, Western blot experiments revealed that the placental exosomes carried FasL and TRAIL in their biologically active trimeric form. In the presence of isolated placental exosomes, activated PBMCs showed signs of apoptosis as assessed by electron microscopy. Furthermore, functional experiments revealed that the apoptosis was enhanced in a dose-dependent manner. It has been shown that membranal rather than soluble FasL is associated with induction of apoptosis in activated Fas-expressing cells. Our results suggest that constitutive exosome-mediated secretion of FasL, TRAIL and PD-L1 ensures that an active membranal form of these molecules is provided at the feto-maternal interface and might be a mechanism for elimination of activated maternal immune cells contributing to the immune privilege of the fetus.

Funded by: Swedish Cancer Foundation, Umeå University Samlings Fond, Cancerforskningsfonden i Norrland, Spjutspetsforskningsfonden VLL

**Proteomic, miRNA, flowcytometric and NTA characterization of placental vesicles in preeclampsia**

D. Tannetta, R. Dragovic, C. Gardner, M. McKeen, E. Ballabio, C. Lawrie, B. Kessler, C. Redman and I. Sargent Nuffield

Departments of Obstetrics & Gynaecology, Medicine and Clinical Laboratory Science, University of Oxford, Untied Kingdom

Email: dionne.tannetta@obs-gyn.ox.ac.uk

The placental surface (syncytiotrophoblast) releases vesicles (STBM) into the maternal circulation in increased amounts in preeclampsia (PE) implicated in the systemic inflammatory response, endothelial dysfunction and coagulation system activation, which underlie this disorder. Better characterization of STBM from normal and PE placentas is essential to elucidate their role in PE pathophysiology. STBM were prepared from placentas (normal term: *n*=8 and PE: *n*=8) by perfusion, isolated by ultracentrifugation and stored at −80C. MudPIT (multidimensional protein identification technology) and Affymetrix GeneChipR miRNA Arrays were used to derive the proteome and miRNA expression profiles. STBM were labeled with a membrane dye (maleimide; vesicle marker) and antibodies to placental alkaline phosphatase (placental vesicle marker), endoglin and VEGF receptor-1 for 4 color flow cytometry (4CFC). Finally, nanosight tracking analysis (NTA) was used to determine the size distribution of vesicles. Using MudPIT, 538 PE STBM unique, 604 normal STBM unique and 1421 proteins common to both preparations were found. Preliminary analysis indicated the presence of alarmins and exosomal, immunoregulatory and antiangiogenic proteins. Several proteins raised in the circulation in PE were also elevated in PE STBM. miRNA analysis revealed the presence of 569 miRNAs; 9 significantly upregulated (2-fold cut-off) and 14 downregulated in PE STBM. Five placental specific miRNAs were also detected. By unsupervised cluster analysis, PE STBM had a distinct miRNA expression profile. A selection of miRNAs was also validated by TaqMan qRT-PCR. 4CFC showed significantly lower endoglin (*p <*0.05) expression on PE STBM. Finally, using NTA, PE placentas were shown to release STBM of a larger size (*p*<0.01), indicative of cellular demise. In conclusion, STBM contain a heterogeneous population of vesicles that convey a large repertoire of placental proteins and miRNA into the maternal circulation. PE alters STBM size distribution and protein and miRNA profiles, suggesting a role in disease development and their potential use as biomarkers.

Funded by: The Wellcome Trust

**Measurement and characterization of cellular microvesicles and nanovesicles in pregnancy and preeclampsia using nanoparticle tracking analysis, ELISA and flow cytometry**

Rebecca Dragovic, Dionne Tannetta, Jennifer Southcombe, Christopher Redman and Ian Sargent

Email: rebecca.dragovic@obs-gyn.ox.ac.uk

Excessive release of syncytiotrophoblast vesicles (STBM) from the placenta into the maternal circulation may cause the systemic inflammatory response, endothelial dysfunction and activation of the coagulation system, which characterize preeclampsia (PE). Other cell types including platelets, leukocytes, red blood cells (RBC) and endothelium may in turn be activated to release cellular vesicles, which exacerbate the disease. We have used nanoparticle tracking analysis (NTA), ELISA and multicolor flow cytometry in parallel to enumerate and phenotype STBM, together with the other vesicle types in pregnancy plasma to determine their potential as biomarkers for PE. Vesicles were measured in ultracentrifuge pellets of platelet free plasma (PFP) from non-pregnant (NonP), normal pregnant (NormP) and PE women. Flow cytometry (BD LSRII) showed that the majority of vesicles (90%) in the PFP pellets labeled positive with the cell membrane marker Bio-Maleimide, demonstrating that the pellet was enriched with cellular vesicles. No difference in total vesicle (<1 m) number between all three groups was detected using flow cytometry (detects vesicles = 290 nm), whereas NTA revealed smaller vesicles (50–300 nm) in significantly elevated numbers in NormP and PE women compared to NonP controls. Flow cytometry and ELISA using the antisyncytiotrophoblast antibody NDOG2 showed that STBM levels were significantly elevated in NormP and PE women compared to NonP, but there was no difference between NormP and PE women. Multicolor flow cytometry showed that the majority of plasma vesicles were derived from platelets (CD41), followed by RBC vesicles (CD235a/b) and leukocyte vesicles (HLA Class-1); but no endothelial vesicles (CD146) were detected. The percentage of platelet vesicles was significantly decreased in PE vs. NonP controls whereas the percentage of leukocyte vesicles was significantly increased, consistent with activation of the inflammatory and coagulation systems in PE. Percentages of RBC vesicles were also significantly elevated in NormP and PE women compared to NonP controls. These data show differences in the composition of plasma vesicles between pregnant and NonP women, demonstrating that pregnancy affects vesicle release. Their use as biomarkers for PE continues to be explored.

Funded by: Wellcome Trust Programme Grant and by the Oxford Partnership Comprehensive Biomedical Research Centre with funding from the Department of Health's NIHR Biomedical Research Centres funding scheme.

**Secretion of oocyte exosomes appears CD9 and CD81 tetraspanins dependant**

J.P. Wolf, A. Benammar, A. Ziyyat and V. Barraud-Lange

Inserm U1016, University Paris Descartes, France

Email: jean-philippe.wolf@cch.aphp.fr

Spermatozoa leaving the testis are immotile and unable to fertilize the oocyte. During their way through male and female genital tracts, they interact with epididymosomes, prostatosomes and tubal epithelium. Any time, they acquire new or modulate preexisting functions. When the fertilizing spermatozoon enters the perivitellin space (PVS), it interacts with oocyte exosomes. This interaction may have important functions in triggering the sperm fusion ability. On the other hand, CD9 deleted oocytes present a dramatic decrease of fusion ability, which can be rescued by overexpression of CD81 tetraspanin. Deletion of CD81 decreases oocyte fusogenic ability, while the double CD9 CD81 knockout renders oocytes totally refractory to fusion. This suggests that these tetraspanins are partially redundant or complementary and that the presence of either one or the other is mandatory for fusion ability. Furthermore, sperm-oocyte fusion takes place in the microvillar region of the egg membrane. CD9 deletion dramatically modifies microvilli number and size. Hence, we investigated the role of both tetraspanines on exosome secretion and microvilli morphology. Oocytes being isolated in their zona pellucid, we used TEM to assess a semiquantitative appreciation of exosomes secretion and microvilli sizes. Density of microvilli decreases from 4.7 per μm in WT GV oocytes to 2.5, 1.3 and 0.7 in CD81, CD9 and double knockout respectively. Microvilli length is not dramatically modified but their width is increased from 77±19.2 to 140±55,8, 116±49.6* and168±69.7*, respectively. Preliminary data show that exosomes-like vesicles are secreted during oocyte maturation since there is none in VG stage WT oocytes. There is 1 exosome per m of membrane in CD9 knockout and 10 times less in CD81 and double knockout oocyte. In conclusion, microvilli morphology and exosome secretion seems to depend on the presence of any of these two tetraspanins as well as oocyte fusing ability.

Funded by: ANR Grant PIRIBIO Intergame 2009

**Plasma membrane vesicles are a rich source of potential biomarkers for preeclampsia**

Kok Hian Tan^1^, Soon Sim Tan^2^, Yanni Xu^1^, Newman Siu Kwan Sze^3^ and Sai Kiang Lim^2,4^

^1^KK Women's and Children's Hospital, Singapore; ^2^Institute of Medical Biology, A*STAR, Singapore; ^3^School of Biological Sciences, Nanyang Technological University, Singapore; ^4^Department of Surgery, YLL School of Medicine, NUS, Singapore

Email: tan.kok.hian@kkh.com.sg

*Aim*: To use circulating membrane vesicles to identify robust plasma biomarkers for preeclampsia. Plasma biomarkers of diseases are usually present in low abundance, and some of these proteins are sequestered in circulating membrane vesicles. We hypothesised that circulating membrane vesicles are good sources of plasma biomarkers for preeclampsia. *Methodology*: We have developed proprietary technologies to isolate two mutually exclusive populations of plasma membrane vesicles from pooled plasma of preeclampsia pregnant (3rd trimester) women and matched controls. The proteome of these two populations of plasma membrane vesicles were analyzed by mass spectrometry. *Results*: Each membrane vesicle population was isolated from 600 l pooled plasma to produce sufficient proteins for analysis by mass spectrometry. More than 99% of plasma albumin and immunoglobulins were also depleted. A total of 581 unique gene products were identified in the two microparticles isolated from plasma and 352 were present in both microparticle populations; 155 were present in only one population and 74 were present in the other population. There were 254 proteins were present at the same level (<2-fold) in both microparticle populations of preeclampsia pregnant (3rd trimester) women and matched controls leaving 327 proteins with > 2-fold differences in either one or both microparticle populations of preeclampsia patients versus their matched controls. *Conclusions*: The identification of an unprecedented number of low-abundant plasma proteins using a small volume of plasma supported our hypothesis that circulating membrane vesicles are good sources of plasma biomarkers of preclampsia. The 327 proteins that are elevated or reduced by 2-fold or more in preeclampsia patients versus their matched controls are potential candidate biomarkers and are currently undergoing validation studies.

Funded by: National Medical Research Council, Singapore

### All Posters in Session 2

#### 1. Protein Detection in Health and Disease

**1. Comparative meta-analysis of proteomic data on extracellular vesicle subsets**

T.G. Szabó, P. Misják, B. Aradi, B. György, M. Pásztói, Zs. Pál, V. László, É. Pállinger, E. Pap, Á. Kittel, Gy. Nagy, K. Taylor-Szabó, A. Falus and E.I. Buzás

Department of Genetics, Cell- and Immunobiology, Semmelweis University, Budapest, Hungary

Email: szabogtamas@gmail.com

*Background*: Distinct populations of extracellular vesicles (EVs) have clearly different size, morphology and subcellular origin. Our aim was to study whether distinct types of EVs were also characterized by differential proteomic composition. *Materials and methods*: Protein contents of EV subsets were analyzed on the basis of data accessible in the ExoCarta Database or extractible from PubMed. Vesicles isolated by density gradient ultracentrifugation were regarded as exosomes and vesicles sedimented at 15,000–20,500g as microvesicles. A third type oft of EV preparation, isolated with 100,000–200,000g was also studied. Proteomic analysis was done with Ingenuity Pathway Analysis (IPA) software. *Results*: Filtering the literature yielded in 6 exosomal, 5 microvesicular and 6 “high-speed isolation” mass spectrometry studies, with 738, 1299 and 872 proteins extractable, respectively. A substantial number of nuclear proteins was also found in EVs, especially in MVs, besides cytoplasmic and membrane proteins. Functional proteomic analysis by the IPA software suggests that cell movement, cell morphology and cell death are strongly associated with vesicular proteins. *Conclusions*: The overlap between the protein content of different types of EVs suggest the existence of a common “vesicular proteome.” Differences between EV proteomes, on the other hand, may also be important to note.Funded by: OTKA K 73247, NK 84043 and Baross Gábor (REG-KM-09-1-2009-0010)

**2. Lyophilized exosomes as calibration standards for exosome proteomic studies**

N. Zarovni, V. Könt, A.L. Kubo, K. Talpsepp, T. Oja and A. Chiesi

HansaBioMed OU, Tallinn, Estonia

Email: natasa.zarovni@hansabiomed.eu

Currently employed methods for exosome isolation and quantification vary considerably, and so do the data reported in literature on exosome yields and molecular composition. From the perspective of exosome proteomics, as a vehicle for the discovery and employment of clinically relevant biomarkers, there is a need of defining reliable and stable standards to allow accurate identification and quantification of exosome associated markers in biological samples. The key aim of this study was the evaluation of lyophilized exosomes purified from cell supernatants, human plasma and urine with optimized protocols based on differential centrifugation and microfiltration. These proved to be stable upon a long-term storage at + 4°C and suitable as reference samples for multiple applications (FACS, WB and ELISA), including the use as calibration standards for quantitative comparison of different biological samples. ELISA was confirmed as a method of choice for the analysis of exosomes from biological fluids allowing linear quantification and normalization of samples and displaying increased sensitivity in detection of exosome-associated proteins compared to conventionally used methods (protein content measurements, FACS and WB). ELISA assays providing specific exosome capture (ExoTESTTM) permitted quantification of exosomes and exosomal proteins in a sample independently on the prior fractionation of the sample and/or stringency of the exosome purification method used.

Funded by: EAS Grant EU29269

**3. Transcriptomics profiling of hepatic extracellular microvesicles**

Juan M. Falcon-Perez^1^, Esperanza Gonzalez^1^, Ana M. Aransay^2^ and Felix Royo^1^

^1^Metabolomics Unit, CIC bioGUNE,CIBERehd, Ikerkasque Foundation, Spain; ^2^Genome Analysis Platform, CIC bioGUNE, Spain

Email: jfalcon@cicbiogune.es

Hepatocytes and hepatocyte-like cell lines release extracellular microvesicles (e.g., exosomes) containing proteins and nucleic acids that eventually will be captured and managed by other far or close localized cells. Previously, our group has shown the proteomic composition and the changes that occur in the microvesicles content when cells are exposed to different stimulus or under pathological conditions. In the present study, we address the mRNA composition of the secreted vesicles, comparing the transcriptomics profile of primary mature hepatocytes with a precursor hepatocyte cell line referred as MLP29. Adult hepatocytes release microvesicles enriched in transcripts that encodes for metabolic proteins, meanwhile in MLP29 cargo, cell cycle and cell proliferation related genes are more represented in concordance with their origin. Moreover, we have identified exosomes as the microvesicles that content those transcripts. The enrichment of certain transcripts in exosomes versus cells suggests a selective mechanism to determine the specific exosomes content that is shared by other hepatic cell lines (e.g., AML12). Finally, the comparison with other published non-hepatic microvesicles transcriptomes reveals a subset of common transcripts enriched in oxidative phosphorylation and mitochondrial dysfunction related genes.

Funded by: Instituto de Salud Carlos III (FIS, PS09/00526), SPAIN CIC bioGUNE, SPAIN CIBERehd, SPAIN IKERBASQUE Foundation, Spain

**4. Quantitative LC-MSE proteomics of hepatocyte-derived microvesicles reveals non-invasive candidate markers for liver injury**

E. Gonzalez^1^, E. Rodríguez-Suárez^2^, J. Conde-Vancells^1^, C. Hughes^3^, A. Rudella^4^, F. Royo^1^, F. Elortza^2^, J.P.C. Vissers^3^ and J.M. Falcón-Pérez^1^

^1^Metabolomic Unit, CIC bioGUNE, Spain; ^2^Proteomic Unit, CIC bioGUNE, CIBERehd, ProteRed, Spain; ^3^Waters Corporation, MS Technologies Center, Manchester, Untied Kingdom; ^4^Waters Corporation, Vimodrone, Italy

Email: egonzalez@cicbiogune.es

Extracellular microvesicles are small natural membrane vesicles released by a wide variety of cell types into the environment that have been detected in blood and urine samples, awaking great interest to identify tissue-specific non-invasive disease biomarkers. In this work, we have achieved a thorough qualitative and quantitative analysis of the proteome of hepatocyte-derived microvesicles by using two complementary strategies. We combined 1D-LC and DIA to perform label-free quantitation in a first scrutiny. Furthermore, proteins were separated by 2D-LC and analyzed by DIA to perform and extensive and complementary protein identification in a second analysis. This integrated proteomic approach has provided a comprehensive qualitative analysis of proteins present in extracellular microvesicles derived from primary hepatocytes. In addition, by applying label-free quantitation proteomics on this easy-to-manage in vitro model combined with well-known liver toxins, we were able to detect significant deregulation of an elevated number of proteins from these vesicles after galN and LPS treatments, identifying candidate markers for liver injury. Finally, we have validated in vivo some of these markers supporting that quantitative proteomics on extracellular microvesicles using in vitro systems is a suitable strategy to unravel non-invasive candidate biomarkers.

Funded by: Fondo de Investigaciones Sanitarias (Institute of Health Carlos III, PS09/00526); Centro de Investigación Biomédica en Red en el Área temática de Enfermedades Hepáticas y Digestivas (CIBERehd); CIC bioGUNE.

**5. Rab 5 as exosomal marker: localization and applications**

N. Zarovni^1^, V. Könt^1^, A.L. Kubo^1^, T. Oja^1^, K. Talpsepp^1^, A. Chiesi^1^, I.C. Ghiran^2^, M. Ericsson^2^, F.W. Luscinskas^2^, C.M. Bonebreak^2^, F.M. Heravi^2^ and W.P. Kuo^2^

^1^HansaBioMed OU, Tallinn, Estonia; ^2^Harvard University, Boston, MA, USA

Email: natasa.zarovni@hansabiomed.eu

Exosome biogenesis is a multistep process that comprised a plethora of proteins ensuring specificity and efficiency of vesicular targeting and fusion with cognate membranes. Rab5 is a member of conserved family of small GTPases, recognized as a critical regulator of clatrin-mediated endocytosis and early endosomal fusion events. Despite these documented roles, its own trafficking and subcellular distribution throughout the endomembrane system is not fully elucidated. Rab5 is reported to be associated to a cytosolic part of plasma membrane and, to a larger extent, to the endosomal compartment where it is dynamically recruited from cytosol in a GTP-GDP switch-dependent manner and is interacting with different partners residing on a local membrane (such as its effectors PI3P, EEA1, rabamozin, etc). Traditionally referred as to an early endosome marker, Rab5 has not been so far unambiguously linked to MVB formation and exosome release but rather suggested to mediate degradation directed cargo transport through endosmal system to lysosomes. In the present study, we report persistent association of Rab5 to exosomal fractions derived from a wide range of biological samples including cultured cancer cells, human plasma and urine. The presence of Rab5 on the exosomes isolated from all analyzed sources is ascertained by WB, FACS and ELISA, in addition to high-resolution fluorescence microscopy, and transmission electron microscopy. Independent and complementary approaches show that Rab5 is also localized on the extraluminal side of the exosomal membrane, constituting thus a (novel) marker for exosome identification and immunocapturing applications.

Funded by: EAS Grant EU29269

**6. Comparative analysis of proteomic composition of thymus-derived extracellular vesicle populations**

P. Misják^1^, L. Turiák^2^, G.T. Szabó^1^, B. Aradi^1^, K. Pálóczi^1^, O. Ozahonocs^2^, L. Drahos^2^, A. Kittel^3^, A. Falus^1^, K. Vékey^2^ and E.I. Buzás^1^

^1^Semmelweis University, DGCI, Budapest, Hungary; ^2^Chemical Research Center of the Hungarian Academy of Sciences, Budapest; Hungary; ^3^Hungarian Academy of Sciences, Budapest, Hungary

Email: petra.misjak@gmail.com

*Background*: Thymus is a primary immune organ that plays a key role in shaping the peripheral T cell repertoire. *Goals*: To compare the protein composition of thymus-derived apoptotic bodies and microvesicles in BALB/c mice. *Methods*: Extracellular vesicle populations were isolated by differential centrifugation and size filtration. The resulting vesicle populations were analyzed by electron microscopy and nano-HPLC/MS(MS), and the functions of proteins were studied by Ingenuity Pathway Analysis.

*Results*: In apoptotic bodies and microvesicles, we have identified 142 and 195 proteins, respectively. A striking overlap was detected between the proteomic compositions of the two subcellular structures. Identified proteins included autoantigens implicated in autoimmune diseases, key regulators of T-cell activation, molecules involved in known immune functions or in leukocyte rolling and transendothelial migration. *Conclusions*: The presence and abundance of proteins with high immunological relevance within thymocyte-derived apoptotic bodies and microvesicles raise the possibility that these subcellular structures may substantially modulate T-cell maturation processes within the thymus.

Funded by: OTKA, 84043

**7. Proteomic characterization of crude and affinity-purified exosomes from pancreatic cancer cells**

Susanne Klein-Scory^1^, Mahnaz Moradian Tehrani^2^, Uwe Warnken^2^, Martina Schnölzer^2^, Wolff Schmiegel^1^ and Irmgard Schwarte-Waldhoff^1^

^1^Department of Medicine, Knappschaftskrankenhaus, Ruhr-University, Bochum, Germany; ^2^Functional Proteome Analysis, German Cancer Research Center, Heidelberg, Germany

Email: susanne.klein-scory@rub.de

Detailed knowledge about the protein composition of tumor-derived exosomes may reveal insights into their putative functions and potential applications as diagnostic tools. We here present protein catalogues from secretomes, crude and affinity-purified exosome preparations derived from human pancreatic carcinoma cell lines. These indicate known and novel markers of pancreatic secretomes and exosomes and their comparison allows to differentiate between real exosomal proteins and not exosome-specific components including proteasomal and ribosomal proteins. On the other hand, a number of membrane proteins, glycoproteins, small GTP binding proteins, transporters and others were enriched in affinity-purified exosomes, among them many with prominent roles in carcinogenesis as modulators of the extracellular matrix, intercellular communication and tumor-stroma interaction.

Funded by: This work was supported by the BMBF, NGFN Plus program

**8. Exosomes—potential regulators and biomarkers of prostate cancer progression**

Claire Corcoran^1^, Sweta Rani^1^, Keith O'Brien^1^, Amanda O'Neill^2^, Maria Prencipe^2^, John Crown^3^, William Watson^2^ and Lorraine O'Driscoll^1^

^1^School of Pharmacy & Pharmaceutical Sciences, Trinity College, Dublin; ^2^UCD School of Medicine and Medical Science, University College, Dublin; ^3^Molecular Therapeutics for Cancer, Ireland

Email: corcorcl@tcd.ie

Hormone-refractory prostate cancer treatment remains hindered by inevitable progression of resistance to first-line treatment with docetaxel. To help determine the complexity of this problem, in vitro cell line models of docetaxel resistance were established representative of the clinical situation. This study aimed to (i) isolate exosomes from medium conditioned by these cell line models and investigate their effects on motility, invasion, proliferation and docetaxel resistance of secondary cells; (ii) perform a proof-of-principle translational investigation of the clinical relevance of exosomes isolated from prostate cancer serum; and (iii) perform microRNA profiling on cells and their corresponding exosomes to determine intracellular and extracellular biomarkers predictive of response to docetaxel. Exosomes expelled from DU145 docetaxel-resistant variant (DU145RD) conferred docetaxel resistance to both DU145 and 22Rv1 cells, which may be partly due to exosomal MDR-1/P-gp transfer. Furthermore, exosomes from prostate cancer patient sera increased cell proliferation and invasion, compared with age-matched controls. Finally, miRNA profiling studies revealed a panel of miRNAs common between cells and exosomes that may offer potential as biomarkers predictive of response to docetaxel. Our in vitro observations and preliminary clinical studies indicate that exosomes play an important role in prostate cancer and may offer potential as vehicles containing predictive biomarkers and new therapeutic targets.

Funded by: Science Foundation Ireland's Strategic Research Cluster award to Molecular Therapecutics for Cancer Ireland (08/SRC/B1410)

**9. Proteomic profiling of exosomes reveals novel candidate markers for prostate cancer**

Diederick Duijvesz, Marina A. Gritsenko, A. Marije Hoogland, Mirella S. Vredenbregt-van den Berg, Kristin E. Burnum, Geert J.L.H. van Leenders, Rob Willemsen, Theo Luider, Ljiljana Pasa-Tolic and Guido Jenster

Departments of Urology, Pathology, Neurology, and Genetics, Erasmus Medical Center, Rotterdam, The Netherlands; Pacific Northwest National Laboratory, Environmental Molecular Sciences Laboratory (EMSL), Richland, WA, USA

Email: d.duijvesz@erasmusmc.nl

*Introduction*: Current markers for prostate cancer (PCa), such as PSA, lack specificity. Therefore, novel biomarkers are needed for optimal diagnoses and prognosis. Profiling of PCa exosomes could reveal new markers for this malignancy. *Materials and methods*: Exosomes from two non-PCa cell lines (PNT2C2 and RWPE-1) and 2 PCa cell lines were isolated (PC346C and VCaP) by ultracentrifugation. Proteomic analyses utilized a NanoLC-LTQ-Orbitrap mass spectrometry, operated in tandem MS (MS/MS) mode using the Accurate Mass and Time (AMT) tag approach. Selected differential exosomal protein expression was validated by Western blotting. A tissue microarray, containing 481 PCa samples, was used to correlate candidate markers with clinicopathological information and follow-up data. *Results*: Proteomic characterization of the exosomes resulted in the identification of 263 proteins. Statistical analyses revealed 52 proteins differently expressed between PCa and control cells: 9 of them more abundant in PCa. Western blotting confirmed a higher expression of FASN and XPO1 and PDCD6IP (ALIX) in exosomes derived from PCa cell lines. Tissue microarray, containing 481 PCa patient samples, showed strong correlation of higher Gleason scores and local recurrence with increased cytoplasmic XPO1 staining (*p <*0.001). *Conclusions*: Proteins in exosomes make a clear subdivision between benign and malignant origin. Validation of protein expression showed a preferential expression of PDCD6IP, FASN and XPO1 in PCa exosomes. Cytoplasmic XPO1 staining is strongly correlated with higher Gleason scores and local recurrence.

Funded by: ErasmusMC grant

**10. Circulating exosomal Hsp60 as a new marker of colon cancer**

Francesco Cappello^1,2^, Claudia Campanella^1,2^, Antonella Marino Gammazza^1,2^, Anna Merendino^1^, Sabrina David^1^, Fabio Bucchieri^1,2^, Alessandro Pitruzzella^1,2^, Giosalba Burgio^3^, Davide Corona^3^, Nunzia Scibetta^4^, Giovanni Tomasello^5^, Carmelo Sciumè^5^, Adriana Lena^5^, Giuseppe Cicero^5^, Everly Conway de Macario^6^, Alberto JL Macario^2,6^ and Giovanni Zummo^1^

^1^Dipartimento di Biomedicina Sperimentale e Neuroscienze Cliniche, UniPA, Palermo, Italy; ^2^Istituto Euro-Mediterraneo di Scienza e Tecnologia, Palermo, Italy; ^3^Dipartimento di Scienze e Tecnologie Molecolari e Biomolecolari, UniPA, Palermo, Italy; ^4^UOC di Anatomia Patologica, ARNAS Civico, Palermo, Italy; ^5^Dipartimento di Discipline Chirurgiche e Oncologiche, UniPA, Palermo, Italy; ^6^Department of Microbiology and Immunology, School of Medicine, University of Maryland at Baltimore, and IMET, Baltimore, MD, USA

Email: francapp@hotmail.com

In the past, we showed thatHsp60 levels are increased in some types of tumor, e.g., colon cancer, and that the chaperonin is secreted by tumor cells, in vitro, via the exosomal pathway. The aim of the present work was to verify if Hsp60 is released by colon cancer cells also in vivo, via exosomes. We studied tissues and blood from 20 colon cancer patients 1 day before and 4 weeks after tumor removal by surgery and from 20 normal controls. We investigated presence of Hsp60 in tissues by immunohistochemistry and immunoelectron microscopy (IEM), in serum by ELISA and in exosomes from blood by Western blotting. We found higher levels of Hsp60 in tissue and serum from patients compared to controls. Hsp60 serum levels decreased significantly after tumor removal. Hsp60 was also present in the exosomes from blood before surgery but it was undetectable after it. IEM showed Hsp60 in the plasma membrane of tumor but not of normal cells. These results support the notion that Hsp60 is secreted by colon cancer cells, in vivo, via the exosomal pathway. The results also indicate that serum and circulating exosomal Hsp60 have potential as useful markers to follow-up colon cancer patients after tumor ablation.

Funded by: University of Palermo, Palermo, Italy; Istituto Euro-Mediterraneo di Scienza e Tecnologia, Palermo, Italy

**11. Diagnostic potential of ELISA-based detection of exosome-associated melanoma markers**

A.L Kubo, K. Talpsepp, T. Oja, N. Zarovni and A. Chiesi A

HansaBioMed OU, Tallinn, Estonia

Email: antonio.chiesi@hansabiomed.eu

High incidence and recurrence rate of melanoma underlines a demand for diagnostic assays for sensitive detection of tumor markers in accessible biofluids such as plasma, which would be suitable for non-invasive screening and early detection of disease and/or recurrence in a risk population. Exosomes are privileged peripheral sources of proteins that are mirroring the proteomic profile of a parent (tumor) cell. We report the analysis of a panel of tumor markers (over)expressed on circulating exosomes from melanoma patients, using an ELISA method that permits the isolation of exosomes of specific origin prior to determining their biosignature (ExoTESTTM). Used capture and detection antibodies can recognize exosome-specific markers (Rab5, CD63 and CD9), melanocyte-specific proteins (MelanA, MCAM, Tyrosinase) and tumor markers (CEACAM, CD44). These markers were assessed in WB and FACS on exosomes purified from model cells and plasma of healthy donors (HD) and selected melanoma patients prior to ELISA screening of a pilot sample of 15 patients and 30 HD. Our results reveal the potential of the used ELISA platform to accurately distinguish cancer samples by detecting the enrichment of tumor tissue specific marker-expressing subpopulation in overall circulating exosomes. The enrichment ratio calculated by normalization of distinct marker signal to that obtained by measuring CD63 on captured exosomes was for CD44+, 0.85–1.28 (1.03±0.12) vs. 0.67 in HD pool sample; for MelanA + 0.83–1.54 (1.14±0.26) vs 0.82; for tyrosinase + 1.11–1.30 (1.22±0.07) vs 0.84; for CEACAM + 0.84–1.36 (1.1±0.37) vs 0.63. We are currently testing extended panel of markers in enlarged cohort of melanoma patients and HDs as to assess the overall resolution power and prognostic value of a multimarker ELISA assay for melanoma detection.

Funded by: EAS EU29269

**12. A highly sensitive ELISA for detection and characterization of (prostate cancer) exosomes**

D. Duijvesz, N. Naimi, M. Peltola, C.Y.L. Versluis, T.M. Luider, K.S.I. Pettersson and G. Jenster

Department of Urology and Neurology, Erasmus Medical Center Rotterdam, The Netherlands; Department of Diagnostic Technologies and Applications, University of Turku, Turku, Finland

Email: d.duijvesz@erasmusmc.nl

*Introduction*: Exosomes contain proteins and RNAs, which are specific to the tissue from which they are derived, and, therefore, can be considered as a source for disease-specific markers. To translate exosomes as biomarkers for prostate cancer to the daily clinic, we developed an improved exosome-based sandwich ELISA. *Material and methods*: Exosomes were isolated by ultracentrifugation and filter steps from different cell lines (PNT2C2, DU145N, VCaP and PC346C), urine from healthy women, men and PCa-diagnosed men. A sandwich-ELISA was developed by using streptavidine plates, biotinylated capture antibodies (CD9 or CD63) and fluorescent europium-labeled detection antibodies (CD9 or CD63). Measurements were performed with a time-resolved fluorescence (TRF) microplate reader (Viktor 1420, Perkin Elmer). *Results*: Exosomes from all samples can be detected by our ELISA, in a concentration-dependent manner. The lowest measurable limit in cell lines was 10 ng of exosomal proteins and in patient samples 50 ng. VCaP- and PC346C-derived exosomes have higher fluorescence intensities compared to exosomes from the PNT2C2, DU145N cell line and patient samples when the same amount of exosomal proteins was added. Aspecific binding and freezing do not influence the ELISA. *Conclusions*: With our ELISA, exosomes from all samples can be detected with high sensitivity. Therefore, an ELISA with biotinylated and europium-labeled antibodies could be useful for the development of an exosome-based assay that can be translated into the clinic. Nevertheless, more and prostate-specific antibodies have to be tested to find the most optimal combination that could preferably outperform current markers.

Funded by: ErasmusMC Grant

**13. Binding of heat shock proteins and extracellular vesicle-like protein complexes from subcellular fractions using venceremin peptides**

Steven G. Griffiths^1^, Scott E. Lewis^2^, Adrian Culf^1^, Miroslava Cuperlovic-Culf^3^, David Barnet^1^, Erin Legace^4^, Daniel Leger^1^ and Rodney J. Ouellette^1^

^1^Atlantic Cancer Research Institute, Moncton, NB, Canada; ^2^New England Peptide, Gardner, MA, USA; ^3^National Research Council of Canada, Institute for Information Technology, Moncton, NB, Canada; ^4^AV Nackawic, NB, Canada

Email: steveg@canceratl.ca

Sharply defined biomarkers of early cancer detection, staging and promotion of recurrence free survival are urgently required. Although of frustrating heterogeneity, some common features of cancer include overactive glycolysis and overproduction of heat shock protein (HSP) networks. As neither feature is shared by healthy cells, we theorized the capture of native HSP complexes would yield cancer-specific biomarkers. To this end, we developed cationic peptides referred to as venceremins (Vns) that were avid binders of individual HSPs by counter migration during isoelectric focusing (IEF). Counter migration of Vn peptides with cancer cell fractions also formed cognate bands during IEF. When analyzed by mass spectrometry, it was determined that the Vn peptides captured a variety of HSPs as well as enzymes typical of cancer metabolism, cytoskeletal cancer biomarkers and a variety of other proteins. Observing these data as a whole, the proteins bore similarity to the contents of extracellular (EVs) vesicles released by cancer [Exocarta 2012; (1)]. It is thus possible Vn peptides captured prereleased “ready to go” EVs. The concept and reagents introduced here may present unique opportunities for biomarker collection from small volumes of biopsies and aspirates.

Funded by: Atlantic Canada Opportunities Agency

**14. Quantification and profiling of exosomes in human plasma using protein microarray**

Malene Jørgensen^1^, Rikke Bæk^1^, Evo Søndergaard^1^, Shona Pedersen^2^, Søren Risom Kristensen^2^ and Kim Varming^1^

^1^Department of Clinical Immunology, Aalborg Hospital, Aarhus University Hospital, Aalborg, Denmark; ^2^Department of Clinical Biochemistry, Aalborg Hospital, Aarhus University Hospital, Aalborg, Denmark

Email: maljoe@rn.dk

Exosomes are endosome-derived vesicles between 40 and 100 nm in diameter that are secreted by many cell types including epithelial cells in both normal and neoplastic states. The quantity and molecular composition of exosomes shed from cancer cells differs considerably from those shed by normal cells. In this study, the novel multiplexed platform of protein microarray is used for quantifying and profiling exosomes in plasma obtained from colon cancer patients and healthy controls. This assay is based on the antibody capture of microvesicles and subsequent detection of the captured microvesicles by biotin labeled antitetraspanin antibodies (CD9, CD63 and CD81). Antibodies used to capture these targeted exosome biomarkers are specific to membrane proteins for: exosomes in general (CD63 and HLA-ABC), exosomes from colon cancer cells (EGFR and STAMP1), lung cancer (SPD, SPA and osteopontin) and tumor-associated exosomes [EpCam, CD276 (B7H3) and 10 other known tumor-associated antigens and cancer-testis antigens]. The method was validated using the nanoparticle tracking analysis (NTA) with NanoSight before and after capture by protein microarray. The validation was performed both as total quantification of all microvesicles and with fluorescence labeling of the exosomes with the detections antibodies (CD9, CD63 and CD81).

Funded by: The Danish Donorfoundation

**15. ExoScreen as a novel ultrasensitive detection technology of serum exosomes**

Yusuke Yoshioka^1–3^, Nobuyoshi Kosaka^1^ and Takahiro Ochiya^1^

^1^Division of Molecular and Cellular Medicine, National Cancer Center Research Institute, Chuo-ku, Tokyo, Japan; ^2^Integrative Bioscience and Biomedical Engineering, Graduate School of Science and Engineering, Waseda University, Shinjuku, Tokyo, Japan; ^3^Japan Society for the Promotion of Science (JSPS), Japan

Email: yyoshiok@ncc.go.jp

Detection of molecular features of tumor-derived in exosomes may provide a novel approach to early detection, diagnosis and prognosis determination of tumors. Complicated handling in exosome isolation, however, has limited the feasibility of this approach in clinical setting. The aim of this study is thus to provide a method to directly detect and quantify exosomes in human serum. We have developed a sandwich immunoassay named ExoScreen, which is based on AlphaLISA technique. In this assay, exosomes are captured by two antibodies modified in distinct ways. One is biotinylated antitetraspanin antibody, and the other is antitetraspanin antibody conjugated to AlphaLISA acceptor beads. We evaluated this assay system with exosomes isolated from culture supernatants of prostate cancer cell line PC3 using anti-CD63 antibody and anti-CD9 antibody. ExoScreen successfully provided a quantification of these exosomes: CD63 + or CD9 + exosomes were detectable in a dose-dependent manner. Moreover, ExoScreen allowed sensitive detection of exosomes in human serum from healthy donors and prostate cancer patients using anti-CD63 antibody and anti-CD9 antibody. These results suggest that ExoScreen system enable us to detect cancer specific antigens loaded on circulating exosomes and, thus, provide a novel cancer biomarker.

Funded by: Project for Development of Innovative Research on Cancer Therapeutics

**16. TM9SF4 level of expression on exosomes as new marker of malignancy in human cancer**

F. Lozupone^1^, V. Kont^2^, A. Logozzi, K. Talpsepp^2^, T. Oja^2^, A.L. Kubo, N. Zarovni^2^, A. Chiesi^2^ and S. Fais^1^

^1^Istituto Superiore di Sanità, Roma, Italy; ^2^HansaBioMed OÜ, Tallinn, Estonia

Email: francesco.lozupone@iss.it

TM9SF4 belongs to transmembrane 9 superfamily (TM9), a family of proteins characterized by a large variable hydrophilic *N*-terminal domain followed by nine transmembrane domains. We have recently shown that: (i) TM9SF4 is detectable is human melanomas, while it is undetectable in healthy skin tissues; (ii) in melanoma cells TM9SF4 localizes in early endosomal compartment and it is involved in the regulation of vesicles pH. TM9SF4 expression on vesicles of early-endosomal origin allowed us to hypothesize that this protein could be included in the tumor exosomes, forming, through a process of multivesicular fusion. Presence of TM9SF4 on exosomes was evaluated by RT-PCR and Western blot analysis of exosomes deriving from different tumor cell lines highly expressing this protein. We observed that TM9SF4 is highly detectable on exosomes deriving from tumor cells while undetectable on in vitro cultured macrophages as well as healthy donors’ body fluids deriving exosomes. In order to quantify exosomal TM9SF4 level of expression in cancer patients, we are setting up a Double-sandwich ELISA test (Exotest™) for quantitative and qualitative analysis of exosomes. With this assay, we analyzed exosomes deriving from different tumor cell lines and from tumor patient plasma samples. Preliminary results clearly suggest that TM9SF4 is highly expressed on exosomes deriving from melanoma and ovary carcinoma patients, while TM9SF4 values in exosomes collected from healthy donors’ plasma are in the same range of background. In conclusion, we propose TM9SF4 detection on exosomes as a new marker of malignancy. Exotest-based analysis of plasma exosomes carrying tumor-associated antigens may represent a novel tool for clinical management of cancer patients.

Funded by: Italian Association for Cancer Research Hansabiomed (Hansabiomed research activities)

**17. Comparative analysis of the surface glycosylation profiles of urine exosomes (uExo) and Tamm Horsfall protein (THP) by lectin microarray and flow cytometry**

Anja Krüger^1,2^, Jared Q. Gerlach^1^, Shirley A. Hanley^2^, Susan Gallolgy^2^, Marie C. Hogan^3^, Christopher J. Ward^3^, Lokesh Joshi^1^ and Matthew D. Griffin^2^

^1^Glycoscience Group and ^2^Regenerative Medicine Institute (REMEDI), NCBES, National University of Ireland, Galway, Ireland; ^3^Department of Medicine, Division of Nephrology & Hypertension, Mayo Clinic, Rochester, MN, USA

Email: anja-krueger@gmx.net

Limited information is available on glycosylation of uExo-associated proteins and its significance for kidney diseases. We characterized surface glycosylation of uExo prepared by two distinct isolation methods and compared the results to purified THP using lectin-based technologies. Exo were enriched from healthy urine by ultracentrifugation or spin-concentration and labeled with PKH26^®^. THP was purified by salt precipitation and labeled with AF647^®^. Microarrays consisting of 43 lectins were incubated with labeled samples, washed and imaged at 5 µm resolution. Carbohydrate-mediated binding was verified based on ≥ 50% inhibition with haptenic sugars. Unsupervised clustering of normalized data was performed using HCE^®^ 3.0 software. For flow cytometry (FCM), 5 µg of uExo or THP was adsorbed to 4 µm aldehyde/sulfate latex beads, which were sequentially incubated with biotinylated lectins and streptavidin-PE-Cy7 and analyzed using a BD FACSCanto^®^ cytometer. Unsupervised clustering of median-normalized lectin microarray data showed that the carbohydrate-mediated profile of uExo was distinct from that of THP for both isolation methods. The lectin array profile of THP indicated abundant sialylated, complex N-glycans evidenced by strong responses with GlcNAc- and sialic acid-binding lectins. In contrast, uExo bound a broader range of lectins indicating greater diversity of surface carbohydrates. FCM also demonstrated differential lectin affinities for uExo and THP. The uExo profile showed diverse lectin binding with potential components of N- and O-glycan structures in contrast to Gal- and GlcNAc-specific lectin preferences for THP. We conclude that uExo from healthy urine samples share a common lectin signature, which is distinct from THP regardless of uExo purification technique. Surface glycosylation of uExo-associated proteins is complex and represents a novel means to interrogate renal cell status in health and disease.

Funded by: Health Research Board of Ireland under grant number HRA_HSR/2010/63

**18. Identification of extracellular vesicles in urine**

Muthuvel Jayachandran^1^, Virginia M. Miller^1^, Andrew D. Rule^2^ and John C. Lieske^2,3^

^1^Departments of Surgery, Physiology and Biomedical Engineering, ^2^Internal Medicine, and ^3^Laboratory Medicine and Pathology, Mayo Clinic, Rochester, MN, USA

Email: jaya.m@mayo.edu

Recent studies implicate extracellular vesicles (exosomes and microvesicles) as potential novel biomarkers for variety of disease. This study was designed to establish a reproducible, simple, rapid and cost-effective assay to characterize urine borne vesicles using digital flow cytometry. Two 24-hour urine samples 3-months apart were prospectively obtained from persons presenting with kidney stone episodes along with age (18–75 years) and gender-matched controls from the general population. A significant number of phosphatidylserine (annexin-V) positive microvesicles (MV) were observed in both control and kidney stone-former urine. Urine pH did not vary depending upon group, gender, age or sample collection (1st vs 2nd visit) and did not affect the number of MV present. The number of annexin-V positive MV did not correlate with concentration (mOsm) of the urine [control (*n*=26), *r* ^2^=0.013, stone formers (*n*=57), *r* ^2^=0.071, respectively]. Centrifugation of urine from 1000g to 3000g for more than 5 minutes lowered (*p*<0.05) the number of annexin-V positive MV. The number of annexin-V positive MV in uncentrifuged urine was not associated with age. However, kidney stone formers excreted significantly higher numbers of annexin-V positive MV compared to age-matched controls. Urinary annexin-V positive MV lack established markers of blood borne MV, suggesting a different cellular origin from within the urological system. Studies are ongoing to identify populations of urinary extracellular vesicles that can serve as diagnostic and prognostic biomarkers of kidney and other disease.

Funded by: Mayo Clinic O'Brian Urology center, Pilot grant DK83007

**19. Marcks peptide as a probe to target microvesicles**

Leslie Morton, Jonel Saludes, Lida Beninson, Edwin Chapman, Monika Fleshner and Hang Yin

University of Colorado, Boulder, CO, USA

Email: Leslie.Morton@colorado.edu/morton.leslie3@gmail.com

Myristoylated alanine-rich C-kinase substrate (MARCKS) is an 87-kDa, intracellular protein whose functions involve sequestering phosphatidylinositol 4,5-bisphosphate (PIP2) in the inner membrane leaflet and regulating phospholipase C signaling. MARCKS binds to calmodulin (CaM) in the presence of Ca^2+^ using its effector domain (ED, a.a. 151–175), thereby reversing its association to the membrane. The ED of MARCKS also recognizes negatively charged PS-enriched inner leaflet of the plasma membrane, consistent with exosome/microvesicle membrane outer leaflet, with an approximate micromolar affinity. We focused on the ED of MARCKS in our search for curvature-sensing peptides due to the following rationales. First, it is reported that the membrane binding by MARCKS is driven by electrostatic interactions between PS and the Lys residues of MARCKS, while the secondary structure is not important. This suggests that the flexible truncated ED may retain the membrane binding ability. Second, curved membranes are known to present more defects due to the asymmetric stretching on the phospholipid bilayer. The MARCKS ED was speculated to bind to membranes by inserting its aromatic side chains into the lipid bilayers. It is conceivable that a peptide corresponding to the ED of MARCKS may stabilize the highly curved membrane by filling in the defects with these aromatic side chains. Therefore, we set out to use the MARCKS ED peptide to target different lipid vesicle sizes with the aim of laying the groundwork in the development of an efficient diagnostic tool for metastatic cancer screening. Recently, microvesicle shedding was shown as a direct indication of cancer metastasis in B16 mouse melanoma cells and thus has the potential as a biomarker. Microvesicles (Ø∼0.1–1 µm) and exosomes (Ø∼30–100 nm), collectively called as MVs, are highly curved lipid particles that shed into body fluids (e.g., blood, urine and ascitic fluid). These membrane-enclosed sacs are released from the cell, during which lipid rearrangement results in the expulsion, and thus enrichment, of negatively charged phosphatidylserine (PS) on the outer leaflet. Since these lipid nanoparticles are difficult to detect using conventional methods (e.g., optical microscopy and FACS), we aim to develop a new method of detecting these potential metastatic cancer biomarkers. Naturally occurring proteins [e.g., Bin-Amphiphysin-Rvs (BAR) domains] are known to sense membrane curvature, but these molecules are not optimal for large-scale productions. Herein, we report a novel strategy of using the MARCKS peptide to selectively bind to highly curved membranes as a chemical probe for MVs.

Funded by: Edwin Chapman thanks the National Institute of Health (MH061876) for support. Monika Fleshner thanks the National Science Foundation (NSF IOS 1022451) for support. Leslie Morton was supported by the Signaling and Cellular Regulation National Institute of Health training grant (T32 GM008759) and is currently supported by the F31 Ruth L. Kirschstein National Institute of Health fellowship award (1F31CA165349-01).

#### 2. RNA Detection in Health and Disease

**20. Healthy and cancerous serum RNA profiling by the novel RNA extraction reagent and highly sensitive DNA chip “3D-Gene”**

Hideo Akiyama, Makiko Ichikawa, Hiroko Sudo, Yoji Ueda and Satoko Takizawa

Toray Industries, Inc., Kanagawa, Japan

Email: Hideo_Akiyama@nts.toray.co.jp

Proteins, metabolites and DNA are already known as components of serum or plasma biomarkers; however, RNA has not been a strong biomarker candidate because of its instability. Exosomes that are small vesicles secreted by various cells are recently reported to play important roles in intercellular communications by transferring proteins, DNA and also RNA to distant cells through circulatory system. Surprisingly, the exosomal RNA in serum preserves its integrity and thus holds a potential to be a new blood biomarkers. In this report, we show the exhaustive analysis of miRNA and mRNA in serum by DNA chip for the highly purified RNA extracted with a novel reagent. Serum contains various types of nucleic acids, mainly small RNA, mRNA and also short DNA fragment. We suppose that the contamination of DNA to the extracted RNA often causes a discrepancy between the DNA chip analysis and qRT-PCR validation. The novel reagent was able to extract RNA from serum without contamination of short DNA fragment, resulting in better RNA quantification and decreasing DNA-related noise outputs. Using this novel RNA extraction reagent and the highly sensitive DNA chip “3D-Gene,” we analyzed healthy and cancerous serum miRNA profiles. Over 500 miRNAs are detected in healthy and cancerous sera reproducibly, and some miRNAs were detected specifically in cancerous sera, such as breast, gastric and cervix cancers. In addition, we detected over 19,000 mRNAs from amplified RNA, which were similarly extracted from healthy serum and detected by 3D-Gene. This indicates that not only serum miRNA but also serum mRNA have a potential capability to be a biomarker.

Funded by: Toray Industries, Inc.

**21. Plasma exosomes can deliver exogenous siRNA to human mononuclear blood cells**

Jessica Wahlgren, Tanya De L. Karlson, Mikael Brisslert, Forugh Vaziri Sani, Esbjörn Telemo, Per Sunnerhagen and Hadi Valadi

Department of Rheumatology and Inflammation Research, Sahlgrenska Academy, University of Gothenburg, Gothenburg, Sweden

Email: hadi.valadi@gu.se

Despite the promise of RNAi and its potential, e.g., for use in cancer therapy, several technical obstacles must first be overcome. The major hurdle of RNAi-based therapeutics is to deliver nucleic acids across the cell's plasma membrane. Our study demonstrates that exosome vesicles derived from humans can deliver short interfering RNA (siRNA) to human mononuclear blood cells. Exosomes are nanosized vesicles of endocytic origin that are involved in cell-to-cell communication, i.e., antigen presentation, tolerance development and shuttle RNA (mainly mRNA and microRNA). In this study, two different strategies were examined for introducing exogenous nucleic acids into various kinds of human exosomes. Electroporation was superior for transferring siRNA across the exosomal membrane and use them as gene-delivery-vectors (GDV). Having discovered the optimized electroporation parameters, siRNA against mitogen-activated protein kinase 1 (MAPK1) was introduced into exosomes originated from peripheral blood of healthy donors (plasma exosomes). The vesicles effectively delivered the administered siRNA into monocytes and lymphocytes, causing selective gene silencing of MAPK1.

Funded by: Swedish Research Council, the Swedish Heart and Lung foundation, and Torsten och Ragnar Söderbergs foundations.

**22. Exosomes derived from blood and cell culture media: isolation, characterization of the RNA content and utilization as siRNA delivery vehicles**

Alexander Vlassov, Tim Barta, Mu Li, Emily Zeringer, Marie Gonzalez, Jeoffrey Schageman, Susan Magdaleno and Robert Setterquist

Rick Conrad Life Technologies, Austin, TX, USA

Email: sasha.vlassov@lifetech.com

Exosomes are small (30–120 nm) vesicles containing nucleic acid and protein cargo secreted by all cell types in culture. They are found in abundance in body fluids including blood, saliva, urine and breast milk. There is an exponentially growing interest to studying the function exosomes and utilizing them for diagnostics development. At the moment, the mechanism of exosome formation, the makeup of the “cargo,” biological pathways and resulting functions are poorly understood. There is an urgent need to further our understanding of microvesicles, and critical to this is the development of reagents and tools for their isolation, characterization and manipulation. We will present data on (1) isolation of exosomes from blood serum/plasma and cell culture media, using modified ultracentrifugation and other protocols; (2) initial characterization of the RNA content using next-generation sequencing; (3) early data on utilization of exosomes as vehicles for siRNA delivery in vitro.

Funded by: Life Technologies

**23. Identification and characterization of RNA-binding proteins (RBP) involved in the transport of esRNA into the exosomes**

L. Statello, J. Wahlgren, M. Purrello and H. Valadi

Department of Gian Filippo Ingrassia, Unità di BioMedicina Molecolare Genomica e dei Sistemi Complessi, Genetica, Biologia Computazionale, University of Catania, Italy; Department of Rheumatology and Inflammation Research, University of Gothenburg, Gothenburg, Sweden

Email: luisastatello@gmail.com

The general goal of this project is to study the RNA transfer mechanism that occurs between cells via exosomes. We have shown that many cells send RNA messages to each other by packaging them into exosome vesicles. These vesicles contain approximately 4000 gene transcript (mRNA) and 500 microRNA many of which are unique and exist dominantly only in exosomes. Since the esRNA (exosomal shuttle RNA) are not randomly packed into the exosomes, we hypothesis that there are a packaging mechanism of RNA (intracellular RNA-transport-proteins) that coordinate the transfer of esRNA into the exosomes during their biosynthesis, before their secretion into the extracellular environment. The proteins (RNA-binding proteins, RBPs) that are involved in the packaging mechanism of esRNA into exosome vesicles are still unknown. Thus, the aim of this project is to identify and characterize the proteins that are involved in (a) the translocation of esRNA into exosomes from their donor cells, (b) the maintenance of esRNA inside the exosomes, and (c) posttranscriptional control of esRNA in recipient cells.

Funded by: Swedish Research Council, the Swedish Heart and Lung foundation, and Torsten och Ragnar Söderbergs foundations

**24. Comparison of exosome isolation methods for miRNA profiling**

Vaidla^1^, A.-L. Kubo^2^, N. Zarovni^2^, A. Chiesi^2^, A. Salumets^1,3^ and M. Peters^1,3^

^1^Competence Center on Reproductive Medicine and Biology, Estonia; ^2^HansaBioMed, Ltd, Estonia; ^3^Department of Obstetrics and Gynecology, University of Tartu, Estonia

Email: kadri.vaidla@gmail.com

*Background*: Exosomes are small membrane-bound vesicles secreted by most cell types. Exosomes contain various functional proteins, mRNAs and microRNAs that could be of use for diagnostic and therapeutic purposes. Currently, a standard method for exosome isolation is ultracentrifugation, which is time consuming and labour intensive. ExoQuick Exosome Precipitation Solution is an easy-to-use commercial kit for exosome isolation without ultracentrifugation step. *Aim of the study*: The aim of our study was to determine whether exosomal miRNA expression profile depends on exosome isolation method. *Methods*: Exosomes were isolated from blood serum of healthy individuals by ultracentrifugation and ExoQuick methods. The expression profile of 378 miRNAs was determined by real-time PCR using Exiqon miRCURY LNA microRNA Human panel I assays. *Results*: The overall expression profile of exosomal miRNAs was similar regardless of the isolation method used. However, some miRNAs showed altered levels of expression, which indicates that observed miRNA profile is dependent upon the isolation method. *Conclusions*: Both exosome isolation methods are suitable for miRNA profiling. Some variances in the miRNA expression levels were revealed by the comparison of two methods, but further studies are needed to find out the biological significance of these differences.

Funded by: Enterprise Estonia, Grant No. EU30200

**25. Quantitation of tissue-specific target gene modulation using circulating RNA**

Alfica Sehgal, Qingmin Chen, Dinah W.Y. Sah and David Bumcrot

Alnylam Pharmaceuticals, Inc., Cambridge, MA, USA

Email: asehgal@alnylam.com

Pharmacologic target gene modulation is the primary objective for RNA antagonist strategies, including small interfering RNA (siRNA), microRNA (miRNA) therapeutics and antisense oligonucleotide therapeutics. In clinical applications, monitoring tissue-specific target mRNA modulation requires tissue procurement from patients that is highly limited in availability, if even justified. Here, we show that circulating RNAs encoding tissue-specific gene transcripts can be detected in biological fluids of humans and experimental animals. Surprisingly, we demonstrate that RNAi-mediated target gene silencing in the liver by systemic administration of siRNA results in quantitative reductions in serum mRNA levels, which closely corroborate the degree and kinetics of tissue mRNA silencing, including proof of the RNAi mechanism of action. Further, administration of an anti-miRNA oligonucleotide directed against a liver-specific miRNA was found to result in decreased levels of the miRNA in circulation. This technique was extended to a different tissue, where silencing of a brain-expressed mRNA was monitored and quantified in cerebrospinal fluid following intraparenchymal infusion of a specific siRNA. This non-invasive method for monitoring tissue-specific RNA modulation could greatly advance clinical development of gene therapy and RNA-based therapeutics.

Funded by: Alnylam Pharmaceuticals

**26. Role of aging-associated exosomes in vitro and in vivo**

Ayumi Nakamura, Megumi Okada, Nobuyuki Kosaka, Takahiro Ochiya and Hidetoshi Tahara

Department of Cellular and Molecular Biology, Graduate School of Biomedical Sciences, Hiroshima University, Hiroshima, Japan; Division of Molecular and Cellular Medicine, National Cancer Center Research Institute, Hiroshima, Japan

Email: toshi@hiroshima-u.ac.jp

Exosomes are 50–100 nm lipid bilayer membrane vesicles containing microRNAs, proteins and DNA secreted from various cells, providing intercellular communication both within and between cells. We postulated that exosomes may influence human aging and the onset of age associated diseases. To test this hypothesis, we first analyzed exosomes during in vitro aging (senescence) of human fibroblasts. To examine the secretion of exosomes during cellular senescence, cell-free conditioned mediums are collected from young and senescent fibroblasts and examined microRNAs expression contained in exosomes derived from young and senescent fibroblast. MicroRNA expression profiling was performed using TORAY 3D-Gene array, and sustained global alteration of miRNAs were seen in senescent derived exosomes. Recently, we identified senescent-associated microRNAs such as miR-22 that negatively regulates cell proliferation in normal and cancer cells. Interestingly, there is little senescent-associated miRNAs in senescent-derived exosomes. Surprisingly, senescent-derived exosomes affected cell morphology as well as cell growth. Furthermore, we also examined exosomes from human sera of healthy volunteers and found that microRNA expressions were drastically altered during in vivo aging. Considering these observations, we suppose that exosomal miRNAs will have a potential role in aging and age-related diseases.

Funded by: Grant-in-Aid for Scientific Research (KAKENHI), The Ministry of Education, Culture, Sports, Science and Technology (MEXT).

#### 3. The Neural Network

**27. Comparative proteomic analysis of the synaptoneurosome reveals synaptic proteins linked to prion infection**

S.A. Booth, S. Gushue, B. Abrenica, Y. Niu, J. Saba and R. Saba

Molecular PathoBiology Unit, Public Health Agency of Canada, Winnipeg, MB, Canada; Department of Medical Microbiology, University of Manitoba, Winnipeg, MB, Canada; Thermo Fisher Scientific, USA

Email: Stephanie.Booth@phac-aspc.gc.ca

Prion diseases such as bovine spongiform encephalopathy (BSE), scrapie and Creutzfeldt-Jakob disease are rare progressive neurodegenerative disorders. The causative agent is a prion, a transmissible protein that is able to induce abnormal folding of normal cellular prion proteins in the brain. In prion diseases, neurons ultimately undergo necrosis and apoptosis, yet the pathways that trigger the damage and dysfunction of nerve cells are as yet unidentified. We previously performed a high-throughput microarray screen for genes and miRNAs specifically altered in the hippocampal CA1 region of infected mice that we dissected by laser capture microdissection and determined a clear temporal genetic response to the challenge of replicating prions. The earliest transcriptional changes within neurons are of particular interest, and we identified a subset of genes dysregulated in CA1 neurons. Comparison with published studies revealed striking deregulation of genes coding for synaptic proteins, especially those involved in vesicle-mediated transport, synaptic transmission and synaptosomes. We then collected synaptoneurosomes from mice and profiled proteins by mass spectrometry and miRNAs by Agilent microarrays.

Synaptoneurosomes contain presynaptic vesicles and densely stained membranes representing the postsynaptic density, which we think are the location of the earliest pathological alterations in prion disease. Correlation with transcriptomic data from infected animals has resulted in the determination of a list of disease-altered proteins and miRNAs within synaptoneurosomes. The significance of these in relation to the molecular mechanism of prion-induced neurodegeneration and the design of novel therapeutics will be discussed.

Funded by: Public Health Agency of Canada PrioNet Canada

**28. Identification and profiling of exosome-derived miRNAs in human cerebrospinal fluid**

Akira Machida, Takuya Ohkubo, Hidehiro Mizusawa and Takanori Yokota

Department of Neurology and Neurological Science, Graduate School of Medicine, Tokyo Medical and Dental University, Japan

Email: akinuro@tmd.ac.jp

MicroRNAs (miRNA) are increasingly used as diagnostic and prognostic markers, and there have been some reports that miRNAs in cerebrospinal fluid (CSF) are useful tools in the neurological disorders such as primary central nervous system lymphoma, glioma and Alzheimer's disease. Recently, CSF has been revealed to contain exosomes by which miRNAs may be delivered. However, there is no standard method for normalization of CSF miRNAs in miRNA arrays nor internal control for miRNA qRT-PCR assays, despite the surge of interest in miRNA identification and quantification. For these purpose, we identified highly expressed miRNA candidates of normal human CSF exosomes. Furthermore, some of these miRNA are more specific for exosome than in fluid fraction of CSF. Our normalization method for the expression level of miRNAs in normal CSF might help searching for the specific miRNA markers in other CNS diseases.

**29. Schwann cell-derived exosomes promote axonal growth and regeneration**

M. Alejandra Lopez-Verrilli and Felipe A. Court

Millennium Nucleus in Regenerative Biology (MINREB), Catholic University of Chile and Neurounion Biomedical Foundation, Santiago, Chile

Email: alejanddra@gmail.com

We have previously shown that, in the peripheral nervous system, ribosomes are transferred from Schwann cells (SC) to axons, but the transfer mechanism was not investigated (1). It has been shown that exosomes mediate cellular communication by transferring proteins, mRNA and/or miRNA between cells. We propose that exosomes mediates macromolecular transfer between SCs and neurons; therefore, we investigated the functional relevance of this process during axonal growth and regeneration.

*Methods*: SC primary cultures were obtained from newborn rat sciatic nerves. Exosomes were purified from SC conditioning medium by ultracentrifugation and characterized by morphological and biochemical analysis. Exosome internalization in dorsal root ganglia (DRG) sensory neurons was observed using vital dyes, immunofluorescence and confocal microscopy, as well as by electron microscopy. Axonal growth and regeneration assays were performed in DRG in vitro and in rat crushed-sciatic nerves in vivo. *Results*: Exosome markers CD63, Hsc70, Tsg101 and flotillin were observed in exosomes secreted by SCs. Internalization of SC-derived exosomes by axons increased after 2 and 4 hours of incubation (1.3 + 0.2 and 4.9 + 0.6 internalization/mm^2^ of axonal area, respectively). Axonal growth increased after 4 days of SC-derived exosome treatment respect to non-treated neurons. When axons were cut, SC-derived exosomes—but not fibroblast-derived ones—increased axonal regeneration by 32% at 3 days-postinjury. Exosomes injection immediately after rat sciatic nerve crush, and daily thereafter, increased nerve regeneration in vivo as determined by functional tests and morphological analysis using growth-associated protein 43. *Discussion*: We demonstrate for the first time that SCs secrete exosomes and that these exosomes are internalized by axons. SC-derived exosomes increases axonal growth and axonal regeneration after injury in vitro and promoted axonal regeneration in peripheral nerves in vivo. Our results support the role of SC exosomes to maintain axonal integrity and to improve nerve repair after peripheral injury.

Funded by: FONDECYT No. 3110014, FONDECYT No. 1110987 and Millennium Nucleus P-07-011-F

**Reference**

1. Court FA, et al. S. J Neurosci. 2008 Oct 22;28(43):11024–9.

**30. The co-chaperone stress inducible protein 1 utilizes the endosomal system to be secreted in microvesicles by astrocytes**

Marcos V.S. Dias^1^, Glaucia N.M. Hajj^1^, Camila Arantes^1^, Marco A.M. Prado^2^ and Vilma R. Martins^1^

^1^International Center for Research and Education, A.C.Camargo Hospital, Brazil; ^2^Robarts Research Institute, University of Western Ontario, Canada

Email: msalles@cipe.accamargo.org.br

The co-chaperone stress inducible protein 1 (STI1) was characterized as a prion protein (PrPC) specific ligand. This interaction modulates a number of functions such as protection against cell death and neuritogenesis in hippocampal neurons. STI1 is abundantly expressed in the cytoplasm but binds PrPC outside of the cell membrane. STI1 can be secreted by astrocytes and tumor cells; however, no signal peptide was found within STI1 sequence and its mechanism of secretion is unknown. Ultracentrifugation protocols of conditioned medium (CM) from primary astrocyte cultures showed that STI1 can be sedimented after 1, 2 and 16 hours of centrifugation at 105,000g. These fractions are positive for several exosomal markers. In order to identify the pathways used by astrocytes to secrete STI1 co-transfection of STI1-GFP with the early endosomal protein mCherry-RAB5 or with the late-endossomal protein mCherry-RAB7 were performed in astrocytes. When STI1 was co-transfected with RAB7, TIRF analysis showed co-localization of both proteins in endosomes, suggesting that STI1 present in CM could be derived from the endosomal system. The AAA-ATPase Vps4 is critical for function of the multivesicular body sorting pathway, and the dominant negative VPS4A(E228Q) was used to test whether STI1 secretion depends on multivesicular body trafficking. Transfection of astrocytes with the DN pEGFP-VPS4A (E228Q) caused a significant reduction of STI1 secretion (about 68%), evaluated by ELISA in the CM, compared to cells transfected with the wild-type protein. In addition, confocal analysis demonstrated that STI1 is recruited to VPS4A-E228Q-induced enlarged endosomes. These results indicate that STI1 secretion requires transport through the endosomal system and depends on sorting into multivesicular bodies.

Funded by: Sao Paulo State Foundation (FAPESP), National Institute for Translational Neuroscience (INNT), PrioNet-Canada

**31. Vesicle-associated protein secretion from dystrophin-deficient myotubes**

Stephanie Duguez^1^, William Duddy^1^, Helen Johnston^1^, Marie Catherine Le Bihan^2^, Douglas Johnson^1^, Kristy J. Brown^1^, Gillian Butler-Browne^2^, Yetrib Hathout^1^ and Terence Partridge^1^

^1^Genetic medicine, Children's Hospital, Washington, DC, USA; ^2^Université Pierre et Marie Curie, Paris 6, Institut de Myologie, Paris, France

Email: stephanie.duguez@upmc.fr

Lack of dystrophin, as in Duchenne muscular dystrophy (DMD) and its mdx murine equivalent, renders myofibres susceptible to recurrent bouts of segmental necrosis. It is thought that resulting inflammation drives progressive accumulation of fibrous and fatty connective tissue, with regeneration gradually becoming ineffectual as satellite cells reach proliferative exhaustion. Although dystrophin is suggested to link the intra- and extracellular cytokeleton network, deficiency in this function is not enough to explain the onset or progression of DMD. The present study shows that mdx myotubes secrete twice as much protein as wild type. An aberrant vesicle trafficking in mdx myotubes was suggested by analysis of the secretome and proteome profiles. Mdx myotubes possessed LAMP-1 positive vesicles containing myosin light chain-1—a marker oversecreted in mdx myotubes—as well as secreted vesicles with aberrant densities. Secretome and proteome profiles were rescued when mdx myotubes were treated with morpholinos to rescue dystrophin expression. These results suggest that increased protein secretion is due to a dysregulation of vesicle trafficking. Furthermore, LAMP-1 accumulation under the plasma membrane in 16-day-old mdx muscles is consistent with the idea that an aberrant vesicle trafficking could occur in vivo as a prepathogenic process. We hypothesize that the export of proteins through vesicles occurs before the onset of the pathological cascade described above, continues thereafter and contributes to the pathophysiology of DMD.

Funded by: IDDRC 1P30HD40677 and NCMRR 2R24HD050846 NIH grants and by funding from the Foundation to Eradicate Duchenne and from Wellstone Center, U54HD053177, W81XWH-05-1-0616, and by the European Community's Seventh Framework Programme project MYOAGE (contract 223576), and the ANR Genopath IN-A-FIB and the AFM (Association Française contre les Myopathies).

**32. In vivo gene delivery using microvesicle-associated AAV**

Johan Skog^1^, Casey A. Maguire^1^, Matheus Crommentuijn^1^, Xandra O. Breakefield^1,2^ and Bakhos A. Tannous^1^

^1^Department of Neurology, Massachusetts General Hospital, Boston, MA, USA; ^2^Department of Radiology, Massachusetts General Hospital, Boston, MA, USA

Email: johan.skog2@gmail.com

Obtaining tissue-restricted transgene expression after intravenous (i.v.) injection of AAV vectors is a challenging task, especially for the brain, as the majority of vector is taken up by the liver. Additionally, preexisting antibodies against AAV can remove vector from the circulation. Other studies have shown that association of virus vectors with nanoparticles, such as microbubbles and cationic liposomes, can alter the vector biodistribution to preferred sites. Another nanoparticle that may offer utility to the field of viral vector gene delivery is microvesicles (MV). MV are small (∼50–200 nm in diameter) membrane-limited structures naturally secreted by many cell types. We have recently shown that MV-associated AAV vectors (MV-AAV) can deliver genes more efficiently on a genome copy per cell basis than AAV vectors alone using cultured cells. In the current study, we are using the MV-AAV for targeted gene delivery to the brain after i.v. injection in mice. We produced standard standard iodixanol gradient purified MV-free AAV9 encoding firefly luciferase (Fluc) or MV-AAV9 encoding Fluc produced in 293T cells and injected these vectors i.v. into nude mice. MV-AAV displayed an approximately 3-fold lower transduction of liver compared to standard AAV vectors. Currently, we are investigating if MV-AAV can be targeted to the brain via the overexpression of specific ligands on the MV surface. Additionally, the antibody response to MV-AAV will be examined in vivo.

Funded by: American Brain Tumor Association National Institutes of Health

**33. C2C12 myoblasts and myotubes secreted specific exosomes-like vesicles involved in myogenesis**

Sophie Rome^1^, Alexis Forterre^1^, Audrey Jalabert^1^, Danty Emmanuelle^1^, Karim Chikh^1^, Elisabeth Errazuriz^2^, Mathieu Baudet^3^, Yoanne Coute^3^, Joffrey De Larichaudy^1^, Cécile Vors^1^, Michel Record^4^, Geloen Alain^1^, André Tchernof^5^, Etienne Lefai^1^ and Hubert Vidal^1^

^1^Laboratory CarMen (INSERM 1060, INRA 1235, INSA), University of Lyon, Faculté de Médecine Lyon-Sud, Chemin du Grand Revoyet, Oullins, France; ^2^Centre Commun d'Imagerie de Laënnec (CeCIL), SFR Santé Lyon-Est, University of Lyon, France; ^3^Étude de la Dynamique des Protéomes (EDyP), CEA, Grenoble, France; ^4^INSERM 563, Département « Lipoprotéines et Médiateurs Lipidiques », CPTP, Place du Dr Baylac, Hôpital Purpan, Toulouse, France; ^5^Endocrinology and Genomics, Laval University Medical Research Center, Quebec City, Quebec, Canada

Email: srome@univ-lyon1.fr

Exosomes represent a discrete population of nanometer-sized microvesicles formed in the endocytic compartments called multivesicular bodies (MVBs) during endosome maturation by inward budding of their limiting membrane. They are released from the cell into the microenvironment following the fusion of MVBs with the plasma membrane. Recent data indicated that exosomes might convey informations and signals not only between neighbor cells but also between distant tissues. During the last decade, it has been demonstrated that skeletal muscle secreted proteins that have important roles in intercellular communications. Thus, in this study, we characterized the exosomes released from skeletal muscle and we studied their biological functions during the myogenic process. We used a proteomic approach, combined with electronic microscopy, Western blot analysis and bioinformatic, to demonstrate that these vesicules not only have the classical properties of exosomes isolated from other cell type including components of the ESCRT machinery and of the MVBs and numerous tetraspanins but also contained specific muscle proteins. In addition, we found that their protein compositions differed in relation with the muscle cell differentiation process and demonstrated for the first time that these exosome-like vesicules participated both in proliferation of myoblasts and in the step of differentiation into multinucleated myotubes, confirming their important role in the skeletal muscle biology.

Funded by: Fondation pour la Recherche Médicale (FRM), Association Française de recherche sur les Myopathies (AFM) and INRA specific grant (ANSSD).

**34. Intraluminal vesicles and exosomes as nucleating platform for physiological amyloids**

Guillaume van Niel^1^, Leila Rochin^1^, Cecile Fort^1^, Lutgarde Serneels^2^, Bart De Strooper^2^, Daniel Levy^3^ and Graça Raposo^1^

^1^Institut Curie, UMR 144, CNRS, Paris, France; ^2^Center for Human Genetics, Leuven, Belgium; ^3^Institut Curie, UMR 168, Paris, France

Email: guillaume.van-niel@curie.fr

Amyloid fibers are insoluble proteinous aggregates that are mainly associated with pathological situations such as neurodegenerative diseases that include Alzheimer's disease. There is increasing evidence for the role of multivesicular endosomes in the initiation and development of amyloidogenesis. Interestingly, a specialized cell type, the melanocyte, has tuned its endocytic pathway and in particular multivesicular endosomes to produce amyloid fibers in a physiological manner during melanogenesis. These physiological fibers are consequently formed upon the sorting and processing of the transmembrane protein Pmel17 (or PMEL) to the intraluminal vesicles (ILVs) of multivesicular endosomes. Our previous studies and unpublished data indicate several homologies between pathological and physiological amyloidogenesis conferring to the ILVs of multivesicular endosomes the status of amyloid nucleator platform. We have combined cell biological methods (biochemistry, immunofluorescence and siRNA inactivation approaches), quantitative mass spectrometry analysis, morphological studies of melanogenesis in KO mice together with high-resolution electron microscopical methods on secreted ILVs to investigate the sorting and processing mechanisms leading to the biogenesis of the lipid vesicles nucleating amyloid fibril. These results lay the foundations for a physiological model of amyloidogenesis that could be exploited by pathological amyloidogenic protein during the development of associated neurodegenerative diseases.

Funded by: ARC, FRM and NIH.

**35. Microglia-derived microvesicles represent a new pathway of glia-to-neuron communication and a biomarker of brain inflammation**

Claudia Verderio^1^, Elena Turola^1^, Flavia Antonucci^1^, Martina Gabrielli^1^, Alessandra Bergami^2^, Francesca Ruffini^2^, Loredana Riganti^1^, Luca Muzio^2^, Roberto Furlan^2^ and Michela Matteoli^1^

^1^CNR Institute of Neuroscience and Department of Medical Pharmacology, Milan, Italy; ^2^Clinical Neuroimmunology Unit, INSPE, San Raffaele Scientific Institute, Milan, Italy

Email: c.verderio@in.cnr.it

Microvesicles (MVs) are emerging as a novel way of cell-to-cell communication and new biomarkers of tissue damage. To investigate whether microglia-derived MVs affect neurotransmission, we analyzed spontaneous and evoked release of glutamate in neurons exposed to MVs and found a dose-dependent stimulation of excitatory transmission. Paired-pulse recording analysis showed that MVs mainly act at the presynaptic site, by increasing release probability. Stimulation of synaptic activity occurred via enhanced sphingolipid metabolism and was prevented by pharmacological or genetic inhibition of sphingosine synthesis. To verify the existence in vivo of MVs, we analyzed the cerebrospinal fluid (CSF) collected from humans and rodents and found MVs immunopositive positive for microglia/macrophage markers. Notably, flow cytometry quantification indicated elevated levels of myeloid MVs in the CSF collected from patients affected by the prototypic inflammatory brain disease multiple sclerosis (MS), in the acute phase of the disease. The amount of myeloid MVs also increased in the CSF during EAE, a widely used model for MS, following the disease course. Overall these data identify MVs as a new mechanism by which microglia influence synaptic activity and identifying CSF myeloid MVs as novel biomarker of brain inflammation.

Funded by: FISM 2010/R/39

**36. Microvesicles and exosomes in Parkinsons disease model systems**

Allan Stensballe and Kenneth Hedegaard Sørensen

Department of Biotechnology, Aalborg University, Aalborg, Denmark

Email: as@bio.aau.dk

Parkinson's disease (PD), a chronic degenerative brain disease, is characterized by a loss of dopiminergic neurons in the substantia nigra area of the brain and the abnormal presence of ubiquitinated cytoplasmic inclusions called Lewy bodies in affected cells. PD can be diagnosed precisely by postmortem analysis of the affected areas of the brain whereas early clinical diagnosis of PD may be based on the patient's medical history and neurological examinations before a symptom calming treatment can be implemented. Exosome secretion is modulated in different cell types by various environmental changes such as stress conditions and could be one of the means used by tissues to adapt to these changes. Thus, the interest in finding early biomarkers for these neurodegenerative diseases before clinical symptoms arise is great. Cerebrospinal fluid (CSF) is the most relevant biological fluid for biomarker study because CSF has direct contact with the extracellular space in the brain. The CSF proteome is, however, extremely complex, and patient samples are often available only in small volume whereby direct analysis by specific PD biomarkers is a major analytical challenge. Alternatively, conditioned media obtained from appropriate PD model cell lines enable identification of potential biomarkers for PD by investigation of expression profiles of secreted proteins and composition of microvesicles. We present here the methodology for purification and identification of secreted proteins and microvesicle-associated proteins from PD cell lines that change the expression level as a consequence of overexpression of specific proteins (e.g., α-synuclein, Parkin or P25α) through a quantitative mass spectrometry-based approach.

**37. Crosstalk between neurons and glia involving exosomes as vesicular carriers of RNA and proteins**

Dominik Fröhlich^1^*, Carsten Frühbeis^1^*, Jesa Amphornrat^1^, Sebastian Thilemann^1^, Aiman Saab^2^, Frank Kirchhoff^2^, Wiebke Möbius^2^, Klaus-Armin Nave^2^, Anja Schneider^2^, Mikael Simons^2^, Matthias Klugmann^3^, Jacqueline Trotter^1^ and Eva-Maria Krämer-Albers^1^

*Both authors contributed equally^1^Molecular Cell Biology, University of Mainz, Germany; ^2^Department of Neurogenetics, Max Planck Institute of Experimental Medicine, Göttingen, Germany; ^3^Translational Neuroscience Facility, Department of Physiology, School of Medical Sciences, University of New South Wales, Sydney, Australia.

Email: dominik.froehlich@uni-mainz.de

In the CNS, myelinating oligodendrocytes and axons form a functional unit based on intimate cell-cell interactions. In addition to axonal insulation serving to increase the conduction velocity of electrical impulses, oligodendrocytes provide trophic support to neurons essential for the long-term functional integrity of axons. The glial signals maintaining axonal functions are unknown. Yet, their determination is highly relevant for all types of demyelinating diseases, where lack of glial support significantly contributes to pathology. We have recently discovered that oligodendrocytes secrete exosomes containing a distinct set of proteins as well as mRNA and microRNA. Intriguingly, oligodendroglial exosome release is stimulated by the neurotransmitter glutamate, indicating that neuronal electrical activity controls glial exosome release. To analyze the putative transfer of oligodendroglial exosomes to neurons and its implications in glial support, we exposed cultured cortical neurons to fluorescently labeled oligodendroglial exosomes. Indeed, cultured cortical neurons internalized and accumulated oligodendroglial exosomes in the neuronal cell soma in a time-dependent manner. Addition of the dynamin inhibitor dynasore interfered with neuronal exosome internalization, indicating that exosome uptake is mediated by clathrin-dependent endocytosis. Furthermore, neuronal internalisation of exosomes resulted in functional retrieval of exosomal cargo in vitro and in vivo upon stereotactic injection of exosomes. Our results thus provide a proof of principle of exosome transmission from oligodendrocytes to neurons and indicate that oligodendroglial exosomes have the potential to influence the neuronal metabolism.

Funded by: ELA, IFSN University of Mainz and Stipendien Stiftung Rheinland Pfalz

**38. Development of exosome-based drug delivery strategies**

Samira Lakhal-Littleton and Matthew J. Wood

Department of Physiology, Anatomy & Genetics, University of Oxford, United Kingdom

Email: samira.lakhal-littleton@dpag.ox.ac.uk

Discovery of the natural function of exosomes in the transport of RNA and protein highlighted their potential as drug delivery vehicles. This potential was first harnessed by our research team, by utilizing dendritic cell-derived exosomes for targeted siRNA delivery across the blood-brain barrier. Here, we describe further strategies for exosome targeting in vitro and in vivo and explore the wider range of cargoes that can be delivered by exosomes. Our results demonstrate the versatility of exosomes as drug delivery vehicles, both in terms of targeting and cargo type, and highlight the wider range of applications in which they would be of benefit.

Funded by: Novartis

**39. Exosomal transfer of RNA based signals between the hematopoietic system and the brain in response to inflammation**

Kirsten Oesterwind^1^, Sascha Keller^2^, Maria Dams^1^, Jadranka Macas^1^, Britta Landfried^1^, Karl H. Plate^1^, Candan Depboylu^3^, Günter Höglinger^4^, Peter Altevogt^2^ and Stefan Momma^1^

^1^Institute of Neurology (Edinger Institute), University Medical School, Frankfurt, Germany; ^2^Tumor Immunology Program, German Cancer Research Center (DKFZ), Heidelberg, Germany; ^3^Philipps University Marburg, Clinic for Neurology, Marburg, Germany; ^4^German Center for Neurodegenerative Diseases e.V. (DZNE), Technical University Munich (TUM), Germany

Email: kirsten.oesterwind@kgu.de

The influence of peripheral inflammation to the central nervous system is highly discussed in the context of different diseases. However, the exact mechanism of this influence still remains unclear. To address the question of a hematopoietic contribution to neurons, we used a transgenic mouse model expressing the Cre recombinase specifically in the hematopoietic compartment. Subsequent irreversible labeling of the recombined cells allows for tracing any contribution to other cell populations. Interestingly, analysis of cerebellar tissue reveals reporter gene induction in non-hematopoietic cells, specifically in Purkinje neurons, without indication for cell fusion. We provide evidence that reporter gene expression is caused by intercellular transfer of functional Cre recombinase messenger RNA contained in secreted membrane vesicles, particularly in exosomes. We could show that exosomal preparations from Cre expressing hematopoietic cells are sufficient to induce recombination of Purkinje neurons when injected directly into cerebellar tissue. Furthermore, recombination of Purkinje neurons increases significantly after inducing inflammatory injuries. Recombination events are not only restricted to Purkinje neurons but also include other neuronal populations in different areas of the brain. These observations reveal the existence of a previously unrecognized way to communicate RNA-based signals between the immune system and the brain after peripheral inflammation.

Funded by: Edinger Foundation; Deutsche Forschungsgesellschaft (DFG)

#### 4. Treatment of Malignancy

**40. Acidic exosomes contain molecules functionally referred to melanoma progression**

I. Parolini^1^, C. Coscia^1^, C. Zanetti^1^, L. Fantozzi^2^, F. Felicetti^1^, E. Pizzi^2^, M. Ponzi^2^, A. Carè^2^ and M. Sargiacomo^2^

^1^Department of Hematology Oncology and Molecular Medicine, Istituto Superiore di Sanità, Rome, Italy; ^2^Department of Infectious Diseases, Istituto Superiore di Sanità, Rome, Italy

Email: isabella.parolini@iss.it

The striving characteristic of metastatic melanoma cells to grow in vitro at pH 6.0 can be referred to exosomes found in the medium of these cells, which through unknown mechanisms promote cell surviving. In such extreme conditions, cells communicate with the surrounding microenvironment through enhanced secretion of specific exosomes (acidic exosomes). We found that acidic exosomes were more efficiently transferred into recipient cells through a fusion membrane mechanism, increasing cell migration ability with respect to a primary melanoma. To seek specific protein profiles accounting for cell modifications in tumor progression, exosomes secreted from melanoma cells kept at pH 6.0 and pH 7.4 for 48 hours were subjected to proteomic analysis by mass spectrometry. We found that pH drives different protein expression into exosomes. More specifically, acidic exosomes contained proteins belonging to leukocyte transendothelial migration (LTM) and extracellular matrix (ECM) family proteins, typically expressed in aggressive tumor cell phenotypes. Among them, the integrin αvβ3, already known as responsible of increased migration and invasive ability, was found enriched by MS/MS and Western blotting analyses. Furthermore, we searched for the possible involvement of microRNAs. Specifically, we focused on selected microRNAs theoretically targeting αv as well as β3 mRNAs, as the miR-200 family and miR-146, already reported to play a tumor suppressor role in cancer.

Funded by: Italian Ministry of Health

**41. Acidity and exosomes contribution in the Cisplatin uptake in resistant melanoma cell line**

C. Federici^1^, F. Petrucci^2^, S. Caimi^2^, N. Violante^2^, A. Cesolini^3^, S. D'Ilio^2^, C. Majorani^2^, M. Borghi^1^, M. Logozzi^1^, L. Lugini^1^ and S. Fais^1^

^1^Department of Therapeutic Research and Medicines Evaluation, ^2^Department of Environment and Primary Prevention; ^3^Department of Cell Biology and Neuroscience, Istituto Superiore di Sanità, Rome, Italy

Email: cristina.federici@iss.it

Multiple drug resistance of tumor cells is a common problem in cancer therapy. An important mechanism of tumor resistance to cytotoxic drugs involves increased acidification of extracellular compartments. Considering that the tumor microenvironment is characterized by regions of hypoxia and acidity, we investigate whether the extracellular acidosis can influence tumor cell sensitivity to chemotherapeutic agents’ treatment. Therefore, we analyzed if proton pump inhibitors (PPIs, Omeprazole), currently used to block the H + transporters, may inhibit the acidification of the tumor microenvironment and increase the sensitivity of tumor cells to cytotoxic agents. We also investigate the importance of exosomes, small vesicles secreted by normal as well as tumor cells, as a novel system in the drug delivery. In this work, we demonstrate that: (a) the drug uptake reduced of about 20% in tumor sensitive cells and about 50% in resistant ones, as a function of pH decrease; (b) at lower pH (5.0 or 6.0), the treatment with PPI implies an enhancement of the drug level into the cell; (c) the same results were also obtained in SCID mice engrafted with Me30966melanoma cells and treated with PPI and Cisplatin; (d) the Cisplatin in the exosomes was essentially present in its native form. Probably, the exosomes incorporated the drug immediately after the cell uptake and before its activation by the hydratation. The release of exosomes at acidic pH was characterized by a higher Cisplatin amount than the exosomes released at neutral pH. So, we advance the hypothesis that the exosomes released may represent a mechanism of cellular drug detoxification and a novel strategy of cancer cells to set up the multidrug resistance.

Funded by: Istituto Superiore di Sanità, Rome, Italy

**42. Oncogene trafficking through the endosomal-exosomal pathway**

Frederik J. Verweij, Monique A.J. v. Eijndhoven, Erik S. Hopmans, Tineke Vendrig, Tom Wurdinger, Ellen Cahir-McFarland, Elliott Kieff, Dirk Geerts, Rik v/d Kant, Jacques Neefjes, Jaap M. Middeldorp and D. Michiel Pegtel

Dept. of Pathology, Cancer Center Amsterdam, Amsterdam, The Netherlands

Email: f.verweij@vumc.nl

Developing B cells require a CD40-mediated signal from T cells (T cell help) to survive in the germinal center reaction. Epstein-Barr virus infects naïve B cells and through expression of a specific latency program drives the newly infected cells through a GC reaction into memory B cells. This process relies heavily on timely expression of the virus-encoded CD40 mimic latent membrane protein 1 (LMP1), thereby playing a key role in establishing viral persistence. Unlike CD40, LMP1 activates NFκB constitutively and poses a risk factor for EBV-associated lymphomas. Despite this knowledge, over 90% of the world population is persistently infected with EBV, seemingly without causing much harm. How EBV-infected cells that express oncogenic LMP1 are protected from malignant conversion is poorly understood. We proposed that a major proportion of LMP1 is restrained from signaling by incorporation into late endosomes/multivesicular bodies, resulting in secretion of the protein via exosomes. We discovered that a single mutation in the active palmitoylation site of LMP1 results in increased intracellular aggregation and elevated recruitment of a key signaling mediator TRAF2. As expected, this co-aggregation enhanced NFκB overstimulation and delayed secretion of the protein via exosomes. Recent studies indicate that internalized signaling receptors such as the Met receptor or Src kinase can continue to signal from endosomal membranes promoting oncogenic transformation. This suggests that LMP1 expressed in healthy EBV-infected cells B cells must be restrained from prolonged signaling from endolysomal membranes. Consistent with this, we found that, while LMP1 is strongly associated with TRAF2 in B cells, TRAF2 is absent in the exosome fraction, indicating that, before the incorporation of LMP1 in intraluminal vesciles with CD63, TRAF2 dissociates from this complex. We are currently investigating the mechanism driving this dissociation, which we believe will provide valuable insights into ESCRT independent sorting of membrane proteins into exosomes. Thus, EBV-infected cells are protected from oncogenic signaling by LMP1 due to recruitment of CD63 into microdomains and dissociation of TRAF2 before incorporation into ILVs that restrain the constitutive NFκB activation. This process depends, at least in part, on the palmitoylation status of the protein.

Funded by: The Cancer Foundation (KWF-3775) and in part by the Dutch Science foundation (NWO-Veni) to DMP.

**43. Role for circulating Hsp72 in hyperthermia-induced tumor regression**

Punit Kaur, Edwina Asea and Alexzander Asea

Division of Investigative Pathology, Scott & White Memorial Hospital and Clinic and the Texas A&M Health Science Center College of Medicine, Temple, TX, USA

Email: pkaur@medicine.tamhsc.edu

The mechanism by which hyperthermia (HT) induces the release of Hsp72 is just in beginning to be elucidated. In this study, we show that the released Hsp72 is found in two forms: (1) within highly immunologically active exosomes, known to be enriched in MHC class I and II complexes, co-stimulatory molecules and members of the HSP70 family, and (2) as heat shock protein peptide complexes, in which free Hsp72 is chaperoning tumor specific peptides. Further, we show that exposure of tumor-bearing mice to HT (41°C, 60 minutes) induces significant tumor regression (*p*<0.05) as compared to tumor-bearing mice maintained at ambient temperature. However, repeated injection of tumor-bearing mice with anti-Hsp72 antibody abrogated HT-induced tumor regression. Blood samples taken at various times confirmed that the blocking antibody significantly reduced plasma Hsp72 levels.

Funded by: Scott & White Memorial Hospital and Clinic Research Advancement Awards (to P.K.); the US National Institute of Health (RO1CA91889), Scott & White Memorial Hospital and Clinic, the Texas A&M Health Science Center College of Medicine, the Central Texas Veterans Health Administration and an Endowment from the Cain Foundation (to A.A.).

**44. Prostate cancer-derived exosomes**

Pedram Kharaziha and Theocharis Panaretakis

Department of Oncology-Pathology, Karolinska Institute, Solna, Sweden

Email: pedram.kharaziha@ki.se

Exosomes constitute the newest mode of intercellular communication, transmitting information between cells. This exchange of molecular information is facilitated by their composition, which is enriched with enzymes, structural proteins, adhesion molecules, lipid rafts, miRNAs and mRNAs. Prostate cancer is the most frequent malignancy in men. Exosomes can be used as diagnostic and prognostic markers in detection and treatment of prostate cancer. We have isolated and characterized exosomes from two prostate cancer cell lines: PC3, an androgen-independent highly, metastatic, therapy-resistant cell line, and 22Rv1, a hormone responsive, non-metastatic and therapy-sensitive cell line. We found 477 proteins in total, 228 of which are involved in tumorigenic pathways. There are significant differences in exosomal protein content between these two cell lines, perhaps signifying their differences in metastatic potential. As expected, the proteome is enriched with proteins regulating vesicular trafficking. In addition, the five most represented signaling cascades are regulating gene expression, adhesion, metastasis and metabolism. The in vivo relevance of these findings is examined in exosomes from prostate cancer patients.

Funded by: Grants from Cancerfonden, Vetenskapsrådet and Karolinska Institutet awarded to T. Panaretakis

**45. Investigation into the role of exosomes in prostate cancer**

Elham Hosseini-Beheshti, Hans Adomat, Steven Pham and Emma S. (Tomlinson) Guns

The Vancouver Prostate Centre, University of British Columbia, Vancouver, BC, Canada

Email: ebeheshti@prostatecentre.com

Prostate cancer is the leading type of cancer diagnosed in men. In 2010, approximately 217,730 new cases of prostate cancer were reported in the United States. Prompt diagnosis of the disease can substantially improve its clinical outcome. Improving capability for early detection as well as developing new therapeutic targets in advanced disease are research priorities that will ultimately lead to better patient survival. Eukaryotic cells secrete proteins via distinctly regulated mechanisms, which are either ER/Golgi dependent or microvesicle mediated. The release of microvesicles provides a novel mechanism for intercellular communication. Exosomes are nanometer sized cup-shaped membrane vesicles, which are secreted from normal and cancerous cells. They are present in various biological fluids and are rich in characteristic proteins. Exosomes may thus have potential both in facilitating early diagnosis via less invasive procedures or be candidates for novel therapeutic approaches for castration resistance prostate cancer. In this study, we hypothesize that exosomes have a pivotal role in cell-cell communication in the local tumor microenvironment, conferring activation of numerous survival mechanisms during prostate cancer progression and development of therapeutic resistance. Exosomes may also facilitate prostate cancer diagnosis via less invasive procedures. We thus characterized exosomes derived from six prostate cell lines and tracked their uptake in both cancerous and benign prostate cell lines, respectively. Our comprehensive proteomic and lipidomic analysis of prostate-derived exosomes forms a platform for future validation of novel biomarkers and therapeutic targets for castration resistance prostate cancer.

Funded by: CIHR

**46. Exosomes and chemotherapy drug resistance intercellular transfer**

P. Colosetti^1^, C. Lässer^2^, A. Jacquel^1^, J. Lötvall^2^ and P. Auberger^1^

^1^Inserm U1065-C3M E2, Université de Nice-Sophia Antipolis, France; ^2^Krefting Research Centre, University of Göteborg, Sweden

Email: Pascal.Colosetti@unice.fr

Drug resistance is a drawback during cancer treatment and it is compromising the benefit of chemotherapy. Chronic myelogenous leukemia (CML) is a myeloproliferative hematological neoplasm with a particular chromosome 22 to chromosome 9 translocation [t(9,22), Philadelphia chromosome], producing a constitutively active tyrosine kinase fusion protein: Bcr-Abl. The tyrosine kinase inhibitor (TKI), Imatinib mesylate (Ima, Gleevec), targeting Bcr-Abl, is the front-line chemotherapy for CML. The majority of the CML patients are demonstrating complete hematological response but first-line failure is observed for around 25% of patients. Exosomes released by cells in their environment and carrying a wealth of biological information can fuse with the target cells, resulting in the non-selective transfer of proteins and RNAs. Few links between drug resistance and microvesicles/exosomes have been reported (doxorubicine, enhanced export of cisplatin and transfer of P-glycoprotein). The purpose of this study is to investigate whether exosomes can contribute to drug resistance during chemotherapy of CML, i.e., deciphering the characteristics of such resistance exosomes and intercellular communication between sensitive and Ima-resistant CML K562 model cells. We have isolated exosomes (from sensitive and Ima resistant cells), extracted and analyzed exosomal RNAs and proteins. Exosome bead capture was also performed and analyzed by flow cytometry. After monitoring/evaluating the exosome cellular transfer, it will be of great interest to characterize the impact of such information transfer and resulting effects on Ima response.

Funded by: Inserm and UICC

**47. Formulation and evaluation of ethosomal vesicular carrier system for vaginal delivery of metronidazole**

Chukwuemeka C. Mbah^1,2^, Philip F. Builders^1^, Anthony A. Attama^2^ and Michael U. Adikwu^2^

^1^Department of Pharmaceutical Technology and Raw materials Development, National Institute for Pharmaceutical Research and Development, Idu, Abuja, Nigeria; ^2^ Department of Pharmaceutics, Faculty of Pharmaceutical Sciences, University of Nigeria, Nsukka, Enugu State, Nigeria

Email: mbaccc@yahoo.com

Vesicular carriers have been identified as useful systems for enhanced delivery of active pharmaceutical ingredients. A pH-responsive ethosomal vesicular carrier system was formulated by the solvent evaporation method and evaluated for enhanced delivery of metronidazole to the vagina using Franz diffusion cell. Data obtained were analyzed by ANOVA and Student's *t*-test using Excel Microsoft Office 2007. The scanning electron microscopy showed presence of spherically shaped, nanosized vesicles in the formulation with sizes (diameter) ranging from 118.0 to 519.1 nm. The entrapment efficiency, loading efficiency and vesicle yield were 50.31±3.38%, 39.89±0.02% and 15.78±1.57%, respectively, and it was most stable at 4–8°C. Over the pH range of 6.5–5.0, the penetration and flux were of the order: 5.6 > 5.0 > 6.5, respectively, with the formulation of pH 5.6 showing a significantly (*p*<0.05) higher release profile than the others. It showed potentials for controlled and enhanced delivery, attributable to increased penetration rate and residence time in the vaginal linings. The vesicular formulation may enhance delivery of the entrapped metronidazole particles, thus could be further developed as an alternative application for treatment of susceptible vaginal infections.

**48. Extracellular vesicles constitute a novel mediator of coagulation-dependent hypoxic signalling in cancer**

Mattias Belting^1^, Johan Bengzon^2^, Helena C. Christianson^1^, Maria C. Johansson^1^, Paulina Kucharzewska^1^, Matthias Mörgelin^3^ and Katrin J. Svensson^1^

Department of Clinical Sciences, Sections of ^1^Oncology, ^2^Neurosurgery, and ^3^Clinical and Experimental Infectious Medicine, Lund University, Lund, Sweden

Email: Mattias.belting@med.lu.se

Highly malignant brain tumors, such as glioblastoma, are characterized by hypoxia, endothelial cell (EC) hyperplasia and hypercoagulation. However, how these phenomena of the tumor microenvironment may be linked at the molecular level during tumor development remains ill-defined. We recently provided evidence that hypoxic cancer cells release substantial amounts of tissue factor (TF), i.e., the major initiator of coagulation, associated with secreted microvesicles with exosome-like characteristics (1). Further, we found that hypoxia upregulates protease-activated receptor 2 (PAR-2), i.e., a G-protein coupled receptor of coagulation-dependent signaling, in ECs. Interestingly, microvesicles derived from glioblastoma cells were found to trigger TF/PAR-2-dependent activation of hypoxic ECs in a paracrine manner. Ongoing studies (2) that expand on these findings indicate that the molecular information (protein, mRNA and miRNA) carried by cancer cell-derived extracellular vesicles is substantially altered by hypoxia, resulting in potent angiogenic activities in vitro and in vivo. We conclude that extracellular vesicles constitute a novel, potentially targetable mediator of hypoxia-driven tumor development and suggest that the hypoxic vesicle signature may serve as a non-invasive biomarker to assess the oxygenation status and aggressiveness of malignant tumors.

Funded by: The Swedish Research Council; the Swedish Cancer Fund; the Swedish Society of Medicine; the Physiographic Society, Lund; the Gunnar Nilsson and Kamprad Foundations; the Lund University Hospital donation funds; and the Governmental funding of clinical research within the national health services (A.L.F.).

**References**

1. Svensson KJ, et al. Proc Natl Acad Sci USA. 2011 Aug 9;108(32):13147–52.

2. Kucharzewska, et al. submitted.

**49. Carboxyamidotriazole-orotate inhibits the growth of Imatinib-resistant chronic myeloid leukemia cells and modulates exosomes-stimulated angiogenesis**

Riccardo Alessandro^1^, Chiara Corrado^1^, Simona Taverna^1^, Stefania Raimondo^1^, Anna Maria Flugy^1^, Rashida Karmali^2^ and Giacomo De Leo^1^

^1^Dipartimento di Biopatologia e Biotecnologie Mediche e Forensi, Sezione di Biologia e Genetica, Università di Palermo, Italy; ^2^Tactical Ttherapeutics, Inc., New York, NY, USA

Email: riccardo.alessandro@unipa.it

Chronic myeloid leukemia (CML) is characterized by the expression of Bcr–Abl oncoprotein with a constitutive tyrosine kinase that drives disease pathogenesis. Imatinib is the election therapy for CML, but some patients are resistant to this drug. Recently, attention is being focused on cell-cell communication that involves membrane vesicles called exosomes. A number of studies have described exosomes as new players in modulating the tumor microenvironment, promoting angiogenesis and tumor development; furthermore neovascularization, is known to exert an important role in the progression of chronic myeloid leukemia and may represent a valid alternative target for therapy. Little is known regarding the role of exosomes in CML biology. Our data indicate that LAMA84 R, an Imatinib-resistant human CML cell line, releases exosomes and that the addition of those microvesicles to human vascular endothelial cells affects in vitro and in vivo angiogenesis. Interestingly, in the last years, some data have indicated that modulation of exosome release by pharmacological agents may affect malignant progression. We have tested the effects of the carboxyamidotriazole-orotate (CTO) on LAMA84 R, and we observed the inhibitor effects of CTO on LAMA84 R cells proliferation and on CML tumor xenografts growth. CTO is able to decrease in vitro BCR/ABL level expression and its phosphorilation and consequently inhibition of the downstream signaling. Our data also show that treatment of endothelial cells with CTO may inhibit exosomes-dependent angiogenesis by interfering with signal transduction pathways activated in endothelial cells by interaction with the microvesicles.

Funded by: University of Palermo and Tactical Therapeutics

**50. Exosomal evasion of humoral immunotherapy in aggressive B-cell lymphoma modulated by ATP-binding cassette transporter A3**

Thiha Aung

University of Goettingen, Department of Haematology/Oncology, Goettingen, Germany

Email: aung.anb@med.uni-goettingen.de

Humoral immunotherapies against CD20 represent a very efficient therapeutic tool against malignant lymphoma. However, in cases of resistant desease, there is mostly a fatal outcome due to the variation of tumor cell susceptibility to immunochemotherapy. Here, we show that lymphoma exosomes shield target cells from antibody attack and that exosome biogenesis is modulated by the lysosome-related organelle-associated ATP-binding cassette (ABC) transporter A3 (1). The protection of target cells from antibody attacks was mediated through exosomes carrying CD20, which bound therapeutic anti-CD20 antibodies and consumed complement. ABCA3, which we previously characterized as a mediator of resistance to chemotherapy, was critical for the amounts of exosomes released, and both pharmacological blockade and the silencing of ABCA3 enhanced susceptibility of target cells to antibody-mediated lysis. Mechanisms of cancer cell resistance to drugs and antibodies are linked in an ABCA3-dependent pathway of exosome secretion.

Funded by: Deutsche Forschungsgemeinschaft Grant DFG

**Reference**

1. Aung T, et al. Proc Natl Acad Sci USA. 2011 Sep 13;108(37):15336–41.

**51. New insights in the potential mechanisms involved in the human breast cancer metastasis and chemotherapy resistance**

M. Thirion^1^, Y. Sugihara^2^, N. Kosaka^1^, T. Kondo^2^ and T. Ochiya^1^

^1^Division of Molecular and Cellular Medicine and ^2^Division of Pharmacoproteomics, National Cancer Center Research Institute, Tokyo, Japan

Email: muriel.th@hotmail.com

The discovery and characterization of extracellular vesicles has broadened understanding of the intercellular communication and the entire physiology of the organism. These message particles consist of proteins, lipids and nucleic acids derived from the originating cells. The release of those extracellular vesicles from cancer cells into their microenvironment is becoming increasingly recognized as a novel feature of cancer biology. Here, we focused on the human breast cancer and performed proteome analysis of the extracellular vesicles secreted by MDA-MB-231 and MDA-MB-231LN malignant breast cancer cells. We identified unique proteins implicated in angiogenesis promotion and stromal remodeling, which would impact the generation of a survival, premetastatic niche. We also identified new extracellular vesicles’ proteins that could either explain the preferential metastatic sites associated to breast cancer or play a critical role in its resistance to therapeutics. This study therefore provides novel potential targets to support the upcoming applications in diagnosis, prognosis and therapeutics.

Funded by: National Cancer Center Research Insitute

**52. Can exosomes influence triple negative breast cancer metastasis?**

K. O'Brien, S. Rani, R. Wallace, S. McDonnell, L. Hughes, M. Radomski and L. O'Driscoll

School of Pharmacy & Pharmaceutical Sciences, Trinity College, Dublin, Ireland; School of Chemical and Bioprocess Engineering, University College, Dublin, Ireland

Email: obrienk9@tcd.ie

Triple-negative breast cancer (TNBC) is associated with high mortality rates and incidence in younger women. Our analysis primarily investigated the relevance of exosomes in TNBC, by comparing the effects of exosomes derived from Hs578T and its more invasive subclone, Hs578Ts(i)8, as well as exosomes derived from TNBC compared to control sera. The effects of exosomes were analyzed on secondary cell proliferation, motility, invasion, anoikis and endothelial tubule/vessel formation. Hs578Ts(i)8 exosomes, compared to Hs578T exosomes, conferred increased proliferation, motility and invasion of SKBR3, MDA-MB-231 and HCC1954 breast cancer cell lines; as well as Hs578Ts(i)8-derived exosomes inducing Hs578T cells to be more invasive. Additionally, Hs578Ts(i)8-derived exosomes stimulated greater tubule formation than Hs578T-derived exosomes. However, Hs578TS(i)8-derived exosomes, compared to Hs578T exosomes, sensitised the SKBR3, MDA-MB-231 and HCC1954 cell lines to anoikis; a finding consistent with the innate phenotype of both cell Hs578T variants. Furthermore, exosomes isolated from TNBC patients’ serum, compared to those from control sera, increased the invasiveness of SKBR3 cells. Further investigation established the presence of RNA in serum-derived exosomes, supporting the potential for exosomes as cargos of cancer biomarkers. Our analyses indicate that exosomes influence secondary cells in a manner indicative of the innate phenotypes of their donor cells, as well as having the potential as carriers of cancer biomarkers.

Funded by: The Marie Keating Foundation; Trinity College Postgraduate Award and Science Foundation Ireland's funding of Strategic Research Cluster, Molecular Therapeutics for Cancer Ireland

**53. Multiple myeloma cells secrete nanovesicles that stimulate IL-11 secretion in osteoblast-like recipient cells**

Olaf Strømme^1^, Katarzyna M. Psonka-Antonczyk^2^, Bjørn Torger Stokke^2^, Anders Sundan^1^ and Gaute Brede^1^

^1^Faculty of Medicine, Department of Cancer Research and Molecular Medicine, Norwegian University of Science and Technology, Trondheim, Norway; ^2^Department of Physics, The Norwegian University of Science and Technology, NTNU, Trondheim, Norway

Email: olafs@stud.ntnu.no

Multiple myeloma (MM) is a neoplasm of terminally differentiated B cells. MM is associated with unbalanced bone remodeling causing lytic bone lesions. Interleukin-11 (IL-11) promotes osteoclast formation and inhibits osteoblast activity and may thus be one factor involved in cancer-induced bone destruction. Exploring the intertwined complexity between the neoplastic cells and the impact of their microenvironment is important for understanding early myelomagenesis. The impact of nanovesicles in myeloma development and signaling within the bone marrow has, to the best of our knowledge, not yet been investigated. We show here that syndecan-1 positive (CD138^+^) myeloma cells secrete nanovesicles that are taken up by osteoblast-like cells and increase the secretion of IL-11 in the recipient cells. Our results suggest a role of exosomes in bone marrow signaling and therefore a functional role of secreted nanovesicles in disease progression.

Funded by: The cooperation entity Helse Midt-Norge RHF and NTNU

**54. Characterization of Epstein-Barr virus (EBV) protein and nucleic acid content of exosomes released in vitro from EBV-infected cells**

Andrea Canitano, Martina Borghi, Stefano Fais and Giulietta Venturi

Department of Therapeutic Research and Medicines Evaluation, Istituto Superiore di Sanità, Rome, Italy

Email: andrea.canitano@iss.it

EBV is a human herpes virus associated with a number of malignancies. Both lymphoblastoid cell lines (LCLs) and EBV-infected nasopharyngeal carcinoma (NPC) cells have been demonstrated to release exosomes containing the EBV-encoded latent membrane protein 1 (LMP1) and mature miRNAs. Moreover, the presence of exosomes with EBV-LMP1 and miRNAs has been demonstrated in serum and saliva samples of patients with EBV-positive NPC and has been proposed as a diagnostic marker for this malignancy. The aim of our work was the characterization of the EBV protein and nucleic acid content of exosomes released in vitro from EBV-infected cells (LCL and PBMC obtained from two EBV-positive healthy donors). We have analyzed the expression of LMP1 protein, a panel of 4 EBV-encoded miRNAs, and EBNA1, EBNA2, LMP1 e LMP2 mRNAs coding sequences. In agreement with literature data, LMP1 protein could be clearly detected in LCL-derived exosomes. All of the EBV-mRNAs analyzed except EBNA1 were present in RNA isolated from LCL-derived exosomes. LMP1 and EBNA2 were present at very low levels, while LMP2 mRNA was the most abundant. No EBV mRNA could be detected in RNA isolated from PBMC of EBV positive healthy donors, nor from PBMC derived exosomes. All of the EBV-encode miRNAs analyzed could be detected in LCLs and also in LCL-derived exosomes. Moreover, some of the EBV-miRNAs analyzed could be detected in PBMC and PBMC-derived exosomes. Overall, a poor correlation in levels of mRNA and miRNA in the cells, compared with exosomes, could be observed, suggesting selective enrichment of some cellular RNA in exosomes.

Funded by: FISM—Fondazione Italiana Sclerosi Multipla

## Scientific Program 2012 ISEV meeting Friday 20th April

### Symposium Session 7          8.30-10.00

#### 1. Extracellular Vesicles in Inflammation and Immunity

**Characterization of exosomes isolated from dendritic cells differentiated from human iPS cells**

Yi Lee, Jinghuan Li, Christopher Gardiner, Alison Leishman, Tim Davies, Samir El Andaloussi, Ian L. Sargent, Paul J. Fairchild and Matthew J.A. Wood

Department of Physiology Anatomy and Genetics, University of Oxford, Oxford, United Kingdom; Nuffield Department of Obstetrics and Gynaecology, University of Oxford, Oxford, United Kingdom; Sir William Dunn School of Pathology, University of Oxford, Oxford, United Kingdom

Email: yi.lee@dpag.ox.ac.uk

Exosomes are membrane vesicles naturally released from most cells and found in most bodily fluids. They can cross many biological barriers and transfer their mRNA and protein content between cells. Exosomes are reported to have a common set of surface markers, yet exosomal RNA and protein content can differ according to their parental cell line. For example, exosomes from embryonic stem cells (ES) carry reprogramming factors that could influence the phenotype and development of other stem cells. Induced pluripotent cells (iPSCs) are very similar to ES cells in terms of cell potency and the expression of specific stem cell genes and proteins. Further, iPSCs can be differentiated into various cell lineages such as dendritic cells (iPS-DC). DCs are generally known to produce exosomes, under both normal and stress conditions, possibly to aid in activating correct immune responses to pathogens. However, little is known about exosomes from iPS-DCs. Human-iPS (hiPS) cells were grown in serum-free media and they were further differentiated into DCs over 1 month. Subsequently, iPS-DCs were matured in a cocktail of proinflammatory cytokines. Supernatants were collected at various stages of DC differentiation and from both immature and mature DCs, where exosomes were isolated using an established ultracentrifugation protocol. We confirmed the presence of exosomes through the detection of exosomal markers such as CD9 and Flotillin-1 in western blots. Nanoparticle tracking analysis (NTA) using Nanosight also showed that these iPS-DCs generally produce large numbers of particles (1012 particles/ml) that are around 150 nm in size, similar to exosomes derived from other cell types. Our results show that exosomes can be isolated easily from iPS-DCs and they may be applicable for in vitro and in vivo experiments to study stem cell communication and/or gene therapy as delivery vehicles.

Funded by: The Agency for Science, Technology and Research (A*STAR, Singapore)

**Characterization of syncytiotrophoblast microparticles (STMP) and their effect on cytokine profiles during in vitro leukocyte culture for de novo anti-human platelet antigen-1a (HPA-1a) antibody production**

Jane Eastlake^1^, Kate Heesom^2^ and Belinda Kumpel^1^

^1^Bristol Institute for Transfusion Sciences, NHS Blood and Transplant, Bristol, United Kingdom; ^2^Proteomics Facility, University of Bristol, School of Biochemistry, Medical Sciences Building, University Walk, Bristol,United Kingdom

Email: Jane.Eastlake@nhsbt.nhs.uk

Placental syncytiotrophoblast microparticles expressing fetal antigens are shed into maternal blood and could potentially be responsible for primary immune responses in neonatal alloimmune thrombocytopenia (NAIT). NAIT is due to maternal antibodies to fetal platelet antigens of paternal origin. Anti-HPA-1a, commonly implicated, targets a polymorphism on CD61, which is also present on STMP. STMP-13g and ultracentrifuged STMP-100g prepared from fresh term placenta have been characterized by electron microscopy, nanoparticle tracking analysis (NTA, Nanosight), Western blot, Orbitrap mass spectrometry, flow cytometry and RT-PCR. They range in size from 32 to 500 nm and consist of ∼2000 proteins with serum albumin, serotransferrin and complement C3 being the most abundant. They also contain CD51 (alphaV), CD61 (beta3) and CD41 (alpha2b) integrin subunits. Placental alkaline phosphatase is present and is used as a specific marker. In vitro immunization of naïve HPA-1b1b, HLA-DRB3*0101 leucocytes with HPA-1a + STMP and/or platelets was performed. Dendritic cells were immunized with antigen, matured, then cultured with Th(CD4^+^)/B(CD19^+^)/other(CD4^−^) cells for 30 days. Culture supernatants were screened for: cytokines by Luminex; human anti-HPA-1a by MAIPA and/or PIFT and total IgG quantitated by ELISA. The most predominant cytokines were IL-6,-8,-12,-13 and TGF-beta with very low levels of IL-1b,-2,-5,-10 and IFN-gamma. No IL-15,-17,-21 or IFN-alpha was detected. IL-4, GM-CSF and TNF-alpha were found at levels correlating with supplemented media. In all cases, increased levels of cytokines were seen with the addition of antigen with the exception of IL-13, where levels decreased. STMP-100g was the most stimulatory, then STMP-13g and platelets. Platelets gave an equivalent response for IL-6 and IL-8 yet caused the greatest suppression of IL-13. In conclusion, placental microparticles are immunomodulatory, enhancing most cytokine responses.

Funded by: National Health Service Blood and Transplant (NHSBT)

**Exosomal CD23: an explanation for the immunogenicity of IgE complexes**

Rebecca K. Martin, Keith B. Brooks and Daniel H. Conrad

Department of Microbiology and Immunology, Virginia Commonwealth University, Richmond, VA, USA

Email: rksmith@vcu.edu

We have previously reported that B-cells, upon stimulation with anti-CD40 and IL-4, secrete CD23 expressing exosomes. The low affinity IgE receptor, CD23, is important in binding monomeric IgE as well as IgE/antigen immune complexes (ICs). IgE ICs, through CD23 on B-cells, induce increased specific T-cell proliferation and specific antibody responses. Thus, we hypothesized that CD23^+^ exosomes would potentially play an important role in this pathway. To elucidate this role, we isolated B-cell derived exosomes using ultracentrifugation from cultures stimulated with anti-CD40 and IL-4 and cultured with IgE ICs. Western blot analysis showed that CD23-expressing exosomes were also positive for IgE, indicating the capacity for CD23^+^ exosomes to carry IgE ICs. To further investigate whether these IgE IC-bearing exosomes were able to induce specific T-cell proliferation, cell proliferation assays using [H3] thymidine. IgE anti-TNP/Ova-TNP IC bearing exosomes were co-cultured with CD11c^+^ cells as antigen presenting cells (APCs), irradiated and subsequently placed with CD4^+^ T cells isolated from DO11.10 mice, transgenic TCR specific for Ova. IgE IC-carrying exosomes induced increased specific T-cell proliferation compared to exosomal controls. Intravenous injection of these IgE IC-exosomes into mice adoptively transferred with CD4^+^ T cells from DO11.10 also resulted in increased specific CD4^+^ T-cell proliferation compared to exosomal controls. These data suggest a capacity for CD23^+^ exosomes to carry IgE ICs and provide a novel transport system for antigens that can induce a specific immune response. This also highlights an exciting application of utilizing IgE IC loaded-exosomes for immunization.

Funded by: American Asthma Foundation Grant 11-0094 and NIH RO1 AI18697

**Regulation of endothelial cell functions by macrophage-derived microRNAs**

Elisa Araldi, Aránzazu Chamorro-Jorganes, Carlos Fernández-Hernando and Yajaira Suárez

Department of Medicine and Cell Biology, Leon H. Charney Division of Cardiology, New York University School of Medicine, New York, New York, USA

Email: elisa.araldi@nyumc.org

Angiogenesis and inflammation are closely integrated processes. In the tumor, microenvironment-activated macrophages are the first population that invades the tumor, but inflammatory macrophages are then substituted by angiogenic macrophages. Because of the intimate relationship between inflammation and angiogenesis, it is essential to understand the crosstalk between macrophages and endothelial cells. Recently, microRNAs have been implicated as important regulators of endothelial cell and macrophage functions. Exosomes have been shown to mediate the transfer of proteins and RNAs, including miRNAs, from cell to cell, facilitating the exchange of information. Intriguingly, stimulated cells secrete exosomes with a different microRNA and mRNA composition, so we reasoned that exosomes from inflammatory macrophages (LPS/IFNγ-treated) would contain a particular population of microRNAs. We analyzed the microRNA content of exosomes from inflammatory and control macrophages and found that exosomes derived from inflammatory macrophages were enriched in antiangiogenic miR-16 and proinflammatory miR-155, among others. We assessed exosome activity in endothelial cells in vitro and found that indeed inflammatory exosomes have an antiangiogenic potential. Altogether, these results indicate that microRNAs in exosomes isolated form inflammatory macrophages participate in the paracrine communication with endothelial cells modifying their phenotype in vitro and may be relevant in the context of tumor angiogenesis.

Funded by: SDG-AHA 0835481N (to Y.S.) National Heart, Lung, and Blood Institute R01-HL105945 (to Y.S.)

**Neutrophilic granulocytes produce different microvesicles upon stimulation with different agents**

A. Lorincz^1^, Cs. Timar^1^, D.W. Powell^2^, K.R. McLeish^2^ and E. Ligeti^1^

^1^Department of Physiology, Semmelweis University, Budapest, Hungary; ^2^Departments of Medicine and of Biochemistry and Molecular Biology, University of Louisville, Louisville, KY, USA

Email: lorincz.akos@med.semmelweis-univ.hu

*Background*: Cell-derived microvesicles (MV) are involved in blood coagulation, antigen presentation and transfer of plasma membrane receptors or miRNA. As we showed before, activated neutrophilic granulocytes (PMN) produce microvesicles with antibacterial effect. In this work, we investigated the composition and the biological effects on other cells of microvesicles derived of activated or non-activated neutrophilic granulocytes (PMN). *Materials and methods*: PMN were prepared from the blood of healthy volunteers. After incubation of PMN with activator, cell-free material was separated by two-step centrifugation and filtration. The structure of MV was analyzed with electron microscopy. The protein content was investigated with proteomics and Western blotting. The MV uptake by cells was followed by flow cytometry. *Results*: We found that PMN release MV spontaneously (sMV), but the MV production could be increased upon stimulation with opsonized bacteria (bMV). The two different types of MV showed no difference in size but bMV contained more granulum proteins, and it was more effective against bacteria than sMV. Both types of MV could be taken up by non-activated PMN, and we could not observe any difference in phagocytosis ratio before and after MV uptake. *Conclusion*: PMN are able to change their MV production after bacterial activation. They produce more MV, with more antibacterial proteins. This could explain the stronger antibacterial effect, what we showed earlier. PMNs are able to reuptake their MVs, and this does not facilitate the phagocytosis.

Funded by: The Hungarian National Research Fund (OTKA K81277 and K75084), the Health Research Council (ETT) and TÁMOP (grants 4.2.1/B-09/1/KMR-2010-0001 and 4.2.2/B10/1-2010-0013)

**A role for exosomes from a human parasite in host cell pathogenesis and parasite communication**

Olivia Twu, Natalia de Miguel, Stephen Douglass, Hong Zhou, Matteo Pellegrini, James Wohlschlegel and Patricia Johnson

University of California-Los Angeles, Los Angeles, CA, USA

Email: oliviatwu@gmail.com

The human-infective, extracellular protozoan parasite *Trichomonas vaginalis* is responsible for the most prevalent non-viral STD. Attachment to host cells and secretion of virulence factors is believed to be vital to establishing infection. By proteomic analyses, we have discovered that *T. vaginalis* secretes exosomes that package proteins that could potentially be involved in pathogenesis. *T. vaginalis* exosomes also contain small RNAs and analyses are currently underway to determine whether these RNAs target the human host mRNAs. The identity or function of small RNAs has not previously been described in *T. vaginalis*; thus, our RNA analyses will provide the first glimpse into possible miRNA or siRNA populations in the parasite. Exosomes labeled with BODIPY-PC can transfer the label to vaginal epithelial cells (VECs) and experiments using split-GFP constructs verify that proteins packaged within the exosomes are delivered into the host cell. Furthermore, while exosomes induce proinflammatory IL-6 and IL-8 secretion from VECs, they specifically downregulate the IL-8 response of VECs to parasites. We have also demonstrated that exosomes from a highly pathogenic strain of *T. vaginalis* can increase the adherence to host cells of a weakly adherent strain via either parasite:host cell or parasite:parasite interaction. Experiments to determine exosome component(s) responsible for the observed immune effects and changes in parasite behavior are underway. In summary, this work indicates that there is a role for *T. vaginalis* exosomes in host cell pathogenesis and parasite communication.

Funded by: The National Institutes of Health

#### 2. Extracellular Vesicles in Malignancy

**Microvesicle-mediated reversal of the malignant phenotype in breast and prostate cancer**

Peter Quesenberry, Devasis Chatterjee, Sam Cross-Knorr, Michael DelTatto, David Mills and John Regan

Division of Hematology and Oncology, Rhode Island Hospital and the Warren Alpert Medical School of Brown University, Providence, RI, USA

Email: pquesenberry@lifespan.org

Epigenetic mechanisms of gene regulation collaborate with genetic alterations during cancer development. This is evident from every aspect of tumor biology including cell growth and differentiation, cell cycle control, DNA repair, angiogenesis and migration. In contrast to the genetic factors that promote cancer, other factors can reverse the cancer phenotype and potentially may provide new targets for therapeutic intervention.

Recent attention has been focused on the task of identifying soluble factors secreted by tumor cells that are responsible for epigenetic changes during cancer development. In addition to soluble paracrine factors, many tumor cells also release microvesicles (MVs). Interestingly, the abundance of MVs released generally correlates positively with advanced grade and stage of cancer progression. However, the mechanism triggering microvesicle generation by cancer cells is unknown. Nonetheless, there is mounting evidence that vesicle trafficking is a highly important process in cancer progression.

We have reported that biopsied prostate tumor cells co-cultured with human bone marrow (BM) cells induce expression of prostate specific genes. Significantly, we have demonstrated that we can reverse resistance and sensitize nonmalignant and malignant breast and prostate cells to the chemotherapy inducing effects of clinically relevant agents via MVs. In breast cancer, we can reverse the resistance of cells to doxorubicin, while, in prostate cancer, we can reverse the resistance to camptothecin via MVs as measured by apoptosis, cytotoxicity and growth in soft agar. The fact that MVs can elicit epigenetic changes presents an opportunity for the use of therapeutic agents to (A) block microvesicle release from cancer cells; (B) block the transfer of genetic material and/or agents, which may block non-malignant cells from accepting the MVs; and (C) accelerate the transfer of genetic material from MVs to malignant cells to inhibit tumor progression. Using stable isotope labeling with amino acids (SILAC) and antibody arrays, we have identified specific proteins that are responsible for the observed phenotypic changes. We are validating if these protein factors and the pathways they regulate can be targeted to improve therapeutic outcome in breast and prostate cancer.

Funded by: COBRE grant, NCRR Grant #P20RR025179, NIGMS Grant #8P20GM103468-04, 9/30/2009–6/30/2014.

**Understanding targeting/internalization mechanisms of exosomes derived from multiple myeloma cells**

K. Agarwal, N.D. Guzman, A. Rocci, F. Pichiorri and M.E. Paulaitis

Departments of Chemical & Biomolecular Engineering; Internal Medicine; Molecular Virology, Immunology & Medical Genetics, Ohio State University, Columbus, OH, USA

Email: agarwalkitty@gmail.com

Exosomes are small, membrane-encapsulated particles released by all cells that, in turn, can mediate intercellular communication by directly stimulating target cells. The uniqueness of exosome-mediated cell-cell communication depends on the ability of exosomes to interact specifically with their target cells. Exosome targeting and internalization mechanisms are, however, still not well understood. Exosomes can associate with cell membranes through ligand-receptor interactions or intermembrane lipid recognition. Internalization can occur through direct fusion of the exosomes with the cell membrane, leading to the release of exosomal content into the cell cytoplasm. Exosomes can also enter target cells by receptor-mediated endocytosis and later fuse with the limiting membrane of the endosome releasing the exosomal content for recycling to the cell surface or degradation in the lysosome. To gain insights into these targeting and internalization mechanisms, we characterized the biophysical and biochemical properties of exosomes secreted from multiple myeloma cell lines. Characterizations of size, morphology and mechanical properties are carried out using dynamic light scattering, asymmetrical flow field flow fractionation, nanoparticle tracking, cryotransmission electron microscopy and atomic force microscopy. Protein surface markers and total lipid analysis are carried out by mass spectroscopy to allow identification of the structural and regulatory elements of the exosomes. Finally, these properties are correlated with the propensity of exosomes to undergo fusion with supported membranes.

Funded by: U.S. National Science Foundation

**MicroRNA 21 in adipocyte-derived microvesicles confers taxol-resistance of ovarian cancer cells through downregulation of apoptotic protease activating factor 1**

Ngai Na Co, Rosemarie Schmandt, Karen H. Lu and Samuel C. Mok

Email: nnco@mdanderson.org

Obesity is one of the risk factors for ovarian cancer. To identify mediators that can support cancer growth in the ovarian cancer-modified omental microenvironment, Ion Torrent RNA sequencing and subsequent qRT-PCR analyses were used to identify non-coding microvesicle RNAs secreted by primary cultures of cancer-associated adipocytes (CAA), cancer-associated fibroblasts (CAF), normal adipocytes, normal fibroblasts and ovarian cancer cells. Of the RNAs identified, microRNA 21 (miR21) showed significantly higher expression in microvesicles secreted from CAA. Using qRT-PCR of RNA isolated from microdissected frozen tissue and in situ hybridization on FFPE tissue sections, CAA showed significant higher miR21 levels compared to the cancer cells and normal adipocytes. MiR21 was upregulated in omental ovarian cancer cells as compared to those in the primary tumor site. Moreover, strong miR21 staining was observed in omental cancer cells particularly at the interface, suggesting that miR21 may be transferred from CAA to their neighboring ovarian cancer cells. To test this hypothesis, SKOV3 ovarian cancer cells were co-cultured with adipocytes transfected with miR21-FAM or incubated with fluorescently labeled adipocyte-derived microvesicles. Confocal microscopy confirmed the delivery of miR21 by adipocyte-derived microvesicles to cancer cells. SKOV3 cells transfected with miR21 precursor showed an increase in cell survival and a decrease in apoptosis in the presence of taxol. Using microarray analysis of RNA isolated from SKOV3 cells transfected with the miR21 precursor, we identified a set of chemoresistance-related genes associated with miR21 expression. One of the most significantly downregulated genes is apoptotic protease activating factor 1 (APAF1). A strong miR21 binding site is predicted on the CDS of APAF1, suggesting APAF1 is the direct target of miR21. In summary, miR21 in adipocyte-derived microvesicles confers taxol-resistance by downregulating APAF1 expression in ovarian cancer cells to decrease taxol-induced apoptosis.

Funded by: Ann Schreiber Ovarian Cancer Research Training Program of Excellence from Ovarian Cancer Research Fund, NIH RO1CA133057 and MD Anderson Ovarian Cancer SPORE

**Role of Rab27 in exosome secretion: a means to understand their physiological functions?**

Angélique Bobrie^1^, Sophie Krumeich^1^, Matías Ostrowski^1^, Marina Colombo^1,2^, Graça Raposo^2^, Miguel Seabra^3^, Fabien Reyal^4^, Luis F. Moita^5^ and Clotilde Théry^1^

^1^Institut Curie, INSERM U932, Immunité et cancer, Paris, France; ^2^Institut Curie, CNRS UMR 144, Compartimentation et dynamique cellulaire, Paris, France; ^3^Molecular Medicine, National Heart and Lung Institute, Imperial College London, London, United Kingdom; ^4^Institut Curie, Département d'oncologie chirurgicale, Paris, France; ^5^Cell Biology of the Immune System Unit, Instituto de Medicina Molecular, Faculdade de Medicina, Universidade de Lisboa, Lisboa, Portugal

Email: angelique.bobrie@curie.fr

Exosomes are particular secreted vesicles produced by various cell types. They are formed in the endocytic pathway by inward budding of multivesicular bodies’ (MVBs) limiting membrane and released to the extracellular space when these compartments fuse with the plasma membrane. Exosomes purified from tumor cell lines or from cancer patients have been shown to modulate immune responses, inhibiting or enhancing them, depending on the experimental systems used and possibly on the actual nature of the vesicles studied. Thus, the role(s) played by tumor-derived exosomes (and other vesicles) in vivo remains unclear. We have previously shown that RAB27A and RAB27B are necessary for exosome secretion by HeLa cells. We here analyzed the roles of Rab27a and Rab27b in mouse tumor cells. Our analysis of tumors cells from different tissue origins shows variability of expression and function of Rab27a and Rab27b. We will describe the effects of inhibiting Rab27 in two mammary cancer cell lines on secretion of exosomes, other vesicles and soluble molecules. We will also discuss the outcomes in terms of tumor development and antitumor immune responses in vivo.

Funded by: French ministry of research and education

**Protein-protein interaction network of extracellular vesicles from human lung cancer cell lines**

Jae Young Hur, Do-young Choi and Kwang Pyo Kim

Department of Molecular Biotechnology, WCU Program, Konkuk University, Seoul, Republic of Korea

Email: kpkim@konkuk.ac.kr

Cancer cells shed extracellular vesicles (EVs), such as exosomes and microvesicles. EVs have various proteins, mRNAs and microRNAs that deliver their information to other cells. Therefore, EVs are very important suborganelle for cell to cell communication. However, their function and mechanism of biosynthesis are unclear. We analyzed vesicular proteins from PC9 human lung cancer cell and gefitinib-resistant PC9 cells (PC9/R) using mass spectrometry (MS). The gefitinib, which is a specific inhibitor of epidermal growth factor receptor (EGFR) tyrosine kinase, has been shown to suppress the activation of EGFR signaling essential for survival in non-small cell lung cancer (NSCLC) cell lines. Total of 1114 proteins and 963 proteins were identified from EVs isolated form PC9 and PC9/R cells, respectively. PC9/R cells had abundant caveolar-mediated endocytosis, integrin and PI3K-AKT signaling proteins compared to PC9 cells. Especially, human scaffold protein MP1 that regulates ERK signaling critical for shedding of EVs was identified and shown to control release of EVs from PC9/R cells. Our result demonstrated that PC9/R cells shed increased amount of EVs than PC9 cells as a result of increased expression of MP1 in PC9/R cells.

Funded by: The WCU program (Project No. R33-10128) and Functional Proteomics Project (FRP08A1-032) from the Korean Ministry of Education, Science and Technology

**Proteomic analysis of microvesicles released by the prostate cancer cell line PC-3**

Alicia Llorente and Kirsten Sandvig

Department of Biochemistry, Institute for Cancer Research, Oslo University Hospital, Oslo, Norway

Email: alillo@rr-research.no

Prostate cancer is one of the most frequent cancer types in men with 679,000 diagnoses and 220,000 deaths worldwide each year (1). There is a need for prostate cancer biomarkers, and a recent strategy to find them is based on microvesicles isolated from biological fluids (2). We have performed a proteomic analysis of the microvesicles released by the metastatic prostate cancer cell line PC-3 to identify potential prostate cancer biomarkers. Using nanocapillary liquid chromatography-tandem mass spectrometry, 266 proteins were identified with two or more peptide sequences. Further analysis showed that 16% of the proteins were classified as extracellular and that intracellular proteins were annotated in a variety of locations. Several of the proteins identified (tetraspanins, annexins, Rab proteins, integrins, heat shock proteins, cytoskeletal proteins and 14-3-3 proteins) have previously been found in microvesicles isolated from other sources (3). However, some of the proteins seem to be more specific to the vesicular population released by the metastatic prostate cancer PC-3 cell line. Interestingly, our results show that some of these proteins are promising biomarkers for prostate cancer and therefore candidates for clinical validation studies in biological fluids.

References

1. CANCERMondial http://www-dep.iarc.fr/, Globocan 2002.

2. Keller S, Ridinger J, Rupp AK, Janssen JW, Altevogt P. Body fluid derived exosomes as a novel template for clinical diagnostics. J Transl Med. 2011;9:86.

3. Mathivanan S, Fahner CJ, Reid GE, Simpson RJ. ExoCarta 2012: database of exosomal proteins, RNA and lipids. Nucl Acids Res. 2012 Jan;40(Database issue):D1241-4.

#### 3. Biomarkers and RNA

**Molecular tools for sensitive detection of microvesicles as biomarkers**

Masood Kamali-Moghaddam, J. Yan, D. Wu, J. Gu, R. Tavoosidana and U. Landegren

Department of Immunology, Genetics and Pathology, Science for Life Laboratory, Uppsala University, Uppsala, Sweden

Email: masood.kamali@genpat.uu.se

The possibility to detect and analyze proteins and other biomolecules in their biological environments with increased specificity and sensitivity will provide opportunities to use also very rare molecules as reliable biomarkers of diseases. We have recently developed a proximity ligation assay, where very high specificity and sensitivity of target molecule detection results from the requirement of multiple recognition events, combined with extremely high efficiency of signal detection due to amplification of DNA molecules that form in the detection reactions. In proximity ligation assays, affinity probes, such as antibodies, are attached to oligonucleotides to form proximity probes. Once a target molecule is recognized by a set of proximity probes, the DNA strands are connected to each other via enzymatic ligation to form a DNA template that can be amplified and quantified as the measure of the target molecule's concentration. Here, we illustrate the application of proximity ligation for sensitive detection of high-order protein complexes – such as microvesicles – as biomarkers for prostate cancer. The PLA technology provides tools with extreme sensitivity and specificity for screening and validation of protein biomarkers in cancers and other diseases.

Funded by: The Nordic Centre of Excellence in Disease Genetics, the Knut and Alice Wallenberg Foundation, Uppsala Berzelii Centre, the Swedish Research Council, Vinnova/Uppsala Bio and by the European Community's 6th and 7th Framework Programs

References

1. Darmanis S, et al. Mol Cell Proteomics. 2010 Feb;9(2):327–35

2. Tavoosidana G, et al. Proc Natl Acad Sci USA. 2011 May 24;108(21):8809–14.

**Identification of potential sequence motifs targeting RNA for secretion via exosomes using computational approaches**

Igor V. Kurochkin, Vladimir A. Kuznetsov and Arsen O. Batagov

Department of Genome and Gene Expression Data Analysis, Bioinformatics Institute, Singapore

Email: igork@bii.a-star.edu.sg

Exosomes contain numerous RNA species that are absent in their donor cells, implying the existence of a dedicated mechanism for the selective packaging of the RNAs into these vesicles. RNA localization depends on interactions between cis-acting elements in the RNA sequence and trans-acting factors, the RNA-binding proteins. Sequence motifs targeting RNA for secretion are currently unknown. We have applied ab initio approach for computational identification of potential RNA secretory motifs that does not require any prior knowledge of motif structure and is based on the comparison of primary sequence of secreted RNAs with that of cell-bound RNAs. An exhaustive motif search, which is not biased to neither positional nor multiple sequence context, revealed that, although there is no single motif specifically associated with exosomal RNAs, there exist combinations of multiple motifs within exosomal transcripts that are specific. We studied in detail one such combination of three 8-nt-long motifs confined to the 3'-end of exosomal RNAs. Interestingly, each of the three identified in this study motifs was predicted to form very similar secondary structures within different transcripts, suggesting that these secondary structure elements may serve as binding sites for cognate RNA-binding factors. Discovery of these motifs will be useful for selective targeting of RNAs of interest to exosomes to modulate the function of the recipient cells.

Funded by: Agency for Science, Technology and Research (A*STAR), Singapore

**Identification of human serum nanovesicles-associated microRNA signature**

Paola de Candia, Anna Torri, Francesco Marabita, Massimiliano Pagani and Sergio Abrignani

Istituto Nazionale Genetica Molecolare, Milan, Italy

Email: decandiapaola@ingm.it

MicroRNAs (miRNAs) are present in the bloodstream in a highly stable extracellular form. The existence of distinct circulating populations of miRNAs, associated to either membranous vesicles or protein complexes, may impact the identification of specific miRNAs as reliable markers of disease. Indeed, isolation procedures have been implemented as the first step in the search for such biomarkers, but there is still lack of consensus on the best method for purifying the nanovesicles from biological fluids. To address this issue, we have started from human serum and compared differential centrifugation and a new filtration-based nanovesicles purification kit. We have found that the two approaches give comparable results but that the filtration-based process requires lower quantities of biological material. We have also compared two different RT-qPCR arrays (Taqman Low Density Arrays by Applied Biosystems and microRNA Ready-to-Use PCR by EXIQON) in profiling the miRNome of serum nanovesicles and have found that 157 miRNAs were detected by at least one type of platform (90 by Applied Biosystems and 130 by EXIQON). This comparison led us to recognize specific skewing effects intrinsic to using a single platform and to identify biologically relevant microRNA signature associated to nanovesicles in sera of healthy donors.

Funded by: ERC, CARIPLO

**Detection of circulating miRNAs in colorectal cancer**

S.G. Jensen, D.K. Jeppesen, T.F. Ørntoft, M.S. Ostenfeld and C.L. Andersen

Department of Molecular Medicine, Aarhus University Hospital, Skejby, Aarhus N, Denmark

Email: steffen.grann@ki.au.dk

*Introduction*: It is expected that overall survival for colorectal cancer (CRC) can be substantially improved if more patients were detected at an earlier stage. Circulating tumor-derived exosomes contain miRNAs with biomarker potential to enable early detection of CRC. *Aim*: To create the basis for testing the hypothesis that miRNAs (exosome contained) can be used as biomarkers for early detection of CRC. Initially, this involves evaluation of methods for isolation of tumor exosomes and for profiling miRNAs with low abundance. *Methods*: Metastatic CRC cells SW620 were cultivated in a CELLine AD 1000 bioreactor. Conditioned cell culture media was harvested and ultracentrifuged to isolate secreted exosomes. The presence of exosomes was demonstrated by TEM and exosomes were quantified by Bradford protein assay. Dynabeads were used to isolate exosomes expressing EpCAM. WB was used to verify presence of EpCAM on the surface of exosomes and for confirmation of successful isolation of exosomes using dynabeads. *Results*: Bradford protein assay and TEM revealed successful isolation of CRC-derived exosomes. The mean size of exosomes was 60±19.7 nm. Western blot demonstrated the expression of EpCAM on the surface of exosomes and verified successful isolation of exosomes using dynabeads. Evaluation of miRNA expression profiling platforms from ABI, Exiqon and Affymetrix for detection of less abundant miRNAs revealed that the Exiqon platform had a better sensitivity and linearity. *Future perspectives*: Future work will focus on isolation of epithelial-derived plasma exosomes from CRC patients and healthy individuals and on profiling the miRNA content within these exosomes.

Funded by: The Danish National Advanced Technology Foundation, the John and Birthe Meyer Foundation, the Danish Council for Independent Research Medical Sciences, the Danish Council for Strategic Research and the Lundbeck Foundation.

**Glioma exosomal microRNAs**

Hélène Ipas^1,2^, Audrey Guttin^1–3^, François Berger^1,2^ and Jean-Paul Issartel^1–4^

^1^Team7 Nanomedicine and Brain, INSERM U836, Grenoble, France; ^2^Institut des Neurosciences, Université Joseph Fourier, Grenoble, France; ^3^Clinical Transcriptomics and Proteomics Facilities, Institut de Biologie et Pathologie, Centre Hospitalier Universitaire, Grenoble, France; ^4^CNRS, Grenoble, France

Email: helene.ipas@gmail.com

Brain glial tumors, in particular glioblastomas, are tumors with a very bad prognosis. Parameters controlling phenotypes as aggressiveness, migration or chemoresistance are not well known. In that tumor context, microRNAs (20-bp long endogenous non coding RNAs) can be considered as major actors of phenotype modification phenomenon as they play an important role in posttranscriptional gene silencing. Altered expression of these miRNAs can contribute to tumorogenesis and tumor development by deregulating the expression of key cancer genes. MicroRNAs are also precious diagnosis biomarkers, as shown in one of our recent study on tumor samples (1). In addition, it is important to notice that microRNAs are also secreted by glial tumors inside exosomes. Secreted glial microRNAs are believed to play a role in tumor development as they are delivered into normal cells, via an exosome mediated-transfer, and can alter specific functions of those targeted normal cells in close vicinity to the tumor. The microRNA contents of the glial exosomes and of their secreting cells should be characterized in order to understand the important physiopathological phenomenon happening in the glial tumors. Transcriptomic molecular analyses (messenger RNAs and microRNAs) are performed by hybridization on DNA chips and by quantitative RT-PCR. This should allow therapeutic and diagnosis applications.

Funded by: INSERM Ligue Contre le Cancer (Ligue Nationale et Départementales, Ardèche, Drôme, Savoie)

**Reference**

1. Lages E, et al. PLoS One. 2011;6(5):e20600. Epub 2011 May 31.

### Coffee and Poster Session 2          10.00-12.00

### Lunch and Poster Viewing          12.00-13.00

### Symposium Session 8          13.00-15.00

#### 1. Immunology and Inflammation

**Activation of potent cytotoxic T cells after exosome immunization is dependent on CD4^+^ T cells and B cells**

Tanja I. Näslund, Ulf Gehrmann, Khaleda R. Qazi, Mikael C.I. Karlsson and Susanne Gabrielsson

Karolinska Institutet, Department of Medicine, Translational Immunology Unit L2:04, Karolinska University Hospital Solna, Stockholm, Sweden

Email: Tanja.Naslund@ki.se

Exosomes are secreted membrane nanovesicles of endosomal origin and are considered as potential cancer vaccine vectors. Phase I clinical trials have been successfully conducted with tumor peptide-loaded exosomes derived from dendritic cells (DC-Exos) and a phase II clinical trial is currently ongoing. However, much is still unknown regarding how DC-Exos exert their function in vivo and if the immunogenicity of DC-Exos can be enhanced. We have recently reported that DC-Exos induce CD4^+^ T-cell responses in a B cell-dependent manner, suggesting that immunizing with exosomes only carrying T cell peptides induce suboptimal immune responses. In this study, we show that CD8^+^ T-cell responses were induced in vivo when mice were immunized with protein, but not peptide, loaded DC-Exos. We could also show that the cytotoxic T-cell response was totally dependent on CD4^+^ T cells and, interestingly, also on B cells. Finally, mice deficient in complement activation and antigen shuttling by B cells also have lower responses to protein-loaded DC-Exos showing involvement of these B cell-mediated mechanisms. In conclusion, when designing cancer vaccines, proper activation of CD4^+^ T and B cells need to be considered to ensure full potential of the treatment.

Funded by: The Swedish Research Council, Torsten Söderberg foundation, Swedish Cancer foundation, Swedish Medical Society, King Gustaf V's 80-years foundation, Magnus Bergvall foundation, Swedish Heart-Lung Foundation, Hesselman's Foundation, KI IMTAC theme center and David and Astrid Hageléns Foundation

**Platelet-derived ectosomes promote the induction of FOXP3+regulatory cells from activated CD4^+^ T cells**

Salima Sadallah, Francesca Amicarella, Ceylan Eken, Giandomenica Iezzi and Jurg A. Schifferli

Immunonephrology Laboratory, Department of Biomedicine and Institute of Surgical Research, University Hospital Basel, Basel, Switzerland

Email: salima.sadallah@unibas.ch

During storage platelets shed vesicles by ectocytosis called ectosomes. Platelet-derived ectosomes (PLT-Ect) express phosphatidylserine and are able to inhibit the inflammatory response of activated macrophages, thus dampening the generation of adaptive immunity. Whether PLT-Ect may also exert a direct effect on T cells remains to be addressed. In this study, we analyzed the in vitro effects of PLT-Ect on activation and differentiation of human CD4^+^ T cells. Activation of CD4^+^ T cells, isolated from peripheral blood of healthy donors, in the presence of PLT-Ect resulted in increased frequencies of CD25high FOXP3+regulatory T cells (Tregs), in a dose- and time-dependent manner. The increase in Tregs was accompanied by a decrease of CD4^+^ T cell proliferation and of IFN-? and IL-10 production. This effect was not observed when PMN- and erythrocyte-derived ectosomes were analyzed in the same conditions. To identify which CD4^+^ T cell subsets were influenced by PLT-Ect, we investigated the effect of PLT-Ect on sorted CD45RA+CD62L+naïve, CD45RA−CD62L+/− memory and CD25high Treg subsets. We found that PLT-Ect induced both differentiation of naïve T cells and conversion of memory T cells into CD25high FOXP3+T cells but had no effect on predifferentiated CD25high FOXP3+Tregs. Preexposure of PLT-Ect to TGF--neutralizing antibodies partially inhibited Treg induction, thus indicating that TGF- expressed by PLT-Ect was involved in these phenomena. Finally, when we tested the putative suppressive function of FOXP3+cells differentiated upon exposure to PLT-Ect on CD8+T cells, they were found to suppress CD8^+^ T cells proliferation to a similar extent to that of Tregs. By promoting the generation of suppressive CD4^+^ T cells, PLT-Ect might contribute to peripheral tolerance. The induction of peripheral blood Treg might occur in patients receiving PLT concentrates, which contain PLT-Ect.

Funded by: Swiss National Science Foundation

**CD4+CD25+FoxP3+T cells produce exosomes with antigen-specific regulatory function: a novel mechanism for suppression**

Lesley Ann Smyth^1^, Kulachelvy Ratnasothy^1^, Julia Y.S. Tsang^2^, Alice Warley^3^, Robert Lechler^1^ and Giovanna Lombardi^1^

^1^MRC Centre for Transplantation, King's College London, Guy's Hospital, London, United Kingdom; ^2^Research Center of Infection and Immunity, University of Hong Kong, Hong Kong SAR, China; ^3^Centre for Ultrastructural Imaging, King's College London, Guy's Campus, London, United Kingdom

Email: lesley.smyth@kcl.ac.uk

Recently, it has been shown that activated T cells can produce exosomes with immune modulating properties both in vitro and in vivo. We addressed whether activated murine CD4+25+FoxP3+T regulatory cells are also capable of producing such structures following TCR activation with the view that this may be a novel pathway by which regulatory T cells function. TCR activation of both freshly isolated CD4+25+FoxP3+and T regulatory cells lines induced the production of exosome-like structures as defined by electron microscopy and flow cytometry. Exosomes expressed the tetraspan molecule LAMP-1/CD63, as well as CD4, CD25, MHC class I, CD39 and CD73. As predicted, we observed that regulatory T cell-derived exosomes were capable of inhibiting both polyclonal and antigen-specific CD4^+^ T-cell responses in vitro. Possible mechanism by which these structures inhibit CD4^+^ T-cell responses are through the expression of high levels of CD25 as well as CD73 molecules or by modifying antigen-presenting cells. From our observations, we suggest that exosomes produce by regulatory T cells may amplify the suppressive capacity of these cells within the local environment.

Funded by: British Heart Foundation

**T cells release different nanosized vesicle subsets depending on their activation status**

Els J. van der Vlist^1^, Ger J.A. Arkesteijn^2^, Chris H.A. van der Lest^1^, Willem Stoorvogel, Esther N.M. Nolte 't Hoen^1^ and Marca H.M. Wauben

^1^Department of Biochemistry & Cell Biology, Utrecht University, Faculty of Veterinary Medicine, Utrecht, The Netherlands; ^2^Department of Infectious Diseases & Immunology, Utrecht University, Faculty of Veterinary Medicine, Utrecht, The Netherlands

Email: e.j.vandervlist@uu.nl

CD4^+^ T cells are important effectors and modulators of the adaptive immune response. Vesicles actively released by CD4^+^ T cells may play a role in the activation or downregulation of immune responses. We investigated how T-cell receptor (TCR)-triggering and co-stimulation signals influence the quantity and heterogeneity of nanosized vesicles released by T cells. We performed quantitative and qualitative analyses of T cell-derived vesicles, using a novel high-resolution flow cytometry-based method. In contrast to bulk-analysis methods, this method allows the analysis of individual nanosized vesicles and gives insight in both the actual number of vesicles and the heterogeneity of the vesicle population. We found that TCR-triggering increased the number of released nanosized vesicles within the “typical exosomal” buoyant density range in sucrose (1.10–1.19 g/ml) and that additional co-stimulation had a potentiating effect on vesicle release. However, the increase in the amount of vesicles varied substantially within the different fractions in the exosomal buoyant density range. Detailed analysis of significantly increased vesicle populations (i.e., sucrose fractions 1.14 and 1.17 g/ml) based on fluorescence and light scattering revealed that each fraction contained three different vesicle subpopulations. Although all three subpopulations increased upon T cell activation by TCR-triggering and co-stimulation, one subpopulation was significantly increased over others. These data show that T cells release a heterogeneous population of nanosized vesicles and indicate that T cells differentially regulate the release of distinct vesicle subpopulations depending on their activation status.

Funded by: Utrecht University

**Enzymatically modified low density lipoproteins (eLDL) and free fatty acid liposomes strongly induce extracellular vesicle (EV) release and cholesterylester storage in human polymorphonucleated neutrophils (PMNs) with major impact on their viability**

Markus Peer, Stefan Wallner, Evelyn Orsó, Alexander Sigruener and Gerd Schmitz

Institute for Laboratory and Transfusion Medicine, University Hospital Regensburg, Germany

Email: markus.peer@klinik.uni-regensburg.de

Multiomics profiling of isolated human blood monocytes, in vitro differentiated macrophages and PMN exposed to eLDL and copper oxidized LDL (oxLDL), as well as NEFA-liposomes has been conducted. PMN upon eLDL and NEFA exposure, in contrast to oxLDL, lead to a much stronger and faster cellular increase of free cholesterol (FC) and cholesterylesters (CE), ceramide (Cer) and lysophosphatidylcholine (LPC) species, in comparison to macrophages. In parallel, a dramatic shedding of EVs and a drop in the viability of PMN is observed. PMN-derived EVs are chemotactic for monocytes, induce apoptosis and necrosis in a concentration-dependent manner, leading to differential surface marker expression promoting an inflammatory M1 macrophage phenotype. The prominent in vitro eLDL response of PMN, in contrast to macrophages, may be associated with the high NEFA and LPC content of eLDL particles, acting as ligands for the FA-receptors GPR40/41/43 or lysophospholipid (LPC, LPA) GPRs coupled to Ca^2+^ and MAPK-dependent secretion of PMN-derived EVs. These EVs possibly enter the vascular lesion while the PMN-remnants are cleared by the spleen. This may provide an explanation for the paucity of PMN in contrast to the predominance of foamy macrophages in atherosclerotic lesions and is consistent with the observation that PMN-depletion counteracts atherogenesis.

Funded by: LipidomicNet, EU-Project FP7

**Activation of autoreactive T and B cells by insulinoma-derived exosomes in autoimmune-prone, non-obese diabetic mice**

Yang D. Dai

Torrey Pines Institute for Molecular Studies, San Diego, CA, USA

Email: ydai@tpims.org

A tumor-derived exosomes can act as endogenous adjuvant and/or an antigen carrier to initiate immune responses. To address whether organ-specific autoimmune disease could be initiated following abnormal exosome production in the target cells or tissues, we studied immune responses in non-obese diabetic (NOD) mice, a model for human type 1 diabetes (T1D), in which over 80% of female mice spontaneously develop diabetes at young age. Exosomes collected from insulinoma cells are strongly immunostimulatory and require MyD88-mediated innate TLR signaling pathways to induce secretion of several proinflammatory cytokines including IL-6, MCP-1, IFN-gamma and TNF-alpha. Accordingly, antigen presenting cells are activated as detected by increased expression of class II MHC and B7 costimulatory molecules on dendritic cells. Importantly, the exosomes activated a subpopulation of marginal zone-like B cells, independent of T cell help, which are expanded in the prediabetic NOD mice. In the pancreatic draining lymph nodes of these mice, exosome-reactive, IFN-gamma-secreting Th1 cells were also expanded, indicating that exosomes may induce tissue/antigen-specific immune response. Finally, injecting exosomes into a diabetes-resistant mouse strain, but carrying the diabetes-susceptible MHC allele, accelerated development of insulitis; however, progression from insulitis to complete destruction of pancreatic islets or diabetes apparently requires additional susceptible genes to further compromise immune regulatory system. Thus, abnormal secretion of intracellular organelles such as exosomes can stimulate immune responses, innate as well as adaptive and may result in autoimmune disease in genetically susceptible individuals.

Funded by: NIH (National Institutes of Health) DNRG (Diabetes National Research Group)

**Neutrophil-derived ectosomes promote resolution of inflammation by altering inflammasome activation, autophagy induction and apoptosis**

Arun Cumpelik and Juerg A. Schifferli

Immunonephrology laboratory, Department of Biomedicine, University Hospital Basel, Basel, Switzerland

Email: arun.cumpelik@unibas.ch

Whereas a lot is known about how inflammation is initiated, very little is known about how inflammatory responses are terminated once the initiating trigger has been brought under control. Upon activation neutrophils rapidly release large numbers of ectosomes (PMN-Ecto) from their surface. Our group has previously shown that PMN-Ecto have potent anti-inflammatory effects when co-incubated with TLR-activated human monocyte-derived macrophages (HMDM) in vitro. We hypothesized that early release of PMN-Ecto during inflammation might alter central pathways of inflammation and cell survival and thereby contain inflammatory responses. We found that PMN-Ecto impair inflammasome activation. LPS-primed HMDM stimulated with a range of NALP3 inflammasome stimuli failed to release IL-1β when co-incubated with PMN-Ecto. PMN-Ecto suppressed IL-1β release in part by limiting the amounts of pro-IL1β available for cleavage by the active caspase-1. This effect could be explained by an increase in autophagy characterized by elevated levels of LC3-II protein and LC3-positive autophagosomal clusters in HMDM following co-incubation with PMN-Ecto. PMN-Ecto-conditioned cells were also found to be resistant to apoptosis. In the presence PMN-Ecto, HMDM treated with staurosporine showed less caspase-3 activation. Taken together, these results offer various new mechanisms of how the early release of PMN-Ecto during an inflammatory response might autoregulate and limit inflammation. Furthermore, these results underscore how central cellular pathways of inflammation and survival are interrelated.

**Microparticles from mice with monocrotaline-induced pulmonary hypertension induce right ventricular hypertrophy and pulmonary vascular remodeling in healthy mice**

Jason M. Aliotta^1,2^, Rabih El-Bizri^1^, Mandy Pereira^2^, Ashley Amaral^2^, Alex Hasslinger^2^, Zenas Igbinoba^2^, Peter J. Quesenberry^2^ and James R. Klinger^1^

^1^Division of Pulmonary, Critical Care and Sleep Medicine; ^2^Division of Hematology and Oncology, Department of Medicine, Rhode Island Hospital, Warren Alpert Medical School of Brown University, Providence, RI

*Rationale:* Circulating endothelial cell-derived microparticles (MPs) are increased in pulmonary hypertension (PH) but whether they are biomarkers of cellular injury or participants in disease pathogenesis is unknown. We have shown that murine lung-derived MPs (LMPs) alter marrow cell phenotype by inducing expression of lung-specific mRNA and protein. We hypothesize that LMPs and plasma-derived MPs (PMPs) from mice with monocrotaline (MCT)-induced PH alter pulmonary vascular endothelial and smooth muscle cell phenotypes or marrow progenitor cell phenotype to induce features of PH in normal mice. *Methods:* LMPs and PMPs were isolated from C57BL/6 mice 7 days after MCT (600 mg/kg weekly for 4 weeks) or vehicle injections. PMPs from each MCT or vehicle-injected mouse were injected into a healthy mouse (three equal doses every other day times three). LMPs from each MCT or vehicle-injected mouse were injected into three healthy mice (same schedule). Recipients were sacrificed 2, 4 and 6 weeks later (three/time point). Right ventricular (RV) hypertrophy was assessed by RV-to-body weight (RV/BW) ratio (mg/g) and pulmonary vascular remodeling by blood vessel wall thickness-to-diameter (WT/D) ratio. MPs from donors were analyzed by microarray focusing on 20 genes that have been implicated in pulmonary vascular remodeling. *Results:* At the time of MP harvest, RV/BW and WT/D ratios were elevated in mice injected with MCT vs. vehicle (0.219+0.005 vs. 0.103+0.004 mg/g; 0.149+0.021 vs. 0.062+0.009%, *p*<0.05). There was no difference in the number of LMPs or PMPs isolated from MCT-injured or vehicle mice; however, qualitative differences were seen. LMPs, but not PMPs, from MCT-injured mice had higher endothelial cell mRNA expression (PECAM-1, E-selectin, endoglin, CD143 and VE-cadherin) and higher expression of mRNAs known to be abnormal in PH (PDGF, BNPR2 and eNOS) vs. vehicle mouse MPs. RV/BW ratios were higher in mice injected with LMPs and PMPs from MCT-injured mice than those injected with vehicle mouse MPs 4 weeks (0.172+0.006 vs. 0.134+0.004 mg/g for LMPs, 0.156+0.008 vs. 0.111+0.004 for PMPs, *p*<0.05) and 6 weeks after injection (0.165+0.009 vs. 0.111+0.012 mg/g for LMPs, 0.154+0.008 vs. 0.096+0.003 for PMPs, *p*<0.05). WT/D ratios were higher in mice injected with LMPs and PMPs from MCT-injured mice than those injected with vehicle mouse MPs 6 weeks after injection (0.103+0.008 vs. 0.078+0.006% for LMPs, 0.110+0.007 vs. 0.066+0.004 for PMPs, *p*<0.05). *Conclusions:* MPs from MCT-injured mice induce RV hypertrophy and pulmonary vascular remodeling when injected into healthy mice. We conclude that MPs may play an important role in the pathogenesis of MCT-induced PH.

#### 2. Immunology and Cancer

**Dendritic cell-derived membrane vesicle vaccine: induction of cytotoxic T lymphocytes cross-priming for tumor rejection**

Xin Tian, Motao Zhu, Yanhua Tian, Xiao Yang, Xiao Song, Yuliang Zhao and Guangjun Nie

CAS Key Lab for Biomedical Effects of Nanomaterials and Nanosafety, National Center for Nanoscience and Technology, Beijing, China

Email: tianx@nanoctr.cn

Tumor vaccine is based on initiation of T-cell-mediated antitumor responses by recognition of tumor-specific antigens. Previous studies have suggested that tumor-derived exosomes, containing tumor relevant antigens, can induce T-cell-mediated antitumor effects on syngeneic and allogeneic established mouse tumors. Here we examine the ability of dendritic cells-derived membrane vesicles (DC-mvs) presenting antigens from two different types of tumor (murine B16 melanoma and murine Lewis lung carcinoma) in stimulating protective T-cell responses among those tumors. We found that DC-mv loaded antigens from two tumors could induce cytotoxic T lymphocytes (CTL)-dependent tumor rejection and suppress the growth of both types of tumor in a murine xenograft tumor model. In addition, initiation of CTL by those DC-mvs presented cross-priming in executing their antitumor effect. DC-mv carrying antigens from two types of tumor have synergistic antitumor activity comparing to those carrying antigens from only one. Our studies suggest that DC-mv containing antigens of different tumors have potential application as a highly effective, versatile cell-free vaccine for the induction of T-cell cross-priming against a variety of tumor types.

Funded by: National Basic Research Program of China (973 program: 2011CB933401; 2012CB934000), National High Technology Research and Development Program (863 program: 2009AA03Z335), National Natural Science Foundation of China (31100721; 10979011; 30900278)

**Immune modulatory properties of glioblastoma multiforme exosomes**

Jeroen de Vrij, Kitty M.C. Kwappenberg, Sybren L.N. Maas, Anne Kleijn, Martine L. Lamfers, Clemens M.F. Dirven, Marco W. Schilham and Marike L.D. Broekman

Department of Neurosurgery, University Medical Center Utrecht, The Netherlands; Department of Neurosurgery, Erasmus Medical Center Rotterdam, The Netherlands; Department of Pediatrics, Leiden University Medical Center, The Netherlands

Email: j.devrij@erasmusmc.nl

Glioblastoma multiforme (GBM) is the most common and most aggressive primary brain tumor in humans. It was recently shown that GBM cells secrete tumor exosomes (TEX) that can stimulate blood vessel formation and tumor cell proliferation in cell culture. We hypothesized on an additional tumor-supportive role of GBM TEX through influencing cells of the immune system. For unknown reasons, GBM patients display a variety of abnormalities in their immune system, which presumably contributes to the highly aggressive nature of GBM tumors. To investigate the immune modulatory properties of GBM TEX, we isolated TEX from the supernatant of GBM cell lines and primary tumor cultures. Extensive quality control analysis was performed on the TEX isolates, including quantification with Sensing Ion Occlusion Sensing (SIOS) technology. Next, peripheral blood mononuclear cells (PBMCs) from healthy donors were exposed (for 3 days) to the TEX, followed by flow cytometric analyses on all PBMC cell types. The incubation of PBMCs with TEX appeared to result in a profound increase in CD14 expression and a decrease in HLA-DR expression on monocytes. The effect on monocytes was also observed on purified monocytes, indicating a direct effect. These results were shown to correspond to changes in the phenotype of monocytes in peripheral blood of GBM patients and suggest an important immune modulatory role for brain tumor exosomes.

Funded by: Hersenstichting (Brain foundation) Nederland

**Tumor-exosomes and leukocyte activation: an ambivalent crosstalk**

Daniela Zech, Sanyukta Rana, Markus W. Büchler and Margot Zöller

Department of Tumor Cell Biology, University Hospital of Surgery, Heidelberg, Germany

Email: danielaz88@aol.com

Dendritic cell (DC)-derived exosomes induce whereas tumor-exosomes might suppress an immune response. We here explored how exosomes from the rat pancreatic adenocarcinoma BSp73ASML (ASML) affect tumor-reactive leukocyte activation and migration and whether a potential negative impact of tumor-exosomes can be circumvented. ASML-exosomes bind to and are taken up by all leukocyte subpopulations in vivo and in vitro, uptake by CD11b+leukocytes exceeding that by T and B cells. ASML-exosomes affect leukocyte proliferation likely via reduced CD44v6 upregulation and lck, ZAP70 and ERK1,2 phosphorylation, which, however, can be compensated by DC. ASML-exosomes do not support Treg or MDSC expansion. Yet, apoptosis susceptibility is slightly increased, which relies on impaired activation of antiapoptotic signals without alterations of receptor or mitochondrial caspase activation. IgM secretion is unaffected and NK and CTL activity are strengthened, ASML-exosomes co-operating with DC in CTL activation. ASML-exosomes transiently interfere with leukocyte migration by occupying during binding/internalization the migration-promoting receptors CD44, CD49d, CD62L and CD54. ASML-exosomes having a minor impact on leukocyte activation, which can be overridden by DC, but supporting leukocyte effector functions could well serve as an adjuvant in immunotherapy. Notably, exosome-induced modulation of immune cells relied on exosome uptake rather than binding, which points toward an important contribution of transferred messages like proteins, mRNA and miRNA. Thus, depending on the exosome composition, different tumor-exosomes may distinctly affect the immune system, which easily can be explored in vitro in advance of taking tumor-exosomes as an adjuvant.

Funded by: Deutsche Krebshilfe

**Transformative model of prostate cancer shows tumor microvesicle suppression of natural killer cell activity**

John L. Reagan, Dong Qin Yang, Martina Srajer Gajdosik, Lulu Cao, Dionysios Pantazatos, Kate E. Brilliant, Loren Fast, Peter J. Quesenberry, Douglas C. Hixson and David R. Mills

Department of Medicine, Division of Hematology and Oncology, Rhode Island Hospital/The Warren Alpert, Medical School of Brown University, Providence, RI, USA

Email: jreagan@lifespan.org

*Background:* Recent studies have described the effect of tumor-derived microvesicles (MVs) on the amplification of T regulatory cells and the induction of apoptosis in CD8^+^ T cells, thereby postulating a role for tumor-derived MVs in tumor tolerance. Here, we describe the effect of rat prostate tumor derived MVs on natural killer (NK) cell activity. *Methods:* A transformed rat prostate epithelial cell (PEC) line has been developed within our laboratory. Cells were isolated from dorsolateral prostate lobes from mature Fisher 344 rats without prior carcinogen treatment or immortalization. This model culminates at high passage (p>85) in anchorage-independent growth when plated in soft agar and tumorigenicity when injected into immunodeficient mice. Low passage (p<35) and intermediate passage (p36–84) cells do not demonstrate this phenotype. MVs were isolated from conditioned media following 3–4 days incubation with low or high passage PEC. Negatively selected splenic NK cells were isolated from Fischer rats using antibodies to remove B cells, T cells, monocytes, macrophages and granulocytes. FACS showed approximately 90% enrichment for NK cells. NK cells were incubated in the presence or absence of microvesicles for 1 hour at 37°C and subsequent NK cytolytic activity was measured against YAC-1 tumor cells using a standard 4-hour 51Cr-release assay. Suspensions of effector cells at effector to target (E:T) ratios of 10:1, 5:1, 2.5:1 and 1.25:1 were plated in triplicate. *Results:* Using a two tailed Mann-Whitney *U* test, a statistically significant decrease in NK cytolytic activity was observed in the 2.5:1, 5:1 and 10:1 E:T groups treated with high pass-derived MVs as compared to untreated NK cells (*p*=0.03, *n*=4). The lowest E:T ratio in this setting (1.25:1) trended toward statistical significance (*p*=0.06). In contrast, low passage-derived microvesicles had no effect on NK cytolytic activity (*p* range 0.2–1, n=3). *Conclusions:* The preincubation of rat splenic NK cells with transformed high passage PEC-derived MVs significantly suppressed NK-mediated cytotoxicity. In contrast, membrane vesicles from low passage PEC had no effect on cytotoxicity. These findings have the potential to explain, in part, how tumors evolve mechanisms to evade NK immune surveillance.

Funded by: COBRE grant 1P20RR17695

**Suppression of tumor antigen-specific immune responses by both tumor-derived and endogenous, plasma-derived MHCII+exosomes in tumor-bearing mice**

Paul Robbins, Chenjie Yang, Seon hee Kim, Nicole Bianco and Melanie Ruffner

Department of Microbiology and Molecular Genetics, University of Pittsburgh School of Medicine, Pittsburgh, PA, USA

Email: probb@pitt.edu

Tumor-specific immunosuppression is frequently observed in tumor-bearing hosts. We have examined the role of tumor-derived exosomes in suppressing the antitumor immune response. We used a mouse model of delayed-type hypersensitivity (DTH) to demonstrate that local administration of tumor-derived exosomes carrying the model antigen chicken ovalbumin (OVA) resulted in the suppression of the DTH response in an antigen-specific manner. Analysis of exosome trafficking demonstrated that following local injection, tumor-derived exosomes were internalized by CD11c+cells and transported to the draining LN. Exosome-mediated DTH suppression is associated with increased mRNA levels of TGF-1 and IL-4 in the draining LN. The tumor-derived exosomes also were able to inhibit DC maturation. These results suggest a role for tumor-derived exosomes in inducing tumor antigen-specific immunosuppression, possibly by modulating APC function. We also demonstrated that plasma-derived exosomes isolated from mice bearing OVA-expressing tumors, but not from naïve mice or mice bearing OVA-negative tumors, were able to suppress the OVA-specific DTH immune response. Depletion of MHC class II+vesicles from the plasma-derived exosomes of mice bearing OVA-expressing tumor resulted in significant abrogation of the antigen-specific suppressive effect. These results suggest that endogenous MHC Class II+exosomes in the blood circulation of tumor-bearing hosts also are able to suppress the tumor antigen-specific immune response. A model for the role of both tumor- and host-derived exosomes in suppressing the antitumor immune response will be presented.

Funded by: Department of Defense grants 17-03-1-0488 and 17-03-0412 and grants NS058451, AG024827, AG033907 and AR051456 from the National Institutes of Health to P.R.

**Prostate cancer-derived exosomes as a mechanism of immune evasion**

Marie Lundholm and Pernilla Wikström

Department of Medical Biosciences, Pathology, Umeå University, Umeå, Sweden

Email: marie.lundholm@medbio.umu.se

Exosomes are endosome-derived vesicles of 50–100 nm size, which are actively secreted by a wide range of cell types. Tumor cells have been shown to release higher amounts of exosomes than their normal counterparts, and several studies have suggested that tumor exosomes mediate immune-suppressive effects. In this study, we have investigated if prostate cancer (PCa)-derived exosomes are able to downregulate expression of the activating receptor NKG2D in NK and CD8^+^ T cells, thereby impairing effector cytotoxic functions. Moreover, we have examined if these exosomes can induce Fas-mediated apoptosis in activated lymphocytes. By using flow cytometry analysis, we show that exosomes derived from the human PCa cell line, 22Rv1, express NKG2D ligands and the proapoptotic protein FasL. We demonstrate that these 22Rv1 exosomes induce downregulation of the NKG2D receptor on NK and CD8^+^ T cells and exert Fas-mediated apoptosis in activated lymphocytes. In addition, plasma-derived exosomes from patients with castration-resistant PCa (CRPC) triggered downregulation of NKG2D expression in NK and CD8^+^ T cells. These findings suggest that exosome-mediated downmodulation of NKG2D receptor in effector cells and Fas-mediated apoptosis in activated lymphocytes are possible mechanisms of immune evasion in PCa.

**Nasopharyngeal carcinoma-derived exosomes recruit, expand and upregulate biological activities of human regulatory T cells (Treg)**

Dhafer Mrizak, Nathalie Martin, Niels Wambre, Laurissa Ouaguia, Yvan de Launoit, Pierre Busson, Olivier Morales and Nadira Delhem

Institut de Biologie de Lille, Immunorégulation des Cancers Viro-Induits, Lille-CEDEX, France

Email: dhafer.mrizak@ibl.fr

Nasopharyngeal carcinoma cells release exosomes in high levels, which present immunosuppressive properties, into body fluids that might be involved in tumor progression. The frequency and suppressor functions of CD4+CD25highCD127lowFoxP3+regulatory T cells (Treg) are also higher in nasopharyngeal carcinoma patients (NPC) than healthy donors. As interactions between NPC-derived exosomes and Treg remains unknown, we investigate here their ability to induce, expand and activate human Treg.

Exosomes isolated from supernatants of NPC cell lines but not from serum of healthy donors induced the generation of Tim3low Treg, enhanced their associated phenotypic markers and their expansion. NPC exosomes strengthened the suppression of responder cell proliferation and also mediated conversion of CD4+CD25- T cells into Treg. Interestingly, exosomes also take part of the recruitment of purified Treg in a CCL20-dependent manner. Our results thus give new insights about NPC-derived exosomes immunoregulatory properties. They induce Treg expansion, upregulate Treg suppressor function, enhance Treg chemoattraction and promote the conversion of CD4+CD25- T cells into Treg. Interactions of NPC-derived exosomes with CD4+regulatory T cells represent a newly defined mechanism that might be involved in regulating peripheral tolerance by tumor cells and in supporting immune evasion of human NPC.

Funded by: CNRS

**Exosomes loaded with α-galactosylceramide amplify antitumor immunity via iNKT cells**

Ulf Gehrmann, Stefanie Hiltbrunner, Mikael C.I. Karlsson, Tanja I. Näslund and Susanne Gabrielsson

Translational Immunology Unit, Department of Medicine, Solna, Karolinska Institutet, Stockholm, Sweden

Email: ulf.gehrmann@ki.se

Exosomes are nanosized membrane vesicles with endosomal origin that can be isolated from a variety of cell types and serve as means of communication between cells. Dendritic cell (DC)-derived exosomes can induce adaptive as well as innate immune responses and have been tested as therapeutic agents in cancer. Here, we investigated whether exosomes can be loaded with the iNKT cell activating ligand α-galactosylceramide (αGC) and whether exosomes loaded with both αGC and the protein antigen ovalbumin (OVA) could amplify both innate and adaptive immune responses. We show that DC-derived exosomes express CD1d and that αGC-loaded exosomes (Exo-αGC) activate iNKT cells in vitro in a CD1d-dependent manner. In vivo, αGC- and OVA-loaded exosomes [Exo(αGC–OVA)] induced NK cell and ?d T cell responses secondary to iNKT cell activation and increased OVA-specific T- and B-cell responses. Moreover, treatment of tumor-bearing mice with Exo(αGC–OVA) slowed down tumor growth and led to immune cell infiltration into tumor tissue in a OVA-expressing mouse melanoma model. Our results show that activation of iNKT cells by exosomes amplifies both innate and adaptive immune responses in a way that might be beneficial for cancer immunotherapy.

Funded by: Swedish Research Council, Swedish Heart-Lung Foundation, Swedish Cancer Society

#### 3. Isolation Methods

**CD36 positive microparticles in plasma are derived from several cell types**

Morten Hjuler Nielsen, Henning Beck-Nielsen and Aase Handberg

Danish PhD School of Molecular Metabolism, Department of Clinical Biochemistry, Aalborg University Hospital, University of Southern Denmark, Denmark

Email: mhn@mb.au.dk

The scavenger receptor CD36 belongs to a family of cell surface receptors involved in lipid uptake and inflammation. CD36 has recently been identified in cell-free plasma. Plasma CD36 measured by ELISA-technique is elevated in patients with type 2 diabetes as well as in other conditions with an elevated risk of cardiovascular disease and in patients with unstable atherosclerotic plaques. Plasma CD36 may partially be associated with microparticles (MPs), and the aim of this study was to set up a method for identification and quantification of CD36-positive MPs derived from platelets, monocytes and endothelial cells.

*Methods:* For the detection of MPs, an initial microparticle-size gate was set using a blend of size-calibrated fluorescent beads, ranging in size from 0.2 (Invitrogen), 0.5, 0.9 and 3.0 μm (Megamix, Biocytex). MPs were identified according to their size (ranging from 0.1 to 1.0 μm) and by Lactadherin-fluorescein isothiocyanate labeling. TruCount beads (BD Biosciences) were used for quantification. Endothelial, monocyte and platelet MPs were defined as CD31+/CD42b-, CD14+and CD41+particles, respectively. CD36-positive MPs were identified by a PE-labeled anti-CD36 antibody. MPs were analyzed by a Becton-Dickinson FACSAriaTM flow cytometer and Flowjo software. *Results:* We have identified platelet-derived, endothelial cell-derived and to a lesser degree monocyte-derived CD36-positive MPs in human plasma. *Conclusions:* Our data underline the presence of CD36-positive MPs, derived from platelets, monocytes and endothelial cells, in plasma. Characterization of MP subpopulations may help resolving their contributions to normal and pathological functions.

Funded by: The Danish Heart Association

**Different phenotype of exosomes from human breast milk of allergic and healthy mothers and the effect of an anthroposophic lifestyle**

Susanne Gabrielsson^1^, Patricia Torregrosa Paredes^1^, Cindy Gutzeit^1^, Fredrik Stenius^2^, Johan Alm^2^, Gunnar Lilja^2^ and Annika Scheynius^1^

^1^Translational Immunology Unit, Department of Medicine Solna, Karolinska Institutet, Sweden; ^2^Sacchsska Children's Hospital, Karolinska Institutet, Stockholm, Sweden

Breastfeeding has beneficial effects on the developing immune system of the neonate. However, the role for breast milk in allergy development is controversial. Maternal allergies or exposures to certain lifestyle factors have been linked to certain immunological profiles in breast milk. The aim of this study was to characterize breast milk exosomes from colostrum and mature milk and to investigate if allergic sensitization as well as an anthroposophic lifestyle could influence the exosome profile in mature milk. Our results show that exosomes from mature milk have higher levels of MHC I compared to those in colostrum, while, the reverse was true for MHC class II. By using different catcher beads, we detected different exosome populations in breast milk. Exosomes from allergic mothers that were selected on anti-MHC class II beads showed a significantly higher expression of MHC class II compared to exosomes from non-allergic mothers. Furthermore, significantly lower levels of MUC1 were found on MHC-class II selected exosomes from anthroposophic mothers compared to conventional mothers. Our data are now compared to the allergic outcome at the age of 2. Our results show for the first time that allergic status and lifestyle can be detected as an altered exosome profile in breast milk and might have implications for the understanding of the influence of breast milk on allergy development.

Funded by: The Swedish Research Council, The Swedish Heart-Lung Foundation, The Cancer and Allergy Foundation, The Hesselman Foundation and The Milk Drop Foundation

**Comparison of protocols for the preservation and isolation of extracellular vesicles in human breast milk**

Marijke I. Zonneveld^1,2^, Esther N.M. Nolte 't Hoen^2^, Frank. A. Redegeld^1^, Johan Garssen^1,3^ and Marca H.M. Wauben^2^

^1^Division of Pharmacology, Department of Pharmaceutical Sciences, Faculty of Science, Utrecht University, Utrecht, The Netherlands; ^2^Department of Biochemistry & Cell Biology, Faculty of Veterinary Medicine, Utrecht University, Utrecht, The Netherlands; ^3^Danone Research Centre for Specialized Nutrition, Wageningen, The Netherlands

Email: m.i.zonneveld@uu.nl

Extracellular membrane vesicles (EMV) with immune modulatory properties have recently been identified in human breast milk. We hypothesize that EMV in mother's milk can instruct the immune system of infants. However, the exact function of EMV in breast milk is unknown, and further analysis of the presence and characteristics of these EMV is necessary. We aimed to define a protocol that ensures efficient recovery of EMV from (stored) human breast milk. Milk stored in milk banks is often frozen without prior processing, which may lead to the destruction of cells present in milk. We found that fresh human breast milk contained±0.3×10^−6^ cells/ml, with the cell count being largely dependent on the centrifugation speed used. The majority of these cells were CD14^+^ macrophages, of which a subpopulation was HLA-DR+. Freezing of unprocessed milk, followed by thawing, resulted in a complete loss of cells. Vesicles were isolated from fresh or stored milk, using differential centrifugation steps and sucrose-cushion and -gradient centrifugation. We found that the vesicle fraction, obtained from frozen unprocessed milk, contained substantially more MHC II than the fraction obtained from milk frozen after cell removal. Freezing after cell removal did, however, not affect the vesicle population, as measured by the amount of recovered CD9. We conclude that freezing of unprocessed milk may lead to contamination of the milk EMV populations with vesicles/debris from damaged cells. Early and complete cell removal is, therefore, a requisite to preserve milk samples for vesicle isolation.

Funded by: The Dutch Technology Foundation STW, which is the applied science division of NWO, and the Technology Programme of the Ministry of Economic Affairs (STW-Danone partnership Grant No. 11676)

**Importance of RNA isolation methods for analysis of exosomal RNA: evaluation of different methods**

M. Eldh, Jan Lötvall, Carina Malmhäll and Karin Ekström

Krefting Research Centre. Department of Internal Medicine, University of Gothenburg, Sweden

Email: maria.eldh@gu.se

*Background:* Exosomes differ compared to their donor cells in RNA, protein and lipid composition. Many studies describe exosomal RNA from different cellular origins using different RNA isolation methods giving rise to different exosomal RNA size distribution patterns. In some publications, it seems that exosomes are enriched in small RNA while, in others, a more wide size distribution pattern is seen. The aim of the current study was to determine whether variation in exosomal RNA quantities, size distribution and quality could be a consequence of the RNA extraction method used and to determine the optimal way to extract RNA from exosomes. *Method:* Exosomes were isolated from a mast cell line (MC/9) and seven different RNA extraction methods were evaluated to determine quality, purity, yield and size of RNA. The methods evaluated were one phenol-based method, three combined phenol and column-based methods and three column-based methods. The RNA quality was determined for cellular RNA by the Bioanalyzer using RNA integrity numbers (RIN) and the purity was evaluated spectrophotometerically at the absorbance 230, 260 and 280 nm. The RNA yield and size of exosomal and cellular total and small RNA was analyzed using the Bioanalyzer. The presence of microRNA was confirmed by real-time PCR. *Results:* All methods evaluated extracted high quality and purity RNA. However, the exosomal RNA yield and Bioanalyzer electropherogram patterns differed substantially between the isolation approaches. *Conclusions:* In the current study, we present a unique comparison of seven different methods for extraction of exosomal RNA. As the different isolation methods give such an extensive variation in exosomal RNA yield and pattern, it is of great importance to choose the method carefully depending on the research question at hand.

Funded by: VBG GROUP'S Herman Krefting Foundation for Allergy and Asthma Research, the Swedish Research Counciland and the Swedish Asthma and Allergy Foundation

### Coffee and Poster Session 3          15.00-15.30

### Symposium Session 9          15.30-17.00

#### 1. Extracellular Vesicles and the Brain

**Characterization of exosomes secreted from neuronal sources**

Jinghuan Li^1^, Yi Lee^1^, Christopher Gardiner^2^, Samir El Andaloussi^1^, Samira Lakhal-Littleton^1^, Ian L. Sargent^2^ and Matthew J.A. Wood^1^

^1^Department of Physiology, Anatomy and Genetics, University of Oxford, Oxford, United Kingdom; ^2^Nuffield Department of Obstetrics and Gynaecology, University of Oxford, Oxford, United Kingdom

Email: jinghuan.li@dpag.ox.ac.uk

Exosomes from bone marrow dendritic cells (BMDC) have been shown to be successful delivery vehicles for targeted siRNA delivery to the brain. However, it is still unknown if exosomes from this source could achieve optimal treatment in different brain diseases. One alternative parental cell type, which may potentially be more applicable is neuronal cells and/or neuronal stem cells. Here, we report the study of a number of neuronal cell lines to assess their potential as sources of exosomes for nervous system drug delivery. We used nanoparticle tracking analysis (NTA) to measure the size and concentration of exosomes isolated from these cell lines. Combined with traditional techniques, sucrose gradient purification and western blotting, we purified and characterized exosomes secreted from cell lines in vitro. Neuronal cell lines (NSC34 and N2a), fibroblasts (NIH) and kidney cells (HEK) were used as sources of exosomes. Exosomes were isolated from culture media by a series of centrifugations. The pellet was suspended in HEPEs solution, loaded onto a sucrose gradient (0.25–2.5 M) and ultracentrifuged overnight. Eleven fractions were collected, diluted in PBS and subjected to ultracentrifugation again to pellet exosomes. NSC34 and N2a, compared to other cell lines, produced a good population of exosomes as shown by their size, concentration and distribution of particles visualized by NTA system. Western blotting showed that exosomal markers, including CD9, are present in both cell lines. Furthermore, sucrose gradient showed that these exosomes float on the density between 1.09 g/ml (0.75 M) and 1.17 g/ml (1.25 M), which separated exosomes from other protein contaminations. Therefore, we have identified two neuronal cell lines that produce an abundant number of exosomes, which appear to have potential as sources of exosomes for future studies on neurological disease applications.

Funded by: MRC biotechnology

**The unconventional secretion of Stress Inducible Protein 1 by astrocytes reveals a heterogeneous population of microvesicles**

Glaucia N.M. Hajj^1^, Camila Arantes^2^, Isabel Porto-Carreiro^3^, Flávia R. Lima^3^, Marco M.A. Prado^4^, Rafael Linden^3^ and Vilma R. Martins^1^

^1^Centro Internacional de Pesquisa, Hospital A.C. Camargo, Brazil; ^2^Instituto de Química/Universidade de São Paulo, Brazil; ^3^Universidade Federal do Rio de Janeiro, Brazil; ^4^Robarts Research Institute, University of Western Ontario, Canada

Email: ghajj@cipe.accamargo.org.br

Astrocytes secrete neurotrophic factors responsible for neuronal survival and differentiation. The co-chaperone Stress Inducible Protein 1 is secreted by astrocytes and has neurotrophic properties upon binding to prion protein (PrPC) at the neuronal surface. However, STI1 lacks a signal peptide and pharmacological approaches pointed that it does not follow a classical secretion mechanism. Ultracentrifugation protocols, size exclusion chromatography, vesicle labeling and electron microscopy demonstrated that STI1 is secreted in microvesicles (MVs) that vary in size from 50 to 500 nm. These microvesicles present many exosomal markers, even though only a subpopulation had the typical exosomal morphology. The only protein present exclusively in vesicles that have exosomal morphology was PrPC. Multivesicular bodies were also found in astrocytes, containing intraluminal vesicles of different sizes. Shedding of microparticles derived from the plasma membrane was ruled out by the use of isopycnic gradiends. STI1 incorporated into MVs but not that found in a soluble form promotes neuronal signaling. These results indicate that astrocytes secrete a population of MVs of different size and morphology that labeled for classical exosomal markers. STI1 secretion in MVs is essential for its ability to trigger neuronal signaling.

Funded by: Supported by Sao Paulo State Foundation (FAPESP) and National Institute for Translational Neuroscience (INNT)

**Loss of exosomal progranulin in frontotemporal dementia**

Luisa Benussi^1^, Michela Glionna^1^, Anna Paterlini^2^, Valentina Albertini^2^, Giuliano Binetti^1^ and Roberta Ghidoni^2^

^1^NeuroBioGen Lab Memory Clinic, IRCCS Istituto Centro San Giovanni di Dio Fatebenefratelli, Brescia, Italy; ^2^Proteomics Unit IRCCS Istituto Centro San Giovanni di Dio Fatebenefratelli, Brescia Italy

Email: lbenussi@fatebenefratelli.it

Progranulin (GRN) is a pleiotropic protein that has gained the attention of the neuroscience community with recent discoveries of mutations in the gene for GRN that cause frontotemporal lobar degeneration (FTLD). It is well known that progranulin is involved in wound repair, inflammation and tumor formation, but its function in the central nervous system or the mechanism by which it leads to neurodegeneration remain open questions. All pathogenic GRN mutations identified thus far cause the disease through a uniform mechanism, i.e., loss of functional progranulin or haploinsufficiency. Herein, we investigated exosomal trafficking and release in human primary fibroblasts obtained from patients carrying GRN mutations and control subjects. Microvesicles released by human primary fibroblasts were isolated by ultracentrifugation and sucrose gradient fractionation as previously described (1,2) and analyzed using Western blot and SELDI TOF mass spectrometry. We demonstrated that (i) progranulin is released in association with exosomes mainly in its glycosylated form; (ii) mutations in GRN gene impair the release of exosomes; and (iii) pathogenic mutations deplete trophic factors exosomal transport (i.e., progranulin and cystatin c). A better understanding of the mechanisms involved in exosomal progranulin processing, release and uptake is of great therapeutic interest and may have important implications for the fight against FTLD and other neurodegenerative diseases.

Funded by: Cariplo Foundation, Grant No. 2009-2633. PI: Dr. Roberta Ghidoni

**References**

1. Théry C, et al. Curr Protoc Cell Biol. 2006 Apr; Chapter 3: Unit 3.22.

2. Ghidoni R, et al. Neurobiol Aging. 2011 Aug;32(8):1435–42.

**Human CSF amyloid-beta peptides exosomal compartimentalization in Alzheimer's disease**

Roberta Ghidoni, Valentina Albertini, Anna Paterlini, Michela Glionna, Giuliano Binetti and Luisa Benussi

Proteomics Unit IRCCS Istituto Centro San Giovanni di Dio Fatebenefratelli, Brescia, Italy; NeuroBioGen Lab Memory Clinic, IRCCS Istituto Centro San Giovanni di Dio Fatebenefratelli, Brescia, Italy

Email: rghidoni@fatebenefratelli.it

The predominant protein component of Abeta plaques in Alzheimer's disease (AD) is strongly aggregating peptides with an approximate molecular mass of 4 kDa. Among these peptides, Abeta1–40 and Abeta1–42 have been the dominant focus research, but it is well established that N- and C-terminally truncated or modified forms of Abeta peptides also exist in AD brain and cerebrospinal fluid (CSF). Herein, we investigated the Abeta CSF exosomal compartimentalization of N- and C-terminally truncated Abeta peptides in patients with AD and with subjective memory complaints (SMCs). Microvesicles released by human CSF (AD: n=10; SMCs: n=10) were isolated by ultracentrifugation and sucrose gradient fractionation as previously described (1,2). The immunoproteomic analysis for truncated Aβ peptides detection was performed using SELDI-TOF mass spectrometry on PS20 chip array and specific monoclonal antibodies (6E10 + 4G8) as previously described (3). We observed (i) that exosomes transport 14 different Abeta peptides (including 3 N-terminally truncated forms) and (ii) a differential exosomal Abeta compartimentalization in health and disease. A better understanding of the mechanisms involved in exosomal Abeta processing, release and uptake is of great therapeutic interest and may have important implications for the fight against AD.

Funded by: AFaR 2012 grant number ID 13; Monzino Foundation; Ricerca Corrente, Italian Ministry of Health.

**References**

1. Théry C, et al. Curr Protoc Cell Biol. 2006 Apr; Chapter 3: Unit 3.22.

2. Ghidoni R, et al. Neurobiol Aging. 2011 Aug;32(8):1435–42.

3. Albertini V, Proteomics Clin Appl. 2010 Mar;4(3):352–7.

**Treatment of brain inflammatory diseases by delivering exosome encapsulated chemotherapeutic agents from the nasal region to the brain**

Lifeng Zhang, Xiaoying Zhuang, Xiaoyu Xiang, William Grizzle, Dongmei Sun, Shuangqin Zhang, Robert C. Axtel, Songwen Ju, Jiangyao Mu, Lawrence Steinman, Donald Miller and Huang-Ge Zhang

University of Louisville, Louisville, KY, USA

Email: L0zhan19@louisville.edu

In this study, exosomes used to encapsulate curcumin (Exo-cur) or a Stat3 inhibitor, i.e., JSI124 (Exo-JSI124) were delivered non-invasively to microglial cells via an intranasal route. The results generated from three inflammation-mediated disease models, i.e., a LPS-induced brain inflammation model, experimental autoimmune encephalitis and a GL26 brain tumor model, showed that mice treated intranasally with Exo-cur or Exo-JSI124 are protected from LPS-induced brain inflammation, the progression of MOG peptide induced EAE, and had significantly delayed brain tumor growth in the GL26 tumor model. Intranasal administration of Exo-cur or Exo-JSI124 led to rapid delivery of exosome encapsulated drug to the brain that was selectively taken up by microglial cells and subsequently induced apoptosis of microglial cells. Our results demonstrate that this strategy may provide a non-invasive and novel therapeutic approach for treating brain inflammatory related diseases.

Funded by: Louisville Veterans Administration Medical Center (VAMC) Merit Review Grants (H.-G.Z.); the National Institutes of Health (NIH) (RO1CA137037, R01AT004294, R01CA116092 and R01CA107181); and a grant from the Susan G. Komen Breast Cancer Foundation

#### 2. NIH Reaches Out to the ISEV Community

Chair: Elizabeth Wilder, Ph.D. Director, Office of Strategic Coordination, National Institute of Health, USA

***Open discussion: Results of outreach to the ISEV community. Discussion of unmet needs**.*

***What can be done to strengthen the field of extracellular vesicles?***

### All Posters in Session 3

#### 1. Immunology and Inflammation

**1. T lymphocytes are targets for platelet- and trophoblast-derived microvesicles during pregnancy**

E. Pap, É. Pállinger, A. Falus and E.I. Buzás

Department of Genetics, Cell and Immunobiology, Semmelweis University, Budapest, Hungary

Email: nyierna@dgci.sote.hu

Successful pregnancy requires a series of interactions between the maternal immune system and the implanted fetus, such that the semiallograft will not be rejected. These interactions occur at the materno-placental interface and/or at a systemic level. In the present study, we identified for the first time the in vivo plasma pattern of the microvesicles (MVs) of third-trimester healthy pregnant women: their cellular origin and their target cells using flow cytometry and confocal laser microscopy. We also examined the in vitro effects of MVs on STAT3 phosphorylation of peripheral lymphocytes. We found that both placental trophoblast-derived and maternal thrombocyte-derived MVs bind to circulating peripheral T lymphocytes but not to B lymphocytes or NK cells. We showed that the P-selectin (CD62P)–PSGL-1 (CD162) interaction is one mechanism binding platelet-derived MVs to T cells. We were also able to demonstrate that pregnant MV–lymphocyte interactions inhibit IL-2-induced STAT3 phosphorylation in T cells. Our findings indicate that both thrombocyte- and trophoblast-derived MVs play role in the communication between the placenta and the maternal immune system and that MVs contribute to the establishment of stable immune tolerance to the semi-allograft fetus.

Funded by: ETT 140/2006 Grants of Hungarian Ministry of Health

**2. Characterization of human thymic exosomes**

Gabriel Skogberg^1^, Judith Gudmundsdottir^1,2^, Esbjörn Telemo^1^ and Olov Ekwall^1,2^

^1^Department of Rheumatology and Inflammation Research, The Sahlgrenska Academy, University of Gothenburg, Sweden; ^2^Department of pediatrics, The Sahlgrenska Academy, University of Gothenburg, Sweden

Email: xskoga@gu.se

This study is focused on the characterization of human thymic exosomes regarding surface molecules, protein content, size, density and morphology. Exosome-like particles have previously been described in mouse thymus where they induce regulatory T cells (1). Thymus was collected from infants undergoing cardiac surgery; 1 − 2 g of thymic tissue was fragmented followed by exosome purification by a protocol according to either directly or after incubation at 4°C or 37°C for 24 hours. We identified and characterized the microparticles, using Nanosight???, electron microscopy as well as separation on a sucrose gradient, where they showed a size within exosome range and float in the density range of 1.13 to 1.20 g/ml. This is consistent with previously described exosomal characteristics. Flow cytometry analysis of the exosomes coupled to latex beads revealed surface markers typical for exosomes such as TSG101 and CD81 as well as other markers typical for interactions between immune cells such as HLA-DR, ICAM-1 and TGF-β. Protein content of the exosomes was also investigated with proteomics approach using nano-LC/MS/MS method. The presence of TGF-β HLA-DR and ICAM-1 on the exosomes in the neonatal thymus may suggest that they participate in the induction of central tolerance as a route of antigen transfer in negative selection and induction of regulatory T cells.

Funded by: The Swedish Research Council, Region Västra Götaland, AFA Försäkring, IngaBritt and Arne Lundbergs Research Foundation and The Göteborg Medical Society

**Reference**

1. Zhang L, et al. Cell Mol Immunol. 2008 Oct;5(5):325–32.

2. Théry C, et al. Curr Protoc Cell Biol. 2006 Apr;Chapter 3:Unit 3.22.

**3. BeWo-derived microvesicles modulate T cell differentiation by the downregulation of IL-6Ralpha expression on CD4 + T lymphocytes**

É. Pállinger^1^, A.A. Kiss^2^, E. Pap^1^, S. Tóth^1^ and A. Falus^1^

^1^Department of Genetics, Cell- and Immunobiology, Semmelweis University, Budapest, Hungary; ^2^Department of Gynecology and Obstetrics of Military Hospital − Hungarian State Health Center, Budapest, Hungary

Email: paleva@dgci.sote.hu

Maternal immunotolerance requires the action of regulatory T cells at the feto-maternal interface, whose differentiation is influenced by the local cytokine milieu. Microvesicles (MVs) have been shown to mediate communication between the maternal immune system and the semiallograft fetus. We assume that trophoblast-derived MVs regulate both the local cytokine production and the cytokine sensitivity of the lymphocytes. BeWo choriocarcinoma cell–lymphocyte co-culture model system was used for the multicolor flow cytometric investigations of cytokine production and IL6Ra expression of pregnants’ lymphocytes. In the present work, we showed that (1) trophoblast-derived MVs induce IL-6 secretion of lymphocytes and (2) we could measure lower IL-6Ra expression of CD4 + T lymphocytes and higher IL-10 secretion, meaning that the inhibitory effect of IL-6 on regulatory T-cell differentiation is diminished by trophoblast-derived MVs. Therefore, trophoblast-derived MVs contribute to the establishment and maintenance of local maternal immunotolerance, which is a prerequisite for healthy pregnancy.

Funded by: ETT050/2009 (grants of Hungarian Ministry of Health)

**4. Could microRNAs derived from lymphocytes trigger beta-cell dysfunction in type 1 diabetes?**

C. Guay^1^, E. Roggli^1^, C. Briet^2^, V. Menoud^1^, S. Gattesco^1^, C. Boitard^2^ and R. Regazzi^1^

^1^DBCM, University of Lausanne, Switzerland; ^2^Hôpital Cochin/St Vincent de Paul, INSERM U986, Paris, France

Email: claudiane.guay@unil.ch

MicroRNAs are important regulators of pancreatic beta-cell functions. During development of type 1 diabetes, lymphocytes infiltrate the pancreatic islets, resulting in beta-cell dysfunction and death. Since during this process beta-cells and T cells are in close proximity, we hypothesized that microvesicle-mediated transfer of specific microRNAs from T cells to insulin-secreting cells could contribute to dysfunction of beta-cells in the early phases of type 1 diabetes. To test this novel concept, microvesicles isolated by ultracentrifugation from the culture media of the Jurkat T cell line or from primary T effector cells of diabetes prone NOD mice were applied to the beta-cell line MIN6 or to dispersed primary rat islet cells. We observed that four microRNAs, miR-142-3p, miR-142-5p, miR-150 and miR-155, are highly upregulated in pancreatic islets during prediabetic insulitis in NOD mice. These microRNAs are over a thousand times more abundant in T cells compared to beta-cells and are contained in microvesicles originating from T cells. When microvesicles of Jurkat T cells were applied to beta-cells, those four microRNAs were increased in recipient beta-cells. This transfer correlated with a rise in beta-cell apoptosis and impairment in insulin secretion. Taken together, our data suggest that microRNAs associated with microvesicles released by T cells can be taken up in active form by beta-cells resulting in alterations of beta-cell functions.

Funded by: FNS, ALFEDIAM, FRSQ and CDA

**5. CD8 + T cell exosomes from tolerized mice deliver inhibitory miRNA-150 to antigen specifically suppress effector T Cells**

Krzysztof Bryniarski^1^ and Philip W. Askenase^2^

^1^Jagiellonian University School of Medicine, Kracow, Poland; ^2^Yale University School of Medicine, New Haven, CT, USA

Email: philip.askenase@yale.edu

Suppressor CD8 + T cells from mice tolerized with a high i.v. doses of antigen produce suppressive supernatant nanovesicle exosomes identified by passing 0.1 µ filters, pelleting at 100,000 g, 132 nm by nanoparticle tracking, typical electron microscopic appearance, sucrose buoyancy and expression of tetraspanins by flow cytometry, immunoblotting and antibody affinity chromatography. The exosomes were antigen specific and could bind antigen enabling antigen affinity column chromatography. This resulted in isolation of an antigen binding 10% minor subpopulation mediating all the suppression vs. non-binding non-suppressive 90% remaining. Then, cDNA cloning, Solexa deep sequencing the two fractions and comparison of miRNA reads revealed 28 of 5 million greater in the adherent suppressive fraction. miR-150 was among the leaders. Antagomirs to miR-150 specifically inhibited exosome suppression. Experiments with miRNA-150-/- mice were definitive. They could not be tolerized and their tolerized exosomes were not suppressive but could be reconstituted for mediating suppression by transfection with miR-150 compared to controls. We concluded that miRNA-150 accounted for the suppressive activity of the exosomes. Overall, our results suggest that such exosomes can be surface coated with antibody of choice, per our other abstract, and loaded with selected miRNA. This enables antigen specific delivery to particular cells and targeting selected dsRNA. Thus, exosomes can be tailored to bind specific cells and deliver selected siRNA, to naturally and systemically treat diseases.

Funded by: NIHAI-076366 and AI-07074

**6. Natural killer cells-derived exosomes: a cell-free support in immune surveillance**

Luana Lugini^1^, Serena Cecchetti^2^, Veronica Huber^3^, Francesca Luciani^4^, Gianfranco Macchia^2^, Francesca Spadaro^2^, Luisa Paris^2^, Laura Abalsamo^2^, Marisa Colone^5^, Agnese Molinari^5^, Franca Podo^2^, Licia Rivoltini^5^, Carlo Ramoni^2^ and Stefano Fais^1^

^1^Department of Therapeutic Research and Medicines Evaluation, Istituto Superiore di Sanità, Rome, Italy; ^2^Department of Cell Biology and Neurosciences, Istituto Superiore di Sanità, Rome, Italy; ^3^Center for Immunobiologicals Research and Evaluation, Istituto Superiore di Sanità, Rome, Italy; ^4^Department of Technologies and Health, Istituto Superiore di Sanità, Rome, Italy; ^5^Unit of Immunotherapy of Human Tumors, Fondazione IRCCS Istituto Nazionale dei Tumori, Milan, Italy

Email: luana.lugini@iss.it

Exosomes are secretory nanovesicles of normal and tumor cells that can be detected in cell culture supernatant and human body fluids. Exosomes derived from hematopoietic cells have been largely investigated, but very little is known on natural killer (NK) cells-derived exosomes, despite of the potential importance of these nanovesicles in NK cell function. In this study, we identified and purified human NK-derived exosomes either from NK cell culture supernatants or plasma. Morphological and phenotypical characterization demonstrated that human resting and activated NK cells release exosomes expressing not only both housekeeping exosome markers (Rab5B and CD63) and typical NK cell markers (CD56, NKG2D, NKp30, NKp46 and NKp44) but also killer molecules (i.e., FasL and perforin). Moreover, NK cell exosomes not only exerted cytotoxic activity against several human tumor cell lines of different histologies but also activated lymphocytes. Plasmatic exosomes from healthy individuals expressed NK-cell markers, including CD56+, were perforin + and displayed cytotoxic activity against different tumor target cells and activated immune cells, as well. This study provides the evidence that NK-derived exosomes may participate in the homeostatic control of our body, representing an almost perfect example of biomimetic nanovesicles with cytotoxic activity, possibly useful in future therapeutic approaches against various diseases, including tumors.

Funded by: Istituto Superiore di Sanità, Rome, Italy

**7. The potential of LCL-derived exosomes to influence B cell biology**

Cindy Gutzeit^1^, Noémi Nagy^2^, Maurizio Gentile^3^, Andrea Cerutti^3^,Eva Klein^2^, Susanne Gabrielsson^1^ and Annika Scheynius^1^

^1^Translational Immunology Unit, Department of Medicine Solna, Karolinska Institutet, Stockholm, Sweden; ^2^Department of Microbiology, Tumor and Cell Biology, Karolinska Institutet, Stockholm, Sweden; ^3^Institut Municipal d‘Investigatió Mèdica (IMIM), Barcelona, Spain

Email: cindy.gutzeit@ki.se

Epstein Barr virus (EBV) transformed B cells (LCLs) release exosomes, which harbor latent membrane protein 1 (LMP1). LMP1 signaling can replace CD40 signaling in B cells in vivo and has unique features of inducing class-switch recombination (CSR). We have previously demonstrated that certain LCL-derived exosomes selectively target human B cells (1). We here investigate the capacity of LMP1-containing exosomes to induce phenotypical and functional changes in naïve B cells. Exosomes derived from several B cell lines were analyzed for their capacity to influence B cell biology by investigating their binding pattern to naïve B cells, their potency to induce morphological changes, to upregulate B cell activation markers and to induce proliferation. Our preliminary data show that depending on the source of exosomes their effect on naïve B cells differed. LMP1-containing exosomes induced morphology changes (clumping) and proliferation of naïve B cells. Currently, we are investigating whether exosomes induce the expression of activation-induced deaminase (AID) and CSR in naïve B cells. We suggest that exosomes can influence the biology of naïve B cells and that the nature of exosomes has an impact on it.

Funded by: The Center for Allergy Research and Hesselman foundation through Junior faculty at Karolinska Institutet, the Swedish Research Council and Swedish Cancer Society (Cancerfonden)

**Reference**

1. Vallhov H, et al. J Immunol. 2011 Jan 1;186(1):73–82.

**8. Targeting of antigen-loaded exosomes to B cells**

Stefanie Hiltbrunner, Tanja Näslund and Susanne Gabrielsson

Translational Immunology Unit, Department of Medicine, Karolinska Institutet, Sweden

Email: stefanie.hiltbrunner@ki.se

It has been shown that B cell help is needed for T cell stimulation after ovalbumin (OVA)-loaded exosomes (Exo-OVA) injection. Furthermore, targeting of antigens to B cells efficiently activates T cells. To investigate whether exosomes engineered to target B cells would further amplify the CD8 + T cell response to exosomes, and if these exosomes are be potent in tumor killing, we engineered an exosome/streptavidin/antibody complex. We produced OVA-biotin expressing dendritic cell-derived exosomes linked with streptavidin, allowing any biotinylated antibody to be added to the system, hence targeting any cell type. Ongoing studies include comparing the antibody-OVA system effect on directly and indirectly loaded exosomes, to determine the role of the B cell in T cell activation. To investigate the nature of the exosome/streptavidin/antibody complex, and if the whole OVA protein is detectable on exosomes, which could bind to the B cell receptor, we analyzed exosomes loaded with biotinylated OVA by FACS and ELISA. Our data suggest that the biotinylated OVA is located in the membrane as a whole protein. Additional preliminary results show that it cannot be removed from the membrane by trypsin digestion. This is now verified by usage of other proteases. To further investigate how a protein is loaded on exosomes, we will compare the loading of different antigens. Our results will give insight into how antigens are incorporated into exosomes and might be valuable for designing exosome-based vaccines.

Funded by: Swedish Research Council, Swedish Heart-Lung Foundation, Swedish Cancer Society

**9. Exosomes as drug carriers in macrophage-mediated drug delivery**

Elena V. Batrakova^1^, Matthew J. Haney^1^, Yuling Zhao^1^, Natalia L. Klyachko^1,2^, R. Lee Mosley^1^, Alexander V. Kabanov^1^ and Howard E. Gendelman^1^

^1^University of Nebraska Medical Center, Omaha, NE, USA; ^2^ M.V. Lomonosov Moscow State University, Moscow, Russia

Email: ebatrako@unmc.edu

Macrophage carried nanoformulated catalase (nanozyme) attenuates neuroinflammation and protects the nigrostriatal neurons in murine models of Parkinson's disease (1,2). This is facilitated by effective enzyme transfer from blood borne macrophages to adjacent endothelial and ultimately to neural target cells leading to the decomposition of reactive oxygen species. The nanozyme transfer occurred in exosomes released from preloaded with drug nanoparticles macrophages. Notably, the delivery of the redox enzyme, catalase, incorporated into a polyion complex micelle (nanozyme) (3) by monocytes protected the nigrostriatal in PD mouse model. In macrophages, nanozymes are internalized by clathrin-mediated endocytosis and localize principally in recycling endosomes. The enzyme is subsequently released in exosomes through bridging conduits. Nanozyme was transferred from macrophages to target brain endothelial and neural cells by endocytosis-independent mechanisms diffusing broadly throughout the recipient cell. In contrast, macrophage-free nanozymes are localized in lysosomes following endocytic entry. Facilitated transfer of nanozyme from cell to cell can improve neuroprotection against oxidative stress commonly seen during neurodegenerative disease processes. These data support the importance of macrophage-based nanozyme carriage for neurodegenerative therapies.

Funded by: National Institutes of Health grants 1R01 NS057748 (to EVB), 2R01 NS034239, 2R37 NS36126, P01 NS31492, P20RR15635, P01 MH64570, P01 NS43985 (to HEG), RR021937 (to AVK), R01 NS070190 (to RLM) and Russian Ministry of Science and Education grants 02.740.11.5231 and 11.G34.31.0004 (to AVK)

**References**

1. Brynskikh AM, et al. Nanomedicine (Lond). 2010 Apr;5(3):379–96.

2. Batrakova EV, et al. Expert Opin Drug Deliv. 2011 Apr;8(4):415–33. Epub 2011 Feb 24. Review.

3. Zhao Y, et al. Nanomedicine (Lond). 2011 Jan;6(1):25–42.

4. Haney MJ, et al. Nanomedicine (Lond). 2011 Sep;6(7):1215–30.

**10. Neutrophilic granulocyte-derived microvesicles in vitro and in vivo**

Cs. Timar^1^, A. Lorincz^1^, Zs. Ivanyi^2^ and E. Ligeti^1^

^1^Department of Physiology, Semmelweis University, Budapest, Hungary; ^2^Department of Anaesthesiology and Intensive Therapy, Semmelweis University, Budapest, Hungary

Email: tcsaba@eok.sote.hu

*Background*: As we showed before, neutrophilic granulocytes (PMN) activated by opsonized bacteria release microvesicles with antibacterial effect (bMV), while spontaneously formed microvesicles (sMV) had no effect on bacterial growth. To investigate the potential mechanism, fate of MV and bacteria was followed by fluorescence microscopy and with bacterium killing assay in the absence and presence of various inhibitors. To determine clinical relevance, we investigated abundance and effect of PMN derived MV from serum of healthy and of bacteremic donors. *Materials and methods*: PMN were prepared from blood of healthy volunteers; serum was collected from bacteremic and from healthy donors. MVs from PMN and from serum were separated by two-step centrifugation and filtration. Amount of MV was determined by flow cytometry, and antibacterial effect was followed by bacteria killing assay and was characterized fluorescence microscopy. *Results and conclusions*: Fluorescence microscopic assays revealed aggregation of bacteria to bMVs but not to sMV. Aggregation depended on intact actin and vesicular structure and correlated well with bacterial growth. PMN-derived MVs could be detected in human serum using CD11b and CD177 co-labeling. Number of labeled MV increased 4-fold in bacteremic donors as compared to healthy volunteers. MV from bacteremic patients formed similar aggregates with bacteria as bMV did, while no aggregation was observable with MV from healthy serum. Funded by: Hungarian National Research Fund (OTKA K75084) and TÁMOP (grants 4.2.1/B-09/1/KMR-2010-0001 and 4.2.2/B10/1-2010-0013)

**11. Effects of metal-rich particulate matter exposure on microRNAs carried in plasma microvesicles**

V. Bollati, L. Angelici, G. Rizzo, F. Nordio, L. Pergoli, M. Bonzini, L. Tarantini, L. Cantone, A.C. Pesatori, P. Apostoli, A. Tripodi, A. Baccarelli andP.A. Bertazzi

Center of Molecular and Genetic Epidemiology, Department of Environmental and Occupational Health, Università di Milano, Fondazione Cà Granda, IRCCS Ospedale Maggiore Policlinico, Milan, Italy; Department of Clinical and Experimental Medicine, University of Insubria, Varese, Italy; Occupational Medicine and Industrial Hygiene, University of Brescia, Department of Experimental and Applied Medicine, Brescia, Italy; Angelo Bianchi Bonomi Haemophilia and Thrombosis Centre, Department of Internal Medicine, Università degli Studi di Milano and IRCCS Maggiore Hospital Foundation, Milan, Italy; Exposure, Epidemiology and Risk Program, Department of Environmental Health, Harvard School of Public Health, Boston, MA, USA

Email: valentina.bollati@unimi.it

*Background and Aims:* Epidemiological studies have reported an increased risk for cardiovascular disease (CVD) in relation to exposure to metal-rich particulate matter. Cell-derived membrane microvesicles (MVs) are released in plasma and transfer microRNAs. MVs might mediate the effects of air pollution, since potentially they could be produced by the respiratory system, reach the systemic circulation and lead to the development of CVD. The aim of the present study was to identify effects of exposure to metal-rich particulate matter on MV-associated microRNAs expression in workers of an electric furnace steel plant with well-characterized exposure. *Methods:* Plasma MVs were isolated by ultracentrifuge from 55 steel workers on the first day of a workweek (baseline) and after 3 days of work (postexposure). We measured the expression of the 88 most abundantly expressed and best characterized microRNA sequences by SYBR green chemistry. The relative expression of miRNAs was measured by real-time PCR using SNORD48, SNORD47, SNORD44 and RNU6-2 as endogenous controls. Relative quantification of miRNA expression was calculated using the 2-ΔCt method. Paired *t*-tests were used to compare baseline and postexposure samples. *Results:* Expression profiling of 88 human miRNAs extracted from baseline and paired postexposure blood samples identified that miR-128 and miR-302c expression was increased in postexposure samples. We also evaluated the associations between microvesicle-associated miRNA expression and inflammation/coagulation markers, and we found 10 microRNAs that were significantly associated. Among these 10 microRNAs, 7 miRNAs were also associated to the exposure to one or more PM metal components. *Conclusion:* These results propose a new role for microvesicles in mediating the effects of air pollution exposure on CVD risk. Our findings could lead to the identification of potentially reversible alterations that might be also considered as potential target for new diagnostic and therapeutic interventions.

Funded by: The National Institute Environmental Health Science grant ES00002 and by additional funding from INAIL Foundation and Lombardy Region Research Contracts DGR VIII/10462

**12. Exosomes as extrapulmonary signaling conveyors for nanoparticle-induced systemic Th1-type immune activation**

Motao Zhu, Xin Tian, Yanhua Tian, Yuliang Zhao and Guangjun Nie

CAS Key Lab for Biomedical Effects of Nanomaterials and Nanosafety, National Center for Nanoscience and Technology, Beijing, China

Email: zhumt@nanoctr.cn

With wide applications of nanotechnology, issues related to occupational nanosafety and public health have provoked significant debate. Evaluation of systemic nanosafety urgently demands comprehensive understanding of the systemic signaling between nanomaterials and biological systems. In the current study, we show that exosomes may act as signaling conveyors for nanoparticle-induced systemic immune responses. Herein, we found exposure of manufactured nanoparticles (magnetic iron oxide nanoparticles, MIONs) could dose-dependently generate a significant number of exosomes in BALB/c mice alveolar region. These exosomes were fast eliminated from alveoli to systemic circulation and largely transferred their signals to immune system. These exosomes act as a source of nanoparticle-induced antigen and target antigen presenting cells (APCs) in reticuloendothelial system to transfer their components. Through exosome-initiated signal transduction, immature dendritic cells (iDCs) underwent maturation and differentiation to DC1 subtype, while macrophages went through classical activation and differentiation to M1 subtype. Activated APCs (especially DC1 and M1 subtypes) consequently primed T cell differentiating into Th1 subtype and resulted in an orchestrated T helper 1 (Th1)-type immune response. Furthermore, exosomes-induced Th1 polarization was more efficient toward sensitized T cells and in ovalbumin (OVA)-sensitized mice than the unsensitized counterparts, which posed potential hazards to the deterioration of allergic diseases in sensitized mice. Our studies suggest that exosomes may act as conveyors for extrapulmonary signal transduction in nanoparticle-induced immune systemic responses, which are the key in vivo processes of manufactured nanoparticles executing either biomedical functions or toxic responses.

Funded by: National Basic Research Program of China (973 program: 2011CB933401; 2010CB933600); National Natural Science Foundation of China (31100721; 10979011; 30900278).

**13. Red blood cell-derived ectosomes amplify LPS-induced pulmonary leukocyte sequestration and systemic inflammation by activating the classical pathway of complement in mice**

Daniel Zecher, Arun Cumpelik and Jürg Schifferli

Departments of Research and Medicine, Basel University Hospital, Switzerland

Email: daniel.zecher@unibas.ch

Transfusion of blood after an extended storage time has been associated with increased morbidity and mortality in critically ill patients and with the development of acute lung injury in animal models. During storage, red blood cells lose both hemoglobin and surface area by the release of microvesicles (ectosomes). These ectosomes (Ecto) accumulate over time in the storage bags and are given in large quantities to patients at the time of transfusion. We hypothesized that Ecto might be responsible for some of the deleterious effects of aged blood transfusions. In a transfusion mouse model, we found that Ecto given intravenously induce pulmonary leukocyte sequestration and peripheral blood leukopenia in LPS-primed mice without causing overt lung injury. LPS-induced proinflammatory cytokines were significantly increased following injection of Ecto. These in vivo effects were mediated by surface expression of phosphatidylserine on Ecto and were dependent on the presence of the complement receptor C5aR. As Ecto bound both C1q and C3b in vitro, these results suggest that Ecto activate the classical pathway of complement. This complement-mediated amplification of systemic inflammation might cause some of the deleterious effects of aged blood transfusions in the critically ill patients. Recognition of Ecto as potential mediators of transfusion-related morbidity has important implications for blood storage, preservation and allocation strategies.

Funded by: Swiss National Foundation (grant 32000-116839), Research Fund Basel University, Fondation Machaon, Geneva, Switzerland

**14. Interplay between the coagulation and the complement systems: microparticle-associated tissue factor activity decreases by inhibition of the complement protein 5**

R. Øvstebø^1^, M. Hellum^1^, H.C.D. Aass^1^, A. Pharo^3^, A.M. Trøseid^1^, E. Amundsen^1^, P. Kierulf^1^, P. Brandtzaeg^2^, T.E. Mollnes^3^ and C.E. Henriksson^1^

^1^Blood Cell Research Unit, Department of Medical Biochemistry; ^2^Department of Pediatrics; ^3^Institute of Immunology, Oslo University Hospital, Norway

*Background*: *Neisseria meningitidis* (Nm) can cause fulminant meningococcal sepsis with massive activation of the coagulation and complement cascades. Bacterial cell wall molecules induce tissue factor (TF) expression on monocytes and monocyte-derived microparticles (MPs). Using human Nm-exposed whole blood, we investigated the interplay between the complement and the coagulation systems with special focus on TF. The complement cascade was challenged by eculizumab, a potent inhibitor of C5, and cell-associated-TF-mRNA and TF-protein, MP-associated TF-activity and the terminal C5b-9 complex (TCC) were measured. *Methods*: Whole blood (lepirudin), from healthy donors (*n*=6), exposed to 108 Nm/mL for 4 hours in the absence or presence of eculizumab (Soliris^®^,100 µg/mL), was subjected to analysis of TF-mRNAs (RT-PCR) and TF protein (flow cytometry). TCC was measured in plasma. To measure MP-associated TF activity, MPs from 0,5 ml plasma were pelleted and washed by sequential centrifugations (2000g, 15 minutes and three times 17,000g, 30 minutes, RT) before subjected to a clot formation assay and the calibrated automated thrombogram (CAT) assay. *Results*: In whole blood exposed to Nm, eculizumab reduced TF mRNA mean relative quantities from 18.6 to 3.3 (*p =*0.012) and monocyte-associated TF mean fluorescence intensity from 5878 to 3552 (*p*=0.0001). Furthermore, MP-associated TF-dependent mean clotting times were prolonged from 98 to 132 s (*p*=0.013), mean lagtimes (CAT) from 4.9 to 8.5 minutes (*p*=0.06) and mean ttpeaks (CAT) from 12.0 to 16.8 minutes (*p*=0.03). Eculizumab reduced mean TCC from 36 to 2 AU/ml (*p*=0.005). *Conclusions*: In Nm exposed whole blood, the coagulation and the complement systems are interplayers. Inhibition of C5 decreased cell-associated TF at the gene and protein level as well as MP-associated tissue factor activity.

**15. Role of exosomes in bronchial asthma**

A. Kulshreshtha, T. Ahmad, A. Agrawal and B. Ghosh

Molecular Immunogenetics Laboratory and Centre of Excellence for Translational Research in Asthma & Lung disease, CSIR-Institute of Genomics and Integrative Biology, Delhi, India

Email: ankur@igib.in, bittu841@gmail.com

Asthma classically has been a disease of respiratory airways, dominated primarily by Th2 cytokines. However, recent scientific advances present compelling evidences that crosstalk among various cell types plays a crucial role in asthma etiology. Considering that exosomes have emerged as the most important pleotropic player for mediating such intercellular communication, it is fair to hypothesize that they might be playing an important role in the pathology of asthma. We isolated and characterized exosomes from BAL fluids of normal and allergen sensitized and challenged mice. We also identified the cells involved in exosome-mediated communication. Our findings indicate that there are elevated levels of exosomes in asthmatic conditions. It also suggested that exosomes derived from epithelial cells can modulate the behavior of monocytes.

Funded by: Council of Scientific and Industrial Research

**16. Blood platelets interact with syncytiotrophoblast vesicles**

C. Gardiner, N.A. Redgrave, R.A. Dragovic, D.S. Tannetta, C.W.G. Redman and I.L. Sargent

Nuffield Department of Obstetrics and Gynaecology, University of Oxford, United Kingdom

Email: chris.gardiner@obs-gyn.ox.ac.uk

Preeclampsia is a common disorder of pregnancy characterised by hypertension, proteinuria, endothelial dysfunction and systemic inflammation. The maternal symptoms are thought to be caused by the increased shedding of syncytiotrophoblast vesicles (STBM) into the maternal circulation observed in preeclampsia. However, the number of STBM observed in the peripheral blood is much lower than predicted by the rate of shedding. We hypothesized that this could be due to STBM binding to platelets. To test this hypothesis, an excess of fluorescently labeled (CELL Mask Orange) STBM (1×1011/mL) were incubated in platelet rich plasma (PRP) at 37°C for 30 minutes with constant stirring. After removal of an aliquot for flow cytometry, the platelets were then removed by centrifugation, and the number of fluorescent STBM in the supernatant was determined by fluorescence nanoparticle tracking analysis (FNTA). This was repeated with the addition of 10 µM thrombin receptor activating peptide (TRAP) to weakly stimulate the platelets. As STBM are known to have tissue factor (TF) activity, STBM were also incubated with recalcified PRP with an inhibitor of fibrin polymerization. Co-localization of platelets and STBM was assessed by flow cytometry with CD61, CD62P, Cellmask and a syncytiotrophoblast specific antibody (NDOG2).FNTA showed no reduction in supernatant STBM following incubation in unstimulated PRP and < 5% of platelets demonstrated STBM binding. In contrast, incubation of STBM with TRAP-stimulated PRP decreased the STBM count by 10–34% and 60% of platelets showed STBM binding. Recalcification of the PRP resulted in a 60% reduction in free STBM. However, STBM were undetectable on the platelets surface by flow cytometry using NDOG2. We have demonstrated that weakly activated but not unstimulated platelets interact with STBM and that the TF activity of STBM can generate sufficient thrombin to activate platelets, thus facilitating their binding to STBM. The data suggest that platelets may have internalized the STBM. We propose that STBM-dependent activation of the hemostatic system and the subsequent binding of STBM may account for the apparent scarcity of circulating STBM and may contribute to the systematic inflammation and endothelial dysfunction characteristic of preeclampsia.

Funded by: This work was supported by a Wellcome Trust Technology Development Grant (ref GR087730), Wellcome Trust Programme Grant (ref GR079862MA) and by the Oxford Partnership Comprehensive Biomedical Research Centre with funding from the Department of Health's NIHR Biomedical Research Centres funding scheme

**17. Modulation of experimental models of autoimmunity by extracellular vesicles**

Borbála Aradi^1^, Krisztina Pálóczi^1^, Petra Misják^1^, Bence György^1^, Tamás G. Szabó^1^, Ágnes Kittel^2^, Szilvia Bősze^3^, Kata Horváthi^3^, Krisztina Holló^4^, Katalin Szabó-Taylor^1^, Mária Pásztói^1^, András Falus^1^ and Edit I. Buzas^1^

^1^Semmelweis University; ^2^Hungarian Academy of Sciences; ^3^Eötvös Lóránd University; ^4^University of Debrecen, Budapest, Hungary

Email: aradi.borbala@gmail.com

*Background*: We have shown recently that thymus-derived extracellular vesicles carry numerous proteins implicated in immunity and immunological tolerance induction. *Goals*: To investigate whether thymus-derived extracellular vesicles may modulate the onset and/or severity of different experimental models of autoimmune diseases. *Methods*: Extracellular vesicles (and vesicle populations) from syngeneic mouse thymuses were isolated by differential centrifugation, size filtration and sucrose flotation. We have investigated the effect of thymus-derived extracellular vesicles on proteoglycan-induced arthritis (PGIA), glucose-6-phosphate isomerase-induced arthritis (GPIA) and experimental allergic encephalomyelitis (EAE). *Results*: A single intravenous injection did not affect the onset and severity of symptoms of mice suffering from either GPIA or EAE. In contrast, in PGIA, a chronic experimental autoimmune model of human rheumatoid arthritis, injection of thymus-derived extracellular vesicles reduced the total acute arthritic clinical scores as compared to arthritic mice without injection of vesicles. *Conclusion*: These data suggest that extracellular vesicles may exert disease modifying effect in certain models of autoimmunity.

Funded by: OTKA K 73247, NK 84043 Baross Gábor (REG-KM-09-1-2009-0010)

**18. A novel flow cytometric approach reveals abundant CD8 + T-cell derived microvesicles in rheumatoid arthritis synovial fluid samples**

B. Gyorgy^1^, M. Wright^2^, B. Carr^2^, G. Nagy^1^, L. Turiak^3^, K. Vekey^3^, A. Kittel^4^, A. Falus^1^ and E.I. Buzas^1^

^1^Department of Genetics, Cell- and Immunobiology, Semmelweis University, Budapest, Hungary; ^2^NanoSight, Ltd., Amesbury, United Kingdom; ^3^Chemical Research Center of the Hungarian Academy of Sciences, Budapest, Hungary; ^4^Institute of Experimental Medicine, Hungarian Academy of Sciences, Budapest, Hungary

Email: gyorgyben@gmail.com

The assessment of microvesicles (MVs) may give insight into the pathomechanism of various disorders, and these vesicles may serve as novel biomarkers. However, the characterization of MVs in body fluids has not been fully standardized yet, and there are numerous pitfalls that may hinder the correct assessment of MVs. In this study, we tested blood plasma and synovial fluid (SF) samples of patients with osteoarthritis (OA), rheumatoid arthritis (RA) and juvenile idiopathic arthritis (JIA). We used electron microscopy (EM) and nanoparticle tracking analysis (NTA) to determine the particle size distributions in SF samples. We also applied mass spectrometry to determine the MV protein composition in SFs. To immune phenotype SF MVs, we applied flow cytometry using the novel “differential detergent lysis” method. EM and MS revealed that most of the particles in SF samples were related to protein aggregates rather than cell-derived vesicles. Using our novel flow cytometric approach, by which we exclude non-vesicular events, we demonstrate for the first time that joint diseases are characterized by unique MV signatures. Of particular importance, CD3 + and CD8 +T-cell-derived SF MVs are highly elevated in SFs of patients with RA compared to OA patients (*p =*0.027 and *p*=0.009, respectively after Bonferroni corrections). The improved assessment of MVs in RA suggests local CD8 + T-cell activation in the joints and may shed light on hidden immunopathological processes of this disease.

Funded by: Semmelweis Foundation, grants OTKA K 73247, NK 84043 and 77537, Kerpel-Fronius Ödön Fellowship and Baross Gábor (REG-KM-09-1-2009-0010).

**19. Characterization of extracellular vesicles released by synovial fibroblasts**

Mária Pásztói^1,2^, Barbara Sódar^1^, Krisztina Pálóczi^1^, Kálmán Tóth^3^, Ágnes Kittel^4^, Katalin Szabó-Taylor^1^, Tamás G. Szabó^1^, Lilla Turiák^5^, Károly Vékey^5^, András Falus^1,2^ and Edit I. Buzás^1^

^1^Semmelweis University, Budapest, Hungary; ^2^Hungarian Academy of Sciences, Semmelweis University, Budapest, Hungary; ^3^Szeged University, Szeged, Hungary; ^4^Hungarian Academy of Sciences, Budapest, Hungary; ^5^Chemical Research Center of the Hungarian Academy of Sciences, Budapest, Hungary

Email: maria.pasztoi@gmail.com

*Background*: Synovial fibroblasts (SFs) are currently considered as key players of cartilage degradation in joint diseases. *Goal*: To characterize SF-derived extracellular vesicles (EVs) as potential mediators of fibroblast activity. *Methods*: SF cell strains of patients with rheumatoid arthritis and osteoarthritis (*n*=4–4) were used as sources of EVs isolated by differential centrifugation. Proteomic composition of the EVs was analyzed by nano-HPLC/MS and the functions of the identified proteins were studied by Ingenuity pathway analysis. Furthermore, activities of enzymes possibly implicated in cartilage degradation were studied by using chromogenic substrates. *Results*: We identified 35 proteins in human SF-derived EVs and 52 proteins in fibroblasts. We found significant activity of b-D-glucuronidase, N-acetyl-glucosaminidase and thermostable hexosaminidase D activities associated with EVs secreted by SFs. *Conclusion*: Our data suggest a potential role of SFs in cartilage erosion characteristic for joint diseases.

Funded by: OTKA K, 3247 OTKA, 84043

**20. Autologous conditioned cell-free serum (ACS) contains exosomes showing anti-inflammatory activity**

M.-P. Weisshaar^1^, Sh. Amin^1^, S. Irsen^2^, J. Feydt^2^, C. Tröger^2^, G. Hoffmann^3^, J. Reineke^4^, P. Wehling^4^

^1^University of applied Scheince Bonn- Rhein-Sieg, Department of Natural Sciences, Rheinbach, Germany; ^2^CAESAR (Center of Advanced European Studies and Research), Bonn, Germany; ^3^Schaefer Technologie GmbH, Langen, Germany; ^4^Orthogen, Düsseldorf, Germany

Email: maria-paz.weisshaar@h-brs.de

Microvesicles (MVs) and exosomes have been shown to be secreted by most cell types and are found as well in biological fluids like serum, urine and synovial fluids. Those MVs have been shown to play an important role not only as potential biomarkers of diseases but as well as potential therapeutic tools. The objective of this study is to show that ACS contains exosomes with an anti-inflammatory activity.

*Methods*: In our study, the production of ACS involves the incubation of the autologous, venous blood in the presence of medical glass beads at 37°C during 24 hours. Exosomes were isolated by known standard procedures using differential ultracentrifugation and sucrose gradient, visualized using a TEM electron microscope and quantifying by NanoSight (NTA) measurements. The protein content was revealed via SDS gel analysis. The anti-inflammatory activity was shown in a controlled equine osteoarthritis model. *Results*: The TEM as well as the NTA analysis showed the highest amount of MV and exosomes (5–29×10^−8^/ml) after an incubation time of 24 h with a mean size over 180 nm showing to be meanly aggregates. *Conclusions*: ACS is known to contain an increased amount of endogenous anti-inflammatory cytokines; our experiments could show that they contain as well a high amount of MV and exosomes that may increase the anti-inflammatory activity.

Funded by: Orthogen AG, G-40210 Düsseldorf

**21. Capture of cell-derived microvesicles (exosomes and apoptotic bodies) by human plasmacytoid dendritic cells**

Patricia Bastos-Amador^1^, Begoña Pérez-Cabezas^1^, Nuria Izquierdo-Useros^2^, Maria C. Puertas^2^, Javier Martinez-Picado^2,3^, Ricardo Pujol-Borrell^1^, Mar Naranjo-Gómez^1^ and Francesc E. Borràs^1^

^1^Laboratory of Immunobiology for Research and Applications to Diagnosis (LIRAD), Blood and Tissue Bank (BST), Department of Cell Biology, Physiology and Immunology, Universitat Autònoma de Barcelona, Institut Investigació Germans Trias i Pujol (IGTP), Badalona (Barcelona), Spain; ^2^Irsicaixa Foundation, HIVACAT; ^3^Institució Catalana de Recerca i Estudis Avançats (ICREA)

Email: patba82@hotmail.com

Conventional and plasmacytoid dendritic cells differ in multiple aspects. Among those, antigen capture is a recognized feature of cDCs while pDCs display poor capacity to capture cell-derived antigens. However, animal models of organ transplantation suggested a role for pDCs in tolerance induction via phagocytosis of donor antigens. In a transplantation setting, microvesicles such as apoptotic bodies and exosomes secreted by the graft may be potential sources of alloantigen. Here, we tested the capacity of human pDCs to capture exosomes and apoptotic bodies from Jurkat T cells. Exosomes and apoptotic bodies were indeed captured by pDCs, although required longer times of incubation when compared to the highly endocytic cDCs. In both cDC and pDC, exosome capture was more efficient than apoptotic bodies. Endocytosis inhibitors clearly impaired exosome capture by cDCs, although this could not be verified in pDCs due to cellular toxicity. Functionally, capture of Jurkat-derived exosomes did not induce nor prevented pDC maturation, and exosome-loaded pDCs induced T cell proliferation, suggesting a link between capture and presentation. Thus, exosomes and apoptotic bodies may be sources of antigen for human pDCs.

Funded by: The “Fondo de Investigaciones Sanitarias” of the Spanish National Institute of Health (FIS 03/0142 and PS 09/00229) to F.E.B. P.B.A. is supported by a grant from the Agència de Gestió d'Ajuts Universitaris i de Recerca (AGAUR) of the Catalan Government (2010FI_B1 00023).

**22. PMN-Ect ingested by macrophages follow the degradation pathway**

Ceylan Eken, Salima Sadallah, and Jürg A. Schifferli

Immunonephrology Laboratory, Department of Biomedicine, University Hospital Basel, Basel, Switzerland

Email: ceylan.eken@unibas.ch

Ectosomes shed by PMN (PMN-Ect) have a size between 50 and 300 nm and express phosphatidylserine as well as a specific selection of cell membrane proteins. When phagocytic cells such as macrophages and dendritic cells encounter PMN-Ect, they lose some of their properties and acquire new phenotypes. Interestingly, extracellular vesicles have been shown to transfer proteins, RNA or various other molecules to the cells, which have ingested them. The uptake mechanism(s) and subsequent intracellular trafficking pathway of exosomes appear to be dependent on the experimental settings. To date, the internalization and trafficking of PMN-Ect have not been described. In this work, human monocyte-derived macrophages were used to track the ingestion and fate of fluorescently labeled PMN-Ect. We performed live-cell confocal microscopy, and the results showed that PMN-Ect did not fuse with the cells but were internalized through an endocytic pathway. Upon ingestion, PMN-Ects were transported to lysosomes labeled with LysoTracker Red DND-99 within 30 minutes, where they stayed trapped for up to 14 hours. The accumulation of PMN-Ect was observed mainly at the perinuclear region. Labeling of both PMN-Ect proteins and lipids showed that they co-localized at the early stages of endocytosis but later started to dissociate (≥10 hours). The analysis using fixed cells confirmed the live-cell microscopy data. We observed the binding of PMN-Ect to cells by labeling the plasma membrane of macrophages using wheat germ agglutinin. In addition, PMN-Ect co-localized with the early endosome markers Rab5 and EEA-1 and finally with the lysosomal-associated membrane protein 2 (Lamp2). There was no co-localization with the Golgi marker Giantin. The long persistence of PMN-Ect inside lysosomes might be explained by the use of resting macrophages. Whether activated macrophages would degrade PMN-Ect more efficiently remains to be studied.

Funded by: Swiss National Science Foundation

**23. Exosomal transfer of RNA-based signals between the hematopoietic system and the brain in response to inflammation**

Kirsten Oesterwind^1^, Sascha Keller^2^, Maria Dams^1^, Jadranka Macas^1^, Britta Landfried^1^, Karl H. Plate^1^, Candan Depboylu^3^, Günter Höglinger^4^, Peter Altevogt^2^ and Stefan Momma^1^

^1^Institute of Neurology (Edinger Institute), University Medical School, Frankfurt, Germany; ^2^Tumor Immunology Program, German Cancer Research Center (DKFZ), Heidelberg, Germany; ^3^Philipps University Marburg, Clinic for Neurology, Marburg, Germany; ^4^German Center for Neurodegenerative Diseases e.V. (DZNE), Technical University Munich (TUM), Germany

Email: kirsten.oesterwind@kgu.de

The influence of peripheral inflammation to the central nervous system is highly discussed in the context of different diseases. However, the exact mechanism of this influence still remains unclear. To address the question of a hematopoietic contribution to neurons, we used a transgenic mouse model expressing the Cre recombinase specifically in the hematopoietic compartment. Subsequent irreversible labeling of the recombined cells allows for tracing any contribution to other cell populations. Interestingly, analysis of cerebellar tissue reveals reporter gene induction in non-hematopoietic cells, specifically in Purkinje neurons, without indication for cell fusion. We provide evidence that reporter gene expression is caused by intercellular transfer of functional Cre recombinase messenger RNA contained in secreted membrane vesicles, particularly in exosomes. We could show that exosomal preparations from Cre expressing hematopoietic cells are sufficient to induce recombination of Purkinje neurons when injected directly into cerebellar tissue. Furthermore, recombination of Purkinje neurons increases significantly after inducing inflammatory injuries. Recombination events are not restricted Purkinje neurons but include other neuronal populations in different areas of the brain. These observations reveal the existence of a previously unrecognized way to communicate RNA-based signals between the immune system and the brain after peripheral inflammation.

Funded by: Edinger Foundation; Deutsche Forschungsgesellschaft (DFG)

#### 2. Cancer and Cancer Progression

**24. Poster 24 is withdrawn**

**25. Exosomal tumor microRNA modulates premetastatic organ cells**

Margot Zöller and Sanyukta Rana

Department of Tumor Cell Biology, University Hospital of Surgery, Heidelberg, Germany

Email: margot.zoeller@uni-heidelberg.de

Exosomes, potent intercellular communicators, are supposed to contribute to metastasis formation. As exosomes of the metastatic rat pancreatic adenocarcinoma line BSp73ASML promote metastatic settlement in lymph nodes and lung, we profiled BSp73ASML and BSp73ASML-CD44vkd exosomes, the latter line having largely lost the capacity to metastasize, and defined the molecular pathway(s), whereby exosomes might contribute to premetastatic niche preparation. BSp73ASML and BSP73ASML-CD44vkd exosomes show similar mRNA profiles but differ strongly in the microRNA composition, tumor growth suppressing microRNA being particularly enriched in BSp73ASML-CD44vkd exosomes. BSp73ASML more readily than BSP73ASML-CD44vkd exosomes are recovered in the draining lymph node after subcutaneous injection. The exosomes preferentially bind and are taken up by lymph node stroma cells (LnStr) and lung fibroblasts. Exosome-uptake is accompanied by significant changes in gene expression. Both exosomal mRNA and microRNA are recovered in the target cells, but changes in gene transcription and/or translation mostly rely on transferred microRNA, demonstrated, among others, for miR-494 and miR-542-3p, abundant in BSp73ASML exosomes and known to target MAL (myelin and lymphocyte protein)/cadherin17 and TRAF4/cadherin17, respectively. MAL, cadherin17 and TRAF4 were downregulated in exosome-treated or miRNA-transfected LnStr. Furthermore, MMP transcription, suggested to accompany cadherin17 downregulation, was upregulated in miR-494/miR-542-3p transfected or exosome co-cultured LnStr. Notably, the majority of LnStr mRNA significantly affected by ASML exosomes/microRNA, relates to proteolysis, adhesion/motility, angiogenesis, oxidative stress response and proliferative activity. Taken together, tumor exosomes target in vivo non-transformed cells in premetastatic organs. Exosomal proteins contribute to target cell selection. Severely altered target cell gene expression is mostly promoted by exosomal microRNA, where we demonstrate for the first time that exosomal microRNA from a metastasizing tumor line modulates stroma cells from premetastatic organs toward favoring metastasizing tumor cell settlement and growth.

Funded by: German Research Foundation

**26. Tumor cell-derived microvesicles enhance human breast cancer invasion through p38-MAPK**

K. Menck, F. Klemm, T. Pukrop, M. Schulz and C. Binder

Department of Hematology and Oncology, University of Göttingen, Göttingen, Germany

Email: Kerstin.Menck@med.uni-goettingen.de

The interaction of human cancer cells with the adjacent tumor stroma is critical for tumor progression. Recent findings suggest that especially the intercellular communication via extracellular vesicles plays an important role in the establishment of a favorable tumor niche. However, little is known about the molecular mechanism of these interactions. We were able to confirm the presence of plasma membrane-derived microvesicles (T-MV) in the supernatant of the human breast cancer cell line MCF-7 under basal conditions by electron microscopy. In a modified Boyden chamber assay T-MV were able to enhance MCF-7 invasiveness by 365%, whereas MV from outdated platelet concentrates (P-MV) as well as MV from the benign human mammary epithelial cell line hTERT-HME1 (hTERT-MV) did not show any effect. As a candidate protein to mediate the proinvasive phenotype of T-MV, we found the MMP inducer EMMPRIN to be enriched in its highly glycosylated form on T-MV but not on hTERT-MV or P-MV. However, after T-MV stimulation, we could not detect any changes in MMP synthesis neither in MCF-7 nor in human macrophages, which represent an important component of the tumor microenvironment. Instead, a phosphorylation of the EMMPRIN target protein p38-MAPK could be detected in both cell types 1 hour after T-MV stimulation. The p38 inhibitor SB-203580 was able to reduce the T-MV-mediated increase in MCF-7 invasiveness. These results indicate that the proinvasive MV phenotype is specifically attributed to distinct MV subpopulations. Highly glycosylated EMMPRIN is a promising molecule to partially mediate the proinvasive phenotype of T-MV in a MMP-independent way through p38 signal transduction.

Funded by: Deutsche Krebshilfe (German Cancer Aid)

**27. Can exosomes influence triple negative breast cancer metastasis?**

K. O'Brien^1^, S. Rani^1^, R. Wallace^1^, S. McDonnell^2^, L. Hughes^2^, M. Radomski^1^ and L. O'Driscoll^1^

^1^School of Pharmacy & Pharmaceutical Sciences, Trinity College, Dublin, Ireland; ^2^School of Chemical and Bioprocess Engineering, University College, Dublin, Ireland

Email: obrienk9@tcd.ie

Triple-negative breast cancer (TNBC) is associated with high mortality rates and incidence in younger women. Our analysis primarily investigated the relevance of exosomes in TNBC, by comparing the effects of exosomes derived from Hs578T and its more invasive subclone, Hs578Ts(i)8, as well as exosomes derived from TNBC compared to control sera. The effects of exosomes were analyzed on secondary cell proliferation, motility, invasion, anoikis and endothelial tubule/vessel formation. Hs578Ts(i)8 exosomes, compared to Hs578T exosomes, conferred increased proliferation, motility and invasion on three studied breast cancer cell lines, as well as Hs578Ts(i)8-derived exosomes inducing Hs578T cells to me more invasive. Additionally, Hs578Ts(i)8-derived exosomes stimulated greater tubule formation than Hs578T derived exosomes. However, Hs578TS(i)8-derived exosomes, compared to Hs578T exosomes, sensitized the three cell lines studied to anoikis, a finding consistent with the innate phenotype of both cell Hs578T variants. Furthermore, exosomes isolated from TNBC patients’ serum, compared to those from control sera, increased the invasiveness of SKBR3 cells. Further investigation discovered the presence of RNA in serum-derived exosomes, supporting the potential for exosomes as cancer biomarkers. Our analyses indicate that the exosomes influence secondary cells in a manner indicative of the innate phenotypes of their donor cells, as well as having the potential as cancerous biomarkers.

Funded by: The Marie Keating Foundation; Trinity College Postgraduate Award and Science Foundation Ireland's funding of Strategic Research Cluster, Molecular Therapeutics for Cancer, Ireland

**28. The role of exosome in brain metastasis of breast cancer**

Makiko Ono, Fumitaka Takeshita, Nobuyoshi Kosaka and Takahiro Ochiya

Division of Molecular and Cellular Medicine, National Cancer Center Research Institute, Tokyo, Japan

Email: makikono@gmail.com

*Background*: Exosome acts as a mediator of intercellular communication between not only normal cells but also cancer cells. Although exosome derived from cancer cells is found to play an important role in progression and the formation of premetastatic niche, precise mechanism has been uncertain in brain metastasis, especially involvement in pass-through the blood brain barrier (BBB). *Methods*: We studied uptake of breast cancer cell-derived exosomes into cells constructing BBB, such as astrocyte, pericyte and brain vascular endothelial cells. Next, we studied BBB permeability of exosome using in vitro BBB model. Then, we compared microRNAs included in exosomes derived from cancer cells with high and low permeability of BBB. *Results*: Among cells composing BBB, exosome was differently taken into cells. In astrocyte and brain vascular endothelial cells, selective uptake of exosome was observed. Permeability of breast cancer-derived exosome was not sufficient to penetrate in vitro BBB model. *Conclusions*: Exosome differently affected cells comprising BBB. Further analysis would be necessary to ascertain possible role of exosomes in brain metastasis.

Funded by: A Grant-in-aid for the Third-Term Comprehensive 10-Year Strategy for Cancer Control, a Grant-in-Aid for Scientific Research on Priority Areas Cancer from the Ministry of Education, Culture, Sports, Science and Technology

**29. Exosomes in bladder cancer**

D.K. Jeppesen^1^, S.G. Jensen^1^, J.P. Morth^2^, M.R. Larsen^3^, L. Dyrskjøt^1^, T. Ørntoft^1^ and M.S. Ostenfeld^1^

^1^Department of Molecular Medicine, Aarhus University Hospital, Denmark; ^2^Centre for Molecular Medicine Norway, Oslo, Norway; ^3^Institute for Biochemistry and Molecular Biology, University of Southern Denmark, Denmark

Email: dennis.jeppesen@ki.au.dk

Bladder cancer is a common malignancy in men in economically advanced nations. In various cancers, exosomes of tumor origin have been detected in plasma or serum from patients. Circulating exosomes contain a large number of proteins and lipids as well as mRNA and miRNA that can impact recipient cells upon cellular entry of exosomes. Bladder cancer cell lines FL3 and T24 with and without metastatic potential, respectively, were cultivated in CELLine AD 1000 Bioreactors for increased exosome yield. Exosome isolation was conducted by ultracentrifugation. Exosomes were characterized by Western blotting and transmission electron microscopy. Western blotting revealed that the isolated membrane vesicles were very highly enriched for the exosomal markers CD9 and CD81. Electron microscopy confirmed the identification of secreted T24 and FL3 exosomes with a mean size of 65.8±13.7 nm and 65.3±19.4 nm in size, respectively. An approximately 10-fold increase in exosome yield was observed when cells were cultivated in bioreactors compared to cultivation in conventional cell culture flasks. The expression of 740 miRNAs in secreted exosomes and parental cells were profiled using an ABI q-RT PCR platform. Specific miRNAs were enriched in exosomes from tumor cells as opposed to normal urothelial cells and in exosomes from metastatic bladder cancer cells. Future work will focus on the transfer of exosomal miRNA into recipient cells and the functional consequences.

Funded by: Aarhus University and private funds

**30. Exosomes as biomarkers for prostate cancer progression and drug resistance**

Carolina Soekmadji, Colleen C. Nelson and Pamela J. Russell

Australian Prostate Cancer Research Centre-Queensland, and Cells and Tissue Domain, Institute of Health and Biomedical Innovation, Queensland University of Technology, Brisbane, Queensland, Australia

Email: carolina.soekmadji@qut.edu.au

Over 20,000 Australian men are diagnosed with prostate cancer (PC) with more than 3500 deaths/year from this disease. Worldwide, 910,000 cases were recorded in 2008, accounting for 14% of men's cancer, and these figures are predicted to reach 1.7 million by 2030. Early PC is treated by surgery or radiation; androgen depletion therapy (ADT) is subsequently used for those who fail treatment, as PC is androgen regulated. Among these, 25–40% of cases develop castrate-resistant PC (CRPC) with a rising PSA, an androgen-regulated gene, despite low androgen levels in the serum. The underlying mechanisms for progression to CRPC are complex. PC cells that survive ADT arrest adapt to a low-androgen environment through various mechanisms, which maintain androgen receptor (AR) signaling and continue to proliferate. The chemotherapeutic drug, docetaxel, is commonly used to treat CRPC patients in the clinic, but ∼30% of patients who receive docetaxel therapy relapse and suffer from severe side effects. Biomarkers, which could define whether patients will respond to ADT and drug treatments, are needed. We believe that exosomes may provide novel biomarkers that will alter with treatment, potentially providing markers that reflect PC progression and drug-resistant disease. We investigated the effects of DHT treatment on exosome secretion using AR positive LNCaP and 22RV1 cell lines as an in vitro PC model. DHT changes the protein profile of exosomes isolated from both cell lines, implying that in PC, the AR plays a role in selecting the content of exosome and confirming that exosome analysis has the potential to provide novel biomarkers for PC progression and drug resistance.

Funded by: Department of Defense US Army Prostate Cancer Program Postdoctoral Training Award, and Queensland University of Technology

**31. The biological and functional role of prostate cancer exosomes**

D. Duijvesz, N. Naimi, M.E. van Royen and G. Jenster

Department of Urology and Pathology, Erasmus Medical Center, Rotterdam, The Netherlands

Email: d.duijvesz@erasmusmc.nl

*Introduction*: Until now, the exact biological function of exosomes (in prostate cancer) remains unknown. With functional experiments, we show how exosomes interact with recipient prostate (cancer) cells and what effect they have on those cells. *Material and methods*: Exosomes were isolated by ultracentrifugation from five different cell lines (PNT2C2, VCaP, PC346C, DU145N and DU145N expressing GFP). Confocal microscopy and 4PI microscopy were performed to study whether exosomes fuse with the membrane of recipient cells or are taken up by endocytosis. Western blotting was carried out to show transfer of specific epithelial proteins (CD24 and multiple keratins) to stromal cells (PrSC) by exosomes. With migration and MTT-assays, we show the biological effect of exosomes from malignant cell lines on non-cancerous prostate cells (PNT2C2). *Results*: After fluorescent labeling of exosomal membranes, confocal microscopy and 4PI microscopy showed uptake of whole exosomes rather than fusion of exosomes with the membrane. So far, no protein transfer has been shown with intraexosomal GFP proteins or by Western blotting. Growth and migration the PNT2C2 cell line was not altered by VCaP exosomes. *Discussion and conclusion*: Exosomes seem to be taken up by cells via endocytosis. So far, no transfer of specific proteins by exosomes could be observed. More sensitive experiments (such as PCR) have to be performed in order to show the transfer of cellular content by exosomes in prostate cancer.Funded by: ErasmusMC Grant

**32. Exosomes—potential regulators and biomarkers of prostate cancer progression**

Claire Corcoran^1^, Sweta Rani^1^, Keith O'Brien^1^, Amanda O'Neill^2^, Maria Prencipe^2^, John Crown^3^, William Watson^2^ and Lorraine O'Driscoll1

^1^School of Pharmacy & Pharmaceutical Sciences, Trinity College, Dublin, Ireland; ^2^UCD School of Medicine and Medical Science, University College, Dublin, Ireland; ^3^Molecular Therapeutics for Cancer, Ireland

Email: corcorcl@tcd.ie

Hormone-refractory prostate cancer treatment remains hindered by inevitable progression of resistance to first-line treatment with docetaxel. To help determine the complexity of this problem, in vitro cell line models of docetaxel resistance were established representative of the clinical situation. This study aimed to (i) isolate exosomes from medium conditioned by these cell line models and investigate their effects on motility, invasion, proliferation and docetaxel resistance of secondary cells; (ii) perform a proof-of-principle translational investigation of the clinical relevance of exosomes isolated from prostate cancer serum; and (iii) perform microRNA profiling on cells and their corresponding exosomes to determine intracellular and extracellular biomarkers predictive of response to docetaxel. Exosomes expelled from DU145 docetaxel-resistant variant (DU145RD) conferred docetaxel-resistance to both DU145 and 22Rv1 cells, which may be partly due to exosomal MDR-1/P-gp transfer. Furthermore, exosomes from prostate cancer patient sera increased cell proliferation and invasion, compared age-matched controls. Finally, miRNA profiling studies revealed a panel of miRNAs common between cells and exosomes that may offer potential as biomarkers predictive of response to docetaxel. Our in vitro observations and preliminary clinical studies indicate that exosomes play an important role in prostate cancer and may offer potential as vehicles containing predictive biomarkers and new therapeutic targets.

Funded by: Science Foundation Ireland's Strategic Research Cluster award to Molecular Therapeutics for Cancer Ireland (08/SRC/B1410)

## Scientific Program 2012 ISEV meeting Saturday 21st April

### Symposium Session 10            8.30-10.00

#### 1. Extracellular Vesicles in Cancer

**Characterization and proteomic analysis of neuroblastoma-derived exosomes**

D. Marimpietri^1^, A. Petretto^2^, M.C. Gagliani^3^, C. Tacchetti^3^, G. Melioli^4^ and V. Pistoia^1^

^1^Laboratory of Oncology and ^2^Core Facilities, Gaslini Children's Hospital, Genoa, Italy; ^3^DIMES3, University of Genoa, Genoa, Italy; ^4^Analysis Laboratory, Gaslini Children's Hospital Genoa, Italy

Email: danilomarimpietri@ospedale-gaslini.ge.it

Neuroblastoma (NB) is the most common extracranial solid tumor in childhood whose prognosis is grim in a half of patients. Exosomes are nanometer-sized membrane vesicles derived from the multivesicular bodies (MVBs) of the endocytic pathway and released by normal and neoplastic cells. In this study, we isolated exosomes from the conditioned media of HTLA-230 human NB cells by ultrafiltration and ultracentrifugation. Electron microscopy demonstrated that NB-derived exosomes exhibited the characteristic cup-shaped morphology. We measured size distribution by dynamic light scattering, showing a bell-shaped curve with a peak at 130 nm. Zeta potential was −32 mV, suggesting a good nanoparticle stability. We performed the first proteomic analysis of NB-derived exosomes with a 2DLC separation and MS/MS analyses using the MudPIT strategy. We found that nearly 70% of the proteins identified in NB-derived exosomes are present in Exocarta database including tetraspanins, heat shock proteins, MVBs proteins and cytoskeleton related proteins. The presence of tetraspanins (CD9, CD63 and CD81) and some intriguing cell-specific proteins such as CD133, CD276 and CD147 was confirmed by flow cytometry after vesicle adsorption onto latex beads. Noteworthy, flow cytometric analysis showed that NB-derived exosomes highly expressed GD2 disialoganglioside, one of the most specific markers of NB disease. Moreover, we developed an ELISA protocol aimed at detecting the presence on exosomes of the GD2 molecule. In conclusion, this study shows that NB-derived exosomes express a discrete set of proteins involved in defense response, cell differentiation, cell proliferation and the regulation of other important biological process, suggesting that NB-derived exosomes may play an important role in the modulation of tumor microenvironment and may provide potential diagnostic biomarkers.

Funded by: Ricerca Corrente, Ministero della Salute

**Rab27B, an ex(o)citing driver of breast cancer growth, invasion and metastasis**

An Hendrix^1^, Wendy Westbroek^2^, Marc Bracke^1^ and Olivier De Wever^1^

^1^Laboratory of Experimental Cancer Research, Department of Radiation Oncology and Experimental Cancer Research, Ghent University Hospital, Ghent, Belgium; ^2^Medical Genetics Branch, National Human Genome Research Institute, Bethesda, MD, USA

Email: an.hendrix@ugent.be

Cancer cells implement various exocytic routes, modulated by small Rab GTPases, to relay crucial information for fostering growth, invasion and matrix degradation. We investigated the biological role and expression status of Rab27B, a regulator of exosome release, in breast cancer. Rab27B-upregulation in estrogen receptor (ER)-positive breast cancer cells promoted G1/S phase cell cycle transition and increased proliferation, F-actin reorganization and invasion in cell culture and invasive tumor growth and hemorrhagic ascites in a xenograft mouse model. Proteomics of purified Rab27B vesicles and the secretome of Rab27B-expressing breast cancer cells were identified HSP90α as key proinvasive growth regulator. HSP90α secretion occurred in a Rab27B-dependent manner and was required for MMP-2 activation. Endogenous Rab27B mRNA and protein, but not Rab3D and Rab27A mRNA, was associated with lymph node metastasis and differentiation grade in ER-positive breast cancer samples. In conclusion, Rab27B regulates invasive growth and metastasis in ER-positive breast cancer cell lines, and increased expression is associated with poor prognosis in humans. Because of the relationship between Rab27B and cancer progression, elucidating the role of exosomes in metastatic niche formation will be the next step forward in cancer research.

Funded by: Intramural Research Program of the National Human Genome Research Institute and Special Research Fund Flanders.

**Influence of oncogenic HPV-types on the composition and function of exosomes**

Anja Honegger, Karin Hoppe-Seyler Felix Hoppe-Seyler

Molecular Therapy of Virus-Associated Cancers, German Cancer Research Center, Heidelberg, Germany

Email: a.honegger@dkfz.de

Oncogenic human papillomavirus (HPV) types, most notably HPV16 and HPV18, are closely linked to the development of several human cancers. Recent findings indicate that tumor viruses can employ exosomes as intercellular mediators to deregulate cellular growth. A connection between HPVs and exosomes has not yet been investigated. To address this issue, our work aims to study the influence of the major transforming oncogenes of HPV, E6 and E7, on the composition (exosomal proteins, mRNAs, non-coding RNAs) and function of exosomes produced by HPV-positive cells. As experimental strategy, the viral E6 and E7 expression will be specifically blocked in HPV18-positive HeLa cells by RNA interference. Next, it will be investigated whether silencing of viral oncogene expression modulates the amount and content of exosomes secreted by HPV-positive tumor cells. In specific, exosomal RNAs will be identified by high-throughput RNA sequencing and exosomal proteins will be analyzed by protein arrays. In a future part of this project, it also will be examined whether HPV-modified exosomes are taken up by HPV-negative cells and whether they, thereby, can modulate cellular pathways within recipient cells. In summary, the intention of our work is to provide novel insights into the transforming activities of oncogenic HPVs and into the relevance of exosomes as vehicles for the intercellular communication of HPV-positive tumor cells. Furthermore, since exosomes can be readily isolated from body fluids, the identification of HPV-associated exosomal markers could possess diagnostic potential.

**Micro-RNA signatures of exosomes derived from melanoma cells**

Florian S. Dreyer, Jochen Dindorf, Nina Koliha, Stefan Wild and Andreas S. Baur

Universitätsklinikum Erlangen, Department of Dermatology, Hartmannstraße, Erlangen, Germany; Miltenyi Biotec, Bergisch, Gladbach, Germany

Email: Florian.Dreyer@uk-erlangen.de

New treatment regimens for cancer require easily accessible biomarkers preferably in peripheral blood of patients. Circulating tumor microvesicles/exosomes are believed to contain cancer-specific protein-, mRNA- and miRNA profiles; however, their relevance for cancer diagnosis and treatment remains to be demonstrated. Here, we analyzed the total RNA profile and miRNA signatures of exosomes isolated from supernatants of different primary melanoma cell lines. Analysis of the total RNA profile revealed the selective accumulation of miRNAs and short mRNA molecules in a manner resembling other cancer miRNA profiles. miRNA profiles from tumor microvesicles and corresponding cell lines will be presented. Using RT-PCR, we could amplify selected miRNAs and, thus, confirm our in vitro results. These miRNAs were not detectable in healthy individuals. Taken together, our results indicate that miRNA profiles in tumor exosomes could serve as an easily accessible diagnostic and/or prognostic marker in individualized cancer treatment.

Funded by: Hanns Seidel Foundation Deutsche Forschungsgemeinschaft Miltenyi Biotec GmbH.

**The tumor suppressor PTEN is transported in exosomes for extracellular phosphatase activity**

Ulrich Putz, Anh Doan and Seong-Seng Tan

Florey Neuroscience Institutes and Centre for Neuroscience, The University of Melbourne, Parkville, Victoria, Australia

Email: uputz@unimelb.edu.au

Exosomes are intercellular messengers with the capacity to alter the internal physiological states of target cells. We demonstrate that PTEN, a tumor suppressor protein normally localized in the cytoplasm and nucleus, can be secreted in exosomes. Exosomes containing secreted PTEN can be internalized by recipient cells with resultant functional activity of PTEN, exhibited by reduced pAkt and recipient cell proliferation. PTEN secretion in exosomes requires Ndfip1, an adaptor for Nedd4-family ubiquitin ligases, and absence of Ndfip1 abolishes exosomal trafficking of PTEN. Without Ndfip1, neither Nedd4-1 nor Nedd4-2 is able to promote PTEN recruitment into exosomes. In addition, a mutation of a key lysine residue K13, previously identified in PTEN to be important for ubiquitination by Nedd4-1, is required for exosomal transport. These results identify Ndfip1, a key member of the Nedd4 ubiquitination pathway, to be an important molecular regulator for exosomal export of PTEN, with consequences for non-cell autonomous PTEN activity. Therefore, the ability of exosomal PTEN to exert phosphatase activity after exosome uptake has significant implications for PTEN and exosomes function during development, health and disease.

Funded by: NHMRC

#### 2. Late Breaking Abstracts oral session

### Coffee          10.00-10.30

### Symposium Session 11          10.30-12.00

#### Late Breaking Abstracts oral session

### Closing Ceremony & Abstract Prizes          10.30-12.00

